# Effect of restricted dissolved oxygen on expression of *Clostridium difficile* toxin A subunit from *E. coli*

**DOI:** 10.1038/s41598-020-59978-1

**Published:** 2020-02-20

**Authors:** Ashish K. Sharma, Jenie Phue, Emir Khatipov, Nimish Dalal, Eric D. Anderson, Joseph Shiloach

**Affiliations:** 1 Biotechnology Core Laboratory, National Institute of Diabetes and Digestive and Kidney Diseases, National Institutes of Health, Bethesda, MD USA; 2 Present Address: Center for Biologics Evaluation and Research, U.S. Food and Drug Administration, Silver Spring, MD USA; 3 National Institutes of Health Library, Division of Library Services, Office of Research Services, National Institutes of Health, Bethesda, MD 20892 USA; 4 Biologics manufacturing Science & Technology Bristol-Myers Squibb East Syracuse, New York, NY 13057 USA; 5 Mass Spectrometry Facility, National Institute of Diabetes and Digestive and Kidney Diseases, National Institutes of Health, Bethesda, MD USA; 6 Present Address: National Institute of Dental and Craniofacial Research, National Institutes of Health, Bethesda, MD 20817 USA

**Keywords:** Expression systems, Industrial microbiology

## Abstract

The repeating unit of the *C. difficile* Toxin A (rARU, also known as CROPS [combined repetitive oligopeptides]) C-terminal region, was shown to elicit protective immunity against *C. difficile* and is under consideration as a possible vaccine against this pathogen. However, expression of recombinant rARU in *E. coli* using the standard vaccine production process was very low. Transcriptome and proteome analyses showed that at restricted dissolved oxygen (DO) the numbers of differentially expressed genes (DEGs) was 2.5-times lower than those expressed at unrestricted oxygen. Additionally, a 7.4-times smaller number of ribosome formation genes (needed for translation) were down-regulated as compared with unrestricted DO. Higher rARU expression at restricted DO was associated with up-regulation of 24 heat shock chaperones involved in protein folding and with the up-regulation of the global regulator RNA chaperone *hfq*. Cellular stress response leading to down-regulation of transcription, translation, and energy generating pathways at unrestricted DO were associated with lower rARU expression. Investigation of the *C. difficile* DNA sequence revealed the presence of cell wall binding profiles, which based on structural similarity prediction by BLASTp, can possibly interact with cellular proteins of *E. coli* such as the transcriptional repressor ulaR, and the ankyrins repeat proteins. At restricted DO, rARU mRNA was 5-fold higher and the protein expression 27-fold higher compared with unrestricted DO. The report shows a strategy for improved production of *C. difficile* vaccine candidate in *E. coli* by using restricted DO growth. This strategy could improve the expression of recombinant proteins from anaerobic origin or those with cell wall binding profiles.

## Introduction

*Clostridium difficile* (*C. difficile*) is an anaerobic bacterial pathogen responsible for diarrhea and pseudomembranous colitis^1,2^. The bacteria produces two high molecular weight toxins, toxin A (308 kDa) and toxin B (269 kDa)^3,4^; both contain a repeating region called rARU (CROPs) (104 kDa) in subunit A and rBRU (70 kDa) in subunit B^5,6^. Both Toxin A and Toxin B are responsible for clinical symptoms and are candidates for vaccine development^7,8^. Pfizer’s genetically detoxified vaccine against C difficile is a toxoid mix of Toxin A and Toxin B expressed recombinantly is about to enter clinical trial phase III, and Valneva has a vaccine candidate of recombinant fusion protein of truncated portions of Toxin A and Toxin B, which completed a clinical trial phase II, with promising results^9–12^.

In this report, we focused on improving the expression of the repeating unit region (rARU) of the *C. difficile* toxin A. The importance of rARU was shown in animal models where serum neutralizing antibodies to toxin A conferred immunity to this pathogen and antiserum against nontoxic recombinant peptide, containing the repeating units region (rARU), neutralized the enterotoxic and cytotoxic activity of the toxin^13,14^. This independent repeating unit region was also found to be an efficient carrier for conjugated polysaccharide vaccines^15,16^, and as a result of this observation, the efficient expression of the subunits of toxins A and B for clinical experiments is needed.

The expression of Clostridial proteins in *E. coli* is poor^17,18^, and is likely due to high AT content that may form accidental polyadenylation sites that cause truncation of mRNA^19,20^. In addition, the presence of rarely used codons for arginine (AGA) and leucine (ATA) may affect the completion of the translation and cause the expression of truncated proteins^18,21–23^. Improved expression was achieved by implementing different strategies such as inserting sequences used frequently in *E. coli* for ATA and AGA tRNAs^18^, reducing growth temperature^24,25^, introducing chaperones, reducing acetate expression, and introducing rare amino acids and codon optimization^24,26,27^. An overlooked option is the possibility of an interaction between the expressed recombinant proteins and the host cellular machinery (e.g., RNA degradation and inhibition of cell growth caused by the expression of human RNase L containing “ankyrin repeat” motifs in *E. coli*)^28^.

The issue of lower rARU yield could also be related to the unique sequence and the structural motifs present in the Clostridial DNA. It was recently shown that certain recombinant proteins (e.g., proteins of a different nature like soluble (xylanase and GFP) or inclusion body (Interferon β)) based on cellular localization (e.g., secreted proteins (NprE, XynA, Usp45, TEM-1 β-lactamase), membrane proteins (LmrA and XylP), or lipoproteins (MntA and YcdH)) caused different host cellular responses when overexpressed^29,30^. It is possible by using next generation sequencing tools to quantify the gene expression profile at different growth and production phases in order to identify changes related to substrate consumption, central carbon metabolism, and energy metabolism based on the sequence and structural features. Here we report that expressing C. *difficile* rARU from *E. coli* in a restricted dissolved oxygen (DO) supply condition is 27-fold higher than expression obtained when regular conditions of unrestricted DO are implemented. We hypothesized that the presence of cell wall binding profiles in the rARU is the cause. Log and late-log phase samples from cultivations at restricted and unrestricted DO were investigated by using transcriptomics and proteomics. The information obtained suggests that the cell wall binding segments of the rARU have a role in gene expression by interacting with the cellular *E. coli* proteins.

## Results

### *E. coli* growth and rARU expression at restricted and unrestricted DO

**Figure 1:**
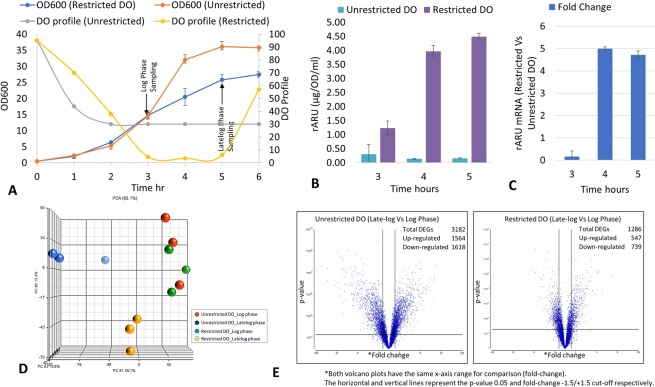
Bioprocess parameters, culture kinetics, rARU production and data processing to identify DEGs. (**A**) Growth (OD_600_) and DO profile of rARU expressing recombinant BL21(DE3)Tuner/pRSETb-rARU at unrestricted DO and restricted DO condition; (**B**) rARU expression in ng/100 µl of extracted intracellular fraction from 1 OD (corrected) culture of 3, 4, and 5 hours of cultures growing at unrestricted DO and restricted DO condition; (**C**) mRNA fold change estimation of 3, 4, and 5 hours samples; (**D**) Principal component analysis (PCA) of the microarray data; (**E**) Volcano plot showing distribution of up- and down-regulation genes at unrestricted and restricted DO conditions with the p-value 0.05 and fold-change −1.5/+1.5 cut-off.

Recombinant *E. coli* BL21(DE3) containing pRSETb-rARU plasmid, was grown in Modified Terrific Broth containing glycerol, at restricted and unrestricted (30% saturation) DO. At unrestricted DO, the culture reached late-log phase at OD_600_ of ~36 after ~5 hours, while at restricted DO the culture continued to grow and reached an OD_600_ of ~27 after ~7.5 hours (Fig. 1A). Maximum rARU expression of 4.36 µg/optical density (OD) was obtained when the cells grew in restricted DO, and 0.16 µg/OD when the culture grew at unrestricted DO (Fig. 1B). Transcription levels of rARU at restricted and unrestricted DO are shown in Fig. 1C. After 3 hours of growth, rARU transcription at restricted DO was only 17% compared with the transcription at unrestricted DO. However, after 4 and 5 hours of growth, it was 500%- and 472%- higher as compared with the unrestricted DO culture.

### Microarray analysis of gene expression in *E. coli* producing rARU at restricted and unrestricted DO

Samples in triplicate from *E. coli* producing rARU were collected from cultures grown at restricted and unrestricted DO for profiling gene expression by microarray. Samples were taken after 3 hours when the cells were at the log phase, and at 5 hours when the cells were at the late-log phase which is when rARU expression stopped (Fig. 1A,B). Principal component analysis (PCA) performed on the microarray showed good correlation among the replicates (Fig. 1D). The PCA data demonstrated that samples collected at log phase from cultures grown in restricted and unrestricted DO were closer to each other, whereas, a significant difference was observed among the late-log phase samples collected from the cultures grown in restricted and unrestricted DO.

**Figure 2:**
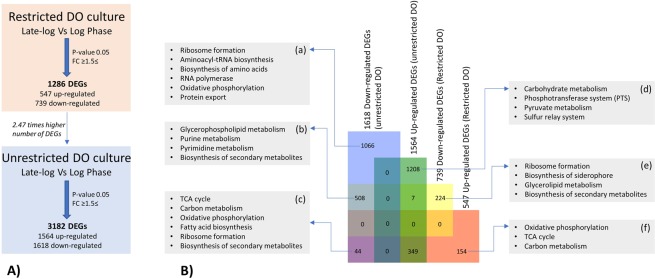
Distribution of DEGs among the growth phases at restricted and unrestricted DO. (**A**) DEGs obtained from ANOVA among late-log and log phase samples at restricted DO condition (late-log phase vs log phase) and unrestricted DO condition (late-log phase vs log phase) and enrichment analysis of obtained gene sets. (**B**) Venn categorization of DEGs and top enriched pathways identified in selected gene sets representing: (a) gene set only down-regulated at unrestricted DO condition; (b) common gene set down-regulated at unrestricted DO and restricted DO condition; (c) gene set down-regulated at unrestricted DO condition but up-regulated at restricted DO; (d) gene set only up-regulated at unrestricted DO condition; (e) gene set only down-regulated at restricted DO; and (f) gene set only up-regulated at restricted DO.

Gene expression at the late-log phase was compared with gene expression at the log phase at both restricted and unrestricted DO by performing a 1-way ANOVA and the results presented by Volcano plot analysis^31^. Figure 1E showed a higher distribution of gene expression at unrestricted DO as compared with restricted DO. By implementing filtration cutoff of log_2_ fold change of ≥1.5 ≤ and by using a p-value of ≤0.05, 3182 DEGs (late-log vs log) were identified at unrestricted DO, and 1286 DEGs were identified at the restricted DO. From the 3182 genes identified as differentially expressed at the unrestricted DO, 1564 were up-regulated and 1618 were down-regulated. From the 1286 DEGs identified at the restricted DO concentration, 547 were up-regulated and 739 were down-regulated (Supplementary Table 1). The culture grown at restricted DO showed a relatively stable transition from log to late-log phase as the unrestricted DO culture showed a 2.5-times greater number of DEGs in transition from log to late-log phase (Fig. 2A).

### Gene enrichment of DEGs from venn analysis

The up-regulated and down-regulated gene sets from the restricted and unrestricted aeration were analyzed using Venn diagram. When grown at unrestricted DO, 1564 DEGs were found to be up-regulated, and of these, 1208 were differentially expressed only in the unrestricted DO. While 349 of the 1564 DEGs were also found to be up-regulated at restricted DO, only 7 up-regulated DEGs were found to be down-regulated at restricted DO. A significantly lower number of genes were differentially expressed uniquely in the restricted DO culture; only 224 were down-regulated and 154 were up-regulated (Fig. 2B).

Gene enrichment analysis was conducted on the gene sets extracted from the Venn analysis and only pathways with a p-value < 0.05 were considered (Supplementary Table 2). The 1208 genes that were up-regulated at unrestricted DO were associated with carbohydrate metabolism, pyruvate metabolism, iron sulfur cluster, sulfur relay system, osmotic stress, and oxidative stress. yggE is a periplasmic protein, which is associated with the inner membrane and found to up-regulate under oxidative stress^32^. See Supplementary Table 1 for the complete list of pathways with their enrichment scores and p-values as an outcome of gene enrichment analysis. The 349 up-regulated DEGs that were identified in both unrestricted and restricted DO were involved in catabolic processes (e.g., organic acids, amino acids, small molecules), fatty acid metabolism, Tricarboxylic acid cycle (TCA), and amino acid degradation. Seven up-regulated DEGs out of the 1564 were down-regulated in restricted DO. Of these 7, 5 genes were related to membrane formation.

From the 1618 differentially down-regulated genes that were identified in the unrestricted DO culture, 1066 were down-regulated only in unrestricted DO (and not in the restricted DO). Therefore, it is possible that the lower activities of these DEGs were associated with the lower expression of the protein at unrestricted DO. Most of these 1066 DEGs were related to cellular processes like ribosome formation (52 DEGs with an enrichment score of 26.9), aminoacyl-tRNA biosynthesis (19 DEGs with an enrichment score of 13.2), biosynthesis of amino acids (48 DEGs with an enrichment score of 5.8), RNA polymerase (4 DEGs with an enrichment score of 5.8), energy generating processes such as oxidative phosphorylation (21 DEGs with an enrichment score of 5.8), and protein export (10 DEGs with an enrichment score of 3.5) were significantly down-regulated. This list of 1066 DEGs indicated that translation, amino acid biosynthesis, and energy generating processes were affected at the unrestricted DO condition since all these functions were down-regulated.

From the same group of 1618 differentially down-regulated genes identified in the unrestricted DO, 508 were down-regulated in both unrestricted and restricted DO cultures. These DEGs were associated with glycerophospholipid metabolism (5 DEGs with an enrichment score of 4.9), purine metabolism (8 DEGs with an enrichment score of 3.5), pyrimidine metabolism (6 DEGs with an enrichment score of 2.9), and biosynthesis of secondary metabolites (15 DEGs with an enrichment score of 1.3).

Another group of genes from the 1618 differentially down-regulated genes at unrestricted DO were the 44 genes up-regulated in the restricted DO. The top pathways associated with this group belonged to the TCA cycle (8 DEGs with an enrichment score of 19.8), carbon metabolism (9 DEGs with an enrichment score of 10.4), oxidative phosphorylation (4 DEGs with an enrichment score of 5.5), fatty acid biosynthesis (2 DEGs with an enrichment score of 3.9), ribosome formation (4 DEGs with an enrichment score of 3.3), and biosynthesis of secondary metabolites (9 DEGs with an enrichment score of 3.1). This group of 44 genes is an indication that translation and energy generating pathways were down-regulated in the unrestricted DO while the same cellular processes were up-regulated in the restricted DO.

From the 1286 DEGs at restricted DO, 547 were up-regulated and 154 of the 547 were exclusively up-regulated in restricted DO. These 154 DEGs were associated with energy generating pathways such as oxidative phosphorylation (6 DEGs with an enrichment score of 6.4), TCA cycle (3 DEGs with an enrichment score of 3.0), and carbon metabolism (7 DEGs with an enrichment score of 3). From the same 1286 DEGs, 224 that were exclusively down-regulated at the restricted DO were also overrepresented in processes related to ribosome formation (7 DEGs with an enrichment score of 5.4), biosynthesis of siderophore group non-ribosomal peptide (2 DEGs with an enrichment score of 4.2), glycerolipid metabolism (2 DEGs with an enrichment score of 3.2), and biosynthesis of secondary metabolites (13 DEGs with an enrichment score of 3.0).

### Pathway analysis in *E. coli* producing rARU at restricted and unrestricted DO

Pathway analysis of the genes at log and late-log phases of the cultures grown at restricted and unrestricted DO was identified by the microarray. It was conducted by determining the overall gene expression flux through pathways using the omics dashboard tool of EcoCyc^33^. The differential gene expression was estimated by applying a 1-way ANOVA on samples from log and late-log phases of the restricted and unrestricted DO. After filtering out the non-significant genes, the identified genes were grouped into major and sub-major pathways by EcoCyc.

**Figure 3:**
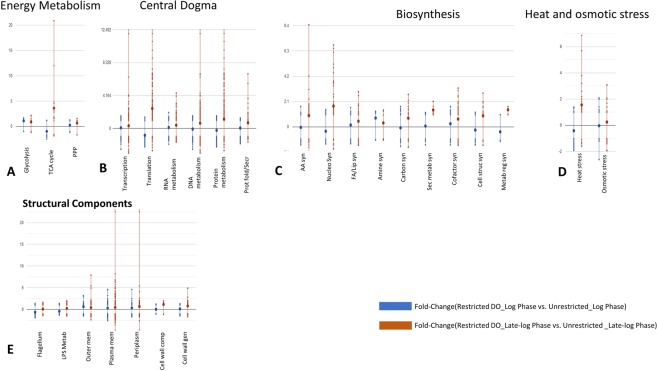
Overall gene expression distribution flux through major metabolic processes at restricted compared to unrestricted DO. (**A**) Energy metabolism, (**B**) Central dogma, (**C**) Biosynthesis, (**D**) Heat and osmotic stress, and (**E**) Structural components. The large dot represents the average (mean) of all data and small dot represents a data value for subsystem or an individual gene (generated with EcoCyc).

The change in the gene expression at log and late-log phases of cellular functions related to biosynthesis, energy metabolism, central dogma, heat and osmotic stress, and cell structural components at both aeration conditions are shown in Fig. 3. The small dot represents fold change expression values for subsystem or individual genes within the subsystem, while the large dot represents the average (mean) fold change expression values for all the subsystems in that pathway. When the gene expression distribution in the log phase was compared, most pathways behaved similarly at both restricted and unrestricted DO, which matched the correlation shown in the PCA among the principal components of the microarray gene expression profiles.

As seen in Fig. 3, the large dots were close to the baseline representing no-significant difference when gene expression of the log phase of restricted and unrestricted DO was compared. However, when the gene expression distribution in the late-log phase was compared, significant differences associated with energy metabolism pathways were observed. A 4-fold increase in expression of genes associated with the TCA cycle and a 2-fold increase in expression of genes associated with aerobic respiration in late-log restricted DO was observed as compared with the late-log phase at unrestricted DO. At the same time, no significant change in the gene expression of glycolysis, pentose phosphate pathway (PPP), and fermentation was observed when the restricted and unrestricted DO conditions were compared.

However, when processes such as translation, RNA metabolism, protein metabolism, and protein folding/secretion were evaluated, there was an increase in overall gene expression of ~3.5, ~1, ~2 and ~1.5-fold, respectively at restricted DO compared with unrestricted DO. Heat stress related genes showed a ~2-fold increase and cell structural component synthesis processes like cell wall, plasma membrane, periplasmic membrane, and outer membrane biosynthesis genes were differentially expressed.

### Proteomics analysis

Proteomics analysis was performed on the same samples used for the gene expression analysis. The PCA of the proteomics data showed correlation among the three replicates (Supplementary Fig. 1) and were correlated with the sample distribution that was observed from the gene expression data. The proteomics data were analyzed with a 1-way ANOVA and used a filtration cutoff of log_2_ fold change of ≥1.5≤ and p-value of ≤ 0.05. The 167 down-regulated and 45 up-regulated differentially expressed proteins were detected when the culture was transitioned from log to late-log (late-log vs log phase) at unrestricted DO. At restricted DO, 197 differentially expressed proteins were up-regulated and 34 were down-regulated. The gene expression data were used to identify affected pathways, and the proteomics data were used for confirmation at the protein level of identified key genes. The complete proteomics data is provided in Supplementary Table 3.

### Sequence feature analysis of rARU

By using ScanProsite^34^, 31 conserved cell wall binding motifs, each composed of 20 amino acids (QNRFLHLLGKIYYFGNNSKA), were detected in the expressed rARU. BLASTp searches based on these motifs against the Protein Data Bank (PDB) database retrieved several highly similar structures in different microorganisms (Supplementary Table 4). The main BLASTp hits were separated into two types: a) single proteins (e.g., choline binding, dextransucrase, autolysin, and endolysin), and b) clostridium Toxin A or Toxin B. Other hits were structures of toxin fragments bound to molecules such as single domain antibodies (e.g., VHH, PA50 Fab), DARPin, the surface protein PspA [*Streptococcus pneumoniae* R6] (NP_357715.1), and the cell wall protein [*Clostridioides difficile* 630] (YP_001089224.2).

VHH protein, as a known interactant of TcdA, was used to identify proteins in the *E. coli* that may have similar structures to VHH, and therefore can unexpectedly interact with the recombinant TcdA. Therefore, subsequent BLASTp searches were performed with single domain antibody sequence VHH (accession 4NC0_B) as a query against the UniProtKB/Swiss-Prot (Swiss-Prot) database using DELTA-BLAST algorithm and word size adjusted to 2. These searches retrieved a short fragment of *E. coli* UlaR protein (accessions P0A9W2.1 and B7UQK0.1) which is a transcription regulator responsible for the utilization of l-ascorbate under anaerobic conditions. By using PSI-BLAST with the A26.8 VHH protein sequence, the following proteins were identified: YahD (accession number: P77736) and ArpA (accession number: P23325) (“ankyrin repeat” proteins), the outer membrane usher protein HtrE (accession P33129.3), Formylglycinamide ribonucleotide amidotransferase (accession Q1R8H7.3), DNA-invertase PinE (accession P03014.2), and Wzc protein (a Tyrosine-protein kinase required for the extracellular polysaccharide colanic acid synthesis with relatively weak yet informative homologies).

### Expression of enhanced green fluorescent protein (eGFP) fused to partial rARU sequence at restricted and unrestricted DO

**Figure 4:**
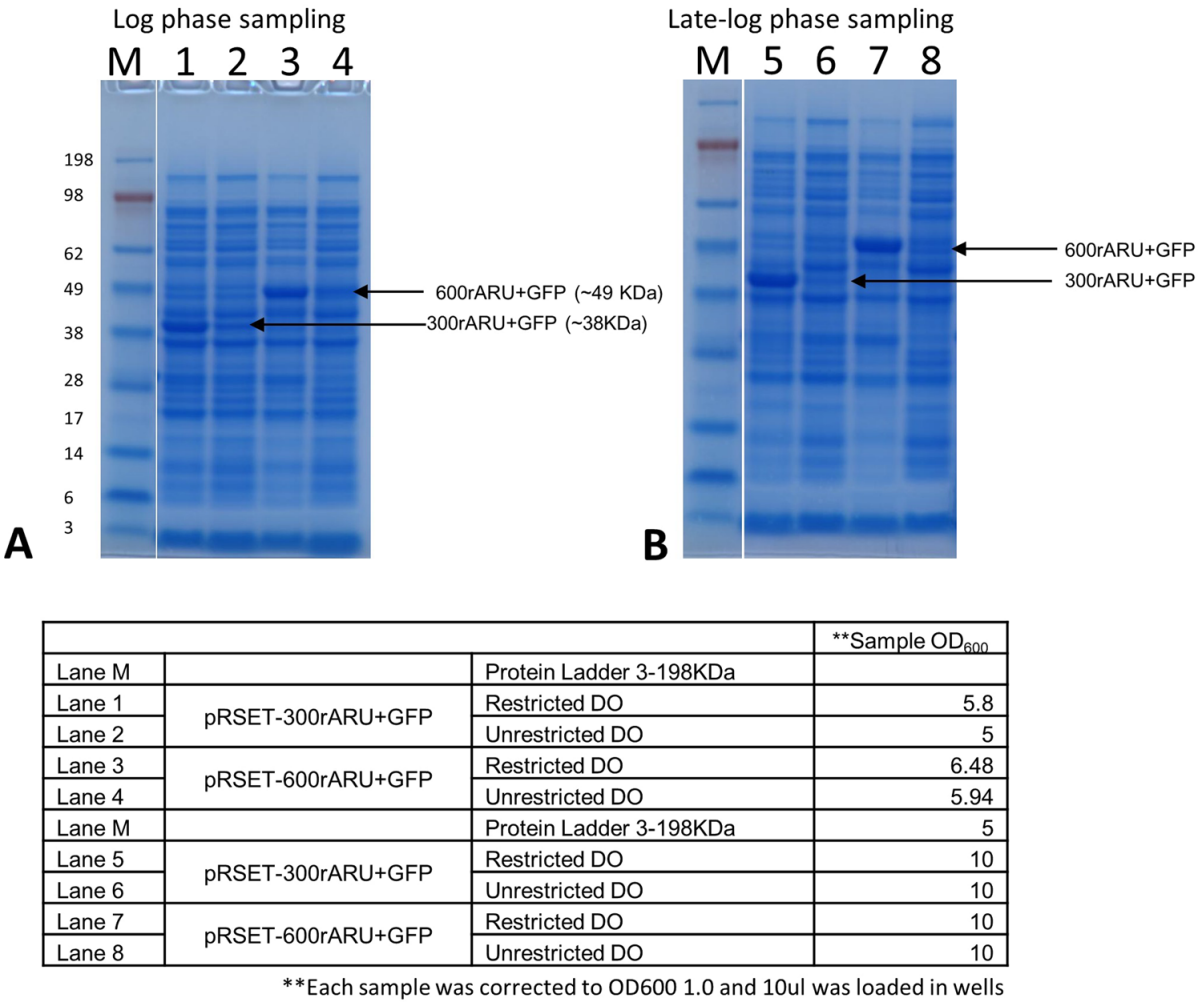
SDS-PAGE showing intracellular expression of eGFP and fusion proteins. (**A**) Log phase samples, 300rARU + GFP expression in lane 1 and 2 at restricted DO and unrestricted DO respectively; 600rARU + GFP expression in lane 3 and 4 at restricted and unrestricted DO respectively; (**B**) Late-log phase samples, 300rARU + GFP expression in lane 5 and 6 at restricted DO and unrestricted DO respectively; 600rARU + GFP expression in lane 7 and 8 at restricted and unrestricted DO respectively. The OD of the sample at harvest are mentioned in the table shown in this figure (Sample OD_600_ during harvest). The marker lane and protein band lanes were cropped from the same gel for clarity of results. The uncropped image of each gel provided in Supplementary Fig. 2.

To assess if the reason for the difference in rARU expression in restricted and unrestricted DO is associated with special properties of the rARU gene, the initial 300 and 600 bp sequence of the rARU gene were fused with the eGFP gene. The resulting constructs were cloned in the pRSETb backbone creating pRSETb-300rARU + eGFP and pRSETb-600rARU + eGFP. The 300 and 600 bp of the rARU contained 3 and 7 cell wall binding motifs respectively. The strain was transformed with the two plasmids and eGFP expression was evaluated at restricted and unrestricted DO. The results in Fig. 4A,B showed that the expression of both fusion constructs was affected by the DO conditions. Expression of the 300rARU + eGFP and the 600rARU + eGFP fusion proteins were 1.8- and 1.99-fold higher in the log phase in cells grown at restricted DO as compared with the log phase of cells grown at unrestricted DO. Correspondingly, late-log phase showed 2.8- and 2.7-fold higher expression at restricted DO as compared with the unrestricted DO condition. The fold change protein expression was calculated using IQTL (ImageQuant TL 8.2) image analysis software.

## Discussion

Recombinant rARU from *C. difficile* was over expressed in *E. coli* only when the bacteria were grown at restricted DO concentrations. Therefore, an optimized expression was established by supplying the growing culture with 2 vvm air that generated restricted DO conditions and supported ~27-fold higher rARU concentrations. The possibility that the restricted DO conditions affected the T7 promoter was ruled out since at these conditions the expression of the T7 RNA polymerase was not affected (Supplementary Figure 3).

### Transcriptomic and proteomic response to restricted and unrestricted DO

A larger number of DEGs were down-regulated at unrestricted DO as compared with restricted DO. A total of 1618 DEGs were down-regulated at unrestricted DO and of these, 1066 were down-regulated exclusively at unrestricted DO. Particularly important is the down-regulation of the RNA polymerases (*rpoB -5.67-fold, rpoC -4.46-fold, rpoZ -3.39-fold* and *rpoA -6.58*) which are essential for transcription initiation. In contrast, except for *rpoD* which was down-regulated, *rpoA*, *rpoC*, and *rpoZ* were up-regulated at restricted DO (1.2-fold, 1.0-fold, and 1.31-fold respectively). Since the translation process depends on the availability of ribosomes, amino acids, aminoacyl tRNA, and ATP molecules, it is not surprising that the genes associated with these processes were also differentially down-regulated in the unrestricted DO. This group includes 52 DEGs related to ribosome formation, 19 related to aminoacyl tRNA biosynthesis, 44 associated amino acid biosynthesis, and 17 involved in oxidative phosphorylation. The down-regulation of genes associated with energy related metabolism, central metabolism, and ribosomal RNA were previously shown to be associated with the cellular stress response (CSR) generated during recombinant protein overexpression^35,36^. The down-regulation of the above-mentioned processes displays a behavior that supports the lower amount of rARU expressed at unrestricted DO. Previous reports showed that the presence of the rarely used codons of arginine and leucine in clostridial proteins is a possible bottleneck in their translations^18,21,22^. The aminoacyl tRNA genes for arginine (*argS*) and leucine (*leuS)* in late-log phase were up-regulated (f.c.1.9, and 1.67 respectively) at restricted DO condition as compared with unrestricted DO condition.

In addition to the down-regulation of the DEGs mentioned above, genes associated with protein folding were also down-regulated at unrestricted DO. This included the 24 heat shock response genes, an indication that, rARU expression was too low to induce protein folding stress. Zhang *et al*. reported^37^ that during recombinant protein expression, there is up-regulation of heat shock genes (chaperones and foldases) which are required for proper folding^37^; however, a high expression of heat shock response genes was observed only when the cells grew at restricted DO thereby indicating folding stress associated with higher rARU expression.

In both unrestricted and restricted DO, 508 DEGs were down-regulated and were associated with glycerophospholipid, purine, and pyrimidine metabolism, and with the biosynthesis of secondary metabolites. The *plsY, glpC, glpQ, glpB*, and *glpD* DEGs from the glycerophospholipid metabolism were down-regulated in unrestricted DO (−1.84-fold, −4.96-fold, −2.47-fold, −1.62-fold, and −4.99-fold respectively) and in restricted DO (−1.63-fold, −8.69-fold, −4.97-fold, −2.38-fold, and −7.03-fold respectively). Glycerophospholipids are essential components of the bacterial cell wall and fatty acid^38^ as well as for glycerol consumption through the glycerol-3-phosphate regulon. The finding that the repressor of the glycerol-3-phosphate regulon (glpR) was up-regulated higher in unrestricted DO (1.28-fold) as compared with restricted DO (1.03-fold) can explain the higher inhibition of glycerol utilization at unrestricted DO.

Culture grown at restricted DO showed a smooth transition from log to late-log possibly because 2.5 times less genes were differentially expressed as compared with unrestricted DO growth. Only 224 genes involved in ribosome formation, siderophore, and secondary metabolite biosynthesis were down-regulated when the cells grew in restricted DO. The number of down-regulated ribosome formation related genes were 7.4 times less in restricted DO compared with unrestricted DO, suggesting a possible reason for the increased translation at restricted DO. At the same time, 154 DEGs were up-regulated only at restricted DO (Fig. 2) and these genes were associated with oxidative phosphorylation, TCA cycle, and carbon metabolism. The oxidative phosphorylation genes *sdhA* (2.63-fold), *cyoD* (3.15-fold), *sdhC* (3.66-fold), *cyoA* (2.03-fold), and *sdhB* (1.67-fold) were up-regulated at restricted DO whereas *sdhA* (−1.18-fold), *cyoD* (−1.06-fold), *sdhC* (−1.27-fold), *cyoA* (−1.31-fold), and *sdhB* (−1.10-fold) were down-regulated at unrestricted DO. As indicated by all the above the transcription, translation, and energy generating pathways were much more active at restricted DO in comparison with the unrestricted DO.

### Changes in the global regulators

The ArcA/ArcB two-component system involved in sensing oxygen availability, acts by triggering oxidative response, regulates central carbon metabolism, and suppresses the TCA cycle under anaerobic conditions^39,40^. No significant differences in the expression level of *arcA* and *arcB* at the two growth conditions were observed and is an indication that there is no difference in the regulation of the central carbon metabolism and the TCA cycle at restricted and unrestricted DO.

The global regulator *lrp* is a leucine-responsive transcriptional regulator which acts by down-regulating nutrient uptake in rich medium^41,42^. *lrp* was up-regulated in restricted DO (1.6-fold), thus reducing nutrient uptake and lowering growth. However, at unrestricted DO, *lrp* was down-regulated (−2.17-fold) which was associated with higher glycerol consumption and a higher growth rate. The increase in glycerol consumption was also supported by the proteomic data which showed up-regulation of glycerol kinase GlpK (2.17-fold) at unrestricted DO as compared to a 1.07-fold up-regulation at restricted DO condition.

The global regulator csrA affects ppGpp expression through relA, which changes σ^s^ behavior to affect the transcription of stress survival genes and DNA replication^43^. *csrA* was found to be up-regulated at unrestricted DO (2.07-fold higher) than at restricted DO (1.26-fold). Although no changes were observed concerning the *relA* expression, there is a possibility that differential expression of *csrA* can affect various cellular processes by altering the translation and stability of target molecules such as tRNA and rRNA^44^. The higher expression of *csrA* could be the reason for down-regulating translation (ribosomal, tRNA, and amino acids) and energy-related genes at the unrestricted DO condition.

The global DNA-binding transcriptional dual regulator *fis*, was up-regulated at restricted DO (1.92-fold) and down-regulated at unrestricted DO (−4.27-fold). *fis* acts by controlling gyrase and changing the expression of topoisomerase I^45^ and alleviates stress response, energy, and carbon metabolism processes. *fis* also up-regulates processes involved in flagellar biosynthesis and translation (rRNA and tRNA genes)^46^. Therefore, it is possible that *fis* down-regulation is the reason for down-regulating 54 genes associated with translation-related processes at unrestricted DO. An additional transcriptional regulator that may have a possible role in different DO conditions is the RNA chaperone *hfq*. This molecule binds to small RNA and is involved in elongating poly(A) tails and in stabilizing mRNA^47,48^. However, there is a possibility that *hfq* can induce mRNA decay by sRNA mediated regulation, which has been shown to be related to different growth conditions^49^. *hfq* was up-regulated (1.34-fold) at restricted DO thereby potentially enhancing rARU mRNA level, and down-regulated (−1.33-fold) at unrestricted DO possibly lowering rARU mRNA levels

### Cell wall binding motifs in rARU

A ProSite scan showed that the expressed rARU gene (Supplementary Table 5(S5a)) contained 31 cell wall binding repeats, each composed of 20 amino acids (QNRFLHLLGKIYYFGNNSKA)^50^ belonging to the class PS51170 which has a unique structure. So far, only 15 protein sequences were identified to contain a similar cell wall binding repeat profile (Supplementary Table 5(S5b)). The Blast search showed proteins with similar structures in *E. coli* that potentially can bind to rARU (Supplementary Table 4). These were:  the transcriptional repressor ulaR, the tyrosine protein kinase Wzc, the outer membrane Usher protein HtrE, ankyrin proteins (YahD and ArpA), the phosphoribosylformylglycinamide synthetase PurL, and the site-specific DNA recombinase PinE. Additional interactions with cellular proteins are possible due to the presence of the two cysteine residues in rARU, which can form disulfide bridges at high dissolved oxygen levels.

**Figure 5:**
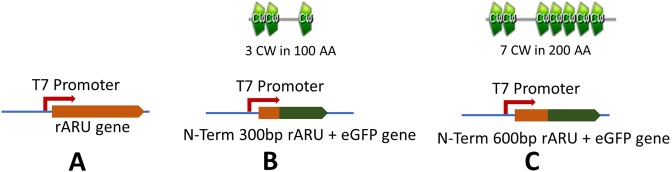
Gene cloning and fusion constructs. (**A**) pRSETb-rARU (original construct); (**B**) 300 bp of N-terminal rARU fused with eGFP gene and cloned in place of rARU in original clone (pRSETb-[N-Term 300 bp rARU + eGFP gene]); (**C**) 600 bp of N-terminal rARU fused with eGFP gene and cloned in place of rARU in original clone (pRSETb-[N-Term 600 bp rARU + eGFP gene]).

The possibility that higher expression at restricted DO is likely due to the interaction between cell wall binding motifs and cellular proteins and was further tested by the fusion of a partial rARU sequence containing cell wall binding motifs with eGFP. Initially to capture the sequence feature, we fused 600 bp of rARU (with 7 cell wall binding motifs) N-terminal region to eGFP to see if this affects the protein expression. As a result, the rARU expression was higher at restricted DO. Then we tried smaller sequence size of 300 bp (with only 3 cell wall binding motifs), which gave similar results. The expression of eGFP fused to both 300 and 600 bp of rARU at the N-terminal region was higher at restricted DO. The selected 300 and 600 bp from the rARU N-terminal contained respectively 3 and 7 cell wall binding repeats (Fig. 5), which was likely responsible for the higher expression at restricted DO. Although the mechanism behind this interaction is unknown, it is possible that the interaction of the rARU sequence with cellular proteins triggered a higher expression at restricted DO condition.

The interaction of rARU with the transcriptional repressor ulaR may influence the biosynthesis of NADH and pentoses by affecting the L-ascorbate uptake^51^. ulaR operates by interaction with L-ascorbate-6-P and by binding to the intergenic region of ulaA and ulaG and inhibition of the *ulaABCDEF* operon transcription. UlaR was down-regulated in the late-log phase (ulaR f.c. −1.03) at restricted DO, which may cause the up regulation of ulaG. As a result, ulaB, ulaD, ulaE, and ulaF of the ulaABCDEF operon remained active in the late log phase of restricted DO enabling the cells to efficiently utilize L-ascorbate and support PPP to produce NADH and pentoses.

**Figure 6:**
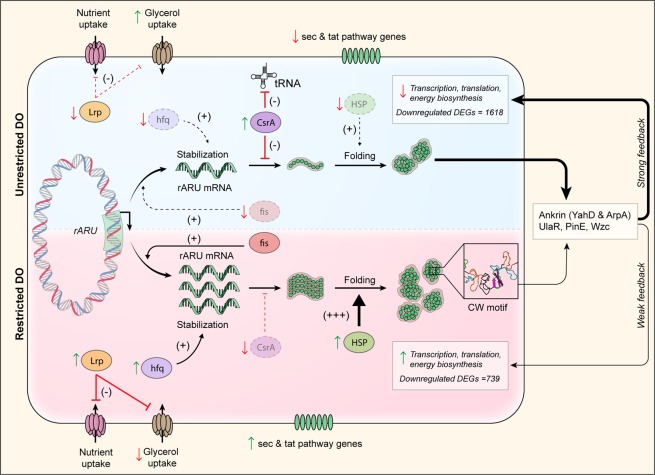
Schematic showing major processes and regulators differentially affected during rARU expression at restricted and unrestricted DO.

The interaction of rARU with the “ankyrin like” proteins of *E. coli* such as YahD (*yahD* f.c. 1.25 at log phase and f.c. 1.09 at late-log phase) and ArpA (*arpA* f.c. −1.05 at log phase and f.c. 1.54 at late-log phase) could also interfere with the integrity of the cell membrane and affect cell growth. The “ankyrin repeat” containing proteins were discovered in mammalian cells and were involved in cell signaling, regulation, and structural integrity. A previous study on the overexpression of an “ankyrin repeat” containing protein (e.g., RNase L) caused RNA degradation and inhibition of cell growth in *E. coli*^28^. Interestingly, the role of “ankyrin repeat” proteins at signaling and regulatory levels was never explored in *E. coli*. A list of many other *E. coli* proteins which may possibly interact with rARU is provided in Supplementary Table 4. The list includes the Wzc protein (*wzc* f.c. −1.12 at log phase and f.c. −1.25 at late-log phase) which is involved in making exopolysaccharide colanic acid (M-antigen) which increases *E. coli*’s ability to survive in environmental stresses^52^. Recently, this ability was shown to be regulated through the master regulator *csrA*^53^, which was up-regulated at unrestricted DO in this study. A schematic representing the state of the major metabolic processes and regulators in *E. coli* at restricted and unrestricted DO during rARU expression are presented in Fig. 6.

## Conclusion

Recombinant rARU, a partial fragment of Toxin A from the anaerobic bacterium *C. difficile*, was expressed efficiently from *E. coli* only at low DO conditions. We observed that rARU transcript accumulation was highest only when the culture was grown at a restricted DO concentration. It is very likely that this unusual expression condition is an indication that a special gene sequence and structure of the produced protein are related to the expression. Therefore, significant production was possible only by applying restricted DO growth strategy. A possible reason for this special phenomenon was the presence of cell wall binding repeats motifs that allowed transcription and translation at restricted DO and therefore increased its expression at these conditions. The role of the heterologous protein sequence in the metabolic toxicity due to overexpression of recombinant proteins was reported, but no analysis was performed to understand the functional features associated with the sequence and how it interacted with the host cellular machinery^54–56^. This report demonstrated a connection between rARU expression and restricted DO, which presents the possibility of the improved expression of other recombinant proteins of anaerobic origin or of proteins that are associated with cell wall binding repeat profiles. The implemented restricted DO expression strategy used in this work will likely improve the expression of other similar proteins in *E. coli*.

## Methods

### Bacterial strain and expression vector

*E. coli* Tuner (DE3) pLacI/TZ56 [F- ompT hsdSB (rB- mB-) gal dcm lacY1 (DE3) pLacI (CmR)] (Novagen, Madison, WI) was used for the growth and production experiments. Expression plasmid pRSET-B-rARU-KanR containing rARU gene under T7 promoter was transformed in the *E. coli* strain. The pRSET-B-rARU plasmid possesses kanamycin resistance along with entire C-terminal repeating region of toxin A (rARU) (861 amino acids plus 4 amino acids upstream) having a Molecular weight (MW) of 104 kDa^16^, along with a 6x His tag at the C-terminal. Both plasmid and strain were provided by TechLab, Inc. (Blacksburg, VA). Two fusion constructs were designed consisting of partial N-terminal regions (i.e., 300 bp and 600 bp of rARU with the eGFP). The fusion constructs were renamed as pRSET-B-300rARU + eGFP and pRSET-B-600rARU + eGFP.

### Media and culture conditions

Modified Terrific Broth (TB) media was used throughout the growth experiments: 24 g/L yeast extract, 12 g/L Tryptone, 12.4 g/L K_2_HPO_4_, 2.35 g/L KH_2_PO_4_, 0.5 g/L MgSO_4_·7H_2_O, 1 ml/L of trace element solution (27 g/L FeCl_3_·6H_2_O, 2 g/L ZnCl_2_·4 H_2_O, 2 g/L CoCl_2_·6H_2_O, 2 g/L NaMoO_4_·2H_2_O, 1 g/L CaCl_2_·2H_2_O, 1 g/L CuCl_2_, 0.5 g/L H_3_BO_3_, 100 ml/L HCl concentrated^57^, and 25 mg/L kanamycin, supplemented with 15 g/L glycerol as indicated.

### Batch fermentation

Starter culture was grown overnight at 37 °C in 200 ml modified TB medium and inoculated into 4.0 L medium in a B. Braun bioreactor. The cultures were grown at unrestricted DO and restricted DO concentrations without addition of Isopropyl β- d-1-thiogalactopyranoside (IPTG). The unrestricted DO (30%) condition was maintained with a constant supply of 2 vvm air cascaded to agitation, and the restricted DO conditions were supplied with 2 vvm air and a fixed agitation of 650 rpm. pH was controlled at 7.0 by addition of 2 M NaOH and 10% H_2_CO_3_.

Samples for rARU, acetic acid, and total RNA were collected at regular intervals. After centrifugation at 14,000 g for 10 min at 4 °C, the supernatant was kept at −20 °C for glycerol and acetate analysis, and the cell pellets were quickly frozen by dry ice and stored at −80 °C for RNA extraction. Biomass was monitored by measuring the OD_600_. Glycerol and acetate concentration were determined using the Cedex Bio HT Analyzer (Roche, Penzberg, Germany).

### rARU quantification

Culture samples were diluted with 1X PBS to bring the cell concentration to 1.0 OD_600_ and the diluted culture was centrifuged at 16,000 g for 5 min, the supernatant was removed, and the pellet was frozen at −20 °C. Each cell pellet was thawed and resuspended in 200 μl of lysis buffer (BugBuster Protein Extraction Reagent, Novagen, Madison, WI). Processed samples were centrifuged at 16,000 g for 20 minutes at 4 °C, and then supernatant was collected. The ELISA based method using monoclonal antibody conjugated to horseradish peroxidase of *C. difficile* Toxin A and B II enzyme immunoassay kit (TechLab, Inc., Blacksburg, VA) was used for quantitation of rARU in the processed supernatant (Fig. 1B). Purified rARU (List Biological Laboratories, Inc. Campbell, CA) was used as the standard, and assay plates were read on UV/VIS microplate spectrophotometer (SpectraMax190 Molecular Devices, Sunnyvale, CA) at 450 nm and 620 nm.

### Total RNA purification and northern blot analysis

Total RNA was isolated with MasterPure RNA Purification Kit (Epicentre Technologies, Madison, WI) which resulted in RNA with a A260/A280 ratio of 1.85–1.95. To ensure equivalency between individual samples, the 23S and 16S rRNA from each sample were analyzed by Agilent 2100 Bioanalyzer (Agilent Technologies, Santa Clara, CA) and the rRNA ratio (23S/16S) for all samples was calculated to be greater than 1.5. The isolated RNA (5 ng/well) was separated using 1% agarose/formaldehyde denaturing gel at 75 V. The gels were blotted on Nytran Super Charge membrane (11 cm × 14 cm) (Schleicher & Schuell, Keene, NH) at room temperature (25 °C) in 20 X SSC, and the membranes were fixed by UV-induced cross-linking. Amplification of DNA fragments of rARU gene were performed using the following primers: Forward primer: 5′-TGCACCTGCTAATACGGATG-3′; Reverse primer: 5′-GCCATCCAGTAACTGCAACA-3′.

PCR products were purified and labeled with ^32^P using Ready-To-Go DNA Labelling Beads (Amersham Pharmacia Biotech, Piscataway, NJ) and were purified by Probe Quant G-50 Micro column (Amersham Pharmacia Biotech, Piscataway, NJ). Hybridization with ^32^P-labeled DNA probes was performed with Quickhyb solution (Stratagene, La Jolla, CA) as recommended by the manufacturer. Northern blots were repeated in triplicate to verify reproducibility and quantification was performed by phosphor image scanning.

### rARU and T7 RNA polymerase mRNA quantitation with qPCR

Total RNA was extracted from cultures grown at restricted and unrestricted DO conditions. The extracted RNA was converted to cDNA using maxima first strand cDNA synthesis kit (Thermo Scientific, Waltham, MA). The cDNA was used as the template for qPCR amplification of the rARU gene transcript and used the following thermocycler steps: 1 cycle: 95 °C for 10 min; 40 cycles: 95 °C for 15 sec, 60 °C for 1 min. The primers used were: Forward primer: 5′-TGCACCTGCTAATACGGATG-3′; and Reverse primer: 5′-GCCATCCAGTAACTGCAACA-3′. The primers used for the T7 RNA polymerase were: Forward primer: 5′-ACAGCCTTCCAGTTCCTGCAAGAAATCAAG-3′; and Reverse primer: 5′-CATTTTGGCGGTGTAAGCTAACCATTCCGG-3′. The thermocycler steps used for the qPCR amplification of T7 RNA polymerase transcript were: 1 cycle: 95 °C for 3 min; 39 cycles: 95 °C for 10 sec, 60 °C for 40 sec.

### Transcriptomic profiling with microarray

#### RNA isolation

culture samples were treated with RNA protect, the suspension was centrifuged at 5000 rpm for 10 min and the pellet was resuspended in TRIzol reagent (Life Technologies, Carlsbad, CA) and immediately frozen in dry ice and stored at −80 °C. RNA was extracted by suspension of the cell pellets in 0.5% SDS, 20 mM NaAc, and 10 mM EDTA and extracted twice with hot acid phenol: chloroform, followed by 2 additional extractions in phenol: chloroform isoamyl alcohol. Absolute ethanol was added, the extract stored at −80 °C for 15 min, and then the stored RNA extract was centrifuged at 14,000 g for 15 min and washed with 70% ethanol and dried. The dried pellet was resuspended in ultrapure water (KD Medical, Columbia, MD) and was evaluated in a Bioanalyzer (Agilent, Santa Clara, CA) using RNA integrity number > 8.0 as a metric of sample quality. To avoid batch effect, all samples were extracted at the same time.

#### DNase treatment

The extracted RNA samples were further treated with the TURBO™ DNase (Invitrogen/Novex, Carlsbad, CA) to remove trace amount of DNA. The absence of DNA in the samples was confirmed with qPCR of housekeeping genes, and no amplification was observed up to 25 cycles.

#### Hybridization and scanning

Microarrays were performed with samples of RNA from log (3 hours) and late-log (5 hours) growth phases. The DNase treated RNA samples were processed, hybridized on microarray chip, and scanned at the genomics core facility (University of Maryland School of Medicine, Center for Innovative Biomedical Resources, Baltimore, MD). Raw data obtained from probe intensities were processed using Partek Genomics Suite 7.0 software (Partek, St. Louis, MO).

### Proteomics with LC/MS/MS

Peptides were obtained from each sample with reduction and alkylation of cysteines using a detergent approach^58^. One-third of peptides obtained from each sample were pooled together. The pool and the remaining sample were loaded (separately) on C8/C18 STAGE tips and subjected to on-column reductive demethylation^59^ with each sample receiving one label from the pool and one from the remaining sample. After elution, the same portion of the pool elute was added to each sample eluate, and these thoroughly “doped” samples were dried under nitrogen and analyzed as described previously^60^.

### Statistical analysis

Time point samples were processed using biological triplicates from three different batch runs on different dates. ANOVA was performed to compare transcriptomic profiles among log and late-log phase samples at unrestricted DO and restricted DO. Significant DEGs were obtained by using filtering criteria of p-value and fold change cut-off of less than or equal to 0.05 and 1.5 respectively.

### Microarray and proteomics data analysis

Raw microarray data were processed and normalized using Partek Genomics Suite7.0 and annotations for *E. coli* BL21 were used to identify the gene ID. The normalized data files were analyzed with PCA to determine the significant differences in the spatial distribution among the populations of biological replicates from the different samples collected from cultures growing at unrestricted (30%) and restricted DO conditions.

Twelve samples were processed: three biological replicates from two time points (log and late-log phase) from each growth condition for both microarray and proteomics studies. Fold change values were calculated among two different contrasts: 1a) log phase (restricted DO) vs log phase (unrestricted DO), and 1b) late-log phase (restricted DO) vs late-log phase (unrestricted DO); and 2a) restricted DO condition (late-log phase vs log phase), and 2b) unrestricted DO (late-log phase vs log phase). Each comparison was performed using ANOVA to generate fold change values among the two groups (ANOVA estimates the geometric means of the samples in each group to calculate the fold change for the contrast conditions chosen).

The significant DEGs were identified by applying a filtering criterion of >1.5 and <−1.5-fold change with p-value < 0.05. Gene ontology was performed using Fisher’s exact test with the ‘gene set analysis’ tool of Partek Genomics Suite 7.0 by integrating the KEGG database for the *E. coli BL21* strain (organism code ‘ebl’). The significant DEGs gene list was used to find the overall gene expression flow of subsystems in the enriched pathways, to predict the relative up or down-regulation of pathways using the omics dashboard tool of EcoCyc^33^.

MaxQuant (version 1.3.0.5, Max-Planck-Institute of Biochemistry, Munich, Germany)^61^ was used with a binary setting and requiring either light or heavy di-methylation of N-termini and lysine for protein raw data analysis. The data was searched using protein sequences from *E. coli* as well as known contaminants and a list of control proteins used to monitor reaction efficiency. Default values were used but matches between runs was enabled with a 0.5 min allowable difference in elution time (which was applied after spline fitting the elution patterns).

### Structural analysis

ScanProsite was used to detect the motifs in the expressed gene^34^. Sequence searching was done with Protein BLAST (BLASTp, version 2.8.1) using the web interface at the National Center for Biotechnology Information (NCBI), National Library of Medicine. BLASTp search parameters were used at the default setting except that the expect threshold was set to 1 and word size set to 3, and the searches were run against the Protein Data Bank (PDB) database.

## Supplementary information

Supplementary file.

## Data Availability

The datasets supporting the conclusions of this article are available in the [NCBI Gene Expression Omnibus (GEO)] database [accession number: GSE131635].
