# Supplementary material for: Effect of restricted dissolved oxygen on expression of Clostridium difficile toxin A subunit from E. coli
Source: Sci Rep. 2020 Feb 20;10:3059. doi: 10.1038/s41598-020-59978-1 (PMC7033237; doi:10.1038/s41598-020-59978-1)

Supplementary Information:

Article in *Scientific Reports*

Effect of restricted dissolved oxygen on expression of *Clostridium difficile* toxin A subunit from *E. coli.*

Ashish K. Sharma [1], Jenie Phue [1,2,] Emir Khatipov[3,6], Nimish Dalal [1,4], Eric D. Anderson[5], Joseph Shiloach[1*]

[1]Biotechnology Core Laboratory, National Institute of Diabetes and Digestive and Kidney Diseases, National Institutes of Health, Bethesda, MD, USA
[2] (current address) Center for Biologics Evaluation and Research, U.S. Food and Drug Administration, Silver Spring, MD, USA
[3]National Institutes of Health Library, Division of Library Services, Office of Research Services, National Institutes of Health, Bethesda, MD, 20892, USA
[4]Biologics manufacturing Science & Technology Bristol-Myers Squibb East Syracuse NY 13057
[5]Mass Spectrometry Facility, National Institute of Diabetes and Digestive and Kidney Diseases, National Institutes of Health, Bethesda, MD, USA
[6] (current address) National Institute of Dental and Craniofacial Research, National Institutes of Health Bethesda MD 20817

Email addresses of authors:
ashish.sharma@nih.gov
Jenie.Phue@fda.hhs.gov
emir.khatipov@nih.gov
DavidA@intra.niddk.nih.gov
nimish.dalal@bms.com
JosephS@niddk.nih.gov

Corresponding Author:
Joseph Shiloach, PhD
National Institute of Diabetes and Digestive and Kidney Diseases,
National Institutes of Health,
Building 14A, Room 173
14 Service Rd West
Bethesda, MD 20814
JosephS@niddk.nih.gov

Supplementary Figure 1. Principle component analysis of the proteomics data.

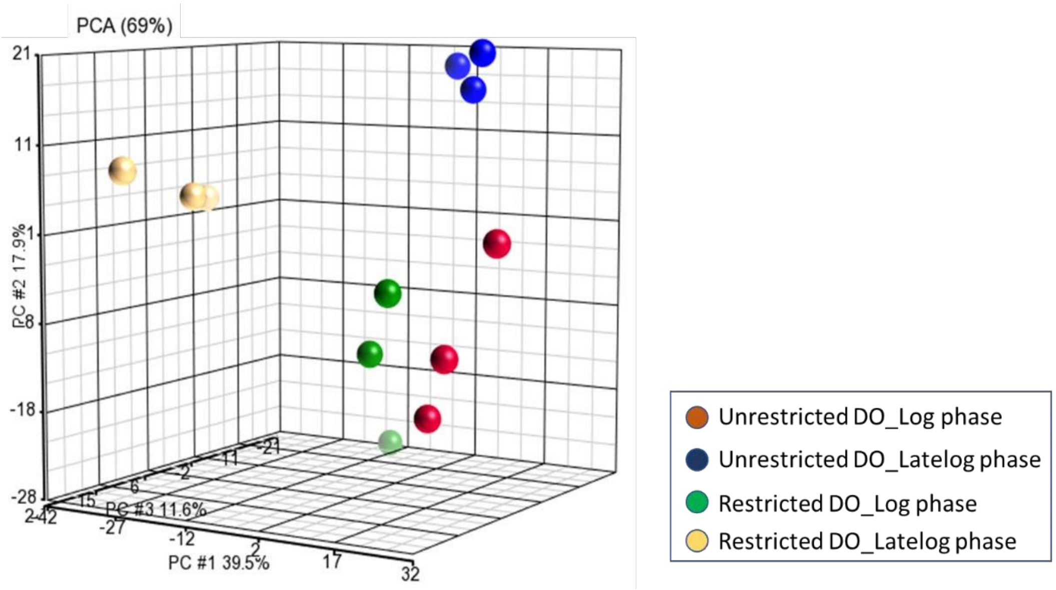

Supplementary Figure 2. Complete (Uncropped) picture of the SDS-PAGE gels shown in Figure 4.

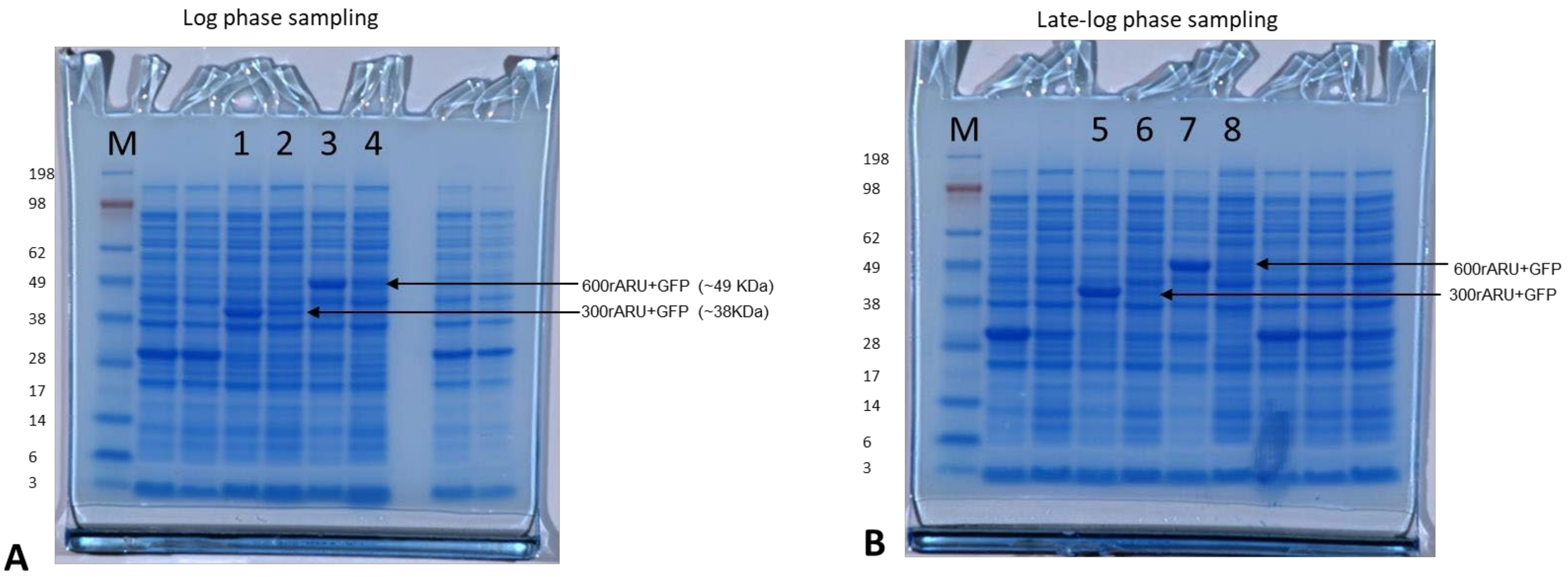

Gel				**Sample OD_{600}
Gel A	Lane M		Protein Ladder 3-198KDa	
	Lane 1	pRSET-300rARU+GFP	Restricted DO	5.8
	Lane 2		Unrestricted DO	5
	Lane 3	pRSET-600rARU+GFP	Restricted DO	6.48
	Lane 4		Unrestricted DO	5.94
Gel B	Lane M		Protein Ladder 3-198KDa	5
	Lane 5	pRSET-300rARU+GFP	Restricted DO	10
	Lane 6		Unrestricted DO	10
	Lane 7	pRSET-600rARU+GFP	Restricted DO	10
	Lane 8		Unrestricted DO	10

**Each sample was corrected to OD600 1.0 and 10ul was loaded in wells

Supplementary Figure 3. qPCR quantitation of T7 RNA polymerase in 3hr and 5hr time point samples of cultures grown at restricted and unrestricted DO.

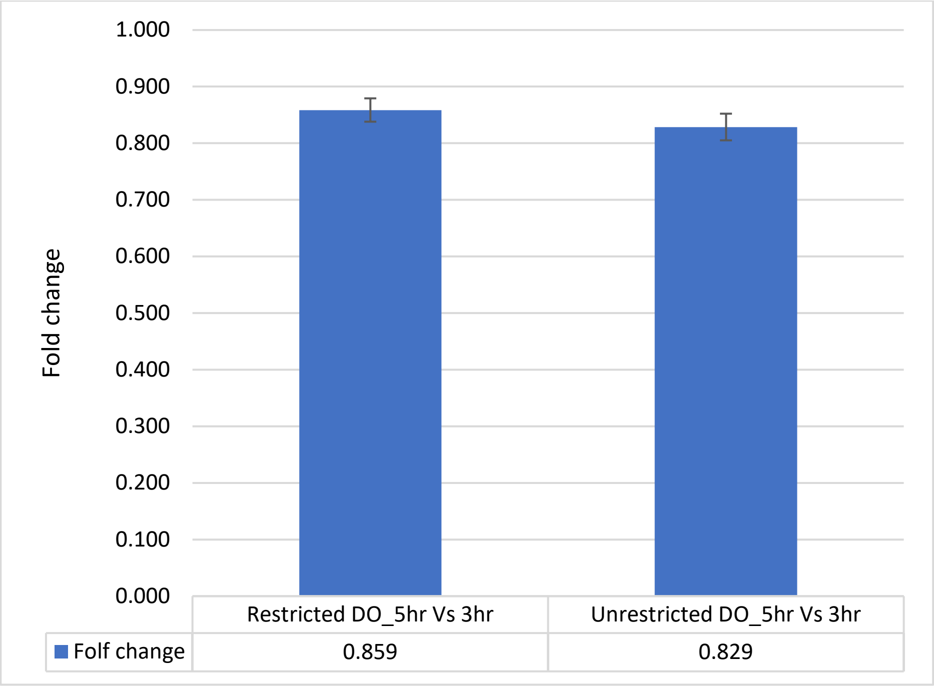

Supplementary Table 1. Gene ontology analysis on the different subsections of Venn categorization shown in Figure 3.

Table S1a. Gene ontology of 1208 DEGs.

function	*Enrichment Score*	*Enrichment p-value*	*% genes in group that are present*	*# genes in list, in group*	*# genes not in list, in group*	*GO ID*
transposition, DNA-mediated	15.94	1.19E-07	34.78	24	45	6313
carbohydrate:proton symporter activity	9.32	8.97E-05	58.33	7	5	5351
sulfur incorporation into metallosulfur cluster	8.85	0.000143806	100.00	4	0	31162
trehalose metabolism in response to cold stress	8.85	0.000143806	100.00	4	0	70415
DNA packaging	8.85	0.000143806	100.00	4	0	6323
cellular response to reactive oxygen species	8.85	0.000143806	100.00	4	0	34614
carbohydrate catabolic process	8.67	0.000170914	16.79	68	337	16052
galactose transmembrane transport	8.65	0.000175999	53.85	7	6	15757
acyl-CoA metabolic process	6.63	0.00131562	100.00	3	0	6637
galactose:proton symporter activity	6.63	0.00131562	100.00	3	0	15517
fucose metabolic process	6.51	0.00148606	46.15	6	7	6004
iron-sulfur cluster assembly	6.43	0.00160984	31.25	10	22	16226
cell wall macromolecule metabolic process	6.32	0.00179741	66.67	4	2	44036
carbohydrate metabolic process	5.94	0.00263805	14.63	85	496	5975

cyclic-guanylate-specific phosphodiesterase activity	5.63	0.00357478	40.00	6	9	71111
carbohydrate catabolic process	5.56	0.00383053	57.14	4	3	16052
divalent metal ion transport	4.96	0.00699988	50.00	4	4	70838
divalent inorganic cation transport	4.96	0.00699988	50.00	4	4	72511
lipoprotein metabolic process	4.96	0.00699988	50.00	4	4	42157
lipoprotein biosynthetic process	4.96	0.00699988	50.00	4	4	42158
response to osmotic stress	4.67	0.00938476	23.91	11	35	6970
transcriptional attenuation	4.50	0.0110933	60.00	3	2	31555
methylation	4.46	0.0115169	44.44	4	5	32259
macromolecule methylation	4.46	0.0115169	44.44	4	5	43414
DNA methylation or demethylation	4.46	0.0115169	44.44	4	5	44728
DNA modification	4.46	0.0115169	44.44	4	5	6304
DNA alkylation	4.46	0.0115169	44.44	4	5	6305
DNA methylation	4.46	0.0115169	44.44	4	5	6306
negative regulation of single-species biofilm formation	4.46	0.0115169	44.44	4	5	1900191
negative regulation of multi-organism process	4.46	0.0115169	44.44	4	5	43901
nitrite transport	4.46	0.0115169	44.44	4	5	15707
phosphotransferase activity, alcohol group as acceptor	4.39	0.0123966	22.22	12	42	16773
organophosphate catabolic process	4.33	0.0131471	26.67	8	22	46434
fatty acid oxidation	4.13	0.01616	24.32	9	28	19395
lipid modification	4.13	0.01616	24.32	9	28	30258
lipid oxidation	4.13	0.01616	24.32	9	28	34440
cellular lipid metabolic process	4.07	0.0171359	18.56	18	79	44255
lipid metabolic process	4.07	0.0171359	18.56	18	79	6629

regulation of single-species biofilm formation	4.06	0.0171764	30.00	6	14	1900190
regulation of multi-organism process	4.06	0.0171764	30.00	6	14	43900
regulation of cell motility	4.06	0.0171764	30.00	6	14	2000145
regulation of locomotion	4.06	0.0171764	30.00	6	14	40012
regulation of cellular component movement	4.06	0.0171764	30.00	6	14	51270
regulation of single-species biofilm formation on inanimate substrate	4.04	0.0175522	40.00	4	6	1900231
carbohydrate derivative catabolic process	3.99	0.018423	33.33	5	10	1901136
lipid catabolic process	3.99	0.018423	33.33	5	10	16042
regulation of carbohydrate utilization	3.89	0.0203891	50.00	3	3	43610
sequence-specific DNA binding	3.78	0.0227695	16.27	27	139	43565
transferase activity, transferring amino-acyl groups	3.68	0.0252319	36.36	4	7	16755
zinc ion transmembrane transport	3.68	0.0252319	36.36	4	7	71577
organic substance catabolic process	3.65	0.0259731	13.39	85	550	1901575
catabolic process	3.65	0.0259731	13.39	85	550	9056
fatty acid metabolic process	3.63	0.0264073	22.50	9	31	6631
copper ion transmembrane transport	3.40	0.0334326	66.67	2	1	35434
asparaginase activity	3.40	0.0334326	66.67	2	1	4067
arabinose transmembrane transport	3.40	0.0334326	66.67	2	1	15751
adenylate kinase activity	3.40	0.0334326	66.67	2	1	4017
glycolytic process through fructose-6phosphate	3.40	0.0334326	66.67	2	1	61615

metal ion transport	3.36	0.0346374	33.33	4	8	30001
response to external stimulus	3.26	0.0383326	19.64	11	45	9605
response to extracellular stimulus	3.26	0.0383326	19.64	11	45	9991
Mo-molybdopterin cofactor metabolic process	3.22	0.0398936	27.78	5	13	19720
molybdopterin cofactor metabolic process	3.22	0.0398936	27.78	5	13	43545
prosthetic group metabolic process	3.22	0.0398936	27.78	5	13	51189
Mo-molybdopterin cofactor biosynthetic process	3.22	0.0398936	27.78	5	13	6777
phosphorylation	3.20	0.0409098	25.00	6	18	16310
nucleoside phosphate catabolic process	3.20	0.0409098	25.00	6	18	1901292
nucleobase-containing small molecule biosynthetic process	3.20	0.0409098	25.00	6	18	34404
pyruvate biosynthetic process	3.20	0.0409098	25.00	6	18	42866
ADP metabolic process	3.20	0.0409098	25.00	6	18	46031
nucleotide phosphorylation	3.20	0.0409098	25.00	6	18	46939
glycolytic process	3.20	0.0409098	25.00	6	18	6096
nucleoside diphosphate phosphorylation	3.20	0.0409098	25.00	6	18	6165
ATP generation from ADP	3.20	0.0409098	25.00	6	18	6757
nucleoside diphosphate metabolic process	3.20	0.0409098	25.00	6	18	9132
purine nucleoside diphosphate metabolic process	3.20	0.0409098	25.00	6	18	9135
nucleotide catabolic process	3.20	0.0409098	25.00	6	18	9166

purine ribonucleoside diphosphate metabolic process	3.20	0.0409098	25.00	6	18	9179
ribonucleoside diphosphate metabolic process	3.20	0.0409098	25.00	6	18	9185
monosaccharide-transporting ATPase activity	3.08	0.0458073	30.77	4	9	15407
ribonuclease activity	3.08	0.0458073	30.77	4	9	4540
carbohydrate transport	3.08	0.0458073	30.77	4	9	8643
cellular response to extracellular stimulus	3.06	0.0468217	22.58	7	24	31668
cellular response to external stimulus	3.06	0.0468217	22.58	7	24	71496
cell communication	3.06	0.0468217	22.58	7	24	7154
SOS response	3.06	0.0468217	22.58	7	24	9432
6-sulfoquinovose(1-) metabolic process	3.03	0.048293	37.50	3	5	1902776
6-sulfoquinovose(1-) catabolic process	3.03	0.048293	37.50	3	5	1902777
sulfur compound catabolic process	3.03	0.048293	37.50	3	5	44273
lipopolysaccharide metabolic process	3.03	0.048293	37.50	3	5	8653
lipopolysaccharide biosynthetic process	3.03	0.048293	37.50	3	5	9103
N-terminal protein amino acid acetylation	3.01	0.0494195	26.32	5	14	6474
regulation of cellular process	2.99	0.0505175	15.48	24	131	50794
monocarboxylic acid metabolic process	2.90	0.0548944	16.10	19	99	32787
Gram-negative-bacterium-type cell wall biogenesis	2.83	0.0587409	28.57	4	10	43164

mRNA catabolic process	2.81	0.0601672	25.00	5	15	6402
fructose metabolic process	2.78	0.0620341	50.00	2	2	6000
3-hydroxypropionate dehydrogenase (NADP+) activity	2.78	0.0620341	50.00	2	2	35527
ferrous iron transport	2.78	0.0620341	50.00	2	2	15684
ferrous iron transmembrane transport	2.78	0.0620341	50.00	2	2	1903874
iron ion transmembrane transport	2.78	0.0620341	50.00	2	2	34755
fucose transmembrane transporter activity	2.78	0.0620341	50.00	2	2	15150
fucose transmembrane transport	2.78	0.0620341	50.00	2	2	15756
succinate transmembrane transporter activity	2.78	0.0620341	50.00	2	2	15141
fumarate transport	2.78	0.0620341	50.00	2	2	15741
glucuronate catabolic process	2.78	0.0620341	50.00	2	2	6064
response to nitric oxide	2.78	0.0620341	50.00	2	2	71731
nitrate transmembrane transporter activity	2.78	0.0620341	50.00	2	2	15112
nitrite transmembrane transporter activity	2.78	0.0620341	50.00	2	2	15113
basic amino acid transport	2.78	0.0620341	50.00	2	2	15802
arginine transport	2.78	0.0620341	50.00	2	2	15809
arginine transmembrane transport	2.78	0.0620341	50.00	2	2	1903826
basic amino acid transmembrane transport	2.78	0.0620341	50.00	2	2	1990822
cellular response to cadmium ion	2.78	0.0620341	50.00	2	2	71276
chromosome organization	2.78	0.0620341	50.00	2	2	51276

magnesium ion transport	2.78	0.0620341	50.00	2	2	15693
magnesium ion transmembrane transport	2.78	0.0620341	50.00	2	2	1903830
pantothenate biosynthetic process from valine	2.78	0.0620341	50.00	2	2	33317
channel activity	2.78	0.0620341	50.00	2	2	15267
passive transmembrane transporter activity	2.78	0.0620341	50.00	2	2	22803
voltage-gated channel activity	2.78	0.0620341	50.00	2	2	22832
gated channel activity	2.78	0.0620341	50.00	2	2	22836
ion gated channel activity	2.78	0.0620341	50.00	2	2	22839
voltage-gated ion channel activity	2.78	0.0620341	50.00	2	2	5244
pyruvate kinase activity	2.78	0.0620341	50.00	2	2	4743
response to reactive oxygen species	2.78	0.0620341	50.00	2	2	302
DNA methylation	2.71	0.066681	33.33	3	6	6306
biological regulation	2.61	0.0731745	14.69	26	151	65007
single-species biofilm formation on inanimate substrate	2.61	0.0734019	26.67	4	11	44011
regulation of biological process	2.51	0.0809456	14.72	24	139	50789
pyruvate metabolic process	2.50	0.0819687	20.00	7	28	6090
oxidoreductase activity, acting on the CH-OH group of donors, NAD or NADP as acceptor	2.49	0.0828396	19.05	8	34	16616
DNA binding	2.46	0.0850607	12.33	116	825	3677
response to zinc ion	2.43	0.0877363	30.00	3	7	10043
dipeptide transmembrane transport	2.43	0.0877363	30.00	3	7	35442
phosphoenolpyruvate-dependent sugar phosphotransferase system	2.43	0.0877363	30.00	3	7	9401

DNA synthesis involved in DNA repair	2.34	0.0959946	40.00	2	3	731
organic anion transport	2.34	0.0959946	40.00	2	3	15711
high-affinity secondary active nitrite transmembrane transporter activity	2.34	0.0959946	40.00	2	3	15513
UMP salvage	2.34	0.0959946	40.00	2	3	44206
translation initiation factor activity	2.34	0.0959946	40.00	2	3	3743
intrinsic component of periplasmic side of cell outer membrane	2.34	0.0959946	40.00	2	3	31246
endoribonuclease activity	2.31	0.099633	21.74	5	18	4521
alditol metabolic process	2.31	0.099633	21.74	5	18	19400
polyol metabolic process	2.31	0.099633	21.74	5	18	19751
glycerol metabolic process	2.31	0.099633	21.74	5	18	6071
glucose metabolic process	2.28	0.102788	18.18	8	36	6006
glutathione transferase activity	2.23	0.107616	23.53	4	13	4364
response to stress	2.21	0.109576	14.05	26	159	6950
lipid biosynthetic process	2.07	0.126119	16.39	10	51	8610
cellular response to hydrogen peroxide	2.06	0.126965	22.22	4	14	70301
monocarboxylic acid catabolic process	2.06	0.126965	22.22	4	14	72329
regulation of localization	2.03	0.131427	18.75	6	26	32879
response to hypochlorite	2.01	0.133797	33.33	2	4	1901530
response to reactive oxygen species	2.01	0.133797	33.33	2	4	302
response to oxidative stress	2.01	0.133797	33.33	2	4	6979
cadmium ion transmembrane transport	2.01	0.133797	33.33	2	4	70574

manganese ion transmembrane transport	2.01	0.133797	33.33	2	4	71421
RNA metabolic process	2.01	0.133797	33.33	2	4	16070
arginine biosynthetic process via ornithine	2.01	0.133797	33.33	2	4	42450
succinate transmembrane transport	2.01	0.133797	33.33	2	4	71422
D-tagatose 6-phosphate metabolic process	2.01	0.133797	33.33	2	4	2001058
D-tagatose 6-phosphate catabolic process	2.01	0.133797	33.33	2	4	2001059
negative regulation of single-species biofilm formation on inanimate substrate	2.01	0.133797	33.33	2	4	1900232
lysozyme inhibitor activity	2.01	0.133797	33.33	2	4	60241
nitrate transport	2.01	0.133797	33.33	2	4	15706
translational initiation	2.01	0.133797	33.33	2	4	6413
cysteine desulfurase activity	2.01	0.133797	33.33	2	4	31071
aspartate kinase activity	2.01	0.133797	33.33	2	4	4072
transferase activity, transferring pentosyl groups	1.99	0.136688	25.00	3	9	16763
RNA catabolic process	1.91	0.147644	21.05	4	15	6401
pentose-phosphate shunt, nonoxidative branch	1.81	0.163952	23.08	3	10	9052
acyl-CoA hydrolase activity	1.75	0.174193	28.57	2	5	47617
C4-dicarboxylate transmembrane transporter activity	1.75	0.174193	28.57	2	5	15556
C4-dicarboxylate transport	1.75	0.174193	28.57	2	5	15740

sulfate transmembrane transporter activity	1.75	0.174193	28.57	2	5	15116
glycerophosphodiester transmembrane transport	1.75	0.174193	28.57	2	5	1407
tRNA wobble position uridine thiolation	1.75	0.174193	28.57	2	5	2143
ethanolamine degradation polyhedral organelle	1.75	0.174193	28.57	2	5	31471
fatty acid beta-oxidation	1.75	0.174193	28.57	2	5	6635
cellular response to cell envelope stress	1.75	0.174193	28.57	2	5	36460
DNA dealkylation involved in DNA repair	1.75	0.174193	28.57	2	5	6307
L-serine catabolic process	1.75	0.174193	28.57	2	5	6565
D-gluconate catabolic process	1.75	0.174193	28.57	2	5	46177
hexose metabolic process	1.73	0.176747	16.00	8	42	19318
monosaccharide metabolic process	1.73	0.176747	16.00	8	42	5996
Mo-molybdopterin cofactor biosynthetic process	1.65	0.192426	19.05	4	17	6777
cell adhesion	1.65	0.192925	14.14	14	85	7155
negative regulation of cellular process	1.63	0.196671	16.67	6	30	48523
nicotinamide nucleotide biosynthetic process	1.58	0.205564	15.38	8	44	19359
pyridine nucleotide biosynthetic process	1.58	0.205564	15.38	8	44	19363
ribokinase activity	1.53	0.21616	25.00	2	6	4747
negative regulation of organelle organization	1.53	0.21616	25.00	2	6	10639
recombinational repair	1.53	0.21616	25.00	2	6	725

regulation of cell division	1.53	0.21616	25.00	2	6	51302
translational attenuation	1.53	0.21616	25.00	2	6	9386
L-fucose catabolic process	1.53	0.21616	25.00	2	6	42355
hexose biosynthetic process	1.53	0.216232	18.18	4	18	19319
monosaccharide biosynthetic process	1.53	0.216232	18.18	4	18	46364
gluconeogenesis	1.53	0.216232	18.18	4	18	6094
glutamate metabolic process	1.50	0.222457	20.00	3	12	6536
glutamate biosynthetic process	1.50	0.222457	20.00	3	12	6537
alcohol metabolic process	1.48	0.227524	16.67	5	25	6066
negative regulation of biological process	1.46	0.233076	15.79	6	32	48519
periplasmic space	1.41	0.243294	12.87	22	149	42597
pyridine nucleotide metabolic process	1.38	0.251908	14.55	8	47	19362
nicotinamide nucleotide metabolic process	1.38	0.251908	14.55	8	47	46496
oxidoreduction coenzyme metabolic process	1.38	0.251908	14.55	8	47	6733
DNA recombination	1.37	0.25428	13.27	15	98	6310
DNA methylation on adenine	1.35	0.258868	22.22	2	7	32775
negative regulation of DNA-binding transcription factor activity	1.35	0.258868	22.22	2	7	43433
cellular response to heat	1.35	0.258868	22.22	2	7	34605
posttranscriptional regulation of gene expression	1.35	0.258868	22.22	2	7	10608
regulation of cellular protein metabolic process	1.35	0.258868	22.22	2	7	32268
regulation of cellular amide metabolic process	1.35	0.258868	22.22	2	7	34248

regulation of protein metabolic process	1.35	0.258868	22.22	2	7	51246
regulation of translation	1.35	0.258868	22.22	2	7	6417
organic acid catabolic process	1.34	0.260711	13.11	16	106	16054
L-amino acid transport	1.31	0.270264	14.58	7	41	15807
small molecule catabolic process	1.30	0.271569	13.01	16	107	44282
regulation of macromolecule biosynthetic process	1.27	0.282141	13.08	14	93	10556
regulation of metabolic process	1.27	0.282141	13.08	14	93	19222
regulation of cellular metabolic process	1.27	0.282141	13.08	14	93	31323
regulation of cellular biosynthetic process	1.27	0.282141	13.08	14	93	31326
regulation of nitrogen compound metabolic process	1.27	0.282141	13.08	14	93	51171
regulation of macromolecule metabolic process	1.27	0.282141	13.08	14	93	60255
regulation of primary metabolic process	1.27	0.282141	13.08	14	93	80090
regulation of biosynthetic process	1.27	0.282141	13.08	14	93	9889
response to nutrient levels	1.23	0.291501	16.00	4	21	31667
response to starvation	1.23	0.291501	16.00	4	21	42594
regulation of translation	1.23	0.292626	15.15	5	28	6417
phosphopyruvate hydratase complex	1.22	0.294377	33.33	1	2	15
phosphopyruvate hydratase activity	1.22	0.294377	33.33	1	2	4634
CTP biosynthetic process	1.22	0.294377	33.33	1	2	6241
glycine biosynthetic process	1.22	0.294377	33.33	1	2	6545
'de novo' CTP biosynthetic process	1.22	0.294377	33.33	1	2	44210

fructose catabolic process	1.22	0.294377	33.33	1	2	6001
organophosphate:inorganic phosphate antiporter activity	1.22	0.294377	33.33	1	2	15315
type III protein secretion system complex	1.22	0.294377	33.33	1	2	30257
protein autoprocessing	1.22	0.294377	33.33	1	2	16540
homocysteine metabolic process	1.22	0.294377	33.33	1	2	50667
lysine catabolic process	1.22	0.294377	33.33	1	2	6554
galactitol transmembrane transporter activity	1.22	0.294377	33.33	1	2	15577
malate synthase activity	1.22	0.294377	33.33	1	2	4474
fatty acid binding	1.22	0.294377	33.33	1	2	5504
DNA replication proofreading	1.22	0.294377	33.33	1	2	45004
	1.22	0.294377	33.33	1	2	42967
monosaccharide transmembrane transport	1.22	0.294377	33.33	1	2	15749
carbohydrate transmembrane transport	1.22	0.294377	33.33	1	2	34219
transcriptional attenuation by ribosome	1.22	0.294377	33.33	1	2	31556
acetate transmembrane transport	1.22	0.294377	33.33	1	2	35433
amino acid transmembrane transport	1.20	0.30116	13.79	8	50	3333
amino acid transport	1.20	0.30116	13.79	8	50	6865
fatty-acyl-CoA binding	1.20	0.301651	20.00	2	8	62
D-ribose metabolic process	1.20	0.301651	20.00	2	8	6014
phosphatidylglycerol biosynthetic process	1.20	0.301651	20.00	2	8	6655
glycerophospholipid biosynthetic process	1.20	0.301651	20.00	2	8	46474

nickel cation transmembrane transport	1.20	0.301651	20.00	2	8	35444
alcohol dehydrogenase (NAD) activity	1.20	0.301651	20.00	2	8	4022
phospholipid metabolic process	1.18	0.306264	14.00	7	43	6644
phospholipid biosynthetic process	1.18	0.306264	14.00	7	43	8654
recombinase activity	1.07	0.343979	18.18	2	9	150
succinate dehydrogenase activity	1.07	0.343979	18.18	2	9	104
regulation of neurotransmitter levels	1.07	0.343979	18.18	2	9	1505
neurotransmitter metabolic process	1.07	0.343979	18.18	2	9	42133
regulation of biological quality	1.07	0.343979	18.18	2	9	65008
ammonium ion metabolic process	1.07	0.343979	18.18	2	9	97164
3'-5'-exoribonuclease activity	1.07	0.343979	18.18	2	9	175
aspartate metabolic process	1.07	0.343979	18.18	2	9	6531
aspartate biosynthetic process	1.07	0.343979	18.18	2	9	6532
efflux transmembrane transporter activity	1.07	0.343979	18.18	2	9	15562
fructose transmembrane transport	1.07	0.343979	18.18	2	9	15755
NAD+ binding	1.07	0.343979	18.18	2	9	70403
intrinsic component of the cytoplasmic side of the plasma membrane	1.07	0.343979	18.18	2	9	31235
methionine metabolic process	1.06	0.347234	15.79	3	16	6555
methionine biosynthetic process	1.06	0.347234	15.79	3	16	9086
glyceraldehyde-3-phosphate metabolic process	1.06	0.347234	15.79	3	16	19682
organic anion transport	1.04	0.35233	13.11	8	53	15711
carboxylic acid transmembrane transport	1.04	0.35233	13.11	8	53	1905039

carboxylic acid transport	1.04	0.35233	13.11	8	53	46942
anion transport	1.01	0.365675	12.66	10	69	6820
anion transmembrane transport	1.01	0.365675	12.66	10	69	98656
integral component of membrane	1.00	0.368653	11.20	210	1665	16021
fermentation	1.00	0.369639	12.90	8	54	6113
dihydrofolate reductase activity	0.99	0.371825	25.00	1	3	4146
phosphate ion homeostasis	0.99	0.371825	25.00	1	3	55062
conjugation	0.99	0.371825	25.00	1	3	746
alkanesulfonate catabolic process	0.99	0.371825	25.00	1	3	46306
phosphopantetheine binding	0.99	0.371825	25.00	1	3	31177
D-xylose transmembrane transport	0.99	0.371825	25.00	1	3	15753
alpha,alpha-trehalase activity	0.99	0.371825	25.00	1	3	4555
ATP metabolic process	0.99	0.371993	13.33	6	39	46034
ATP biosynthetic process	0.99	0.371993	13.33	6	39	6754
nucleoside monophosphate metabolic process	0.99	0.371993	13.33	6	39	9123
nucleoside monophosphate biosynthetic process	0.99	0.371993	13.33	6	39	9124
purine nucleoside monophosphate metabolic process	0.99	0.371993	13.33	6	39	9126
purine nucleoside monophosphate biosynthetic process	0.99	0.371993	13.33	6	39	9127
nucleoside triphosphate metabolic process	0.99	0.371993	13.33	6	39	9141
nucleoside triphosphate biosynthetic process	0.99	0.371993	13.33	6	39	9142
purine nucleoside triphosphate metabolic process	0.99	0.371993	13.33	6	39	9144

purine nucleoside triphosphate biosynthetic process	0.99	0.371993	13.33	6	39	9145
ribonucleoside monophosphate biosynthetic process	0.99	0.371993	13.33	6	39	9156
ribonucleoside monophosphate metabolic process	0.99	0.371993	13.33	6	39	9161
purine ribonucleoside monophosphate metabolic process	0.99	0.371993	13.33	6	39	9167
purine ribonucleoside monophosphate biosynthetic process	0.99	0.371993	13.33	6	39	9168
ribonucleoside triphosphate metabolic process	0.99	0.371993	13.33	6	39	9199
ribonucleoside triphosphate biosynthetic process	0.99	0.371993	13.33	6	39	9201
purine ribonucleoside triphosphate metabolic process	0.99	0.371993	13.33	6	39	9205
purine ribonucleoside triphosphate biosynthetic process	0.99	0.371993	13.33	6	39	9206
regulation of nucleic acid-templated transcription	0.98	0.374931	12.37	12	85	1903506
regulation of RNA biosynthetic process	0.98	0.374931	12.37	12	85	2001141
regulation of RNA metabolic process	0.98	0.374931	12.37	12	85	51252
protein autophosphorylation	0.96	0.383175	13.51	5	32	46777
organic acid transport	0.95	0.387014	12.70	8	55	15849
organic acid transmembrane transport	0.95	0.387014	12.70	8	55	1903825
regulation of gene expression	0.94	0.388842	12.24	12	86	10468
ribosome binding	0.93	0.395589	13.79	4	25	43022

regulation of nucleobase-containing compound metabolic process	0.91	0.402796	12.12	12	87	19219
monocarboxylic acid biosynthetic process	0.88	0.412918	12.77	6	41	72330
L-alpha-amino acid transmembrane transport	0.88	0.412918	12.77	6	41	1902475
regulation of cellular macromolecule biosynthetic process	0.88	0.416774	12.00	12	88	2000112
serine-type endopeptidase activity	0.86	0.421445	13.33	4	26	4252
pyridine-containing compound biosynthetic process	0.86	0.421823	12.31	8	57	72525
glycerol metabolic process	0.85	0.425707	15.38	2	11	6071
protein ubiquitination	0.85	0.425707	15.38	2	11	16567
negative regulation of bacterial-type flagellum-dependent cell motility	0.85	0.425707	15.38	2	11	1902201
negative regulation of cell motility	0.85	0.425707	15.38	2	11	2000146
negative regulation of locomotion	0.85	0.425707	15.38	2	11	40013
negative regulation of cellular component movement	0.85	0.425707	15.38	2	11	51271
phenylacetate catabolic process	0.85	0.425707	15.38	2	11	10124
response to abiotic stimulus	0.84	0.433216	11.76	14	105	9628
fatty acid metabolic process	0.84	0.433297	12.50	6	42	6631
DNA-dependent DNA replication	0.82	0.440161	13.64	3	19	6261
succinate-semialdehyde dehydrogenase (NAD+) activity	0.82	0.440782	20.00	1	4	4777
cobalamin-transporting ATPase activity	0.82	0.440782	20.00	1	4	15420

vitamin transmembrane transport	0.82	0.440782	20.00	1	4	35461
protein secretion by the type III secretion system	0.82	0.440782	20.00	1	4	30254
metallochaperone activity	0.82	0.440782	20.00	1	4	16530
S-adenosylmethionine metabolic process	0.82	0.440782	20.00	1	4	46500
S-adenosylmethionine biosynthetic process	0.82	0.440782	20.00	1	4	6556
amino-acid betaine biosynthetic process	0.82	0.440782	20.00	1	4	6578
heat shock protein binding	0.82	0.440782	20.00	1	4	31072
pyruvate catabolic process	0.82	0.440782	20.00	1	4	42867
galactokinase activity	0.82	0.440782	20.00	1	4	4335
enoyl-CoA hydratase activity	0.82	0.440782	20.00	1	4	4300
translation release factor activity	0.82	0.440782	20.00	1	4	3747
cell	0.81	0.447026	12.90	4	27	5623
organophosphate metabolic process	0.80	0.447911	11.39	27	210	19637
phosphate-containing compound metabolic process	0.80	0.447911	11.39	27	210	6796
transcription, DNA-templated	0.80	0.451122	11.18	68	540	6351
membrane	0.77	0.464471	11.04	262	2112	16020
sulfate assimilation	0.77	0.46455	14.29	2	12	103
sulfate transmembrane transport	0.77	0.46455	14.29	2	12	1902358
sulfate transport	0.77	0.46455	14.29	2	12	8272
regulation of organelle organization	0.77	0.46455	14.29	2	12	33043
ion transmembrane transport	0.75	0.470068	13.04	3	20	34220

pyridine-containing compound metabolic process	0.75	0.473683	11.76	8	60	72524
regulation of transcription, DNAtemplated	0.72	0.484494	11.08	72	578	6355
coenzyme biosynthetic process	0.72	0.484628	11.38	14	109	9108
carboxylic acid catabolic process	0.72	0.485508	11.40	13	101	46395
hydrolase activity, hydrolyzing Oglycosyl compounds	0.70	0.495138	11.90	5	37	4553
oxidoreductase activity, acting on CHOH group of donors	0.70	0.497017	12.12	4	29	16614
regulation of bacterial-type flagellumdependent cell motility	0.69	0.501795	13.33	2	13	1902021
monosaccharide transmembrane transporter activity	0.69	0.501795	13.33	2	13	15145
purine nucleotide metabolic process	0.69	0.502179	16.67	1	5	6163
polysaccharide catabolic process	0.69	0.502179	16.67	1	5	272
phosphorylase activity	0.69	0.502179	16.67	1	5	4645
sulfathiazole transmembrane transporter activity	0.69	0.502179	16.67	1	5	15546
sulfathiazole transport	0.69	0.502179	16.67	1	5	15906
sulfathiazole transmembrane transport	0.69	0.502179	16.67	1	5	1902599
xenobiotic transport	0.69	0.502179	16.67	1	5	42908
azole transport	0.69	0.502179	16.67	1	5	45117
regulation of DNA-templated transcription, termination	0.69	0.502179	16.67	1	5	31554
locomotion	0.68	0.507667	11.43	8	62	40011
taxis	0.68	0.507667	11.43	8	62	42330

transferase activity, transferring glycosyl groups	0.66	0.516076	11.22	11	87	16757
coenzyme metabolic process	0.65	0.522642	11.11	14	112	6732
response to stimulus	0.64	0.52531	10.98	37	300	50896
DNA recombination	0.64	0.52756	12.00	3	22	6310
liposaccharide metabolic process	0.64	0.52756	12.00	3	22	1903509
amino acid transmembrane transporter activity	0.63	0.532442	11.32	6	47	15171
regulation of transcription, DNAtemplated	0.63	0.533347	11.11	10	80	6355
fermentation	0.62	0.537325	12.50	2	14	6113
ATP-dependent DNA helicase activity	0.62	0.537325	12.50	2	14	4003
uracil catabolic process	0.62	0.537325	12.50	2	14	6212
molybdopterin cofactor binding	0.62	0.537325	12.50	2	14	43546
ribosomal small subunit binding	0.62	0.537325	12.50	2	14	43024
negative regulation of cellular component organization	0.62	0.537325	12.50	2	14	51129
nucleobase-containing small molecule interconversion	0.60	0.548018	10.99	10	81	15949
ribose phosphate metabolic process	0.59	0.55229	10.98	9	73	19693
response to silver ion	0.59	0.556844	14.29	1	6	10272
cellular metabolic process	0.59	0.556844	14.29	1	6	44237
molybdopterin cofactor biosynthetic process	0.59	0.556844	14.29	1	6	32324
secondary metabolite biosynthetic process	0.59	0.556844	14.29	1	6	44550
ion channel activity	0.59	0.556844	14.29	1	6	5216
cellular protein modification process	0.54	0.581414	11.11	3	24	6464

sulfur amino acid metabolic process	0.54	0.581414	11.11	3	24	96
sulfur amino acid biosynthetic process	0.54	0.581414	11.11	3	24	97
ion transmembrane transporter activity	0.54	0.581414	11.11	3	24	15075
cellular catabolic process	0.54	0.582538	10.71	24	200	44248
cellular amino acid catabolic process	0.51	0.598709	10.58	11	93	9063
transporter activity	0.51	0.60049	10.66	34	285	5215
inorganic anion transport	0.51	0.602985	11.11	2	16	15698
inorganic anion transmembrane transport	0.51	0.602985	11.11	2	16	98661
nucleoside-triphosphatase activity	0.50	0.605513	12.50	1	7	17111
erythrose 4phosphate/phosphoenolpyruvate family amino acid metabolic process	0.50	0.605513	12.50	1	7	1902221
erythrose 4phosphate/phosphoenolpyruvate family amino acid biosynthetic process	0.50	0.605513	12.50	1	7	1902223
L-phenylalanine metabolic process	0.50	0.605513	12.50	1	7	6558
L-phenylalanine biosynthetic process	0.50	0.605513	12.50	1	7	9094
antibiotic transport	0.50	0.605513	12.50	1	7	42891
detoxification of copper ion	0.50	0.605513	12.50	1	7	10273
motor activity	0.50	0.605513	12.50	1	7	3774
ethanolamine catabolic process	0.50	0.606837	10.71	3	25	46336
aromatic compound catabolic process	0.48	0.619744	10.39	8	69	19439
nucleobase-containing compound catabolic process	0.48	0.619744	10.39	8	69	34655
dephosphorylation	0.48	0.619744	10.39	8	69	16311

ion transmembrane transport	0.47	0.625393	10.42	15	129	34220
organophosphate biosynthetic process	0.47	0.62577	10.48	22	188	90407
phosphorus metabolic process	0.47	0.626127	10.53	30	255	6793
polyamine metabolic process	0.46	0.631212	10.34	3	26	6595
sulfur compound transport	0.46	0.631212	10.34	3	26	72348
transmembrane transporter activity	0.46	0.631212	10.34	3	26	22857
transporter activity	0.46	0.631212	10.34	3	26	5215
arginine metabolic process	0.46	0.633073	10.53	2	17	6525
DNA-templated transcription, initiation	0.46	0.633073	10.53	2	17	6352
inorganic cation transmembrane transporter activity	0.43	0.648844	11.11	1	8	22890
metal ion transmembrane transporter activity	0.43	0.648844	11.11	1	8	46873
aldehyde catabolic process	0.43	0.648844	11.11	1	8	46185
glyoxylate catabolic process	0.43	0.648844	11.11	1	8	9436
tryptophan biosynthetic process	0.43	0.648844	11.11	1	8	162
drug:proton antiporter activity	0.43	0.648844	11.11	1	8	15307
metal ion transport	0.43	0.653389	10.00	4	36	30001
response to antibiotic	0.42	0.657818	10.20	15	132	46677
phosphoprotein phosphatase activity	0.41	0.661348	10.00	2	18	4721
aminopeptidase activity	0.41	0.661348	10.00	2	18	4177
pyrimidine nucleotide metabolic process	0.41	0.661348	10.00	2	18	6220
pyrimidine nucleotide biosynthetic process	0.41	0.661348	10.00	2	18	6221
positive regulation of biological process	0.41	0.661348	10.00	2	18	48518

positive regulation of cellular process	0.41	0.661348	10.00	2	18	48522
DNA integration	0.39	0.674877	9.86	7	64	15074
organic substance transport	0.38	0.681506	10.00	13	117	71702
DNA-binding transcription factor activity	0.38	0.683567	10.33	38	330	3700
aromatic amino acid family biosynthetic process, prephenate pathway	0.37	0.687421	10.00	1	9	9095
terpenoid biosynthetic process	0.37	0.687421	10.00	1	9	16114
response to radiation	0.37	0.687421	10.00	1	9	9314
sodium ion transmembrane transport	0.37	0.687421	10.00	1	9	35725
cellular response to stress	0.37	0.689302	9.72	7	65	33554
cellular response to stimulus	0.37	0.689302	9.72	7	65	51716
cellular response to DNA damage stimulus	0.37	0.689302	9.72	7	65	6974
DNA replication	0.37	0.6927	9.78	9	83	6260
organic hydroxy compound metabolic process	0.35	0.705377	9.43	5	48	190161 5
defense response to bacterium	0.34	0.712614	9.09	2	20	42742
glycoprotein metabolic process	0.34	0.712614	9.09	2	20	9100
glycoprotein biosynthetic process	0.34	0.712614	9.09	2	20	9101
cytoplasm	0.34	0.715009	10.57	141	1193	5737
amino acid-transporting ATPase activity	0.33	0.721765	9.09	1	10	15424
cellular amine metabolic process	0.32	0.726673	9.09	4	40	44106
cellular biogenic amine metabolic process	0.32	0.726673	9.09	4	40	6576
cellular aldehyde metabolic process	0.32	0.726673	9.09	4	40	6081

iron ion binding	0.31	0.730327	9.57	11	104	5506
integral component of external side of plasma membrane	0.31	0.735713	8.70	2	21	71575
inorganic ion transmembrane transport	0.30	0.742789	9.21	7	69	98660
cation transmembrane transporter activity	0.28	0.75234	8.33	1	11	8324
glutamine metabolic process	0.28	0.75234	8.33	1	11	6541
glutamine biosynthetic process	0.28	0.75234	8.33	1	11	6542
translational termination	0.28	0.75234	8.33	1	11	6415
cofactor biosynthetic process	0.28	0.752976	9.52	14	133	51188
oxidoreductase activity, acting on the aldehyde or oxo group of donors, NAD or NADP as acceptor	0.28	0.755038	8.57	3	32	16620
protein dephosphorylation	0.28	0.757211	8.33	2	22	6470
organic cyclic compound catabolic process	0.27	0.764091	9.09	8	80	190136 1
heterocycle catabolic process	0.27	0.764091	9.09	8	80	46700
transmembrane transport	0.27	0.764935	9.55	17	161	55085
amino acid transmembrane transport	0.27	0.766971	8.97	7	71	3333
extracellular region	0.26	0.772037	8.33	3	33	5576
fatty acid biosynthetic process	0.26	0.772037	8.33	3	33	6633
drug transport	0.25	0.777181	8.00	2	23	15893
2 iron, 2 sulfur cluster binding	0.25	0.777882	8.62	5	53	51537
cofactor metabolic process	0.25	0.778873	9.33	14	136	51186
amide transport	0.25	0.77956	7.69	1	12	42886
intrinsic component of periplasmic side of plasma membrane	0.25	0.77956	7.69	1	12	31237

ribose phosphate biosynthetic process	0.24	0.783392	8.70	6	63	46390
purine ribonucleotide metabolic process	0.24	0.783392	8.70	6	63	9150
purine ribonucleotide biosynthetic process	0.24	0.783392	8.70	6	63	9152
ribonucleotide metabolic process	0.24	0.783392	8.70	6	63	9259
ribonucleotide biosynthetic process	0.24	0.783392	8.70	6	63	9260
cell redox homeostasis	0.24	0.788059	8.11	3	34	45454
flavin adenine dinucleotide binding	0.24	0.790359	9.01	10	101	50660
inorganic cation transmembrane transport	0.23	0.790599	8.47	5	54	98662
heme binding	0.23	0.790599	8.47	5	54	20037
xenobiotic metabolic process	0.22	0.802742	8.33	5	55	6805
pyrimidine nucleobase metabolic process	0.22	0.803791	7.14	1	13	6206
phosphate ion transmembrane transport	0.22	0.803791	7.14	1	13	35435
amino-acid betaine metabolic process	0.22	0.803791	7.14	1	13	6577
inorganic molecular entity transmembrane transporter activity	0.22	0.803791	7.14	1	13	15318
threonine metabolic process	0.22	0.803791	7.14	1	13	6566
threonine biosynthetic process	0.22	0.803791	7.14	1	13	9088
kinase activity	0.22	0.805956	9.59	28	264	16301
response to acid chemical	0.21	0.812841	7.41	2	25	1101
protein histidine kinase activity	0.21	0.813942	8.00	4	46	4673
manganese ion binding	0.20	0.814995	8.60	8	85	30145
phosphorylation	0.20	0.816967	9.52	28	266	16310
response to drug	0.20	0.81997	8.65	9	95	42493

tryptophan biosynthetic process	0.19	0.825362	6.67	1	14	162
indole-containing compound metabolic process	0.19	0.825362	6.67	1	14	42430
indole-containing compound biosynthetic process	0.19	0.825362	6.67	1	14	42435
indolalkylamine biosynthetic process	0.19	0.825362	6.67	1	14	46219
tryptophan metabolic process	0.19	0.825362	6.67	1	14	6568
indolalkylamine metabolic process	0.19	0.825362	6.67	1	14	6586
aspartate family amino acid metabolic process	0.19	0.826803	8.22	6	67	9066
aspartate family amino acid biosynthetic process	0.19	0.826803	8.22	6	67	9067
transferase activity	0.19	0.827628	10.15	100	885	16740
protein maturation	0.19	0.828687	7.14	2	26	51604
NAD metabolic process	0.19	0.828687	7.14	2	26	19674
NAD biosynthetic process	0.19	0.828687	7.14	2	26	9435
cytosolic small ribosomal subunit	0.19	0.828687	7.14	2	26	22627
NADP binding	0.19	0.830591	7.50	3	37	50661
nitrogen compound transport	0.18	0.836634	8.49	9	97	71705
response to inorganic substance	0.17	0.843315	6.90	2	27	10035
copper ion binding	0.17	0.843315	6.90	2	27	5507
histidine biosynthetic process	0.17	0.844565	6.25	1	15	105
response to oxygen-containing compound	0.15	0.856801	6.67	2	28	1901700
rRNA base methylation	0.15	0.856801	6.67	2	28	70475
cellular macromolecule catabolic process	0.15	0.85837	7.41	4	50	44265
ATP biosynthetic process	0.15	0.861658	5.88	1	16	6754

aromatic amino acid family metabolic process	0.14	0.865583	6.98	3	40	9072
aromatic amino acid family biosynthetic process	0.14	0.865583	6.98	3	40	9073
pilus organization	0.14	0.869218	6.45	2	29	43711
endopeptidase activity	0.14	0.869218	6.45	2	29	4175
nucleobase-containing small molecule metabolic process	0.14	0.87043	8.98	22	223	55086
cation transmembrane transport	0.14	0.870765	7.87	7	82	98655
pyrimidine nucleotide biosynthetic process	0.13	0.876875	5.56	1	17	6221
glyoxylate metabolic process	0.13	0.876875	5.56	1	17	46487
pseudouridine synthesis	0.13	0.876875	5.56	1	17	1522
histidine biosynthetic process	0.13	0.876875	5.56	1	17	105
imidazole-containing compound metabolic process	0.13	0.876875	5.56	1	17	52803
histidine metabolic process	0.13	0.876875	5.56	1	17	6547
nucleoside phosphate biosynthetic process	0.13	0.879752	8.33	12	132	1901293
nucleotide biosynthetic process	0.13	0.879752	8.33	12	132	9165
cellular nitrogen compound catabolic process	0.12	0.884844	7.84	8	94	44270
dicarboxylic acid biosynthetic process	0.12	0.885224	6.67	3	42	43650
phosphorelay sensor kinase activity	0.12	0.885456	7.02	4	53	155
signal transduction by protein phosphorylation	0.12	0.885456	7.02	4	53	23014
rRNA processing	0.12	0.888427	7.25	5	64	6364
diguanylate cyclase activity	0.12	0.890419	5.26	1	18	52621

arginine biosynthetic process	0.12	0.890419	5.26	1	18	6526
response to chemical	0.11	0.893942	8.09	11	125	42221
nucleoside phosphate metabolic process	0.11	0.895628	8.16	12	135	6753
nucleotide metabolic process	0.11	0.895628	8.16	12	135	9117
phosphorelay signal transduction system	0.11	0.895628	8.16	12	135	160
cell division site	0.10	0.900763	5.88	2	32	32153
oxidoreductase activity, acting on NAD(P)H	0.10	0.902475	5.00	1	19	16651
single-species biofilm formation	0.10	0.907915	6.67	4	56	44010
ion transport	0.09	0.910072	8.24	15	167	6811
transition metal ion transport	0.09	0.917687	5.56	2	34	41
iron ion transport	0.09	0.917687	5.56	2	34	6826
drug transmembrane transport	0.08	0.922759	4.55	1	21	6855
response to temperature stimulus	0.08	0.925094	5.41	2	35	9266
phosphorelay response regulator activity	0.08	0.925094	5.41	2	35	156
sulfur compound metabolic process	0.08	0.925688	7.58	10	122	6790
DNA replication	0.08	0.925847	6.67	5	70	6260
DNA-dependent DNA replication	0.08	0.925847	6.67	5	70	6261
transferase activity, transferring phosphorus-containing groups	0.08	0.926378	6.35	4	59	16772
glutamine metabolic process	0.07	0.931261	4.35	1	22	6541
cytochrome complex assembly	0.07	0.931261	4.35	1	22	17004
regulation of cellular component organization	0.07	0.931869	5.26	2	36	51128
localization	0.07	0.93481	8.26	20	222	51179

establishment of localization	0.07	0.93481	8.26	20	222	51234
transport	0.07	0.93481	8.26	20	222	6810
cell division	0.07	0.935278	7.14	8	104	51301
pyrimidine-containing compound metabolic process	0.06	0.938062	5.13	2	37	72527
pyrimidine-containing compound biosynthetic process	0.06	0.938062	5.13	2	37	72528
nucleic acid binding	0.06	0.942928	7.65	13	157	3676
protein modification process	0.06	0.944718	6.80	7	96	36211
cellular protein modification process	0.06	0.944718	6.80	7	96	6464
cellular modified amino acid biosynthetic process	0.06	0.945564	4.00	1	24	42398
leucine metabolic process	0.06	0.945564	4.00	1	24	6551
branched-chain amino acid metabolic process	0.06	0.945564	4.00	1	24	9081
branched-chain amino acid biosynthetic process	0.06	0.945564	4.00	1	24	9082
leucine biosynthetic process	0.06	0.945564	4.00	1	24	9098
peroxidase activity	0.06	0.945564	4.00	1	24	4601
macromolecule catabolic process	0.06	0.9458	5.97	4	63	9057
DNA metabolic process	0.06	0.945954	7.69	14	168	6259
chaperone-mediated protein folding	0.05	0.948881	4.88	2	39	61077
protein disulfide oxidoreductase activity	0.05	0.951559	3.85	1	25	15035
transcription regulatory region DNA binding	0.05	0.951559	3.85	1	25	44212
cellular biogenic amine biosynthetic process	0.05	0.953591	4.76	2	40	42401

amine biosynthetic process	0.05	0.953591	4.76	2	40	9309
electron transfer activity	0.05	0.953591	4.76	2	40	9055
cell projection	0.05	0.953642	5.80	4	65	42995
pilus	0.05	0.953642	5.80	4	65	9289
amine catabolic process	0.05	0.953959	5.36	3	53	9310
intracellular	0.05	0.954814	7.86	18	211	5622
polyamine biosynthetic process	0.04	0.956894	3.70	1	26	6596
transferase activity, transferring acyl groups	0.04	0.95738	6.54	7	100	16746
purine nucleotide metabolic process	0.04	0.957402	6.32	6	89	6163
purine nucleotide biosynthetic process	0.04	0.957402	6.32	6	89	6164
purine-containing compound biosynthetic process	0.04	0.957402	6.32	6	89	72522
oxidoreductase activity	0.04	0.957884	4.65	2	41	16491
nucleobase-containing compound biosynthetic process	0.04	0.958597	7.27	12	153	34654
purine-containing compound metabolic process	0.04	0.960268	6.25	6	90	72521
glutamine family amino acid metabolic process	0.04	0.960428	5.63	4	67	9064
glutamine family amino acid biosynthetic process	0.04	0.960428	5.63	4	67	9084
oxidation-reduction process	0.04	0.962363	9.25	77	755	55114
signal transduction	0.04	0.963465	5.56	4	68	7165
exonuclease activity	0.03	0.968598	4.35	2	44	4527
amine metabolic process	0.03	0.971324	5.68	5	83	9308
cell wall organization	0.03	0.971393	6.40	8	117	71555
primary metabolic process	0.02	0.976009	9.71	163	1516	44238

cellular amino acid metabolic process	0.02	0.976662	4.08	2	47	6520
DNA repair	0.02	0.979156	4.62	3	62	6281
organonitrogen compound catabolic process	0.02	0.980248	6.74	12	166	1901565
cellular modified amino acid metabolic process	0.02	0.980964	2.94	1	33	6575
nickel cation binding	0.02	0.980964	2.94	1	33	16151
transferase activity, transferring acyl groups other than amino-acyl groups	0.02	0.983063	2.86	1	34	16747
organic acid metabolic process	0.02	0.98364	8.30	42	464	6082
catalytic activity	0.02	0.984111	4.41	3	65	3824
cellular carbohydrate metabolic process	0.01	0.985406	5.93	8	127	44262
polysaccharide biosynthetic process	0.01	0.985659	5.45	6	104	271
carbohydrate biosynthetic process	0.01	0.986011	6.25	10	150	16051
protein metabolic process	0.01	0.988392	7.03	18	238	19538
organelle inner membrane	0.01	0.988616	8.86	79	813	19866
organelle membrane	0.01	0.988616	8.86	79	813	31090
cation transport	0.01	0.989467	5.51	7	120	6812
dicarboxylic acid metabolic process	0.01	0.989963	4.11	3	70	43648
iron-sulfur cluster binding	0.01	0.992088	6.84	18	245	51536
carboxylic acid metabolic process	0.01	0.992252	7.91	39	454	19752
oxoacid metabolic process	0.01	0.992252	7.91	39	454	43436
structural constituent of ribosome	0.01	0.992319	3.33	2	58	3735
structural molecule activity	0.01	0.992319	3.33	2	58	5198
macromolecule modification	0.01	0.992657	6.04	11	171	43412
organelle part	0.01	0.993037	8.71	79	828	44422
GTP binding	0.01	0.993205	4.35	4	88	5525
sulfur compound biosynthetic process	0.01	0.99371	3.85	3	75	44272

structural constituent of ribosome	0.01	0.993746	3.23	2	60	3735
protein binding	0.01	0.994181	8.91	100	1022	5515
aerobic respiration	0.01	0.994276	3.80	3	76	9060
polysaccharide metabolic process	0.01	0.994343	4.92	6	116	5976
oxidation-reduction process	0.01	0.994631	7.10	24	314	55114
small molecule metabolic process	0.00	0.995978	8.38	68	743	44281
molecular_function	0.00	0.996003	5.23	8	145	3674
translation	0.00	0.99678	4.65	6	123	6412
cytochrome complex assembly	0.00	0.997683	1.92	1	51	17004
cellular protein-containing complex assembly	0.00	0.997683	1.92	1	51	34622
protein-containing complex subunit organization	0.00	0.997683	1.92	1	51	43933
protein-containing complex assembly	0.00	0.997683	1.92	1	51	65003
organic acid biosynthetic process	0.00	0.997797	6.32	18	267	16053
carboxylic acid biosynthetic process	0.00	0.997797	6.32	18	267	46394
monovalent inorganic cation transport	0.00	0.997939	1.89	1	52	15672
proton transmembrane transport	0.00	0.997939	1.89	1	52	1902600
pyridoxal phosphate binding	0.00	0.998192	3.30	3	88	30170
ATP binding	0.00	0.998211	7.97	58	670	5524
protein transport	0.00	0.998387	3.70	4	104	15031
alpha-amino acid metabolic process	0.00	0.998409	5.53	12	205	1901605
alpha-amino acid biosynthetic process	0.00	0.998409	5.53	12	205	1901607
cellular amino acid biosynthetic process	0.00	0.998516	5.50	12	206	8652

cellular amino acid metabolic process	0.00	0.998555	6.49	22	317	6520
cellular component assembly	0.00	0.99871	1.75	1	56	22607
external encapsulating structure	0.00	0.998912	8.49	96	1035	30312
cell wall	0.00	0.998912	8.49	96	1035	5618
peptidoglycan-based cell wall	0.00	0.998912	8.49	96	1035	9274
aromatic compound biosynthetic process	0.00	0.998915	5.77	15	245	19438
cellular polysaccharide biosynthetic process	0.00	0.998996	3.09	3	94	33692
cellular carbohydrate biosynthetic process	0.00	0.998996	3.09	3	94	34637
cellular polysaccharide metabolic process	0.00	0.998996	3.09	3	94	44264
cellular component organization	0.00	0.999093	1.67	1	59	16043
cellular component organization or biogenesis	0.00	0.999093	1.67	1	59	71840
drug metabolic process	0.00	0.999147	4.62	8	165	17144
small molecule biosynthetic process	0.00	0.999234	6.13	20	306	44283
generation of precursor metabolites and energy	0.00	0.999324	6.01	19	297	6091
organic cyclic compound biosynthetic process	0.00	0.999328	5.92	18	286	1901362
heterocycle biosynthetic process	0.00	0.999418	5.78	17	277	18130
nucleic acid metabolic process	0.00	0.999481	5.84	18	290	90304
carbohydrate derivative metabolic process	0.00	0.999644	5.83	19	307	1901135
membrane	0.00	0.999669	8.04	81	927	16020

nucleobase-containing compound metabolic process	0.00	0.999688	6.92	38	511	6139
cellular protein metabolic process	0.00	0.999704	4.50	9	191	44267
RNA metabolic process	0.00	0.999777	3.10	4	125	16070
anaerobic respiration	0.00	0.999812	4.72	11	222	9061
translation	0.00	0.999847	2.06	2	95	6412
RNA binding	0.00	0.9999	4.02	8	191	3723
nucleotide binding	0.00	0.999921	7.29	58	738	166
energy derivation by oxidation of organic compounds	0.00	0.999939	4.98	15	286	15980
organic substance metabolic process	0.00	0.999942	8.74	170	1775	71704
peptide biosynthetic process	0.00	0.999959	1.83	2	107	43043
peptide metabolic process	0.00	0.999959	1.83	2	107	6518
outer membrane	0.00	0.999981	1.72	2	114	19867
cell outer membrane	0.00	0.999981	1.72	2	114	9279
cytoplasm	0.00	0.999985	7.78	96	1138	5737
cellular respiration	0.00	0.999986	4.15	11	254	45333
cellular aromatic compound metabolic process	0.00	0.999988	6.40	41	600	6725
external encapsulating structure part	0.00	0.99999	1.64	2	120	44462
carbohydrate derivative biosynthetic process	0.00	0.999992	4.04	11	261	1901137
cellular macromolecule metabolic process	0.00	0.999995	5.94	34	538	44260
organic cyclic compound metabolic process	0.00	0.999996	6.32	44	652	1901360
heterocycle metabolic process	0.00	0.999997	6.27	43	643	46483
macromolecule biosynthetic process	0.00	0.999997	4.64	17	349	9059

protein folding	0.00	0.999997	1.95	3	151	6457
cellular nitrogen compound biosynthetic process	0.00	0.999999	4.44	17	366	44271
organonitrogen compound metabolic process	0.00	1	5.57	53	899	1901564
organonitrogen compound biosynthetic process	0.00	1	5.44	35	608	1901566
organic substance biosynthetic process	0.00	1	5.90	55	877	1901576
cellular nitrogen compound metabolic process	0.00	1	5.71	46	760	34641
cellular metabolic process	0.00	1	6.78	127	1745	44237
cellular biosynthetic process	0.00	1	5.74	50	821	44249
nitrogen compound metabolic process	0.00	1	6.16	81	1234	6807
biological_process	0.00	1	8.67	257	2706	8150
metabolic process	0.00	1	8.33	194	2135	8152
biosynthetic process	0.00	1	5.90	55	877	9058
cellular process	0.00	1	6.46	130	1882	9987
intracellular part	0.00	1	8.22	175	1954	44424
cell part	0.00	1	8.21	216	2414	44464
cellular_component	0.00	1	8.19	216	2421	5575
macromolecule metabolic process	0.00	1	5.59	42	710	43170
cellular macromolecule biosynthetic process	0.00	1	4.01	14	335	34645
cellular amide metabolic process	0.00	1	1.20	2	164	43603
amide biosynthetic process	0.00	1	1.29	2	153	43604

Table S1b. Gene ontology of 154 DEGs.

function	*Enrichment Score*	*Enrichment p-value*	*% genes in group that are present*	*# genes in list, in group*	*# genes not in list, in group*	*GO ID*
intracellular part	16.42	7.41E-08	3.66	78	2051	44424
aerobic respiration	15.90	1.24E-07	15.19	12	67	9060
organelle inner membrane	15.07	2.85E-07	4.82	43	849	19866
organelle membrane	15.07	2.85E-07	4.82	43	849	31090
organelle part	14.60	4.56E-07	4.74	43	864	44422
glyoxylate cycle	14.37	5.77E-07	55.56	5	4	6097
monovalent inorganic cation transport	13.17	1.90E-06	16.98	9	44	15672
proton transmembrane transport	13.17	1.90E-06	16.98	9	44	1902600
cation transmembrane transport	12.56	3.50E-06	12.36	11	78	98655
inorganic cation transmembrane transport	12.24	4.81E-06	15.25	9	50	98662
membrane	11.74	7.95E-06	4.27	43	965	16020
oxidoreduction-driven active transmembrane transporter activity	11.11	1.49E-05	50.00	4	4	15453
ion transmembrane transport	11.08	1.54E-05	9.03	13	131	34220
glyoxylate metabolic process	10.31	3.34E-05	27.78	5	13	46487
inorganic ion transmembrane transport	10.14	3.96E-05	11.84	9	67	98660
cell part	10.11	4.07E-05	3.12	82	2548	44464
cellular_component	10.00	4.55E-05	3.11	82	2555	5575
aspartate metabolic process	9.61	6.69E-05	36.36	4	7	6531
aspartate biosynthetic process	9.61	6.69E-05	36.36	4	7	6532
cation transport	9.17	0.000104019	8.66	11	116	6812
transmembrane transport	8.86	0.000141523	7.30	13	165	55085
ion transport	8.64	0.000176903	7.14	13	169	6811

glutamate metabolic process	8.26	0.000258198	26.67	4	11	6536
glutamate biosynthetic process	8.26	0.000258198	26.67	4	11	6537
external encapsulating structure	8.16	0.000286201	3.71	42	1089	30312
cell wall	8.16	0.000286201	3.71	42	1089	5618
peptidoglycan-based cell wall	8.16	0.000286201	3.71	42	1089	9274
carboxylic acid metabolic process	7.69	0.00045608	4.67	23	470	19752
oxoacid metabolic process	7.69	0.00045608	4.67	23	470	43436
organic acid metabolic process	7.33	0.00065772	4.55	23	483	6082
glutamine family amino acid metabolic process	7.00	0.000907713	9.86	7	64	9064
glutamine family amino acid biosynthetic process	7.00	0.000907713	9.86	7	64	9084
dicarboxylic acid metabolic process	6.84	0.00107202	9.59	7	66	43648
aspartate family amino acid metabolic process	6.84	0.00107202	9.59	7	66	9066
aspartate family amino acid biosynthetic process	6.84	0.00107202	9.59	7	66	9067
lactate dehydrogenase activity	6.55	0.00143329	66.67	2	1	4457
malate synthase activity	6.55	0.00143329	66.67	2	1	4474
lactate transport	6.55	0.00143329	66.67	2	1	15727
bacterial-type RNA polymerase core enzyme binding	6.55	0.00143329	66.67	2	1	1000
integral component of external side of plasma membrane	6.53	0.00145973	17.39	4	19	71575
amino acid transmembrane transport	6.45	0.00158594	8.97	7	71	3333
transporter activity	6.37	0.00170971	5.02	16	303	5215
cellular respiration	6.18	0.00206558	5.28	14	251	45333
localization	5.96	0.00257462	5.37	13	229	51179

establishment of localization	5.96	0.00257462	5.37	13	229	51234
transport	5.96	0.00257462	5.37	13	229	6810
cellular aldehyde metabolic process	5.93	0.00266739	11.36	5	39	6081
small molecule metabolic process	5.88	0.00280837	3.70	30	781	44281
extrinsic component of periplasmic side of plasma membrane	5.87	0.00282499	50.00	2	2	31236
D-alanine catabolic process	5.87	0.00282499	50.00	2	2	55130
succinate transmembrane transporter activity	5.87	0.00282499	50.00	2	2	15141
fumarate transport	5.87	0.00282499	50.00	2	2	15741
negative regulation of fatty acid biosynthetic process	5.87	0.00282499	50.00	2	2	45717
alpha-amino acid metabolic process	5.82	0.0029607	5.53	12	205	1901605
alpha-amino acid biosynthetic process	5.82	0.0029607	5.53	12	205	1901607
oxidation-reduction process	5.79	0.00307305	4.73	16	322	55114
cellular amino acid biosynthetic process	5.78	0.00307459	5.50	12	206	8652
transcription, DNA-templated	5.67	0.00344864	3.95	24	584	6351
carbohydrate-transporting ATPase activity	5.37	0.00464014	40.00	2	3	43211
energy derivation by oxidation of organic compounds	5.04	0.00648035	4.65	14	287	15980
glyceraldehyde-3-phosphate dehydrogenase (NAD+) (phosphorylating) activity	4.98	0.00685959	33.33	2	4	4365
aromatic amino acid transmembrane transporter activity	4.98	0.00685959	33.33	2	4	15173
succinate transmembrane transport	4.98	0.00685959	33.33	2	4	71422

capsule polysaccharide biosynthetic process	4.98	0.00685959	33.33	2	4	45227
phosphotransferase activity, carboxyl group as acceptor	4.98	0.00685959	33.33	2	4	16774
metabolic process	4.87	0.00770434	2.83	66	2263	8152
oxidation-reduction process	4.86	0.00776925	3.49	29	803	55114
regulation of transcription, DNAtemplated	4.84	0.00794217	3.69	24	626	6355
cell redox homeostasis	4.76	0.00857368	10.81	4	33	45454
tRNA wobble position uridine thiolation	4.66	0.00946484	28.57	2	5	2143
sister chromatid cohesion	4.66	0.00946484	28.57	2	5	7062
transcription initiation from bacterialtype RNA polymerase promoter	4.66	0.00946484	28.57	2	5	1123
generation of precursor metabolites and energy	4.63	0.00978063	4.43	14	302	6091
primary metabolic process	4.60	0.0100858	2.98	50	1629	44238
Mo-molybdopterin cofactor biosynthetic process	4.56	0.0104944	14.29	3	18	6777
membrane	4.39	0.0123559	2.78	66	2308	16020
proton-transporting ATP synthase complex, coupling factor F(o)	4.39	0.012438	25.00	2	6	45263
sodium:dicarboxylate symporter activity	4.39	0.012438	25.00	2	6	17153
glucose metabolic process	4.30	0.0135325	13.04	3	20	6006
monocarboxylic acid metabolic process	4.18	0.0152691	5.93	7	111	32787
DNA-binding transcription factor activity	4.17	0.0155052	4.08	15	353	3700

sulfur amino acid transport	4.15	0.0157617	22.22	2	7	101
cysteine transmembrane transport	4.15	0.0157617	22.22	2	7	1903712
cysteine transport	4.15	0.0157617	22.22	2	7	42883
dicarboxylic acid biosynthetic process	4.08	0.016892	8.89	4	41	43650
sodium ion transmembrane transport	3.94	0.0194194	20.00	2	8	35725
catalytic activity, acting on a protein	3.94	0.0194194	20.00	2	8	140096
proteolysis	3.94	0.0194194	20.00	2	8	6508
peptidase activity	3.94	0.0194194	20.00	2	8	8233
diaminopimelate metabolic process	3.77	0.0231081	10.71	3	25	46451
lysine metabolic process	3.77	0.0231081	10.71	3	25	6553
lysine biosynthetic process	3.77	0.0231081	10.71	3	25	9085
lysine biosynthetic process via diaminopimelate	3.77	0.0231081	10.71	3	25	9089
organic acid biosynthetic process	3.76	0.0233791	4.21	12	273	16053
carboxylic acid biosynthetic process	3.76	0.0233791	4.21	12	273	46394
succinate dehydrogenase activity	3.76	0.0233947	18.18	2	9	104
chromosome condensation	3.76	0.0233947	18.18	2	9	30261
tricarboxylic acid cycle	3.58	0.02774	10.00	3	27	6099
citrate metabolic process	3.58	0.02774	10.00	3	27	6101
tricarboxylic acid metabolic process	3.58	0.02774	10.00	3	27	72350
monosaccharide-transporting ATPase activity	3.43	0.0322366	15.38	2	11	15407
galactose transmembrane transport	3.43	0.0322366	15.38	2	11	15757
fucose metabolic process	3.43	0.0322366	15.38	2	11	6004
biological_process	3.43	0.0322777	2.60	77	2886	8150
integral component of membrane	3.42	0.0328334	2.77	52	1823	16021

oxidoreductase activity, acting on CHOH group of donors	3.34	0.0355429	9.09	3	30	16614
cellular process	3.33	0.0357528	2.73	55	1957	9987
cellular amino acid metabolic process	3.31	0.0363386	3.83	13	326	6520
oxidoreductase activity, acting on the CH-CH group of donors	3.29	0.0370734	14.29	2	12	16627
amino acid transmembrane transport	3.25	0.0386671	6.90	4	54	3333
amino acid transport	3.25	0.0386671	6.90	4	54	6865
2 iron, 2 sulfur cluster binding	3.25	0.0386671	6.90	4	54	51537
tryptophan biosynthetic process	3.17	0.0421685	13.33	2	13	162
indole-containing compound metabolic process	3.17	0.0421685	13.33	2	13	42430
indole-containing compound biosynthetic process	3.17	0.0421685	13.33	2	13	42435
indolalkylamine biosynthetic process	3.17	0.0421685	13.33	2	13	46219
tryptophan metabolic process	3.17	0.0421685	13.33	2	13	6568
indolalkylamine metabolic process	3.17	0.0421685	13.33	2	13	6586
ribosomal large subunit binding	3.17	0.0421685	13.33	2	13	43023
neutral amino acid transport	3.17	0.0421685	13.33	2	13	15804
xenobiotic metabolic process	3.15	0.0429829	6.67	4	56	6805
phosphorelay signal transduction system	3.13	0.0437218	4.76	7	140	160
antibiotic metabolic process	3.12	0.0443554	8.33	3	33	16999
organic anion transport	3.10	0.0452388	6.56	4	57	15711
carboxylic acid transmembrane transport	3.10	0.0452388	6.56	4	57	1905039
carboxylic acid transport	3.10	0.0452388	6.56	4	57	46942
cellular metabolic process	3.06	0.0469679	2.72	51	1821	44237

organic acid transport	3.00	0.049946	6.35	4	59	15849
organic acid transmembrane transport	3.00	0.049946	6.35	4	59	1903825
glutathione transferase activity	2.94	0.0530798	11.76	2	15	4364
protein-DNA complex	2.94	0.0530798	11.76	2	15	32993
small molecule biosynthetic process	2.88	0.0561546	3.68	12	314	44283
NADP binding	2.85	0.0576253	7.50	3	37	50661
catalytic activity	2.77	0.0628464	5.88	4	64	3824
cytoplasm	2.76	0.0633495	2.84	35	1199	5737
phosphopyruvate hydratase complex	2.74	0.0648321	33.33	1	2	15
phosphopyruvate hydratase activity	2.74	0.0648321	33.33	1	2	4634
CTP biosynthetic process	2.74	0.0648321	33.33	1	2	6241
glycine biosynthetic process	2.74	0.0648321	33.33	1	2	6545
'de novo' CTP biosynthetic process	2.74	0.0648321	33.33	1	2	44210
acetate transmembrane transport	2.74	0.0648321	33.33	1	2	35433
cell envelope Sec protein transport complex	2.74	0.0648321	33.33	1	2	31522
DNA-templated transcription, initiation	2.74	0.064867	10.53	2	17	6352
intrinsic component of plasma membrane	2.74	0.064867	10.53	2	17	31226
aromatic amino acid family metabolic process	2.68	0.068667	6.98	3	40	9072
aromatic amino acid family biosynthetic process	2.68	0.068667	6.98	3	40	9073
energy coupled proton transport, down electrochemical gradient	2.56	0.077434	9.52	2	19	15985
ATP synthesis coupled proton transport	2.56	0.077434	9.52	2	19	15986
nucleotide-sugar metabolic process	2.48	0.0839816	9.09	2	20	9225
nucleotide-sugar biosynthetic process	2.48	0.0839816	9.09	2	20	9226

peptidoglycan metabolic process	2.48	0.0839816	9.09	2	20	270
organic substance metabolic process	2.46	0.0851263	2.62	51	1894	71704
dihydrofolate reductase activity	2.46	0.0855011	25.00	1	3	4146
P-P-bond-hydrolysis-driven protein transmembrane transporter activity	2.46	0.0855011	25.00	1	3	15450
tyrosine biosynthetic process	2.46	0.0855011	25.00	1	3	6571
malate dehydrogenase (decarboxylating) (NAD+) activity	2.46	0.0855011	25.00	1	3	4471
DNA binding	2.42	0.0889465	2.87	27	914	3677
protein transport	2.41	0.0902099	4.63	5	103	15031
hydrolase activity	2.40	0.090691	8.70	2	21	16787
anion transport	2.34	0.0967188	5.06	4	75	6820
anion transmembrane transport	2.34	0.0967188	5.06	4	75	98656
flavin adenine dinucleotide binding	2.32	0.0985681	4.50	5	106	50660
nitrogen compound transport	2.25	0.105716	20.00	1	4	71705
regulation of potassium ion transport	2.25	0.105716	20.00	1	4	43266
cytochrome complex assembly	2.24	0.106873	5.77	3	49	17004
cellular protein-containing complex assembly	2.24	0.106873	5.77	3	49	34622
protein-containing complex subunit organization	2.24	0.106873	5.77	3	49	43933
protein-containing complex assembly	2.24	0.106873	5.77	3	49	65003
amino acid transmembrane transporter activity	2.19	0.111545	5.66	3	50	15171
protein disulfide oxidoreductase activity	2.19	0.11169	7.69	2	24	15035
transcription regulatory region DNA binding	2.19	0.11169	7.69	2	24	44212

cellular component assembly	2.03	0.13098	5.26	3	54	22607
sulfur compound transport	2.01	0.133798	6.90	2	27	72348
copper ion binding	2.01	0.133798	6.90	2	27	5507
carbohydrate metabolic process	1.96	0.140283	2.93	17	564	5975
serine-type endopeptidase activity	1.96	0.141374	6.67	2	28	4252
pyridoxal phosphate binding	1.96	0.141458	4.40	4	87	30170
alanine metabolic process	1.93	0.144825	14.29	1	6	6522
alanine biosynthetic process	1.93	0.144825	14.29	1	6	6523
pyruvate family amino acid metabolic process	1.93	0.144825	14.29	1	6	9078
pyruvate family amino acid biosynthetic process	1.93	0.144825	14.29	1	6	9079
molybdopterin cofactor biosynthetic process	1.93	0.144825	14.29	1	6	32324
tyrosine metabolic process	1.93	0.144825	14.29	1	6	6570
tyrosine biosynthetic process	1.93	0.144825	14.29	1	6	6571
cellular component organization	1.92	0.146282	5.00	3	57	16043
cellular component organization or biogenesis	1.92	0.146282	5.00	3	57	71840
cytoplasm	1.91	0.147653	2.62	35	1299	5737
cell	1.90	0.149039	6.45	2	29	5623
endopeptidase activity	1.90	0.149039	6.45	2	29	4175
arginine metabolic process	1.90	0.149039	6.45	2	29	6525
arginine biosynthetic process	1.90	0.149039	6.45	2	29	6526
sequence-specific DNA binding	1.83	0.160706	3.61	6	160	43565
L-fucose catabolic process	1.81	0.163737	12.50	1	7	42355

erythrose 4-phosphate/phosphoenolpyruvate family amino acid metabolic process	1.81	0.163737	12.50	1	7	1902221
erythrose 4-phosphate/phosphoenolpyruvate family amino acid biosynthetic process	1.81	0.163737	12.50	1	7	1902223
L-phenylalanine metabolic process	1.81	0.163737	12.50	1	7	6558
L-phenylalanine biosynthetic process	1.81	0.163737	12.50	1	7	9094
establishment of integrated proviral latency	1.81	0.163737	12.50	1	7	75713
cellular carbohydrate metabolic process	1.73	0.178075	3.70	5	130	44262
oxidoreductase activity, acting on the aldehyde or oxo group of donors, NAD or NADP as acceptor	1.71	0.180449	5.71	2	33	16620
protein targeting	1.70	0.182234	11.11	1	8	6605
carbohydrate catabolic process	1.70	0.183334	2.96	12	393	16052
drug metabolic process	1.70	0.183337	3.47	6	167	17144
protein-containing complex	1.67	0.188455	5.56	2	34	32991
fatty acid biosynthetic process	1.67	0.188455	5.56	2	34	6633
molybdenum ion binding	1.63	0.196509	5.41	2	35	30151
phosphorelay response regulator activity	1.63	0.196509	5.41	2	35	156
fatty acid oxidation	1.63	0.196509	5.41	2	35	19395
lipid modification	1.63	0.196509	5.41	2	35	30258
lipid oxidation	1.63	0.196509	5.41	2	35	34440
protein serine/threonine kinase activity	1.61	0.200325	10.00	1	9	4674

aromatic amino acid family biosynthetic process, prephenate pathway	1.61	0.200325	10.00	1	9	9095
nitrogen compound transport	1.58	0.206521	3.77	4	102	71705
negative regulation of DNA-templated transcription, termination	1.52	0.218018	9.09	1	10	60567
NAD+ binding	1.52	0.218018	9.09	1	10	70403
fatty acid metabolic process	1.51	0.220902	5.00	2	38	6631
iron-sulfur cluster binding	1.49	0.225231	3.04	8	255	51536
intracellular protein transmembrane transport	1.45	0.235322	8.33	1	11	65002
glutamine metabolic process	1.45	0.235322	8.33	1	11	6541
glutamine biosynthetic process	1.45	0.235322	8.33	1	11	6542
iron ion homeostasis	1.44	0.237301	4.76	2	40	55072
cellular biogenic amine biosynthetic process	1.44	0.237301	4.76	2	40	42401
amine biosynthetic process	1.44	0.237301	4.76	2	40	9309
electron transfer activity	1.44	0.237301	4.76	2	40	9055
dephosphorylation	1.42	0.241661	3.90	3	74	16311
oxidoreductase activity	1.40	0.245528	4.65	2	41	16491
molecular_function	1.39	0.249413	3.27	5	148	3674
organonitrogen compound biosynthetic process	1.38	0.251769	2.64	17	626	1901566
transferase activity, transferring alkyl or aryl (other than methyl) groups	1.38	0.252246	7.69	1	12	16765
cellular amine metabolic process	1.37	0.253765	4.55	2	42	44106
cellular biogenic amine metabolic process	1.37	0.253765	4.55	2	42	6576
ATP metabolic process	1.34	0.262009	4.44	2	43	46034

ATP biosynthetic process	1.34	0.262009	4.44	2	43	6754
nucleoside monophosphate metabolic process	1.34	0.262009	4.44	2	43	9123
nucleoside monophosphate biosynthetic process	1.34	0.262009	4.44	2	43	9124
purine nucleoside monophosphate metabolic process	1.34	0.262009	4.44	2	43	9126
purine nucleoside monophosphate biosynthetic process	1.34	0.262009	4.44	2	43	9127
nucleoside triphosphate metabolic process	1.34	0.262009	4.44	2	43	9141
nucleoside triphosphate biosynthetic process	1.34	0.262009	4.44	2	43	9142
purine nucleoside triphosphate metabolic process	1.34	0.262009	4.44	2	43	9144
purine nucleoside triphosphate biosynthetic process	1.34	0.262009	4.44	2	43	9145
ribonucleoside monophosphate biosynthetic process	1.34	0.262009	4.44	2	43	9156
ribonucleoside monophosphate metabolic process	1.34	0.262009	4.44	2	43	9161
purine ribonucleoside monophosphate metabolic process	1.34	0.262009	4.44	2	43	9167
purine ribonucleoside monophosphate biosynthetic process	1.34	0.262009	4.44	2	43	9168
ribonucleoside triphosphate metabolic process	1.34	0.262009	4.44	2	43	9199
ribonucleoside triphosphate biosynthetic process	1.34	0.262009	4.44	2	43	9201

purine ribonucleoside triphosphate metabolic process	1.34	0.262009	4.44	2	43	9205
purine ribonucleoside triphosphate biosynthetic process	1.34	0.262009	4.44	2	43	9206
response to osmotic stress	1.31	0.270255	4.35	2	44	6970
L-alpha-amino acid transmembrane transport	1.28	0.278499	4.26	2	45	1902475
cellular biosynthetic process	1.27	0.281567	2.53	22	849	44249
monosaccharide transmembrane transporter activity	1.26	0.284985	6.67	1	14	15145
L-amino acid transport	1.25	0.286738	4.17	2	46	15807
fatty acid metabolic process	1.25	0.286738	4.17	2	46	6631
nucleobase-containing small molecule metabolic process	1.21	0.297412	2.86	7	238	55086
transcription antitermination	1.20	0.300817	6.25	1	15	31564
extrinsic component of cytoplasmic side of plasma membrane	1.15	0.3163	5.88	1	16	31234
organic substance transport	1.13	0.323433	3.08	4	126	71702
nucleobase-containing small molecule interconversion	1.12	0.326124	3.30	3	88	15949
pyrimidine nucleotide biosynthetic process	1.10	0.331443	5.56	1	17	6221
manganese ion binding	1.08	0.338299	3.23	3	90	30145
aromatic compound biosynthetic process	1.05	0.351249	2.69	7	253	19438
phosphoprotein phosphatase activity	1.02	0.360737	5.00	1	19	4721
translation	1.01	0.362605	3.09	3	94	6412
cellular lipid metabolic process	1.01	0.362605	3.09	3	94	44255

lipid metabolic process	1.01	0.362605	3.09	3	94	6629
ubiquinone biosynthetic process	0.94	0.388756	4.55	1	21	6744
intracellular	0.93	0.39461	2.62	6	223	5622
glutamine metabolic process	0.91	0.402304	4.35	1	22	6541
cytochrome complex assembly	0.91	0.402304	4.35	1	22	17004
organic substance biosynthetic process	0.91	0.403265	2.36	22	910	1901576
biosynthetic process	0.91	0.403265	2.36	22	910	9058
protein dephosphorylation	0.88	0.415554	4.17	1	23	6470
peptide biosynthetic process	0.83	0.434413	2.75	3	106	43043
peptide metabolic process	0.83	0.434413	2.75	3	106	6518
protein folding	0.81	0.444076	2.60	4	150	6457
organonitrogen compound metabolic process	0.81	0.444978	2.31	22	930	1901564
ribose phosphate biosynthetic process	0.79	0.453113	2.90	2	67	46390
purine ribonucleotide metabolic process	0.79	0.453113	2.90	2	67	9150
purine ribonucleotide biosynthetic process	0.79	0.453113	2.90	2	67	9152
ribonucleotide metabolic process	0.79	0.453113	2.90	2	67	9259
ribonucleotide biosynthetic process	0.79	0.453113	2.90	2	67	9260
cytosolic small ribosomal subunit	0.76	0.4657	3.57	1	27	22627
cellular nitrogen compound biosynthetic process	0.75	0.474069	2.35	9	374	44271
DNA replication	0.70	0.496721	2.67	2	73	6260
DNA-dependent DNA replication	0.70	0.496721	2.67	2	73	6261
nucleobase-containing compound biosynthetic process	0.70	0.497534	2.42	4	161	34654
protein metabolic process	0.69	0.500892	2.34	6	250	19538

organic cyclic compound biosynthetic process	0.67	0.51094	2.30	7	297	1901362
iron-sulfur cluster assembly	0.67	0.51157	3.13	1	31	16226
protein binding	0.66	0.516091	2.23	25	1097	5515
periplasmic space	0.64	0.525781	2.34	4	167	42597
nitrogen compound metabolic process	0.62	0.537951	2.21	29	1286	6807
ribose phosphate metabolic process	0.61	0.544739	2.44	2	80	19693
organic substance catabolic process	0.61	0.545219	2.20	14	621	1901575
catabolic process	0.61	0.545219	2.20	14	621	9056
extracellular region	0.59	0.553526	2.78	1	35	5576
amine metabolic process	0.54	0.583312	2.27	2	86	9308
oxidoreductase activity, acting on the CH-OH group of donors, NAD or NADP as acceptor	0.49	0.609841	2.38	1	41	16616
purine nucleotide metabolic process	0.47	0.625231	2.11	2	93	6163
purine nucleotide biosynthetic process	0.47	0.625231	2.11	2	93	6164
purine-containing compound biosynthetic process	0.47	0.625231	2.11	2	93	72522
kinase activity	0.46	0.630934	2.05	6	286	16301
purine-containing compound metabolic process	0.46	0.630948	2.08	2	94	72521
phosphorylation	0.45	0.637577	2.04	6	288	16310
heterocycle biosynthetic process	0.45	0.637577	2.04	6	288	18130
cellular protein metabolic process	0.43	0.650228	2.00	4	196	44267
cellular amino acid metabolic process	0.41	0.666681	2.04	1	48	6520
amide biosynthetic process	0.40	0.671138	1.94	3	152	43604
phospholipid metabolic process	0.39	0.674099	2.00	1	49	6644
phospholipid biosynthetic process	0.39	0.674099	2.00	1	49	8654

cellular amino acid catabolic process	0.39	0.674268	1.92	2	102	9063
transferase activity	0.36	0.695488	2.03	20	965	16740
nucleotide binding	0.36	0.696039	2.01	16	780	166
cellular amide metabolic process	0.33	0.715777	1.81	3	163	43603
carboxylic acid catabolic process	0.32	0.722552	1.75	2	112	46395
heme binding	0.31	0.7339	1.69	1	58	20037
single-species biofilm formation	0.30	0.739831	1.67	1	59	44010
structural constituent of ribosome	0.30	0.739831	1.67	1	59	3735
structural molecule activity	0.30	0.739831	1.67	1	59	5198
response to abiotic stimulus	0.30	0.744364	1.68	2	117	9628
lipid biosynthetic process	0.29	0.745631	1.64	1	60	8610
structural constituent of ribosome	0.29	0.751303	1.61	1	61	3735
organic acid catabolic process	0.28	0.756739	1.64	2	120	16054
small molecule catabolic process	0.27	0.760748	1.63	2	121	44282
cell wall organization	0.26	0.768597	1.60	2	123	71555
cellular aromatic compound metabolic process	0.26	0.769214	1.87	12	629	6725
DNA integration	0.23	0.797013	1.41	1	70	15074
peptidoglycan metabolic process	0.22	0.805978	1.37	1	72	270
glycosaminoglycan metabolic process	0.22	0.805978	1.37	1	72	30203
aminoglycan metabolic process	0.22	0.805978	1.37	1	72	6022
nucleoside phosphate biosynthetic process	0.18	0.832654	1.39	2	142	1901293
nucleotide biosynthetic process	0.18	0.832654	1.39	2	142	9165
nucleoside phosphate metabolic process	0.17	0.841177	1.36	2	145	6753
nucleotide metabolic process	0.17	0.841177	1.36	2	145	9117
organophosphate biosynthetic process	0.16	0.848887	1.43	3	207	90407

carbohydrate derivative metabolic process	0.16	0.854239	1.53	5	321	1901135
organic cyclic compound metabolic process	0.16	0.856034	1.72	12	684	1901360
carbohydrate derivative biosynthetic process	0.15	0.858061	1.47	4	268	1901137
cellular nitrogen compound metabolic process	0.14	0.866427	1.74	14	792	34641
nucleobase-containing compound metabolic process	0.14	0.867023	1.64	9	540	6139
GTP binding	0.13	0.873763	1.09	1	91	5525
transferase activity, transferring glycosyl groups	0.12	0.889814	1.02	1	97	16757
cellular macromolecule biosynthetic process	0.11	0.891683	1.43	5	344	34645
ATP binding	0.11	0.89357	1.65	12	716	5524
anaerobic respiration	0.11	0.894146	1.29	3	230	9061
nucleic acid binding	0.11	0.894539	1.18	2	168	3676
organophosphate metabolic process	0.10	0.900655	1.27	3	234	19637
phosphate-containing compound metabolic process	0.10	0.900655	1.27	3	234	6796
protein modification process	0.10	0.901629	0.97	1	102	36211
cellular protein modification process	0.10	0.901629	0.97	1	102	6464
heterocycle metabolic process	0.10	0.903997	1.60	11	675	46483
organonitrogen compound catabolic process	0.10	0.908837	1.12	2	176	1901565
transferase activity, transferring acyl groups	0.09	0.910167	0.93	1	106	16746
macromolecule biosynthetic process	0.09	0.913797	1.37	5	361	9059

DNA metabolic process	0.09	0.915291	1.10	2	180	6259
response to stress	0.08	0.919848	1.08	2	183	6950
DNA recombination	0.08	0.921614	0.88	1	112	6310
iron ion binding	0.08	0.925098	0.87	1	114	5506
translation	0.06	0.945536	0.78	1	128	6412
phosphorus metabolic process	0.05	0.955035	1.05	3	282	6793
cellular catabolic process	0.04	0.961607	0.89	2	222	44248
response to antibiotic	0.04	0.96388	0.68	1	146	46677
macromolecule modification	0.02	0.983801	0.55	1	181	43412
RNA binding	0.01	0.989044	0.50	1	198	3723
macromolecule metabolic process	0.01	0.989059	1.20	9	743	43170
cellular macromolecule metabolic process	0.01	0.989791	1.05	6	566	44260
nucleic acid metabolic process	0.01	0.992772	0.65	2	306	90304
response to stimulus	0.00	0.996023	0.59	2	335	50896

Table S1c. Gene ontology of 349 DEGs.

function	***Enrichment Score***	***Enrichment p-value***	***% genes in group that are present***	***# genes in list, in group***	***# genes not in list, in group***	***GO ID***
carboxylic acid catabolic process	25.47	8.70E-12	22.81	26	88	46395
organic acid catabolic process	23.83	4.48E-11	21.31	26	96	16054
small molecule catabolic process	23.63	5.45E-11	21.14	26	97	44282
organic substance catabolic process	23.24	8.05E-11	10.55	67	568	1901575
catabolic process	23.24	8.05E-11	10.55	67	568	9056
cellular amino acid catabolic process	20.18	1.72E-09	21.15	22	82	9063

carboxylic acid metabolic process	18.95	5.89E-09	10.75	53	440	19752
oxoacid metabolic process	18.95	5.89E-09	10.75	53	440	43436
organic acid metabolic process	18.04	1.47E-08	10.47	53	453	6082
arginine metabolic process	16.61	6.13E-08	47.37	9	10	6525
carbohydrate catabolic process	16.09	1.03E-07	10.86	44	361	16052
arginine catabolic process	15.83	1.34E-07	63.64	7	4	6527
periplasmic space	14.93	3.29E-07	14.62	25	146	42597
cellular catabolic process	13.28	1.70E-06	12.50	28	196	44248
negative regulation of cellular macromolecule biosynthetic process	12.45	3.91E-06	54.55	6	5	2000113
negative regulation of nucleobasecontaining compound metabolic process	12.45	3.91E-06	54.55	6	5	45934
fatty acid beta-oxidation	12.35	4.34E-06	71.43	5	2	6635
mannose transmembrane transport	12.25	4.81E-06	100.00	4	0	15761
negative regulation of macromolecule biosynthetic process	11.80	7.51E-06	50.00	6	6	10558
negative regulation of macromolecule metabolic process	11.80	7.51E-06	50.00	6	6	10605
negative regulation of cellular metabolic process	11.80	7.51E-06	50.00	6	6	31324
negative regulation of cellular biosynthetic process	11.80	7.51E-06	50.00	6	6	31327
negative regulation of nitrogen compound metabolic process	11.80	7.51E-06	50.00	6	6	51172
negative regulation of biosynthetic process	11.80	7.51E-06	50.00	6	6	9890
negative regulation of metabolic process	11.80	7.51E-06	50.00	6	6	9892

cellular amino acid metabolic process	11.80	7.54E-06	10.32	35	304	6520
organonitrogen compound catabolic process	11.67	8.55E-06	12.92	23	155	1901565
oxidoreductase activity, acting on the aldehyde or oxo group of donors, NAD or NADP as acceptor	10.64	2.40E-05	25.71	9	26	16620
small molecule metabolic process	10.49	2.77E-05	7.77	63	748	44281
antibiotic metabolic process	10.39	3.07E-05	25.00	9	27	16999
carbohydrate metabolic process	9.63	6.59E-05	8.26	48	533	5975
response to ethanol	9.61	6.68E-05	66.67	4	2	45471
amino acid transmembrane transporter activity	8.82	0.000147275	18.87	10	43	15171
primary metabolic process	8.61	0.000182182	6.37	107	1572	44238
oxidation-reduction process	8.49	0.000205874	7.33	61	771	55114
negative regulation of DNA-templated transcription, initiation	8.15	0.000289283	50.00	4	4	2000143
negative regulation of protein complex assembly	8.15	0.000289283	50.00	4	4	31333
negative regulation of transcription, DNA-templated	8.15	0.000289283	50.00	4	4	45892
oxidoreductase activity, acting on the CH-CH group of donors	8.07	0.000314242	35.71	5	9	16627
tricarboxylic acid cycle	7.87	0.000380137	23.33	7	23	6099
citrate metabolic process	7.87	0.000380137	23.33	7	23	6101
tricarboxylic acid metabolic process	7.87	0.000380137	23.33	7	23	72350
ferroxidase activity	7.83	0.000398119	75.00	3	1	4322
negative regulation of gene expression	7.60	0.00050147	44.44	4	5	10629

negative regulation of RNA biosynthetic process	7.60	0.00050147	44.44	4	5	1902679
negative regulation of nucleic acidtemplated transcription	7.60	0.00050147	44.44	4	5	1903507
negative regulation of RNA metabolic process	7.60	0.00050147	44.44	4	5	51253
cellular ketone metabolic process	7.60	0.00050147	44.44	4	5	42180
methylglyoxal metabolic process	7.60	0.00050147	44.44	4	5	9438
cellular response to hydrogen peroxide	6.77	0.001151	27.78	5	13	70301
monocarboxylic acid metabolic process	6.72	0.00120355	11.86	14	104	32787
biological_process	6.72	0.00120836	5.60	166	2797	8150
fumarate hydratase activity	6.29	0.00185432	50.00	3	3	4333
regulation of nucleobase-containing compound metabolic process	6.11	0.00221117	12.12	12	87	19219
regulation of cellular macromolecule biosynthetic process	6.03	0.00240945	12.00	12	88	2000112
response to stress	5.95	0.00259398	9.73	18	167	6950
organic substance metabolic process	5.83	0.0029398	5.86	114	1831	71704
amino acid transmembrane transport	5.70	0.00335654	12.82	10	68	3333
DNA-binding transcription factor activity	5.51	0.00404779	7.88	29	339	3700
metabolic process	5.47	0.00420143	5.67	132	2197	8152
regulation of macromolecule biosynthetic process	5.46	0.00424034	11.22	12	95	10556
regulation of metabolic process	5.46	0.00424034	11.22	12	95	19222
regulation of cellular metabolic process	5.46	0.00424034	11.22	12	95	31323
regulation of cellular biosynthetic process	5.46	0.00424034	11.22	12	95	31326

regulation of nitrogen compound metabolic process	5.46	0.00424034	11.22	12	95	51171
regulation of macromolecule metabolic process	5.46	0.00424034	11.22	12	95	60255
regulation of primary metabolic process	5.46	0.00424034	11.22	12	95	80090
regulation of biosynthetic process	5.46	0.00424034	11.22	12	95	9889
lipid catabolic process	5.44	0.00433964	26.67	4	11	16042
response to osmotic stress	5.25	0.00524279	15.22	7	39	6970
ribosomal small subunit binding	5.19	0.00557478	25.00	4	12	43024
negative regulation of cellular component organization	5.19	0.00557478	25.00	4	12	51129
fermentation	5.19	0.00557478	25.00	4	12	6113
negative regulation of cellular process	5.10	0.0060865	16.67	6	30	48523
mRNA 3'-UTR binding	5.05	0.00641162	66.67	2	1	3730
3-hydroxyoctanoyl-[acyl-carrier-protein] dehydratase activity	5.05	0.00641162	66.67	2	1	47451
fatty acid metabolic process	5.01	0.00665691	14.58	7	41	6631
drug metabolic process	4.94	0.00718229	9.25	16	157	17144
negative regulation of biological process	4.83	0.00797404	15.79	6	32	48519
monocarboxylic acid catabolic process	4.74	0.00870224	22.22	4	14	72329
regulation of transcription, DNAtemplated	4.68	0.00929633	11.11	10	80	6355
arginine biosynthetic process	4.54	0.0106221	21.05	4	15	6526
glutamine catabolic process	4.39	0.0124251	50.00	2	2	6543
D-alanine catabolic process	4.39	0.0124251	50.00	2	2	55130
glucuronate catabolic process	4.39	0.0124251	50.00	2	2	6064
acetate catabolic process	4.39	0.0124251	50.00	2	2	45733
glutathione metabolic process	4.39	0.0124251	50.00	2	2	6749
glutathione biosynthetic process	4.39	0.0124251	50.00	2	2	6750

cellular response to UV	4.39	0.0124251	50.00	2	2	34644
spermidine transmembrane transporter activity	4.39	0.0124251	50.00	2	2	15606
sodium ion transmembrane transporter activity	4.39	0.0124251	50.00	2	2	15081
conjugation	4.39	0.0124251	50.00	2	2	746
cellular response to cadmium ion	4.39	0.0124251	50.00	2	2	71276
oxidoreductase activity, acting on a sulfur group of donors, disulfide as acceptor	4.39	0.0124251	50.00	2	2	16671
ion transmembrane transporter activity	4.39	0.0124251	50.00	2	2	15075
fumarate metabolic process	4.39	0.0124251	50.00	2	2	6106
threonine catabolic process	4.36	0.012832	27.27	3	8	6567
response to acidic pH	4.18	0.015234	19.05	4	17	10447
regulation of nucleic acid-templated transcription	4.18	0.0153272	10.31	10	87	1903506
regulation of RNA biosynthetic process	4.18	0.0153272	10.31	10	87	2001141
regulation of RNA metabolic process	4.18	0.0153272	10.31	10	87	51252
cellular aldehyde metabolic process	4.13	0.0161192	13.64	6	38	6081
regulation of gene expression	4.11	0.0163829	10.20	10	88	10468
glutamine family amino acid metabolic process	4.04	0.0176729	11.27	8	63	9064
glutamine family amino acid biosynthetic process	4.04	0.0176729	11.27	8	63	9084
regulation of DNA-templated transcription, initiation	4.02	0.0179461	18.18	4	18	2000142
regulation of protein complex assembly	4.02	0.0179461	18.18	4	18	43254
localization	3.96	0.0191119	7.85	19	223	51179

establishment of localization	3.96	0.0191119	7.85	19	223	51234
transport	3.96	0.0191119	7.85	19	223	6810
biological regulation	3.95	0.0191691	8.47	15	162	65007

oxidoreductase activity, oxidizing metal ions	3.91	0.0200682	40.00	2	3	16722
peptide binding	3.91	0.0200682	40.00	2	3	42277
glucose catabolic process	3.91	0.0200682	40.00	2	3	6007
enoyl-CoA hydratase activity	3.91	0.0200682	40.00	2	3	4300
spermidine transport	3.91	0.0200682	40.00	2	3	15848
spermidine transmembrane transport	3.91	0.0200682	40.00	2	3	1903711
amino acid transmembrane transporter activity	3.91	0.0200682	40.00	2	3	15171
carboxylic acid transmembrane transporter activity	3.91	0.0200682	40.00	2	3	46943
organic acid transmembrane transporter activity	3.91	0.0200682	40.00	2	3	5342
organic anion transmembrane transporter activity	3.91	0.0200682	40.00	2	3	8514
succinate-semialdehyde dehydrogenase (NAD+) activity	3.91	0.0200682	40.00	2	3	4777
plasmid recombination	3.91	0.0200682	40.00	2	3	42150
regulation of isomerase activity	3.91	0.0200682	40.00	2	3	10911
regulation of DNA topoisomerase (ATPhydrolyzing) activity	3.91	0.0200682	40.00	2	3	2000371
negative regulation of DNA topoisomerase (ATP-hydrolyzing) activity	3.91	0.0200682	40.00	2	3	2000372
negative regulation of ATPase activity	3.91	0.0200682	40.00	2	3	32780

negative regulation of catalytic activity	3.91	0.0200682	40.00	2	3	43086
regulation of ATPase activity	3.91	0.0200682	40.00	2	3	43462
regulation of catalytic activity	3.91	0.0200682	40.00	2	3	50790
regulation of hydrolase activity	3.91	0.0200682	40.00	2	3	51336
negative regulation of hydrolase activity	3.91	0.0200682	40.00	2	3	51346
organic cation transport	3.88	0.0207445	23.08	3	10	15695
ammonium transport	3.88	0.0207445	23.08	3	10	15696
polyamine transport	3.88	0.0207445	23.08	3	10	15846
ion transmembrane transport	3.87	0.02094	17.39	4	19	34220
ion transport	3.73	0.0240087	8.24	15	167	6811
transition metal ion transport	3.67	0.0253757	13.89	5	31	41
iron ion transport	3.67	0.0253757	13.89	5	31	6826
fermentation	3.67	0.0254775	11.29	7	55	6113
alpha-amino acid metabolic process	3.64	0.0263285	7.83	17	200	1901605
alpha-amino acid biosynthetic process	3.64	0.0263285	7.83	17	200	1901607
cellular amino acid biosynthetic process	3.60	0.0273755	7.80	17	201	8652
response to nutrient levels	3.58	0.0277993	16.00	4	21	31667
response to starvation	3.58	0.0277993	16.00	4	21	42594
coenzyme binding	3.58	0.0277993	16.00	4	21	50662
peroxidase activity	3.58	0.0277993	16.00	4	21	4601
antibiotic catabolic process	3.53	0.0291751	33.33	2	4	17001
acetate catabolic process	3.53	0.0291751	33.33	2	4	45733
acetate metabolic process	3.53	0.0291751	33.33	2	4	6083
arginine biosynthetic process via ornithine	3.53	0.0291751	33.33	2	4	42450
threonine efflux transmembrane transporter activity	3.53	0.0291751	33.33	2	4	15565

tripeptide transmembrane transport	3.53	0.0291751	33.33	2	4	35443
5-phosphoribose 1-diphosphate biosynthetic process	3.53	0.0291751	33.33	2	4	6015
N,N'-diacetylchitobiose import	3.53	0.0291751	33.33	2	4	1902815
glyceraldehyde-3-phosphate dehydrogenase (NAD+) (phosphorylating) activity	3.53	0.0291751	33.33	2	4	4365
protein transport	3.50	0.0300927	9.26	10	98	15031
regulation of cellular process	3.50	0.0302616	8.39	13	142	50794
metal ion transport	3.27	0.0380729	12.50	5	35	30001
L-serine catabolic process	3.23	0.0395921	28.57	2	5	6565
alanine metabolic process	3.23	0.0395921	28.57	2	5	6522
alanine biosynthetic process	3.23	0.0395921	28.57	2	5	6523
pyruvate family amino acid metabolic process	3.23	0.0395921	28.57	2	5	9078
pyruvate family amino acid biosynthetic process	3.23	0.0395921	28.57	2	5	9079
ammonium transmembrane transport	3.23	0.0395921	28.57	2	5	72488
dormancy process	3.23	0.0395921	28.57	2	5	22611
sodium ion binding	3.23	0.0395921	28.57	2	5	31402
anion transmembrane transporter activity	3.23	0.0395921	28.57	2	5	8509
primary alcohol metabolic process	3.23	0.0395921	28.57	2	5	34308
glycolate metabolic process	3.23	0.0395921	28.57	2	5	9441
negative regulation of molecular function	3.23	0.0395921	28.57	2	5	44092
regulation of molecular function	3.23	0.0395921	28.57	2	5	65009
protein tetramerization	3.23	0.0395921	28.57	2	5	51262

organic substance transport	3.17	0.0418178	8.46	11	119	71702
regulation of biological process	3.15	0.0428816	7.98	13	150	50789
response to external stimulus	3.07	0.0465527	10.71	6	50	9605
response to extracellular stimulus	3.07	0.0465527	10.71	6	50	9991
organic acid biosynthetic process	3.06	0.0466649	7.02	20	265	16053
carboxylic acid biosynthetic process	3.06	0.0466649	7.02	20	265	46394
peptide:proton symporter activity	2.97	0.0511768	25.00	2	6	15333
polyamine transmembrane transport	2.97	0.0511768	25.00	2	6	1902047
monovalent inorganic cation transmembrane transporter activity	2.97	0.0511768	25.00	2	6	15077
L-fucose catabolic process	2.97	0.0511768	25.00	2	6	42355
disulfide oxidoreductase activity	2.97	0.0511768	25.00	2	6	15036
intracellular pH elevation	2.97	0.0511768	25.00	2	6	51454
glucose metabolic process	2.92	0.0540635	11.36	5	39	6006
small molecule biosynthetic process	2.91	0.0545623	6.75	22	304	44283
arginine metabolic process	2.89	0.0555851	12.90	4	27	6525
arginine biosynthetic process	2.89	0.0555851	12.90	4	27	6526
transcription, DNA-templated	2.81	0.0599645	6.09	37	571	6351
single-species biofilm formation	2.79	0.0615566	10.00	6	54	44010
nucleoside transmembrane transporter activity	2.75	0.0637968	22.22	2	7	5337
neurotransmitter biosynthetic process	2.75	0.0637968	22.22	2	7	42136
glycine metabolic process	2.75	0.0637968	22.22	2	7	6544
glycine biosynthetic process	2.75	0.0637968	22.22	2	7	6545
inorganic cation transmembrane transporter activity	2.75	0.0637968	22.22	2	7	22890
metal ion transmembrane transporter activity	2.75	0.0637968	22.22	2	7	46873

aldehyde catabolic process	2.75	0.0637968	22.22	2	7	46185
glyoxylate catabolic process	2.75	0.0637968	22.22	2	7	9436
regulation of transcription, DNAtemplated	2.74	0.0646306	6.00	39	611	6355
regulation of translation	2.70	0.0672528	12.12	4	29	6417
cation transport	2.59	0.0752402	7.87	10	117	6812
aerobic respiration	2.56	0.0771189	8.86	7	72	9060
NADH dehydrogenase (quinone) activity	2.56	0.0773298	20.00	2	8	50136
dipeptide transmembrane transport	2.56	0.0773298	20.00	2	8	35442
hexose metabolic process	2.47	0.0843215	10.00	5	45	19318
monosaccharide metabolic process	2.47	0.0843215	10.00	5	45	5996
regulation of cellular component biogenesis	2.44	0.0869225	11.11	4	32	44087
sequence-specific DNA binding	2.40	0.0907564	7.23	12	154	43565
glutamine metabolic process	2.40	0.0911556	13.04	3	20	6541
carbohydrate derivative transport	2.40	0.0911556	13.04	3	20	1901264
chromosome condensation	2.39	0.0916627	18.18	2	9	30261
regulation of neurotransmitter levels	2.39	0.0916627	18.18	2	9	1505
neurotransmitter metabolic process	2.39	0.0916627	18.18	2	9	42133
regulation of biological quality	2.39	0.0916627	18.18	2	9	65008
secondary active transmembrane transporter activity	2.39	0.0916627	18.18	2	9	15291
succinate dehydrogenase activity	2.39	0.0916627	18.18	2	9	104
integral component of membrane	2.37	0.0934238	5.28	99	1776	16021
fatty acid oxidation	2.36	0.0940344	10.81	4	33	19395
lipid modification	2.36	0.0940344	10.81	4	33	30258
lipid oxidation	2.36	0.0940344	10.81	4	33	34440

outer membrane	2.35	0.0950116	7.76	9	107	19867
cell outer membrane	2.35	0.0950116	7.76	9	107	9279
regulation of cellular component organization	2.29	0.101412	10.53	4	34	51128
nonribosomal peptide biosynthetic process	2.24	0.106691	16.67	2	10	19184
cation transmembrane transporter activity	2.24	0.106691	16.67	2	10	8324
NADP binding	2.15	0.116935	10.00	4	36	50661
fatty acid metabolic process	2.15	0.116935	10.00	4	36	6631
membrane	2.14	0.117811	5.14	122	2252	16020
external encapsulating structure part	2.12	0.119849	7.38	9	113	44462
transcription regulatory region DNA binding	2.11	0.120963	11.54	3	23	44212
response to hydrogen peroxide	2.10	0.122317	15.38	2	11	42542
proline metabolic process	2.10	0.122317	15.38	2	11	6560
proline biosynthetic process	2.10	0.122317	15.38	2	11	6561
nitrogen compound transport	2.08	0.125133	7.55	8	98	71705
peptidoglycan metabolic process	2.06	0.127729	8.22	6	67	270
glycosaminoglycan metabolic process	2.06	0.127729	8.22	6	67	30203
aminoglycan metabolic process	2.06	0.127729	8.22	6	67	6022
oxidoreductase activity, acting on the CH-OH group of donors, NAD or NADP as acceptor	2.01	0.133421	9.52	4	38	16616
phosphopyruvate hydratase complex	2.01	0.134603	33.33	1	2	15
phosphopyruvate hydratase activity	2.01	0.134603	33.33	1	2	4634
CTP biosynthetic process	2.01	0.134603	33.33	1	2	6241
glycine biosynthetic process	2.01	0.134603	33.33	1	2	6545

'de novo' CTP biosynthetic process	2.01	0.134603	33.33	1	2	44210
regulation of transcription by RNA polymerase II	2.01	0.134603	33.33	1	2	6357
	2.01	0.134603	33.33	1	2	15235
anaerobic ribonucleoside-triphosphate reductase complex	2.01	0.134603	33.33	1	2	31250
glutamate biosynthetic process	1.98	0.138453	14.29	2	12	6537
threonine metabolic process	1.98	0.138453	14.29	2	12	6566
threonine biosynthetic process	1.98	0.138453	14.29	2	12	9088
inorganic molecular entity transmembrane transporter activity	1.98	0.138453	14.29	2	12	15318
diaminopimelate metabolic process	1.95	0.142555	10.71	3	25	46451
lysine metabolic process	1.95	0.142555	10.71	3	25	6553
lysine biosynthetic process	1.95	0.142555	10.71	3	25	9085
lysine biosynthetic process via diaminopimelate	1.95	0.142555	10.71	3	25	9089
xenobiotic metabolic process	1.89	0.150409	8.33	5	55	6805
glutamate metabolic process	1.86	0.155017	13.33	2	13	6536
glutamate biosynthetic process	1.86	0.155017	13.33	2	13	6537
single-species biofilm formation on inanimate substrate	1.86	0.155017	13.33	2	13	44011
organophosphate catabolic process	1.80	0.16531	10.00	3	27	46434
alcohol metabolic process	1.80	0.16531	10.00	3	27	6066
oxidation-reduction process	1.77	0.169919	5.92	20	318	55114
bacteriocin transport	1.76	0.171932	12.50	2	14	43213
dihydrofolate reductase activity	1.74	0.175329	25.00	1	3	4146
alpha,alpha-trehalase activity	1.74	0.175329	25.00	1	3	4555

N-acetylmannosamine metabolic process	1.74	0.175329	25.00	1	3	6051
[formate-C-acetyltransferase]-activating enzyme activity	1.74	0.175329	25.00	1	3	43365
riboflavin reductase (NADPH) activity	1.74	0.175329	25.00	1	3	42602
D-xylose transmembrane transport	1.74	0.175329	25.00	1	3	15753
monocarboxylic acid biosynthetic process	1.72	0.178351	8.51	4	43	72330
L-alpha-amino acid transmembrane transport	1.72	0.178351	8.51	4	43	1902475
L-amino acid transport	1.67	0.187891	8.33	4	44	15807
extrinsic component of cytoplasmic side of plasma membrane	1.67	0.189129	11.76	2	15	31234
glutathione transferase activity	1.67	0.189129	11.76	2	15	4364
DNA repair	1.66	0.18969	7.69	5	60	6281
cellular amino acid metabolic process	1.62	0.197591	8.16	4	45	6520
oxidoreductase activity, acting on CHOH group of donors	1.60	0.201224	9.09	3	30	16614
plasma membrane respiratory chain complex I	1.58	0.206545	11.11	2	16	45272
glyoxylate metabolic process	1.58	0.206545	11.11	2	16	46487
histidine biosynthetic process	1.58	0.206545	11.11	2	16	105
imidazole-containing compound metabolic process	1.58	0.206545	11.11	2	16	52803
histidine metabolic process	1.58	0.206545	11.11	2	16	6547
cell division site	1.54	0.213582	8.82	3	31	32153
N-terminal protein amino acid acetylation	1.50	0.224122	10.53	2	17	6474

generation of precursor metabolites and energy	1.47	0.230897	5.70	18	298	6091
ion transmembrane transport	1.44	0.236345	6.25	9	135	34220
extracellular region	1.43	0.23875	8.33	3	33	5576
oxidoreductase activity, acting on NAD(P)H	1.42	0.241805	10.00	2	18	16651
aminopeptidase activity	1.42	0.241805	10.00	2	18	4177
cellular response to stress	1.39	0.249882	6.94	5	67	33554
cellular response to stimulus	1.39	0.249882	6.94	5	67	51716
cellular response to DNA damage stimulus	1.39	0.249882	6.94	5	67	6974
L-serine biosynthetic process	1.38	0.251139	16.67	1	5	6564
putrescine transport	1.38	0.251139	16.67	1	5	15847
D-tagatose 6-phosphate metabolic process	1.38	0.251139	16.67	1	5	2001058
D-tagatose 6-phosphate catabolic process	1.38	0.251139	16.67	1	5	2001059
phosphorelay response regulator activity	1.38	0.251519	8.11	3	34	156
dicarboxylic acid metabolic process	1.35	0.258863	6.85	5	68	43648
aspartate family amino acid metabolic process	1.35	0.258863	6.85	5	68	9066
aspartate family amino acid biosynthetic process	1.35	0.258863	6.85	5	68	9067
active transmembrane transporter activity	1.28	0.277301	9.09	2	20	22804
hexose biosynthetic process	1.28	0.277301	9.09	2	20	19319
monosaccharide biosynthetic process	1.28	0.277301	9.09	2	20	46364
gluconeogenesis	1.28	0.277301	9.09	2	20	6094
periplasmic side of cell outer membrane	1.25	0.286396	14.29	1	6	31241

acyl-CoA hydrolase activity	1.25	0.286396	14.29	1	6	47617
sulfate transmembrane transporter activity	1.25	0.286396	14.29	1	6	15116
asparagine metabolic process	1.25	0.286396	14.29	1	6	6528
asparagine biosynthetic process	1.25	0.286396	14.29	1	6	6529
amino acid transmembrane transport	1.24	0.290324	6.90	4	54	3333
amino acid transport	1.24	0.290324	6.90	4	54	6865
glucose metabolic process	1.22	0.295029	8.70	2	21	6006
phosphorylation	1.16	0.312694	8.33	2	22	16310
nucleoside phosphate catabolic process	1.16	0.312694	8.33	2	22	1901292
nucleobase-containing small molecule biosynthetic process	1.16	0.312694	8.33	2	22	34404
pyruvate biosynthetic process	1.16	0.312694	8.33	2	22	42866
ADP metabolic process	1.16	0.312694	8.33	2	22	46031
nucleotide phosphorylation	1.16	0.312694	8.33	2	22	46939
glycolytic process	1.16	0.312694	8.33	2	22	6096
nucleoside diphosphate phosphorylation	1.16	0.312694	8.33	2	22	6165
ATP generation from ADP	1.16	0.312694	8.33	2	22	6757
nucleoside diphosphate metabolic process	1.16	0.312694	8.33	2	22	9132
purine nucleoside diphosphate metabolic process	1.16	0.312694	8.33	2	22	9135
nucleotide catabolic process	1.16	0.312694	8.33	2	22	9166
purine ribonucleoside diphosphate metabolic process	1.16	0.312694	8.33	2	22	9179
ribonucleoside diphosphate metabolic process	1.16	0.312694	8.33	2	22	9185
anion transport	1.16	0.314121	6.33	5	74	6820

anion transmembrane transport	1.16	0.314121	6.33	5	74	98656
response to stimulus	1.14	0.321338	5.34	18	319	50896
organonitrogen compound metabolic process	1.13	0.321451	5.04	48	904	1901564
organic anion transport	1.13	0.322591	6.56	4	57	15711
carboxylic acid transmembrane transport	1.13	0.322591	6.56	4	57	1905039
carboxylic acid transport	1.13	0.322591	6.56	4	57	46942
cell part	1.12	0.325872	4.87	128	2502	44464
response to abiotic stimulus	1.12	0.327126	5.88	7	112	9628
transmembrane transport	1.12	0.327389	5.62	10	168	55085
aromatic amino acid family metabolic process	1.11	0.329541	6.98	3	40	9072
aromatic amino acid family biosynthetic process	1.11	0.329541	6.98	3	40	9073
cellular modified amino acid biosynthetic process	1.11	0.330262	8.00	2	23	42398
transporter activity	1.10	0.331505	5.33	17	302	5215
cellular_component	1.08	0.340077	4.85	128	2509	5575
energy derivation by oxidation of organic compounds	1.07	0.342229	5.32	16	285	15980
organic acid transport	1.07	0.344251	6.35	4	59	15849
organic acid transmembrane transport	1.07	0.344251	6.35	4	59	1903825
ATP-dependent peptidase activity	1.04	0.352025	11.11	1	8	4176
cellular protein modification process	1.01	0.364992	7.41	2	25	6464
ion transmembrane transporter activity	1.01	0.364992	7.41	2	25	15075
cellular respiration	1.01	0.365624	5.28	14	251	45333
cellular metabolic process	0.98	0.373695	4.86	91	1781	44237
cytosolic small ribosomal subunit	0.96	0.382102	7.14	2	26	22627

fatty-acyl-CoA binding	0.96	0.382546	10.00	1	9	62
heme transporter activity	0.96	0.382546	10.00	1	9	15232
phosphotransferase activity, phosphate group as acceptor	0.96	0.382546	10.00	1	9	16776
phosphatidylglycerol biosynthetic process	0.96	0.382546	10.00	1	9	6655
glycerophospholipid biosynthetic process	0.96	0.382546	10.00	1	9	46474
phosphoenolpyruvate-dependent sugar phosphotransferase system	0.96	0.382546	10.00	1	9	9401
transferase activity, transferring acyl groups	0.94	0.389952	5.61	6	101	16746
ribosome binding	0.92	0.399014	6.90	2	27	43022
transmembrane transporter activity	0.92	0.399014	6.90	2	27	22857
transporter activity	0.92	0.399014	6.90	2	27	5215
copper ion binding	0.92	0.399014	6.90	2	27	5507
kinase activity	0.92	0.400068	5.14	15	277	16301
cellular process	0.90	0.404724	4.82	97	1915	9987
phosphorylation	0.89	0.41049	5.10	15	279	16310
peptide catabolic process	0.89	0.411635	9.09	1	10	43171
external encapsulating structure	0.88	0.41494	4.86	55	1076	30312
cell wall	0.88	0.41494	4.86	55	1076	5618
peptidoglycan-based cell wall	0.88	0.41494	4.86	55	1076	9274
flavin adenine dinucleotide binding	0.86	0.423991	5.41	6	105	50660
cellular response to extracellular stimulus	0.84	0.432165	6.45	2	29	31668
cellular response to external stimulus	0.84	0.432165	6.45	2	29	71496
cell communication	0.84	0.432165	6.45	2	29	7154

SOS response	0.84	0.432165	6.45	2	29	9432
nucleoside transport	0.82	0.439357	8.33	1	11	15858
nucleobase-containing compound transport	0.82	0.439357	8.33	1	11	15931
nucleoside transmembrane transport	0.82	0.439357	8.33	1	11	1901642
serine family amino acid metabolic process	0.80	0.448373	6.25	2	30	9069
serine family amino acid biosynthetic process	0.80	0.448373	6.25	2	30	9070
cytoplasm	0.79	0.451726	4.80	64	1270	5737
iron ion binding	0.78	0.457765	5.22	6	109	5506
organic hydroxy compound metabolic process	0.78	0.457925	5.66	3	50	1901615
protein refolding	0.76	0.465777	7.69	1	12	42026
biotin metabolic process	0.76	0.465777	7.69	1	12	6768
biotin biosynthetic process	0.76	0.465777	7.69	1	12	9102
galactose transmembrane transport	0.76	0.465777	7.69	1	12	15757
glycerol metabolic process	0.76	0.465777	7.69	1	12	6071
phenylacetate catabolic process	0.76	0.465777	7.69	1	12	10124
carbohydrate transport	0.76	0.465777	7.69	1	12	8643
iron-sulfur cluster binding	0.76	0.46819	4.94	13	250	51536
DNA replication	0.75	0.472715	5.33	4	71	6260
DNA-dependent DNA replication	0.75	0.472715	5.33	4	71	6261
cellular modified amino acid metabolic process	0.73	0.479989	5.88	2	32	6575
sulfate assimilation	0.71	0.490955	7.14	1	13	103
sulfate transmembrane transport	0.71	0.490955	7.14	1	13	1902358
sulfate transport	0.71	0.490955	7.14	1	13	8272

pyruvate metabolic process	0.70	0.495376	5.71	2	33	6090
transferase activity, transferring acyl groups other than amino-acyl groups	0.70	0.495376	5.71	2	33	16747
fatty acid biosynthetic process	0.67	0.510471	5.56	2	34	6633
tryptophan biosynthetic process	0.66	0.514951	6.67	1	14	162
indole-containing compound metabolic process	0.66	0.514951	6.67	1	14	42430
indole-containing compound biosynthetic process	0.66	0.514951	6.67	1	14	42435
indolalkylamine biosynthetic process	0.66	0.514951	6.67	1	14	46219
tryptophan metabolic process	0.66	0.514951	6.67	1	14	6568
indolalkylamine metabolic process	0.66	0.514951	6.67	1	14	6586
carbohydrate derivative catabolic process	0.66	0.514951	6.67	1	14	1901136
response to ionizing radiation	0.62	0.537819	6.25	1	15	10212
defense response to virus	0.58	0.559612	5.88	1	16	51607
pyrimidine nucleotide biosynthetic process	0.54	0.580381	5.56	1	17	6221
inorganic anion transport	0.54	0.580381	5.56	1	17	15698
inorganic anion transmembrane transport	0.54	0.580381	5.56	1	17	98661
sulfur compound metabolic process	0.52	0.593675	4.55	6	126	6790
cellular biogenic amine biosynthetic process	0.52	0.59463	4.76	2	40	42401
amine biosynthetic process	0.52	0.59463	4.76	2	40	9309
cation transmembrane transport	0.50	0.608877	4.49	4	85	98655
DNA binding	0.50	0.608905	4.57	43	898	3677

regulation of single-species biofilm formation	0.48	0.619034	5.00	1	19	1900190
regulation of multi-organism process	0.48	0.619034	5.00	1	19	43900
cellular amine metabolic process	0.48	0.620177	4.55	2	42	44106
cellular biogenic amine metabolic process	0.48	0.620177	4.55	2	42	6576
pyridoxal phosphate binding	0.47	0.626505	4.40	4	87	30170
nucleobase-containing small molecule interconversion	0.47	0.626505	4.40	4	87	15949
ATP metabolic process	0.46	0.632479	4.44	2	43	46034
ATP biosynthetic process	0.46	0.632479	4.44	2	43	6754
nucleoside monophosphate metabolic process	0.46	0.632479	4.44	2	43	9123
nucleoside monophosphate biosynthetic process	0.46	0.632479	4.44	2	43	9124
purine nucleoside monophosphate metabolic process	0.46	0.632479	4.44	2	43	9126
purine nucleoside monophosphate biosynthetic process	0.46	0.632479	4.44	2	43	9127
nucleoside triphosphate metabolic process	0.46	0.632479	4.44	2	43	9141
nucleoside triphosphate biosynthetic process	0.46	0.632479	4.44	2	43	9142
purine nucleoside triphosphate metabolic process	0.46	0.632479	4.44	2	43	9144
purine nucleoside triphosphate biosynthetic process	0.46	0.632479	4.44	2	43	9145
ribonucleoside monophosphate biosynthetic process	0.46	0.632479	4.44	2	43	9156

ribonucleoside monophosphate metabolic process	0.46	0.632479	4.44	2	43	9161
purine ribonucleoside monophosphate metabolic process	0.46	0.632479	4.44	2	43	9167
purine ribonucleoside monophosphate biosynthetic process	0.46	0.632479	4.44	2	43	9168
ribonucleoside triphosphate metabolic process	0.46	0.632479	4.44	2	43	9199
ribonucleoside triphosphate biosynthetic process	0.46	0.632479	4.44	2	43	9201
purine ribonucleoside triphosphate metabolic process	0.46	0.632479	4.44	2	43	9205
purine ribonucleoside triphosphate biosynthetic process	0.46	0.632479	4.44	2	43	9206
dicarboxylic acid biosynthetic process	0.46	0.632479	4.44	2	43	43650
amino sugar biosynthetic process	0.42	0.654138	4.55	1	21	46349
amino sugar metabolic process	0.42	0.654138	4.55	1	21	6040
defense response to bacterium	0.42	0.654138	4.55	1	21	42742
ubiquinone biosynthetic process	0.42	0.654138	4.55	1	21	6744
purine nucleotide metabolic process	0.42	0.660221	4.21	4	91	6163
purine nucleotide biosynthetic process	0.42	0.660221	4.21	4	91	6164
purine-containing compound biosynthetic process	0.42	0.660221	4.21	4	91	72522
purine-containing compound metabolic process	0.40	0.668322	4.17	4	92	72521
ribosome biogenesis	0.40	0.670461	4.35	1	22	42254
alditol metabolic process	0.40	0.670461	4.35	1	22	19400
polyol metabolic process	0.40	0.670461	4.35	1	22	19751

glycerol metabolic process	0.40	0.670461	4.35	1	22	6071
cellular lipid metabolic process	0.39	0.67629	4.12	4	93	44255
lipid metabolic process	0.39	0.67629	4.12	4	93	6629
leucine metabolic process	0.36	0.700839	4.00	1	24	6551
branched-chain amino acid metabolic process	0.36	0.700839	4.00	1	24	9081
branched-chain amino acid biosynthetic process	0.36	0.700839	4.00	1	24	9082
leucine biosynthetic process	0.36	0.700839	4.00	1	24	9098
inorganic ion transmembrane transport	0.35	0.701186	3.95	3	73	98660
dephosphorylation	0.34	0.709612	3.90	3	74	16311
nicotinamide nucleotide biosynthetic process	0.34	0.709994	3.85	2	50	19359
pyridine nucleotide biosynthetic process	0.34	0.709994	3.85	2	50	19363
sulfur compound biosynthetic process	0.33	0.717854	3.85	3	75	44272
monovalent inorganic cation transport	0.33	0.719879	3.77	2	51	15672
proton transmembrane transport	0.33	0.719879	3.77	2	51	1902600
polyamine biosynthetic process	0.32	0.728425	3.70	1	26	6596
phosphotransferase activity, alcohol group as acceptor	0.32	0.72948	3.70	2	52	16773
nitrogen compound metabolic process	0.31	0.73415	4.41	58	1257	6807
pyridine nucleotide metabolic process	0.30	0.7388	3.64	2	53	19362
nicotinamide nucleotide metabolic process	0.30	0.7388	3.64	2	53	46496
oxidoreduction coenzyme metabolic process	0.30	0.7388	3.64	2	53	6733
protein folding	0.30	0.738891	3.90	6	148	6457
amide biosynthetic process	0.30	0.744513	3.87	6	149	43604

cell wall macromolecule metabolic process	0.29	0.747846	3.57	2	54	44036
cell wall macromolecule biosynthetic process	0.29	0.747846	3.57	2	54	44038
aminoglycan biosynthetic process	0.29	0.747846	3.57	2	54	6023
glycosaminoglycan biosynthetic process	0.29	0.747846	3.57	2	54	6024
cellular component macromolecule biosynthetic process	0.29	0.747846	3.57	2	54	70589
peptidoglycan biosynthetic process	0.29	0.747846	3.57	2	54	9252
polyamine metabolic process	0.28	0.753474	3.45	1	28	6595
sulfur compound transport	0.28	0.753474	3.45	1	28	72348
DNA metabolic process	0.27	0.76043	3.85	7	175	6259
peptide biosynthetic process	0.27	0.761519	3.67	4	105	43043
peptide metabolic process	0.27	0.761519	3.67	4	105	6518
inorganic cation transmembrane transport	0.26	0.773387	3.39	2	57	98662
anaerobic respiration	0.25	0.775565	3.86	9	224	9061
membrane	0.24	0.784534	4.27	43	965	16020
DNA recombination	0.24	0.785744	3.54	4	109	6310
cytoplasm	0.23	0.794414	4.29	53	1181	5737
cellular amide metabolic process	0.22	0.800665	3.61	6	160	43603
pyridine-containing compound biosynthetic process	0.20	0.817772	3.08	2	63	72525
manganese ion binding	0.20	0.820649	3.23	3	90	30145
cofactor biosynthetic process	0.19	0.828727	3.40	5	142	51188
protein autophosphorylation	0.18	0.832652	2.70	1	36	46777
protein binding	0.18	0.834047	4.19	47	1075	5515

pyridine-containing compound metabolic process	0.18	0.836917	2.94	2	66	72524
cofactor metabolic process	0.17	0.841607	3.33	5	145	51186
ribose phosphate biosynthetic process	0.17	0.842883	2.90	2	67	46390
purine ribonucleotide metabolic process	0.17	0.842883	2.90	2	67	9150
purine ribonucleotide biosynthetic process	0.17	0.842883	2.90	2	67	9152
ribonucleotide metabolic process	0.17	0.842883	2.90	2	67	9259
ribonucleotide biosynthetic process	0.17	0.842883	2.90	2	67	9260
transferase activity, transferring glycosyl groups	0.17	0.847001	3.06	3	95	16757
DNA integration	0.16	0.854228	2.82	2	69	15074
hydrolase activity, hydrolyzing Oglycosyl compounds	0.14	0.86867	2.38	1	41	4553
iron ion homeostasis	0.14	0.86867	2.38	1	41	55072
electron transfer activity	0.14	0.86867	2.38	1	41	9055
protein modification process	0.14	0.869934	2.91	3	100	36211
cellular protein modification process	0.14	0.869934	2.91	3	100	6464
oxidoreductase activity	0.13	0.874887	2.33	1	42	16491
aromatic compound catabolic process	0.12	0.883926	2.60	2	75	19439
nucleobase-containing compound catabolic process	0.12	0.883926	2.60	2	75	34655
heterocycle biosynthetic process	0.11	0.893398	3.40	10	284	18130
nucleobase-containing small molecule metabolic process	0.11	0.897639	3.27	8	237	55086
cell division	0.10	0.903673	2.68	3	109	51301
ribose phosphate metabolic process	0.10	0.904297	2.44	2	80	19693

nucleoside phosphate biosynthetic process	0.09	0.913305	2.78	4	140	1901293
nucleotide biosynthetic process	0.09	0.913305	2.78	4	140	9165
organic cyclic compound biosynthetic process	0.09	0.914464	3.29	10	294	1901362
nucleoside phosphate metabolic process	0.08	0.921057	2.72	4	143	6753
nucleotide metabolic process	0.08	0.921057	2.72	4	143	9117
organic cyclic compound catabolic process	0.08	0.924337	2.27	2	86	1901361
heterocycle catabolic process	0.08	0.924337	2.27	2	86	46700
amine metabolic process	0.08	0.924337	2.27	2	86	9308
organelle inner membrane	0.07	0.927917	3.81	34	858	19866
organelle membrane	0.07	0.927917	3.81	34	858	31090
amine catabolic process	0.07	0.933441	1.79	1	55	9310
coenzyme biosynthetic process	0.07	0.934095	2.44	3	120	9108
GTP binding	0.07	0.935429	2.17	2	90	5525
organonitrogen compound biosynthetic process	0.06	0.939992	3.58	23	620	1901566
coenzyme metabolic process	0.06	0.940704	2.38	3	123	6732
heme binding	0.06	0.942472	1.69	1	58	20037
organelle part	0.06	0.942493	3.75	34	873	44422
translation	0.05	0.947138	2.06	2	95	6412
carbohydrate derivative metabolic process	0.05	0.948748	3.07	10	316	1901135
water-soluble vitamin biosynthetic process	0.05	0.950282	1.61	1	61	42364
vitamin metabolic process	0.05	0.950282	1.61	1	61	6766
water-soluble vitamin metabolic process	0.05	0.950282	1.61	1	61	6767
vitamin biosynthetic process	0.05	0.950282	1.61	1	61	9110

nucleobase-containing compound biosynthetic process	0.05	0.955842	2.42	4	161	34654
intracellular part	0.04	0.956177	4.09	87	2042	44424
cellular nitrogen compound catabolic process	0.04	0.956812	1.96	2	100	44270
nucleic acid binding	0.04	0.962622	2.35	4	166	3676
cellular protein metabolic process	0.04	0.962796	2.50	5	195	44267
catalytic activity	0.04	0.962872	1.47	1	67	3824
transposition, DNA-mediated	0.04	0.964636	1.45	1	68	6313
aromatic compound biosynthetic process	0.03	0.965641	2.69	7	253	19438
phosphorelay signal transduction system	0.03	0.972335	2.04	3	144	160
molecular_function	0.02	0.977895	1.96	3	150	3674
cellular nitrogen compound biosynthetic process	0.01	0.988391	2.61	10	373	44271
organophosphate metabolic process	0.01	0.988932	2.11	5	232	19637
phosphate-containing compound metabolic process	0.01	0.988932	2.11	5	232	6796
heterocycle metabolic process	0.01	0.990702	3.06	21	665	46483
organophosphate biosynthetic process	0.01	0.990811	1.90	4	206	90407
nucleic acid metabolic process	0.01	0.991892	2.27	7	301	90304
organic cyclic compound metabolic process	0.01	0.992597	3.02	21	675	1901360
macromolecule modification	0.01	0.992776	1.65	3	179	43412
phosphorus metabolic process	0.01	0.993684	2.11	6	279	6793
organic substance biosynthetic process	0.01	0.993706	3.22	30	902	1901576
biosynthetic process	0.01	0.993706	3.22	30	902	9058
cellular macromolecule biosynthetic process	0.01	0.994258	2.29	8	341	34645

protein metabolic process	0.01	0.994271	1.95	5	251	19538
nucleobase-containing compound metabolic process	0.01	0.994496	2.73	15	534	6139
cellular biosynthetic process	0.00	0.9954	3.10	27	844	44249
cellular aromatic compound metabolic process	0.00	0.995845	2.81	18	623	6725
carbohydrate biosynthetic process	0.00	0.99629	1.25	2	158	16051
RNA binding	0.00	0.996333	1.51	3	196	3723
macromolecule biosynthetic process	0.00	0.996644	2.19	8	358	9059
carbohydrate derivative biosynthetic process	0.00	0.996763	1.84	5	267	1901137
cell wall organization	0.00	0.997714	0.80	1	124	71555
cellular nitrogen compound metabolic process	0.00	0.998136	2.85	23	783	34641
cellular carbohydrate metabolic process	0.00	0.998602	0.74	1	134	44262
cellular macromolecule metabolic process	0.00	0.998642	2.45	14	558	44260
nucleotide binding	0.00	0.998772	2.76	22	774	166
intracellular	0.00	0.998928	1.31	3	226	5622
response to antibiotic	0.00	0.999226	0.68	1	146	46677
transferase activity	0.00	0.999431	2.84	28	957	16740
ATP binding	0.00	0.999604	2.47	18	710	5524
macromolecule metabolic process	0.00	0.999803	2.39	18	734	43170

Table S1d. Gene ontology of 44 DEGs.

function	*Enrichment Score*	*Enrichment p-value*	*% genes in group that are present*	*# genes in list, in group*	*# genes not in list, in group*	*GO ID*
tricarboxylic acid cycle	35.90	2.57E-16	36.67	11	19	6099
citrate metabolic process	35.90	2.57E-16	36.67	11	19	6101
tricarboxylic acid metabolic process	35.90	2.57E-16	36.67	11	19	72350
antibiotic metabolic process	33.54	2.72E-15	30.56	11	25	16999
drug metabolic process	20.60	1.13E-09	7.51	13	160	17144
oxidoreduction-driven active transmembrane transporter activity	15.00	3.07E-07	50.00	4	4	15453
oxoglutarate dehydrogenase complex	14.39	5.65E-07	100.00	3	0	45252
protein binding	12.44	3.96E-06	2.14	24	1098	5515
monovalent inorganic cation transport	12.30	4.57E-06	11.32	6	47	15672
proton transmembrane transport	12.30	4.57E-06	11.32	6	47	1902600
intracellular part	11.68	8.49E-06	1.60	34	2095	44424
carboxylic acid metabolic process	11.67	8.53E-06	3.04	15	478	19752
oxoacid metabolic process	11.67	8.53E-06	3.04	15	478	43436
inorganic cation transmembrane transport	11.66	8.63E-06	10.17	6	53	98662
cellular respiration	11.53	9.81E-06	4.15	11	254	45333
succinate-CoA ligase (ADP-forming) activity	11.41	1.11E-05	50.00	3	3	4775
organic acid metabolic process	11.36	1.17E-05	2.96	15	491	6082
metabolic process	10.67	2.33E-05	1.50	35	2294	8152
energy derivation by oxidation of organic compounds	10.33	3.25E-05	3.65	11	290	15980
inorganic ion transmembrane transport	10.19	3.75E-05	7.89	6	70	98660
aerobic respiration	9.97	4.68E-05	7.59	6	73	9060

cellular metabolic process	9.89	5.05E-05	1.60	30	1842	44237
generation of precursor metabolites and energy	9.89	5.08E-05	3.48	11	305	6091
cation transmembrane transport	9.30	9.17E-05	6.74	6	83	98655
oxidation-reduction process	9.28	9.37E-05	3.25	11	327	55114
biological_process	9.02	0.000121433	1.32	39	2924	8150
structural constituent of ribosome	8.91	0.000135163	8.33	5	55	3735
structural molecule activity	8.91	0.000135163	8.33	5	55	5198
cell part	8.82	0.000147482	1.37	36	2594	44464
cellular_component	8.76	0.000157164	1.37	36	2601	5575
structural constituent of ribosome	8.75	0.000158113	8.06	5	57	3735
cysteine synthase activity	8.48	0.000207594	66.67	2	1	4124
cellular process	8.44	0.000216108	1.49	30	1982	9987
fatty acid biosynthetic process	8.44	0.000216978	11.11	4	32	6633
small molecule metabolic process	8.36	0.000234586	2.10	17	794	44281
transferase activity, transferring acyl groups	8.28	0.000253961	5.61	6	101	16746
organic substance metabolic process	8.08	0.000310006	1.49	29	1916	71704
peptidoglycan metabolic process	7.98	0.000342111	6.85	5	68	270
glycosaminoglycan metabolic process	7.98	0.000342111	6.85	5	68	30203
aminoglycan metabolic process	7.98	0.000342111	6.85	5	68	6022
oxidoreductase activity, acting on the CH-OH group of donors, NAD or NADP as acceptor	7.83	0.000397137	9.52	4	38	16616
cell wall organization	7.44	0.000586834	4.80	6	119	71555
cation transport	7.36	0.000638436	4.72	6	121	6812
fatty acid metabolic process	7.32	0.000665024	8.33	4	44	6631
lipoic acid binding	7.29	0.000684517	40.00	2	3	31405
succinyl-CoA metabolic process	7.29	0.000684517	40.00	2	3	6104

3-oxoacyl-[acyl-carrier-protein] synthase activity	6.89	0.00102123	33.33	2	4	4315
capsule polysaccharide biosynthetic process	6.89	0.00102123	33.33	2	4	45227
ion transmembrane transport	6.70	0.00123329	4.17	6	138	34220
translation	6.68	0.00125954	5.15	5	92	6412
cysteine biosynthetic process from serine	6.56	0.001422	28.57	2	5	6535
transcription initiation from bacterialtype RNA polymerase promoter	6.56	0.001422	28.57	2	5	1123
succinate metabolic process	6.56	0.001422	28.57	2	5	6105
cytosolic small ribosomal subunit	6.44	0.00159359	10.71	3	25	22627
cysteine biosynthetic process	6.27	0.00188577	25.00	2	6	19344
cysteine metabolic process	6.27	0.00188577	25.00	2	6	6534
proton-transporting ATP synthase complex, coupling factor F(o)	6.27	0.00188577	25.00	2	6	45263
peptide biosynthetic process	6.16	0.00211534	4.59	5	104	43043
peptide metabolic process	6.16	0.00211534	4.59	5	104	6518
organonitrogen compound biosynthetic process	6.15	0.00213857	2.02	13	630	1901566
primary metabolic process	5.85	0.00288225	1.43	24	1655	44238
transmembrane transport	5.63	0.00360654	3.37	6	172	55085
transferase activity, transferring aminoacyl groups	5.61	0.00364461	18.18	2	9	16755
ion transport	5.52	0.00402379	3.30	6	176	6811
organonitrogen compound metabolic process	5.49	0.00411815	1.68	16	936	1901564

cytoplasm	5.44	0.00432883	1.54	19	1215	5737
translation	5.43	0.00438844	3.88	5	124	6412
plasma membrane ATP synthesis coupled proton transport	5.28	0.00511323	15.38	2	11	42777
organelle inner membrane	5.18	0.00561113	1.68	15	877	19866
organelle membrane	5.18	0.00561113	1.68	15	877	31090
Gram-negative-bacterium-type cell wall biogenesis	5.13	0.00593335	14.29	2	12	43164
organelle part	5.03	0.00655885	1.65	15	892	44422
lipid catabolic process	4.99	0.00680938	13.33	2	13	16042
ATP biosynthetic process	4.74	0.0087253	11.76	2	15	6754
molecular_function	4.72	0.00895563	3.27	5	148	3674
amide biosynthetic process	4.66	0.00944428	3.23	5	150	43604
external encapsulating structure	4.66	0.0094959	1.50	17	1114	30312
cell wall	4.66	0.0094959	1.50	17	1114	5618
peptidoglycan-based cell wall	4.66	0.0094959	1.50	17	1114	9274
DNA-templated transcription, initiation	4.52	0.0108534	10.53	2	17	6352
cellular amide metabolic process	4.38	0.0124653	3.01	5	161	43603
anaerobic respiration	4.34	0.0129992	2.58	6	227	9061
heme binding	4.33	0.0131611	5.08	3	56	20037
energy coupled proton transport, down electrochemical gradient	4.33	0.0131863	9.52	2	19	15985
ATP synthesis coupled proton transport	4.33	0.0131863	9.52	2	19	15986
peptidoglycan metabolic process	4.24	0.0144273	9.09	2	20	270
localization	4.17	0.01544	2.48	6	236	51179
establishment of localization	4.17	0.01544	2.48	6	236	51234

transport	4.17	0.01544	2.48	6	236	6810
membrane	4.08	0.0168795	1.49	15	993	16020
leucine metabolic process	3.99	0.0184379	8.00	2	23	6551
branched-chain amino acid metabolic process	3.99	0.0184379	8.00	2	23	9081
branched-chain amino acid biosynthetic process	3.99	0.0184379	8.00	2	23	9082
leucine biosynthetic process	3.99	0.0184379	8.00	2	23	9098
oxidation-reduction process	3.99	0.018458	1.56	13	819	55114
carbohydrate derivative metabolic process	3.98	0.0186784	2.15	7	319	1901135
sulfur amino acid metabolic process	3.85	0.0213426	7.41	2	25	96
sulfur amino acid biosynthetic process	3.85	0.0213426	7.41	2	25	97
copper ion binding	3.71	0.0244243	6.90	2	27	5507
phosphopyruvate hydratase complex	3.69	0.0250398	33.33	1	2	15
phosphopyruvate hydratase activity	3.69	0.0250398	33.33	1	2	4634
CTP biosynthetic process	3.69	0.0250398	33.33	1	2	6241
glycine biosynthetic process	3.69	0.0250398	33.33	1	2	6545
'de novo' CTP biosynthetic process	3.69	0.0250398	33.33	1	2	44210
cell envelope Sec protein transport complex	3.69	0.0250398	33.33	1	2	31522
mRNA 3'-UTR binding	3.69	0.0250398	33.33	1	2	3730
cellular protein metabolic process	3.66	0.0258	2.50	5	195	44267
cellular macromolecule biosynthetic process	3.65	0.0260525	2.01	7	342	34645
cellular biosynthetic process	3.65	0.0260748	1.49	13	858	44249
endopeptidase activity	3.59	0.0276765	6.45	2	29	4175

serine family amino acid metabolic process	3.53	0.0293646	6.25	2	30	9069
serine family amino acid biosynthetic process	3.53	0.0293646	6.25	2	30	9070
macromolecule biosynthetic process	3.42	0.0326553	1.91	7	359	9059
dihydrofolate reductase activity	3.40	0.0332488	25.00	1	3	4146
protein autophosphorylation	3.26	0.0383931	5.41	2	35	46777
response to temperature stimulus	3.26	0.0383931	5.41	2	35	9266
cellular nitrogen compound biosynthetic process	3.21	0.0403108	1.83	7	376	44271
organic substance biosynthetic process	3.16	0.0424326	1.39	13	919	1901576
biosynthetic process	3.16	0.0424326	1.39	13	919	9058
intracellular	3.16	0.0425853	2.18	5	224	5622
manganese ion binding	3.15	0.0430446	3.23	3	90	30145
cytoplasm	3.14	0.0433996	1.27	17	1317	5737
sequence-specific DNA binding	2.99	0.0503582	2.41	4	162	43565
ATP metabolic process	2.91	0.0547001	4.44	2	43	46034
ATP biosynthetic process	2.91	0.0547001	4.44	2	43	6754
nucleoside monophosphate metabolic process	2.91	0.0547001	4.44	2	43	9123
nucleoside monophosphate biosynthetic process	2.91	0.0547001	4.44	2	43	9124
purine nucleoside monophosphate metabolic process	2.91	0.0547001	4.44	2	43	9126
purine nucleoside monophosphate biosynthetic process	2.91	0.0547001	4.44	2	43	9127
nucleoside triphosphate metabolic process	2.91	0.0547001	4.44	2	43	9141

nucleoside triphosphate biosynthetic process	2.91	0.0547001	4.44	2	43	9142
purine nucleoside triphosphate metabolic process	2.91	0.0547001	4.44	2	43	9144
purine nucleoside triphosphate biosynthetic process	2.91	0.0547001	4.44	2	43	9145
ribonucleoside monophosphate biosynthetic process	2.91	0.0547001	4.44	2	43	9156
ribonucleoside monophosphate metabolic process	2.91	0.0547001	4.44	2	43	9161
purine ribonucleoside monophosphate metabolic process	2.91	0.0547001	4.44	2	43	9167
purine ribonucleoside monophosphate biosynthetic process	2.91	0.0547001	4.44	2	43	9168
ribonucleoside triphosphate metabolic process	2.91	0.0547001	4.44	2	43	9199
ribonucleoside triphosphate biosynthetic process	2.91	0.0547001	4.44	2	43	9201
purine ribonucleoside triphosphate metabolic process	2.91	0.0547001	4.44	2	43	9205
purine ribonucleoside triphosphate biosynthetic process	2.91	0.0547001	4.44	2	43	9206
protein transport	2.78	0.0620692	2.78	3	105	15031
protein metabolic process	2.76	0.0630803	1.95	5	251	19538
cytochrome complex assembly	2.65	0.0706073	3.85	2	50	17004
cellular protein-containing complex assembly	2.65	0.0706073	3.85	2	50	34622
protein-containing complex subunit organization	2.65	0.0706073	3.85	2	50	43933

protein-containing complex assembly	2.65	0.0706073	3.85	2	50	65003
nitrogen compound metabolic process	2.63	0.0719518	1.22	16	1299	6807
cell wall macromolecule metabolic process	2.52	0.0802951	3.57	2	54	44036
cell wall macromolecule biosynthetic process	2.52	0.0802951	3.57	2	54	44038
aminoglycan biosynthetic process	2.52	0.0802951	3.57	2	54	6023
glycosaminoglycan biosynthetic process	2.52	0.0802951	3.57	2	54	6024
cellular component macromolecule biosynthetic process	2.52	0.0802951	3.57	2	54	70589
peptidoglycan biosynthetic process	2.52	0.0802951	3.57	2	54	9252
cellular component assembly	2.49	0.0827791	3.51	2	55	22607
RNA binding	2.46	0.0856476	2.01	4	195	3723
succinate dehydrogenase activity	2.42	0.088841	9.09	1	10	104
cellular component organization	2.40	0.0903707	3.33	2	58	16043
cellular component organization or biogenesis	2.40	0.0903707	3.33	2	58	71840
macromolecule metabolic process	2.36	0.0945646	1.33	10	742	43170
alpha-amino acid metabolic process	2.22	0.108919	1.84	4	213	1901605
alpha-amino acid biosynthetic process	2.22	0.108919	1.84	4	213	1901607
cellular amino acid biosynthetic process	2.20	0.11029	1.83	4	214	8652
oxidoreductase activity, acting on the CH-CH group of donors	2.19	0.111694	7.14	1	13	16627
ribose phosphate biosynthetic process	2.17	0.114278	2.90	2	67	46390
purine ribonucleotide metabolic process	2.17	0.114278	2.90	2	67	9150

purine ribonucleotide biosynthetic process	2.17	0.114278	2.90	2	67	9152
ribonucleotide metabolic process	2.17	0.114278	2.90	2	67	9259
ribonucleotide biosynthetic process	2.17	0.114278	2.90	2	67	9260
transferase activity	2.16	0.11534	1.22	12	973	16740
sulfur compound biosynthetic process	1.97	0.139602	2.56	2	76	44272
pyrimidine nucleotide biosynthetic process	1.96	0.14129	5.56	1	17	6221
ribose phosphate metabolic process	1.89	0.151225	2.44	2	80	19693
regulation of transcription, DNAtemplated	1.74	0.175012	2.22	2	88	6355
glutamine metabolic process	1.73	0.176927	4.35	1	22	6541
cytochrome complex assembly	1.73	0.176927	4.35	1	22	17004
ribosome biogenesis	1.73	0.176927	4.35	1	22	42254
pyridoxal phosphate binding	1.73	0.178029	2.20	2	89	30170
purine nucleotide metabolic process	1.66	0.190176	2.11	2	93	6163
purine nucleotide biosynthetic process	1.66	0.190176	2.11	2	93	6164
purine-containing compound biosynthetic process	1.66	0.190176	2.11	2	93	72522
purine-containing compound metabolic process	1.64	0.193231	2.08	2	94	72521
carbohydrate derivative biosynthetic process	1.64	0.194726	1.47	4	268	1901137
regulation of nucleic acid-templated transcription	1.63	0.196294	2.06	2	95	1903506
regulation of RNA biosynthetic process	1.63	0.196294	2.06	2	95	2001141
regulation of RNA metabolic process	1.63	0.196294	2.06	2	95	51252

transferase activity, transferring glycosyl groups	1.61	0.199362	2.04	2	96	16757
regulation of gene expression	1.61	0.199362	2.04	2	96	10468
regulation of nucleobase-containing compound metabolic process	1.60	0.202437	2.02	2	97	19219
cellular macromolecule metabolic process	1.60	0.202649	1.22	7	565	44260
regulation of cellular macromolecule biosynthetic process	1.58	0.205517	2.00	2	98	2000112
organic acid biosynthetic process	1.53	0.217576	1.40	4	281	16053
carboxylic acid biosynthetic process	1.53	0.217576	1.40	4	281	46394
regulation of macromolecule biosynthetic process	1.48	0.227217	1.87	2	105	10556
regulation of metabolic process	1.48	0.227217	1.87	2	105	19222
regulation of cellular metabolic process	1.48	0.227217	1.87	2	105	31323
regulation of cellular biosynthetic process	1.48	0.227217	1.87	2	105	31326
regulation of nitrogen compound metabolic process	1.48	0.227217	1.87	2	105	51171
regulation of macromolecule metabolic process	1.48	0.227217	1.87	2	105	60255
regulation of primary metabolic process	1.48	0.227217	1.87	2	105	80090
regulation of biosynthetic process	1.48	0.227217	1.87	2	105	9889
cytosolic large ribosomal subunit	1.44	0.237449	3.13	1	31	22625
regulation of translation	1.41	0.243898	3.03	1	32	6417
transcription, DNA-templated	1.39	0.247873	1.15	7	601	6351
iron ion binding	1.38	0.252219	1.74	2	113	5506

extracellular region	1.34	0.262924	2.78	1	35	5576
response to abiotic stimulus	1.33	0.264761	1.68	2	117	9628
NADP binding	1.25	0.287562	2.50	1	39	50661
small molecule biosynthetic process	1.23	0.293756	1.23	4	322	44283
regulation of transcription, DNAtemplated	1.19	0.304357	1.08	7	643	6355
sulfur compound metabolic process	1.19	0.305502	1.52	2	130	6790
cellular amino acid metabolic process	1.14	0.318763	1.18	4	335	6520
nucleoside phosphate biosynthetic process	1.07	0.342791	1.39	2	142	1901293
nucleotide biosynthetic process	1.07	0.342791	1.39	2	142	9165
nucleoside phosphate metabolic process	1.04	0.35203	1.36	2	145	6753
nucleotide metabolic process	1.04	0.35203	1.36	2	145	9117
phosphorelay signal transduction system	1.04	0.35203	1.36	2	145	160
protein folding	0.99	0.373418	1.30	2	152	6457
DNA-binding transcription factor activity	0.98	0.375116	1.09	4	364	3700
regulation of cellular process	0.98	0.376452	1.29	2	153	50794
regulation of biological process	0.92	0.400512	1.23	2	161	50789
nucleobase-containing compound biosynthetic process	0.90	0.406464	1.21	2	163	34654
periplasmic space	0.86	0.424153	1.17	2	169	42597
biological regulation	0.82	0.441579	1.13	2	175	65007
cellular nitrogen compound metabolic process	0.64	0.525767	0.87	7	799	34641
organophosphate biosynthetic process	0.63	0.532051	0.95	2	208	90407

ATP binding	0.54	0.58552	0.82	6	722	5524
organophosphate metabolic process	0.51	0.598543	0.84	2	235	19637
phosphate-containing compound metabolic process	0.51	0.598543	0.84	2	235	6796
nucleobase-containing small molecule metabolic process	0.48	0.616875	0.82	2	243	55086
aromatic compound biosynthetic process	0.43	0.649551	0.77	2	258	19438
nucleotide binding	0.39	0.675287	0.75	6	790	166
DNA binding	0.36	0.696038	0.74	7	934	3677
phosphorus metabolic process	0.36	0.699168	0.70	2	283	6793
response to antibiotic	0.34	0.715288	0.68	1	146	46677
heterocycle biosynthetic process	0.33	0.715588	0.68	2	292	18130
organic cyclic compound biosynthetic process	0.31	0.732966	0.66	2	302	1901362
membrane	0.28	0.754002	0.76	18	2356	16020
nucleic acid binding	0.27	0.766696	0.59	1	169	3676
response to stimulus	0.24	0.784162	0.59	2	335	50896
integral component of membrane	0.18	0.835941	0.69	13	1862	16021
carbohydrate catabolic process	0.15	0.863624	0.49	2	403	16052
iron-sulfur cluster binding	0.11	0.896472	0.38	1	262	51536
nucleobase-containing compound metabolic process	0.05	0.952061	0.36	2	547	6139
carbohydrate metabolic process	0.04	0.962426	0.34	2	579	5975
organic substance catabolic process	0.03	0.975293	0.31	2	633	1901575
catabolic process	0.03	0.975293	0.31	2	633	9056
cellular aromatic compound metabolic process	0.02	0.976432	0.31	2	639	6725
heterocycle metabolic process	0.02	0.983517	0.29	2	684	46483

organic cyclic compound metabolic process	0.02	0.984789	0.29	2	694	1901360

Table S1e. Gene ontology of 508 DEGs.

function	*Enrichment Score*	*Enrichment p-value*	*% genes in group that are present*	*# genes in list, in group*	*# genes not in list, in group*	*GO ID*
alditol metabolic process	14.90	3.38E-07	39.13	9	14	19400
polyol metabolic process	14.90	3.38E-07	39.13	9	14	19751
glycerol metabolic process	14.90	3.38E-07	39.13	9	14	6071
cell projection	13.18	1.88E-06	20.29	14	55	42995
pilus	13.18	1.88E-06	20.29	14	55	9289
'de novo' UMP biosynthetic process	12.66	3.17E-06	54.55	6	5	44205
purine nucleotide biosynthetic process	12.64	3.25E-06	31.03	9	20	6164
alcohol metabolic process	12.32	4.46E-06	30.00	9	21	6066
pyrimidine nucleotide biosynthetic process	11.78	7.65E-06	38.89	7	11	6221
urea cycle	10.15	3.89E-05	50.00	5	5	50
'de novo' pyrimidine nucleobase biosynthetic process	10.00	4.53E-05	37.50	6	10	6207
transposition, DNA-mediated	9.85	5.27E-05	17.39	12	57	6313
glycerol-3-phosphate metabolic process	9.75	5.81E-05	66.67	4	2	6072
ATP-dependent RNA helicase activity	8.64	0.000177448	38.46	5	8	4004
RNA secondary structure unwinding	8.64	0.000177448	38.46	5	8	10501

glycerol metabolic process	8.64	0.000177448	38.46	5	8	6071
nucleoside phosphate biosynthetic process	8.26	0.000257572	11.81	17	127	1901293
nucleotide biosynthetic process	8.26	0.000257572	11.81	17	127	9165
nucleoside phosphate metabolic process	8.02	0.000330005	11.56	17	130	6753
nucleotide metabolic process	8.02	0.000330005	11.56	17	130	9117
organic hydroxy compound metabolic process	7.50	0.000550417	16.98	9	44	1901615
outer membrane	7.24	0.000715927	12.07	14	102	19867
cell outer membrane	7.24	0.000715927	12.07	14	102	9279
external encapsulating structure part	6.74	0.00118371	11.48	14	108	44462
nucleobase-containing compound biosynthetic process	6.68	0.00124998	10.30	17	148	34654
nucleobase-containing small molecule metabolic process	6.43	0.00161344	8.98	22	223	55086
pyrimidine nucleotide metabolic process	6.41	0.00164414	25.00	5	15	6220
pyrimidine nucleotide biosynthetic process	6.41	0.00164414	25.00	5	15	6221
small molecule metabolic process	6.18	0.00206783	6.66	54	757	44281
organophosphate biosynthetic process	5.80	0.00304054	9.05	19	191	90407
glutamine metabolic process	5.75	0.00319072	21.74	5	18	6541
purine nucleotide metabolic process	5.62	0.00363517	11.58	11	84	6163

purine nucleotide biosynthetic process	5.62	0.00363517	11.58	11	84	6164
purine-containing compound biosynthetic process	5.62	0.00363517	11.58	11	84	72522
tryptophan biosynthetic process	5.57	0.00381463	26.67	4	11	162
indole-containing compound metabolic process	5.57	0.00381463	26.67	4	11	42430
indole-containing compound biosynthetic process	5.57	0.00381463	26.67	4	11	42435
indolalkylamine biosynthetic process	5.57	0.00381463	26.67	4	11	46219
tryptophan metabolic process	5.57	0.00381463	26.67	4	11	6568
indolalkylamine metabolic process	5.57	0.00381463	26.67	4	11	6586
purine-containing compound metabolic process	5.54	0.00394414	11.46	11	85	72521
cellular_component	5.39	0.00454206	5.38	142	2495	5575
cell adhesion	5.30	0.0049986	11.11	11	88	7155
cell part	5.16	0.005722	5.36	141	2489	44464
amidase activity	5.12	0.00597498	66.67	2	1	4040
ribosome assembly	5.12	0.00597498	66.67	2	1	42255
aspartate carbamoyltransferase activity	5.12	0.00597498	66.67	2	1	4070
lysine catabolic process	5.12	0.00597498	66.67	2	1	6554
glycine metabolic process	5.12	0.00597498	66.67	2	1	6544
aromatic compound biosynthetic process	4.95	0.00709874	8.08	21	239	19438
nucleobase-containing compound metabolic process	4.71	0.00902361	6.74	37	512	6139
intrinsic component of plasma membrane	4.67	0.00938632	21.05	4	15	31226
nucleic acid binding	4.60	0.0100833	8.82	15	155	3676

organophosphate metabolic process	4.51	0.0110111	8.02	19	218	19637
phosphate-containing compound metabolic process	4.51	0.0110111	8.02	19	218	6796
2,4,6-trinitrotoluene catabolic process	4.46	0.011592	50.00	2	2	46256
water channel activity	4.46	0.011592	50.00	2	2	15250
glycerol channel activity	4.46	0.011592	50.00	2	2	15254
3-hydroxypropionate dehydrogenase (NADP+) activity	4.46	0.011592	50.00	2	2	35527
plasmid partitioning	4.46	0.011592	50.00	2	2	30541
chromosome organization	4.46	0.011592	50.00	2	2	51276
cellular response to zinc ion starvation	4.46	0.011592	50.00	2	2	34224
biotin carboxyl carrier protein biosynthetic process	4.46	0.011592	50.00	2	2	42966
glycine catabolic process	4.46	0.011592	50.00	2	2	6546
hypoxanthine phosphoribosyltransferase activity	4.46	0.011592	50.00	2	2	4422
GMP salvage	4.46	0.011592	50.00	2	2	32263
nitric oxide catabolic process	4.46	0.011592	50.00	2	2	46210
response to nitric oxide	4.46	0.011592	50.00	2	2	71731
cellular response to osmotic stress	4.46	0.011592	50.00	2	2	71470
fimbrial usher porin activity	4.45	0.011639	27.27	3	8	15473
pilus organization	4.42	0.0119797	16.13	5	26	43711
cellular aromatic compound metabolic process	4.25	0.0142264	6.40	41	600	6725
primary metabolic process	4.21	0.0148308	5.54	93	1586	44238
translational termination	4.20	0.0150049	25.00	3	9	6415
DNA binding	4.03	0.0177188	5.95	56	885	3677
ribosome biogenesis	3.98	0.0185998	17.39	4	19	42254

transcription antitermination factor activity, RNA binding	3.98	0.0187435	40.00	2	3	1072
single-stranded RNA binding	3.98	0.0187435	40.00	2	3	3727
cyanate metabolic process	3.98	0.0187435	40.00	2	3	9439
cyanate catabolic process	3.98	0.0187435	40.00	2	3	9440
thiamine metabolic process	3.98	0.0187435	40.00	2	3	6772
lipopolysaccharide binding	3.98	0.0187435	40.00	2	3	1530
lipoic acid binding	3.98	0.0187435	40.00	2	3	31405
response to hydrogen peroxide	3.97	0.0188622	23.08	3	10	42542
cellular carbohydrate metabolic process	3.95	0.0192266	8.89	12	123	44262
cytoplasm	3.82	0.02197	5.62	75	1259	5737
cellular nitrogen compound biosynthetic process	3.70	0.0247716	6.79	26	357	44271
heterocycle biosynthetic process	3.67	0.0255671	7.14	21	273	18130
hydroxymethyl-, formyl- and related transferase activity	3.60	0.0272796	33.33	2	4	16742
copper ion transmembrane transporter activity	3.60	0.0272796	33.33	2	4	5375
lysophospholipase activity	3.60	0.0272796	33.33	2	4	4622
cysteine desulfurase activity	3.60	0.0272796	33.33	2	4	31071
IMP salvage	3.60	0.0272796	33.33	2	4	32264
tRNA (uracil-2'-O-)-methyltransferase activity	3.60	0.0272796	33.33	2	4	52665
tRNA (cytosine-2'-O-)methyltransferase activity	3.60	0.0272796	33.33	2	4	52666
purine nucleotide metabolic process	3.60	0.0272796	33.33	2	4	6163
oxidoreductase activity, acting on other nitrogenous compounds as donors	3.60	0.0272796	33.33	2	4	16661

aromatic amino acid transmembrane transporter activity	3.60	0.0272796	33.33	2	4	15173
cellular nitrogen compound metabolic process	3.59	0.0274903	5.96	48	758	34641
pyrimidine-containing compound metabolic process	3.50	0.0302754	12.82	5	34	72527
pyrimidine-containing compound biosynthetic process	3.50	0.0302754	12.82	5	34	72528
transcription antitermination	3.40	0.0334102	18.75	3	13	31564
phosphorus metabolic process	3.38	0.0340185	7.02	20	265	6793
response to antibiotic	3.37	0.0345398	8.16	12	135	46677
organic cyclic compound biosynthetic process	3.35	0.035114	6.91	21	283	1901362
diaminopimelate metabolic process	3.32	0.0360507	14.29	4	24	46451
lysine metabolic process	3.32	0.0360507	14.29	4	24	6553
lysine biosynthetic process	3.32	0.0360507	14.29	4	24	9085
lysine biosynthetic process via diaminopimelate	3.32	0.0360507	14.29	4	24	9089
RNA binding	3.31	0.036421	7.54	15	184	3723
tRNA wobble position uridine thiolation	3.30	0.0370606	28.57	2	5	2143
response to silver ion	3.30	0.0370606	28.57	2	5	10272
copper ion export	3.30	0.0370606	28.57	2	5	60003
tetrapyrrole biosynthetic process	3.30	0.0370606	28.57	2	5	33014
sister chromatid cohesion	3.30	0.0370606	28.57	2	5	7062
catalytic complex	3.30	0.0370606	28.57	2	5	1902494
protein tetramerization	3.30	0.0370606	28.57	2	5	51262
heterocycle metabolic process	3.26	0.0385454	5.98	41	645	46483

cellular amino acid biosynthetic process	3.25	0.0386762	7.34	16	202	8652
iron ion homeostasis	3.22	0.0401055	11.90	5	37	55072
metalloendopeptidase activity	3.21	0.0403766	13.79	4	25	4222
rRNA base methylation	3.10	0.0449845	13.33	4	26	70475
organic cyclic compound metabolic process	3.06	0.0468516	5.89	41	655	1901360
detoxification of copper ion	3.04	0.0479568	25.00	2	6	10273
K antigen metabolic process	3.04	0.0479568	25.00	2	6	46375
K antigen biosynthetic process	3.04	0.0479568	25.00	2	6	9248
Kdo2-lipid A biosynthetic process	3.04	0.0479568	25.00	2	6	36104
carboxyl- or carbamoyltransferase activity	3.04	0.0479568	25.00	2	6	16743
iron assimilation	3.04	0.0479568	25.00	2	6	33212
nickel cation transport	3.04	0.0479568	25.00	2	6	15675
cellular metabolic process	2.96	0.0516859	5.24	98	1774	44237
arginine biosynthetic process	2.95	0.0523373	15.79	3	16	6526
cellular amino acid metabolic process	2.88	0.0561833	6.49	22	317	6520

ATP binding	2.84	0.0586834	5.77	42	686	5524
purine ribonucleoside salvage	2.82	0.0595753	15.00	3	17	6166
regulation of single-species biofilm formation	2.82	0.0595753	15.00	3	17	1900190
regulation of multi-organism process	2.82	0.0595753	15.00	3	17	43900
phosphoprotein phosphatase activity	2.82	0.0595753	15.00	3	17	4721
DNA methylation	2.82	0.0598475	22.22	2	7	6306
DNA methylation on adenine	2.82	0.0598475	22.22	2	7	32775
toxic substance binding	2.82	0.0598475	22.22	2	7	15643
fermentation	2.80	0.0609662	9.68	6	56	6113

oligosaccharide metabolic process	2.71	0.0662151	11.76	4	30	9311
oligosaccharide biosynthetic process	2.71	0.0662151	11.76	4	30	9312
alpha-amino acid metabolic process	2.69	0.067834	6.91	15	202	1901605
alpha-amino acid biosynthetic process	2.69	0.067834	6.91	15	202	1901607
double-strand break repair	2.62	0.0726206	20.00	2	8	6302
organic substance metabolic process	2.60	0.0742139	5.14	100	1845	71704
defense response to bacterium	2.59	0.0753606	13.64	3	19	42742
organonitrogen compound biosynthetic process	2.58	0.0756664	5.75	37	606	1901566
cytoplasm	2.56	0.0771253	5.35	66	1168	5737
macromolecule catabolic process	2.50	0.0823294	8.96	6	61	9057
response to temperature stimulus	2.47	0.0849934	10.81	4	33	9266
DNA catabolic process	2.47	0.0849934	10.81	4	33	6308
negative regulation of DNA-templated transcription, termination	2.45	0.086172	18.18	2	9	60567
3'-5'-exoribonuclease activity	2.45	0.086172	18.18	2	9	175
ATPase activity, coupled	2.45	0.086172	18.18	2	9	42623
cellular nitrogen compound catabolic process	2.40	0.0911285	7.84	8	94	44270
protein dephosphorylation	2.38	0.0927793	12.50	3	21	6470
cellular process	2.34	0.0966573	5.07	102	1910	9987
cellular macromolecule catabolic process	2.34	0.0968107	9.26	5	49	44265
response to drug	2.31	0.0992384	7.69	8	96	42493
glutamine metabolic process	2.30	0.100405	16.67	2	10	6541
glutamine biosynthetic process	2.30	0.100405	16.67	2	10	6542
organic phosphonate transport	2.30	0.100405	16.67	2	10	15716
metabolic process	2.17	0.113686	4.98	116	2213	8152

phosphorelay sensor kinase activity	2.16	0.115203	8.77	5	52	155
signal transduction by protein phosphorylation	2.16	0.115203	8.77	5	52	23014
DNA topological change	2.16	0.115231	15.38	2	11	6265
cellular aromatic compound metabolic process	2.16	0.115231	15.38	2	11	6725
intrinsic component of periplasmic side of plasma membrane	2.16	0.115231	15.38	2	11	31237
nucleobase-containing small molecule interconversion	2.13	0.118358	7.69	7	84	15949
cellular biogenic amine biosynthetic process	2.11	0.121311	9.52	4	38	42401
amine biosynthetic process	2.11	0.121311	9.52	4	38	9309
aromatic amino acid family metabolic process	2.05	0.129265	9.30	4	39	9072
aromatic amino acid family biosynthetic process	2.05	0.129265	9.30	4	39	9073
phosphopyruvate hydratase complex	2.04	0.130091	33.33	1	2	15
phosphopyruvate hydratase activity	2.04	0.130091	33.33	1	2	4634
CTP biosynthetic process	2.04	0.130091	33.33	1	2	6241
glycine biosynthetic process	2.04	0.130091	33.33	1	2	6545
'de novo' CTP biosynthetic process	2.04	0.130091	33.33	1	2	44210
organophosphate:inorganic phosphate antiporter activity	2.04	0.130091	33.33	1	2	15315
integral component of membrane	2.04	0.130091	33.33	1	2	16021
response to amino acid	2.04	0.130091	33.33	1	2	43200
positive regulation of oxidoreductase activity	2.04	0.130091	33.33	1	2	51353

glutamate biosynthetic process	2.04	0.130567	14.29	2	12	6537
RNA methylation	2.04	0.130567	14.29	2	12	1510
single-species biofilm formation	2.00	0.135129	8.33	5	55	44010
cellular amine metabolic process	1.98	0.137429	9.09	4	40	44106
cellular biogenic amine metabolic process	1.98	0.137429	9.09	4	40	6576
cellular catabolic process	1.97	0.13913	6.25	14	210	44248
energy derivation by oxidation of organic compounds	1.97	0.139223	5.98	18	283	15980
carbohydrate metabolic process	1.95	0.142772	5.51	32	549	5975
iron ion binding	1.89	0.150721	6.96	8	107	5506
uracil catabolic process	1.82	0.162469	12.50	2	14	6212
response to ionizing radiation	1.82	0.162469	12.50	2	14	10212
arginine metabolic process	1.81	0.16439	9.68	3	28	6525
arginine biosynthetic process	1.81	0.16439	9.68	3	28	6526
dihydrofolate reductase activity	1.77	0.169591	25.00	1	3	4146
glycerol-3-phosphate O-acyltransferase activity	1.77	0.169591	25.00	1	3	4366
phospholipid metabolic process	1.77	0.169591	25.00	1	3	6644
glutathione metabolic process	1.77	0.169591	25.00	1	3	6749
glutathione biosynthetic process	1.77	0.169591	25.00	1	3	6750
organic phosphonate transmembrane transporter activity	1.77	0.169591	25.00	1	3	15604
phosphate ion homeostasis	1.77	0.169591	25.00	1	3	55062
lipooligosaccharide metabolic process	1.72	0.1789	11.76	2	15	1901269
lipooligosaccharide biosynthetic process	1.72	0.1789	11.76	2	15	1901271
membrane lipid biosynthetic process	1.72	0.1789	11.76	2	15	46467

lipid A metabolic process	1.72	0.1789	11.76	2	15	46493
membrane lipid metabolic process	1.72	0.1789	11.76	2	15	6643
glycolipid metabolic process	1.72	0.1789	11.76	2	15	6664
lipid A biosynthetic process	1.72	0.1789	11.76	2	15	9245
glycolipid biosynthetic process	1.72	0.1789	11.76	2	15	9247
cellular amino acid metabolic process	1.71	0.181105	8.16	4	45	6520
organonitrogen compound metabolic process	1.68	0.185603	5.15	49	903	1901564
generation of precursor metabolites and energy	1.67	0.188194	5.70	18	298	6091
carbohydrate binding	1.66	0.190346	8.00	4	46	30246
cellular biosynthetic process	1.65	0.19142	5.17	45	826	44249
nitrogen compound metabolic process	1.64	0.193802	5.02	66	1249	6807
integral component of cell outer membrane	1.63	0.195568	11.11	2	16	45203
rRNA processing	1.60	0.202874	7.25	5	64	6364
glutathione biosynthetic process	1.57	0.207303	20.00	1	4	6750
intrinsic component of membrane	1.57	0.207303	20.00	1	4	31224
fructose 1,6-bisphosphate 1phosphatase activity	1.57	0.207303	20.00	1	4	42132
translation release factor activity	1.57	0.207303	20.00	1	4	3747
cytochrome complex assembly	1.56	0.209257	7.69	4	48	17004
cellular protein-containing complex assembly	1.56	0.209257	7.69	4	48	34622
protein-containing complex subunit organization	1.56	0.209257	7.69	4	48	43933
protein-containing complex assembly	1.56	0.209257	7.69	4	48	65003
RNA modification	1.56	0.211025	7.14	5	65	9451

extracellular polysaccharide biosynthetic process	1.55	0.212418	10.53	2	17	45226
glutamine family amino acid metabolic process	1.52	0.21928	7.04	5	66	9064
glutamine family amino acid biosynthetic process	1.52	0.21928	7.04	5	66	9084
organic acid biosynthetic process	1.50	0.222287	5.61	16	269	16053
carboxylic acid biosynthetic process	1.50	0.222287	5.61	16	269	46394
transition metal ion transport	1.50	0.222845	8.33	3	33	41
iron ion transport	1.50	0.222845	8.33	3	33	6826
signal transduction	1.48	0.227633	6.94	5	67	7165
mRNA catabolic process	1.47	0.229401	10.00	2	18	6402
protein autophosphorylation	1.45	0.235008	8.11	3	34	46777
organic substance biosynthetic process	1.45	0.235189	5.04	47	885	1901576
biosynthetic process	1.45	0.235189	5.04	47	885	9058
aspartate family amino acid metabolic process	1.44	0.236078	6.85	5	68	9066
aspartate family amino acid biosynthetic process	1.44	0.236078	6.85	5	68	9067
arginine biosynthetic process via ornithine	1.41	0.243307	16.67	1	5	42450
response to acidic pH	1.40	0.246469	9.52	2	19	10447
lipopolysaccharide core region metabolic process	1.40	0.246469	9.52	2	19	46401
lipopolysaccharide core region biosynthetic process	1.40	0.246469	9.52	2	19	9244
cellular component assembly	1.35	0.258586	7.02	4	53	22607
anaerobic respiration	1.35	0.259503	5.58	13	220	9061
glycoprotein metabolic process	1.33	0.263579	9.09	2	20	9100

glycoprotein biosynthetic process	1.33	0.263579	9.09	2	20	9101
nucleotide binding	1.32	0.265961	5.03	40	756	166
aromatic compound catabolic process	1.31	0.270665	6.49	5	72	19439
nucleobase-containing compound catabolic process	1.31	0.270665	6.49	5	72	34655
regulation of transcription, DNAtemplated	1.31	0.270775	5.08	33	617	6355
transcription, DNA-templated	1.31	0.270888	5.10	31	577	6351
metal ion transport	1.30	0.272112	7.50	3	37	30001
oxidation-reduction process	1.30	0.273114	5.33	18	320	55114
response to chemical	1.29	0.276014	5.88	8	128	42221
type II protein secretion system complex	1.28	0.277682	14.29	1	6	15627
asparagine metabolic process	1.28	0.277682	14.29	1	6	6528
asparagine biosynthetic process	1.28	0.277682	14.29	1	6	6529
heme binding	1.28	0.278938	6.78	4	55	20037
organic acid metabolic process	1.27	0.280097	5.14	26	480	6082
ion transmembrane transport	1.27	0.280694	8.70	2	21	34220
endoribonuclease activity	1.27	0.280694	8.70	2	21	4521
chaperone-mediated protein folding	1.26	0.284628	7.32	3	38	61077
xenobiotic metabolic process	1.24	0.289211	6.67	4	56	6805
cellular component organization	1.24	0.289211	6.67	4	56	16043
cellular component organization or biogenesis	1.24	0.289211	6.67	4	56	71840
organic substance catabolic process	1.24	0.289606	5.04	32	603	1901575
catabolic process	1.24	0.289606	5.04	32	603	9056

oxidoreductase activity, acting on the CH-OH group of donors, NAD or NADP as acceptor	1.21	0.297191	7.14	3	39	16616
Gram-negative-bacterium-type cell outer membrane assembly	1.21	0.297777	8.33	2	22	43165
intracellular part	1.17	0.310611	4.74	101	2028	44424
DNA-binding transcription factor activity	1.16	0.311944	5.16	19	349	3700
liposaccharide metabolic process	1.16	0.314797	8.00	2	23	1903509
biological_process	1.15	0.316369	4.69	139	2824	8150
cellular respiration	1.15	0.316651	5.28	14	251	45333
membrane	1.14	0.32077	4.86	49	959	16020
cellular amino acid catabolic process	1.10	0.333431	5.77	6	98	9063
cellular modified amino acid catabolic process	1.07	0.341831	11.11	1	8	42219
carnitine catabolic process	1.07	0.341831	11.11	1	8	42413
amino-acid betaine catabolic process	1.07	0.341831	11.11	1	8	6579
carnitine metabolic process	1.07	0.341831	11.11	1	8	9437
negative regulation of single-species biofilm formation	1.07	0.341831	11.11	1	8	1900191
negative regulation of multi-organism process	1.07	0.341831	11.11	1	8	43901
exonuclease activity	1.06	0.347615	6.52	3	43	4527
RNA metabolic process	1.00	0.369504	5.43	7	122	16070
organic cyclic compound catabolic process	0.99	0.369855	5.68	5	83	1901361
heterocycle catabolic process	0.99	0.369855	5.68	5	83	46700
amine metabolic process	0.99	0.369855	5.68	5	83	9308

L-serine metabolic process	0.99	0.371743	10.00	1	9	6563
L-serine biosynthetic process	0.99	0.371743	10.00	1	9	6564
proteolysis	0.99	0.371743	10.00	1	9	6508
nickel cation transmembrane transport	0.99	0.371743	10.00	1	9	35444
periplasmic space	0.99	0.372794	5.26	9	162	42597
copper ion binding	0.96	0.38169	6.90	2	27	5507
carboxylic acid metabolic process	0.94	0.389762	4.87	24	469	19752
oxoacid metabolic process	0.94	0.389762	4.87	24	469	43436
protein histidine kinase activity	0.92	0.397678	6.00	3	47	4673
tricarboxylic acid cycle	0.92	0.398002	6.67	2	28	6099
citrate metabolic process	0.92	0.398002	6.67	2	28	6101
tricarboxylic acid metabolic process	0.92	0.398002	6.67	2	28	72350
amino acid-transporting ATPase activity	0.92	0.4003	9.09	1	10	15424
ribosomal large subunit assembly	0.92	0.4003	9.09	1	10	27
plasmid maintenance	0.92	0.4003	9.09	1	10	6276
ammonium ion metabolic process	0.92	0.4003	9.09	1	10	97164
GTP binding	0.90	0.406361	5.43	5	87	5525
small molecule biosynthetic process	0.89	0.409821	4.91	16	310	44283
carboxylic acid catabolic process	0.88	0.415427	5.26	6	108	46395
iron-sulfur cluster binding	0.88	0.416317	4.94	13	250	51536
organonitrogen compound catabolic process	0.87	0.418734	5.06	9	169	1901565
dicarboxylic acid metabolic process	0.86	0.424495	5.48	4	69	43648
nucleic acid metabolic process	0.85	0.427071	4.87	15	293	90304
nonribosomal peptide biosynthetic process	0.85	0.427564	8.33	1	11	19184

polysaccharide catabolic process	0.85	0.427564	8.33	1	11	272
iron-sulfur cluster assembly	0.84	0.430008	6.25	2	30	16226
DNA metabolic process	0.81	0.444981	4.95	9	173	6259
monosaccharide-transporting ATPase activity	0.79	0.453591	7.69	1	12	15407
cellular modified amino acid metabolic process	0.77	0.461093	5.88	2	32	6575
nickel cation binding	0.77	0.461093	5.88	2	32	16151
phosphate ion transmembrane transport	0.74	0.478439	7.14	1	13	35435
membrane part	0.74	0.478439	7.14	1	13	44425
amino-acid betaine metabolic process	0.74	0.478439	7.14	1	13	6577
organic acid catabolic process	0.73	0.480354	4.92	6	116	16054
transporter activity	0.73	0.482415	4.70	15	304	5215
sequence-specific DNA binding	0.73	0.483084	4.82	8	158	43565
small molecule catabolic process	0.72	0.488337	4.88	6	117	44282
protein-containing complex	0.71	0.491168	5.56	2	34	32991
antibiotic metabolic process	0.71	0.491168	5.56	2	34	16999
2 iron, 2 sulfur cluster binding	0.71	0.493988	5.17	3	55	51537
phosphorelay signal transduction system	0.69	0.503864	4.76	7	140	160
molybdopterin cofactor binding	0.64	0.524807	6.25	1	15	43546
RNA catabolic process	0.64	0.524807	6.25	1	15	6401
translation	0.62	0.535332	4.65	6	123	6412
transferase activity, transferring phosphorus-containing groups	0.60	0.550179	4.76	3	60	16772
carbohydrate catabolic process	0.56	0.573521	4.44	18	387	16052
hydrolase activity, hydrolyzing Oglycosyl compounds	0.55	0.574773	4.76	2	40	4553

electron transfer activity	0.55	0.574773	4.76	2	40	9055
phosphorylation	0.54	0.580676	4.42	13	281	16310
RNA catabolic process	0.53	0.586767	5.26	1	18	6401
enterobacterial common antigen metabolic process	0.53	0.586767	5.26	1	18	46378
enterobacterial common antigen biosynthetic process	0.53	0.586767	5.26	1	18	9246
oxidoreductase activity	0.53	0.587699	4.65	2	41	16491
macromolecule modification	0.53	0.589024	4.40	8	174	43412
intracellular	0.52	0.597005	4.37	10	219	5622
DNA replication	0.50	0.606651	4.35	4	88	6260
manganese ion binding	0.49	0.615244	4.30	4	89	30145
locomotion	0.47	0.62233	4.29	3	67	40011
taxis	0.47	0.62233	4.29	3	67	42330
peptidoglycan metabolic process	0.45	0.640672	4.55	1	21	270
oxidation-reduction process	0.43	0.650034	4.33	36	796	55114
polysaccharide biosynthetic process	0.42	0.657035	4.35	1	22	271
external encapsulating structure	0.41	0.66685	4.33	49	1082	30312
cell wall	0.41	0.66685	4.33	49	1082	5618
peptidoglycan-based cell wall	0.41	0.66685	4.33	49	1082	9274
DNA replication	0.40	0.668816	4.00	3	72	6260
DNA-dependent DNA replication	0.40	0.668816	4.00	3	72	6261
phospholipid metabolic process	0.40	0.670017	4.00	2	48	6644
phospholipid biosynthetic process	0.40	0.670017	4.00	2	48	8654
kinase activity	0.38	0.682025	4.11	12	280	16301
cellular modified amino acid biosynthetic process	0.37	0.687565	4.00	1	24	42398

coenzyme binding	0.37	0.687565	4.00	1	24	50662
amino acid transmembrane transport	0.36	0.694638	3.85	3	75	3333
aerobic respiration	0.35	0.702902	3.80	3	76	9060
phosphotransferase activity, alcohol group as acceptor	0.34	0.710798	3.70	2	52	16773
purine nucleobase metabolic process	0.33	0.715386	3.70	1	26	6144
sulfur compound metabolic process	0.32	0.7226	3.79	5	127	6790
NAD metabolic process	0.32	0.728357	3.57	1	27	19674
NAD biosynthetic process	0.32	0.728357	3.57	1	27	9435
cytosolic small ribosomal subunit	0.32	0.728357	3.57	1	27	22627
peptide biosynthetic process	0.31	0.736227	3.67	4	105	43043
peptide metabolic process	0.31	0.736227	3.67	4	105	6518
ribosome binding	0.30	0.740738	3.45	1	28	43022
DNA recombination	0.27	0.761542	3.54	4	109	6310
structural constituent of ribosome	0.27	0.764007	3.33	2	58	3735
structural molecule activity	0.27	0.764007	3.33	2	58	5198
lipid biosynthetic process	0.26	0.772003	3.28	2	59	8610
serine family amino acid metabolic process	0.26	0.774606	3.13	1	31	9069
serine family amino acid biosynthetic process	0.26	0.774606	3.13	1	31	9070
cytosolic large ribosomal subunit	0.26	0.774606	3.13	1	31	22625
structural constituent of ribosome	0.25	0.779762	3.23	2	60	3735
macromolecule biosynthetic process	0.24	0.785713	3.83	14	352	9059
response to abiotic stimulus	0.23	0.795936	3.36	4	115	9628
carbohydrate derivative biosynthetic process	0.23	0.798182	3.68	10	262	1901137

transferase activity, transferring acyl groups other than amino-acyl groups	0.22	0.804063	2.86	1	34	16747
polysaccharide metabolic process	0.21	0.811582	3.28	4	118	5976
extracellular region	0.21	0.813003	2.78	1	35	5576
negative regulation of cellular process	0.21	0.813003	2.78	1	35	48523
catalytic activity	0.20	0.821625	2.94	2	66	3824
translation	0.19	0.823784	3.09	3	94	6412
cellular polysaccharide biosynthetic process	0.19	0.823784	3.09	3	94	33692
cellular carbohydrate biosynthetic process	0.19	0.823784	3.09	3	94	34637
cellular polysaccharide metabolic process	0.19	0.823784	3.09	3	94	44264
cell wall organization	0.19	0.826237	3.20	4	121	71555
ribose phosphate biosynthetic process	0.19	0.827867	2.90	2	67	46390
purine ribonucleotide metabolic process	0.19	0.827867	2.90	2	67	9150
purine ribonucleotide biosynthetic process	0.19	0.827867	2.90	2	67	9152
ribonucleotide metabolic process	0.19	0.827867	2.90	2	67	9259
ribonucleotide biosynthetic process	0.19	0.827867	2.90	2	67	9260
transferase activity, transferring glycosyl groups	0.19	0.829069	3.06	3	95	16757
negative regulation of biological process	0.19	0.829681	2.63	1	37	48519
protein metabolic process	0.19	0.829777	3.52	9	247	19538
amide biosynthetic process	0.17	0.839619	3.23	5	150	43604
NADP binding	0.17	0.844877	2.50	1	39	50661

ncRNA metabolic process	0.17	0.844877	2.50	1	39	34660
amino acid activation	0.17	0.844877	2.50	1	39	43038
tRNA aminoacylation	0.17	0.844877	2.50	1	39	43039
tRNA metabolic process	0.17	0.844877	2.50	1	39	6399
tRNA aminoacylation for protein translation	0.17	0.844877	2.50	1	39	6418
response to stimulus	0.17	0.846841	3.56	12	325	50896
organelle inner membrane	0.16	0.850013	3.92	35	857	19866
organelle membrane	0.16	0.850013	3.92	35	857	31090
macromolecule metabolic process	0.16	0.853231	3.86	29	723	43170
protein modification process	0.16	0.853502	2.91	3	100	36211
cellular protein modification process	0.16	0.853502	2.91	3	100	6464
carbohydrate biosynthetic process	0.15	0.858854	3.13	5	155	16051
organelle part	0.13	0.875139	3.86	35	872	44422
cellular macromolecule biosynthetic process	0.13	0.877306	3.44	12	337	34645
cellular amide metabolic process	0.13	0.879361	3.01	5	161	43603
polysaccharide biosynthetic process	0.12	0.882577	2.73	3	107	271
carbohydrate derivative metabolic process	0.12	0.883115	3.37	11	315	1901135
monocarboxylic acid biosynthetic process	0.12	0.888177	2.13	1	46	72330
ribose phosphate metabolic process	0.11	0.89285	2.44	2	80	19693
cellular protein metabolic process	0.11	0.897951	3.00	6	194	44267
nicotinamide nucleotide biosynthetic process	0.09	0.911508	1.92	1	51	19359
pyridine nucleotide biosynthetic process	0.09	0.911508	1.92	1	51	19363

cellular macromolecule metabolic process	0.09	0.916825	3.50	20	552	44260
molecular_function	0.08	0.922496	2.61	4	149	3674
pyridine nucleotide metabolic process	0.08	0.923107	1.82	1	54	19362
nicotinamide nucleotide metabolic process	0.08	0.923107	1.82	1	54	46496
oxidoreduction coenzyme metabolic process	0.08	0.923107	1.82	1	54	6733
pyridoxal phosphate binding	0.08	0.92362	2.20	2	89	30170
protein folding	0.08	0.924815	2.60	4	150	6457
cell wall macromolecule metabolic process	0.08	0.926626	1.79	1	55	44036
cell wall macromolecule biosynthetic process	0.08	0.926626	1.79	1	55	44038
aminoglycan biosynthetic process	0.08	0.926626	1.79	1	55	6023
glycosaminoglycan biosynthetic process	0.08	0.926626	1.79	1	55	6024
cellular component macromolecule biosynthetic process	0.08	0.926626	1.79	1	55	70589
peptidoglycan biosynthetic process	0.08	0.926626	1.79	1	55	9252
amine catabolic process	0.08	0.926626	1.79	1	55	9310
cation transport	0.07	0.932919	2.36	3	124	6812
cellular lipid metabolic process	0.06	0.939293	2.06	2	95	44255
lipid metabolic process	0.06	0.939293	2.06	2	95	6629
pyridine-containing compound biosynthetic process	0.05	0.951883	1.54	1	64	72525
pyridine-containing compound metabolic process	0.04	0.958201	1.47	1	67	72524
transferase activity	0.04	0.958209	3.55	35	950	16740

transferase activity, transferring acyl groups	0.04	0.958856	1.87	2	105	16746
protein transport	0.04	0.960441	1.85	2	106	15031
DNA integration	0.04	0.963692	1.41	1	70	15074
cell division	0.03	0.966217	1.79	2	110	51301
peptidoglycan metabolic process	0.03	0.966947	1.37	1	72	270
glycosaminoglycan metabolic process	0.03	0.966947	1.37	1	72	30203
aminoglycan metabolic process	0.03	0.966947	1.37	1	72	6022
dephosphorylation	0.03	0.972611	1.30	1	76	16311
sulfur compound biosynthetic process	0.03	0.973868	1.28	1	77	44272
regulation of cellular process	0.03	0.974801	1.94	3	152	50794
regulation of biological process	0.02	0.981153	1.84	3	160	50789
membrane	0.02	0.983893	3.83	91	2283	16020
biological regulation	0.01	0.988774	1.69	3	174	65007
ion transport	0.01	0.990696	1.65	3	179	6811
cofactor biosynthetic process	0.01	0.99183	1.36	2	145	51188
cofactor metabolic process	0.01	0.992786	1.33	2	148	51186
flavin adenine dinucleotide binding	0.01	0.994483	0.90	1	110	50660
monocarboxylic acid metabolic process	0.00	0.996037	0.85	1	117	32787
coenzyme biosynthetic process	0.00	0.996872	0.81	1	122	9108
drug metabolic process	0.00	0.997253	1.16	2	171	17144
coenzyme metabolic process	0.00	0.997286	0.79	1	125	6732
localization	0.00	0.99911	1.24	3	239	51179
establishment of localization	0.00	0.99911	1.24	3	239	51234
transport	0.00	0.99911	1.24	3	239	6810
integral component of membrane	0.00	0.999729	3.20	60	1815	16021
protein binding	0.00	0.999915	2.58	29	1093	5515

Table S1f. Gene ontology of 1066 DEGs.

function	*Enrichment Score*	*Enrichment p-value*	*% genes in group that are present*	*# genes in list, in group*	*# genes not in list, in group*	*GO ID*
translation	107.12	3.00E-47	72.87	94	35	6412
cellular nitrogen compound metabolic process	104.12	6.02E-46	35.24	284	522	34641
cellular process	79.58	2.74E-35	25.00	503	1509	9987
cytoplasm	77.70	1.80E-34	28.53	352	882	5737
RNA binding	75.57	1.51E-33	52.76	105	94	3723
nitrogen compound metabolic process	75.56	1.53E-33	27.83	366	949	6807
cellular aromatic compound metabolic process	67.98	3.00E-30	33.54	215	426	6725
cellular biosynthetic process	64.91	6.48E-29	30.20	263	608	44249
nucleobase-containing compound metabolic process	63.99	1.62E-28	34.61	190	359	6139
organic substance biosynthetic process	63.98	1.63E-28	29.51	275	657	1901576
biosynthetic process	63.98	1.63E-28	29.51	275	657	9058
structural constituent of ribosome	62.39	8.02E-28	79.03	49	13	3735
organic cyclic compound metabolic process	62.16	1.01E-27	31.90	222	474	1901360
cellular metabolic process	61.40	2.16E-27	24.31	455	1417	44237
heterocycle metabolic process	61.37	2.22E-27	31.92	219	467	46483
macromolecule metabolic process	58.81	2.89E-26	30.72	231	521	43170
organonitrogen compound biosynthetic process	57.93	6.96E-26	32.04	206	437	1901566
translation	56.77	2.22E-25	62.89	61	36	6412
structural constituent of ribosome	56.26	3.69E-25	76.67	46	14	3735

structural molecule activity	56.26	3.69E-25	76.67	46	14	5198
cellular nitrogen compound biosynthetic process	51.23	5.63E-23	36.29	139	244	44271
nucleic acid metabolic process	51.07	6.63E-23	38.96	120	188	90304
peptide biosynthetic process	50.00	1.94E-22	56.88	62	47	43043
peptide metabolic process	50.00	1.94E-22	56.88	62	47	6518
protein binding	49.50	3.18E-22	26.47	297	825	5515
RNA metabolic process	48.88	5.91E-22	52.71	68	61	16070
biological_process	46.82	4.64E-21	20.99	622	2341	8150
primary metabolic process	46.53	6.18E-21	23.76	399	1280	44238
organonitrogen compound metabolic process	45.61	1.56E-20	27.10	258	694	1901564
organic substance metabolic process	44.44	5.03E-20	22.83	444	1501	71704
cellular macromolecule biosynthetic process	42.39	3.90E-19	35.24	123	226	34645
cytoplasm	42.06	5.40E-19	24.59	328	1006	5737
cytosolic large ribosomal subunit	41.36	1.09E-18	87.50	28	4	22625
macromolecule biosynthetic process	40.11	3.80E-18	34.15	125	241	9059
intracellular part	39.18	9.65E-18	21.98	468	1661	44424
metabolic process	38.76	1.46E-17	21.55	502	1827	8152
amide biosynthetic process	37.70	4.25E-17	44.52	69	86	43604
cellular_component	36.83	1.01E-16	20.90	551	2086	5575
cell part	36.38	1.59E-16	20.87	549	2081	44464
cellular amide metabolic process	33.36	3.25E-15	41.57	69	97	43603
protein folding	31.02	3.37E-14	64.00	32	18	6457
cellular macromolecule metabolic process	30.97	3.53E-14	28.15	161	411	44260
ncRNA metabolic process	30.74	4.49E-14	70.00	28	12	34660

amino acid activation	30.74	4.49E-14	70.00	28	12	43038
tRNA aminoacylation	30.74	4.49E-14	70.00	28	12	43039
tRNA metabolic process	30.74	4.49E-14	70.00	28	12	6399
tRNA aminoacylation for protein translation	30.74	4.49E-14	70.00	28	12	6418
molecular_function	28.70	3.44E-13	40.52	62	91	3674
cellular protein metabolic process	25.06	1.31E-11	35.50	71	129	44267
RNA modification	23.74	4.88E-11	50.00	35	35	9451
small molecule metabolic process	23.72	5.02E-11	24.54	199	612	44281
purine nucleotide metabolic process	23.13	8.99E-11	44.21	42	53	6163
purine nucleotide biosynthetic process	23.13	8.99E-11	44.21	42	53	6164
purine-containing compound biosynthetic process	23.13	8.99E-11	44.21	42	53	72522
purine-containing compound metabolic process	22.72	1.35E-10	43.75	42	54	72521
plasma membrane respiratory chain complex I	21.13	6.68E-10	83.33	15	3	45272
nucleotide binding	19.54	3.26E-09	23.74	189	607	166
intracellular	19.14	4.85E-09	31.44	72	157	5622
aromatic compound biosynthetic process	19.13	4.91E-09	30.38	79	181	19438
ribose phosphate metabolic process	17.01	4.10E-08	41.46	34	48	19693
organic cyclic compound biosynthetic process	16.97	4.28E-08	28.29	86	218	1901362
ATP biosynthetic process	16.56	6.45E-08	76.47	13	4	6754
protein metabolic process	16.51	6.78E-08	29.30	75	181	19538
ribose phosphate biosynthetic process	16.45	7.17E-08	43.48	30	39	46390

purine ribonucleotide metabolic process	16.45	7.17E-08	43.48	30	39	9150
purine ribonucleotide biosynthetic process	16.45	7.17E-08	43.48	30	39	9152
ribonucleotide metabolic process	16.45	7.17E-08	43.48	30	39	9259
ribonucleotide biosynthetic process	16.45	7.17E-08	43.48	30	39	9260
protein-containing complex	16.42	7.39E-08	55.56	20	16	32991
nucleoside phosphate metabolic process	16.42	7.40E-08	34.01	50	97	6753
nucleotide metabolic process	16.42	7.40E-08	34.01	50	97	9117
proton-transporting ATP synthase complex, catalytic core F(1)	16.38	7.67E-08	100.00	9	0	45261
heterocycle biosynthetic process	16.31	8.23E-08	28.23	83	211	18130
ATP binding	16.29	8.44E-08	23.35	170	558	5524
vitamin B6 metabolic process	16.22	9.02E-08	80.00	12	3	42816
vitamin B6 biosynthetic process	16.22	9.02E-08	80.00	12	3	42819
pyridoxine metabolic process	16.22	9.02E-08	80.00	12	3	8614
pyridoxine biosynthetic process	16.22	9.02E-08	80.00	12	3	8615
nucleoside phosphate biosynthetic process	16.13	9.86E-08	34.03	49	95	1901293
nucleotide biosynthetic process	16.13	9.86E-08	34.03	49	95	9165
nucleobase-containing small molecule metabolic process	16.03	1.09E-07	29.39	72	173	55086
plasma membrane ATP synthesis coupled proton transport	16.00	1.13E-07	84.62	11	2	42777
cytosolic small ribosomal subunit	15.92	1.22E-07	60.71	17	11	22627
cellular amino acid metabolic process	15.91	1.23E-07	27.14	92	247	6520
transferase activity	15.67	1.57E-07	22.03	217	768	16740

protein folding	14.77	3.85E-07	32.47	50	104	6457
phosphorus metabolic process	14.75	3.93E-07	27.72	79	206	6793
protein peptidyl-prolyl isomerization	14.45	5.32E-07	68.42	13	6	413
peptidyl-prolyl cis-trans isomerase activity	14.45	5.32E-07	68.42	13	6	3755
intracellular protein transmembrane transport	14.34	5.95E-07	83.33	10	2	65002
DNA replication	14.24	6.54E-07	40.00	30	45	6260
DNA-dependent DNA replication	14.24	6.54E-07	40.00	30	45	6261
small molecule biosynthetic process	13.63	1.21E-06	26.38	86	240	44283
organophosphate metabolic process	13.45	1.45E-06	28.27	67	170	19637
phosphate-containing compound metabolic process	13.45	1.45E-06	28.27	67	170	6796
nucleobase-containing compound biosynthetic process	13.36	1.58E-06	30.91	51	114	34654
energy coupled proton transport, down electrochemical gradient	12.75	2.89E-06	61.90	13	8	15985
ATP synthesis coupled proton transport	12.75	2.89E-06	61.90	13	8	15986
response to iron(III) ion	12.69	3.08E-06	81.82	9	2	10041
organophosphate biosynthetic process	12.55	3.55E-06	28.57	60	150	90407
translational elongation	11.93	6.58E-06	71.43	10	4	6414
rRNA processing	11.24	1.32E-05	37.68	26	43	6364
NADH dehydrogenase (quinone) activity	11.06	1.57E-05	80.00	8	2	50136
DNA metabolic process	11.05	1.59E-05	28.57	52	130	6259
ribosomal large subunit binding	10.99	1.69E-05	66.67	10	5	43023
cytoplasmic part	10.92	1.82E-05	100.00	6	0	44444
cytosolic part	10.92	1.82E-05	100.00	6	0	44445

DNA-templated transcription, termination	10.92	1.82E-05	100.00	6	0	6353
protein unfolding	10.81	2.02E-05	87.50	7	1	43335
response to antibiotic	10.81	2.03E-05	29.93	44	103	46677
Gram-negative-bacterium-type cell outer membrane assembly	10.73	2.19E-05	54.17	13	11	43165
alpha-amino acid metabolic process	10.69	2.27E-05	27.19	59	158	1901605
alpha-amino acid biosynthetic process	10.69	2.27E-05	27.19	59	158	1901607
cellular amino acid biosynthetic process	10.54	2.63E-05	27.06	59	159	8652
histidine biosynthetic process	10.17	3.84E-05	62.50	10	6	105
translation elongation factor activity	10.17	3.84E-05	62.50	10	6	3746
purine nucleotide biosynthetic process	9.77	5.71E-05	48.28	14	15	6164
ribosome binding	9.77	5.71E-05	48.28	14	15	43022
organic hydroxy compound biosynthetic process	9.50	7.47E-05	52.17	12	11	1901617
DNA replication	9.50	7.48E-05	32.61	30	62	6260
protein disulfide isomerase activity	9.12	0.000109573	85.71	6	1	3756
protein insertion into membrane	8.97	0.000126724	66.67	8	4	51205
histidine biosynthetic process	8.78	0.000153319	55.56	10	8	105
imidazole-containing compound metabolic process	8.78	0.000153319	55.56	10	8	52803
histidine metabolic process	8.78	0.000153319	55.56	10	8	6547
phosphotransferase activity, phosphate group as acceptor	8.41	0.000223481	70.00	7	3	16776
terpenoid biosynthetic process	8.41	0.000223481	70.00	7	3	16114
fatty acid biosynthetic process	8.30	0.000247676	41.67	15	21	6633
carboxylic acid metabolic process	8.29	0.00025107	22.11	109	384	19752

oxoacid metabolic process	8.29	0.00025107	22.11	109	384	43436
organic acid biosynthetic process	8.27	0.000256301	24.21	69	216	16053
carboxylic acid biosynthetic process	8.27	0.000256301	24.21	69	216	46394
enterobacterial common antigen metabolic process	8.19	0.00027678	52.63	10	9	46378
enterobacterial common antigen biosynthetic process	8.19	0.00027678	52.63	10	9	9246
protein refolding	8.17	0.000282586	61.54	8	5	42026
primosome complex	8.17	0.000282586	61.54	8	5	1990077
chromosomal part	8.17	0.000282586	61.54	8	5	44427
intracellular organelle part	8.17	0.000282586	61.54	8	5	44446
organic acid metabolic process	8.10	0.000303474	21.94	111	395	6082
protein disulfide oxidoreductase activity	8.01	0.000331503	46.15	12	14	15035
response to drug	7.93	0.000359249	29.81	31	73	42493
Bam protein complex	7.88	0.000377738	75.00	6	2	1990063
oxidoreductase activity, acting on NAD(P)H, quinone or similar compound as acceptor	7.88	0.000377738	75.00	6	2	16655
nucleobase-containing small molecule interconversion	7.86	0.000385366	30.77	28	63	15949
ATP metabolic process	7.83	0.000395949	37.78	17	28	46034
ATP biosynthetic process	7.83	0.000395949	37.78	17	28	6754
nucleoside monophosphate metabolic process	7.83	0.000395949	37.78	17	28	9123
nucleoside monophosphate biosynthetic process	7.83	0.000395949	37.78	17	28	9124

purine nucleoside monophosphate metabolic process	7.83	0.000395949	37.78	17	28	9126
purine nucleoside monophosphate biosynthetic process	7.83	0.000395949	37.78	17	28	9127
nucleoside triphosphate metabolic process	7.83	0.000395949	37.78	17	28	9141
nucleoside triphosphate biosynthetic process	7.83	0.000395949	37.78	17	28	9142
purine nucleoside triphosphate metabolic process	7.83	0.000395949	37.78	17	28	9144
purine nucleoside triphosphate biosynthetic process	7.83	0.000395949	37.78	17	28	9145
ribonucleoside monophosphate biosynthetic process	7.83	0.000395949	37.78	17	28	9156
ribonucleoside monophosphate metabolic process	7.83	0.000395949	37.78	17	28	9161
purine ribonucleoside monophosphate metabolic process	7.83	0.000395949	37.78	17	28	9167
purine ribonucleoside monophosphate biosynthetic process	7.83	0.000395949	37.78	17	28	9168
ribonucleoside triphosphate metabolic process	7.83	0.000395949	37.78	17	28	9199
ribonucleoside triphosphate biosynthetic process	7.83	0.000395949	37.78	17	28	9201
purine ribonucleoside triphosphate metabolic process	7.83	0.000395949	37.78	17	28	9205
purine ribonucleoside triphosphate biosynthetic process	7.83	0.000395949	37.78	17	28	9206
GTP binding	7.66	0.000469968	30.43	28	64	5525

macromolecule modification	7.50	0.000550745	25.82	47	135	43412
membrane part	7.48	0.000565723	57.14	8	6	44425
	7.27	0.000693346	100.00	4	0	32403
ATP metabolic process	7.27	0.000693346	100.00	4	0	46034
aspartate biosynthetic process	7.27	0.000693346	100.00	4	0	6532
glyoxylate reductase (NADP) activity	7.27	0.000693346	100.00	4	0	30267
chaperone complex	7.27	0.000693346	100.00	4	0	101031
chaperonin ATPase complex	7.27	0.000693346	100.00	4	0	16465
GroEL-GroES complex	7.27	0.000693346	100.00	4	0	1990220
DNA strand elongation involved in DNA replication	7.27	0.000693346	100.00	4	0	6271
response to chemical	7.21	0.000736664	27.21	37	99	42221
membrane protein complex	6.93	0.000977176	66.67	6	3	98796
cellular response to heat	6.93	0.000977176	66.67	6	3	34605
protein targeting	6.93	0.000977176	66.67	6	3	6605
DNA-dependent DNA replication	6.71	0.00121352	45.45	10	12	6261
tRNA modification	6.51	0.00149431	47.37	9	10	6400
rRNA base methylation	6.45	0.00157871	40.00	12	18	70475
carbohydrate derivative biosynthetic process	6.45	0.00158416	23.16	63	209	1901137
phospholipid binding	6.34	0.00176617	71.43	5	2	5543
transcription antitermination	6.33	0.0017868	50.00	8	8	31564
response to ionizing radiation	6.33	0.0017868	50.00	8	8	10212
proline metabolic process	6.20	0.00203334	53.85	7	6	6560
proline biosynthetic process	6.20	0.00203334	53.85	7	6	6561
drug metabolic process	6.16	0.00210529	24.86	43	130	17144
aromatic amino acid family metabolic process	6.12	0.00219921	34.88	15	28	9072

aromatic amino acid family biosynthetic process	6.12	0.00219921	34.88	15	28	9073
nucleoside bisphosphate metabolic process	6.06	0.00232952	45.00	9	11	33865
nucleoside bisphosphate biosynthetic process	6.06	0.00232952	45.00	9	11	33866
ribonucleoside bisphosphate metabolic process	6.06	0.00232952	45.00	9	11	33875
ribonucleoside bisphosphate biosynthetic process	6.06	0.00232952	45.00	9	11	34030
purine nucleoside bisphosphate metabolic process	6.06	0.00232952	45.00	9	11	34032
purine nucleoside bisphosphate biosynthetic process	6.06	0.00232952	45.00	9	11	34033
protein-DNA complex	5.84	0.00289912	47.06	8	9	32993
carbohydrate derivative metabolic process	5.84	0.00290206	22.09	72	254	1901135
NADP binding	5.83	0.00295212	35.00	14	26	50661
response to stimulus	5.81	0.00298997	21.96	74	263	50896
transferase activity, transferring glycosyl groups	5.80	0.00301683	27.55	27	71	16757
cell projection organization	5.80	0.00301757	80.00	4	1	30030
cell projection assembly	5.80	0.00301757	80.00	4	1	30031
pilus organization	5.80	0.00301757	80.00	4	1	43711
pilus assembly	5.80	0.00301757	80.00	4	1	9297
glutamine family amino acid metabolic process	5.68	0.00340039	29.58	21	50	9064
glutamine family amino acid biosynthetic process	5.68	0.00340039	29.58	21	50	9084

threonine metabolic process	5.65	0.00350096	50.00	7	7	6566
threonine biosynthetic process	5.65	0.00350096	50.00	7	7	9088
regulation of translational fidelity	5.65	0.00350096	50.00	7	7	6450
cell redox homeostasis	5.53	0.00396824	35.14	13	24	45454
3'-5'-exoribonuclease activity	5.52	0.00400296	54.55	6	5	175
regulation of translation	5.51	0.00404285	36.36	12	21	6417
tRNA methylthiolation	5.45	0.00427778	100.00	3	0	35600
macromolecule modification	5.45	0.00427778	100.00	3	0	43412
CTP biosynthetic process	5.45	0.00427778	100.00	3	0	6241
'de novo' CTP biosynthetic process	5.45	0.00427778	100.00	3	0	44210
glutamyl-tRNA aminoacylation	5.45	0.00427778	100.00	3	0	6424
cellular response to cold	5.45	0.00427778	100.00	3	0	70417
oxidoreductase activity, acting on the CH-OH group of donors, NAD or NADP as acceptor	5.32	0.00489923	33.33	14	28	16616
hexose biosynthetic process	5.28	0.00507719	40.91	9	13	19319
monosaccharide biosynthetic process	5.28	0.00507719	40.91	9	13	46364
gluconeogenesis	5.28	0.00507719	40.91	9	13	6094
rRNA methylation	5.23	0.00536441	38.46	10	16	31167
regulation of organelle assembly	4.97	0.0069153	50.00	6	6	1902115
regulation of bacterial-type flagellum assembly	4.97	0.0069153	50.00	6	6	1902208
enzyme-directed rRNA pseudouridine synthesis	4.97	0.0069153	50.00	6	6	455
ATP synthesis coupled electron transport	4.97	0.0069153	50.00	6	6	42773
ribosome biogenesis	4.94	0.00715949	39.13	9	14	42254
integral component of external side of plasma membrane	4.94	0.00715949	39.13	9	14	71575

lipopolysaccharide-transporting ATPase activity	4.84	0.00788693	66.67	4	2	15437
malonyl-CoA metabolic process	4.84	0.00788693	66.67	4	2	2001293
malonyl-CoA biosynthetic process	4.84	0.00788693	66.67	4	2	2001295
tRNA dihydrouridine synthase activity	4.84	0.00788693	66.67	4	2	17150
NADPH binding	4.84	0.00788693	66.67	4	2	70402
glyceraldehyde-3-phosphate dehydrogenase (NAD+) (phosphorylating) activity	4.84	0.00788693	66.67	4	2	4365
selenocysteine biosynthetic process	4.84	0.00788693	66.67	4	2	16260
glucan metabolic process	4.84	0.00788693	66.67	4	2	44042
cellular glucan metabolic process	4.84	0.00788693	66.67	4	2	6073
glucan biosynthetic process	4.84	0.00788693	66.67	4	2	9250
L-valine transmembrane transporter activity	4.84	0.00788693	66.67	4	2	5304
branched-chain amino acid transport	4.84	0.00788693	66.67	4	2	15803
valine transport	4.84	0.00788693	66.67	4	2	15829
L-valine transmembrane transport	4.84	0.00788693	66.67	4	2	1903785
ribosomal small subunit biogenesis	4.84	0.00788693	66.67	4	2	42274
L-serine biosynthetic process	4.84	0.00788693	66.67	4	2	6564
cellular response to antibiotic	4.83	0.00795902	55.56	5	4	71236
carbohydrate biosynthetic process	4.79	0.00828098	23.75	38	122	16051
RNA catabolic process	4.75	0.00866161	43.75	7	9	6401
pyridoxal phosphate binding	4.71	0.00896703	26.37	24	67	30170
serine family amino acid metabolic process	4.67	0.0094046	34.38	11	21	9069
serine family amino acid biosynthetic process	4.67	0.0094046	34.38	11	21	9070

iron-sulfur cluster assembly	4.67	0.0094046	34.38	11	21	16226
oxidoreductase activity, acting on NAD(P)H	4.65	0.00954518	40.00	8	12	16651
pyridine-containing compound metabolic process	4.60	0.0100699	27.94	19	49	72524
DNA recombination	4.33	0.013202	36.00	9	16	6310
pyridine-containing compound biosynthetic process	4.32	0.0132656	27.69	18	47	72525
coenzyme A metabolic process	4.28	0.0138103	50.00	5	5	15936
coenzyme A biosynthetic process	4.28	0.0138103	50.00	5	5	15937
dihydrofolate reductase activity	4.20	0.0150311	75.00	3	1	4146
polyamine-transporting ATPase activity	4.20	0.0150311	75.00	3	1	15417
tyrosine biosynthetic process	4.20	0.0150311	75.00	3	1	6571
lysyl-tRNA aminoacylation	4.20	0.0150311	75.00	3	1	6430
malate dehydrogenase (decarboxylating) (NAD+) activity	4.20	0.0150311	75.00	3	1	4471
cell cycle	4.20	0.0150311	75.00	3	1	7049
	4.20	0.0150311	75.00	3	1	15542
oligosaccharide metabolic process	4.18	0.0153103	32.35	11	23	9311
oligosaccharide biosynthetic process	4.18	0.0153103	32.35	11	23	9312
	4.13	0.016048	57.14	4	3	6461
positive regulation of translation	4.13	0.016048	57.14	4	3	45727
regulation of cell projection organization	4.09	0.016799	42.86	6	8	31344
regulation of organelle organization	4.09	0.016799	42.86	6	8	33043
regulation of cell projection assembly	4.09	0.016799	42.86	6	8	60491
one-carbon metabolic process	4.09	0.016799	42.86	6	8	6730

water-soluble vitamin biosynthetic process	4.05	0.0174664	27.42	17	45	42364
vitamin metabolic process	4.05	0.0174664	27.42	17	45	6766
water-soluble vitamin metabolic process	4.05	0.0174664	27.42	17	45	6767
vitamin biosynthetic process	4.05	0.0174664	27.42	17	45	9110
pseudouridine synthesis	4.02	0.0179205	38.89	7	11	1522
organic hydroxy compound metabolic process	3.98	0.0186111	28.30	15	38	1901615
cellular amino acid metabolic process	3.87	0.0208089	28.57	14	35	6520
ribosomal large subunit assembly	3.82	0.0219831	45.45	5	6	27
protein homooligomerization	3.82	0.0219831	45.45	5	6	51260
efflux transmembrane transporter activity	3.82	0.0219831	45.45	5	6	15562
polyamine biosynthetic process	3.80	0.0223755	33.33	9	18	6596
proton-transporting ATP synthase complex, coupling factor F(o)	3.57	0.0280157	50.00	4	4	45263
aminoacyl-tRNA editing activity	3.42	0.0327458	41.67	5	7	2161
protein catabolic process	3.42	0.0327458	41.67	5	7	30163
lipopolysaccharide binding	3.41	0.0330513	60.00	3	2	1530
translation initiation factor activity	3.41	0.0330513	60.00	3	2	3743
UMP salvage	3.41	0.0330513	60.00	3	2	44206
polyamine metabolic process	3.34	0.035383	31.03	9	20	6595
cofactor metabolic process	3.27	0.0380832	22.00	33	117	51186
leucine metabolic process	3.25	0.0388366	32.00	8	17	6551
branched-chain amino acid metabolic process	3.25	0.0388366	32.00	8	17	9081

branched-chain amino acid biosynthetic process	3.25	0.0388366	32.00	8	17	9082
leucine biosynthetic process	3.25	0.0388366	32.00	8	17	9098
drug transport	3.25	0.0388366	32.00	8	17	15893
DNA methylation on adenine	3.12	0.044061	44.44	4	5	32775
posttranscriptional regulation of gene expression	3.12	0.044061	44.44	4	5	10608
regulation of cellular protein metabolic process	3.12	0.044061	44.44	4	5	32268
regulation of cellular amide metabolic process	3.12	0.044061	44.44	4	5	34248
regulation of protein metabolic process	3.12	0.044061	44.44	4	5	51246
regulation of translation	3.12	0.044061	44.44	4	5	6417
lipooligosaccharide metabolic process	3.10	0.0449554	35.29	6	11	1901269
lipooligosaccharide biosynthetic process	3.10	0.0449554	35.29	6	11	1901271
membrane lipid biosynthetic process	3.10	0.0449554	35.29	6	11	46467
lipid A metabolic process	3.10	0.0449554	35.29	6	11	46493
membrane lipid metabolic process	3.10	0.0449554	35.29	6	11	6643
glycolipid metabolic process	3.10	0.0449554	35.29	6	11	6664
lipid A biosynthetic process	3.10	0.0449554	35.29	6	11	9245
glycolipid biosynthetic process	3.10	0.0449554	35.29	6	11	9247
aerobic respiration	3.08	0.0461047	24.05	19	60	9060
DNA topological change	3.07	0.0462746	38.46	5	8	6265
cofactor biosynthetic process	3.07	0.046277	21.77	32	115	51188
nucleic acid binding	2.96	0.0519441	21.18	36	134	3676
homoserine biosynthetic process	2.84	0.0582157	50.00	3	3	9090
homoserine metabolic process	2.84	0.0582157	50.00	3	3	9092

D-alanine biosynthetic process	2.84	0.0582157	50.00	3	3	30632
translational initiation	2.84	0.0582157	50.00	3	3	6413
pyrimidine nucleotide biosynthetic process	2.84	0.0584728	33.33	6	12	6221
response to osmotic stress	2.83	0.0592495	26.09	12	34	6970
cell wall macromolecule metabolic process	2.81	0.0602314	25.00	14	42	44036
cell wall macromolecule biosynthetic process	2.81	0.0602314	25.00	14	42	44038
aminoglycan biosynthetic process	2.81	0.0602314	25.00	14	42	6023
glycosaminoglycan biosynthetic process	2.81	0.0602314	25.00	14	42	6024
cellular component macromolecule biosynthetic process	2.81	0.0602314	25.00	14	42	70589
peptidoglycan biosynthetic process	2.81	0.0602314	25.00	14	42	9252
pyrimidine nucleobase metabolic process	2.77	0.0626499	35.71	5	9	6206
protein serine/threonine kinase activity	2.75	0.0642282	40.00	4	6	4674
thioester metabolic process	2.75	0.0642282	40.00	4	6	35383
thioester biosynthetic process	2.75	0.0642282	40.00	4	6	35384
acyl-CoA metabolic process	2.75	0.0642282	40.00	4	6	6637
acyl-CoA biosynthetic process	2.75	0.0642282	40.00	4	6	71616
L-serine metabolic process	2.75	0.0642282	40.00	4	6	6563
L-serine biosynthetic process	2.75	0.0642282	40.00	4	6	6564
cellular biogenic amine biosynthetic process	2.70	0.0674714	26.19	11	31	42401
amine biosynthetic process	2.70	0.0674714	26.19	11	31	9309
phosphopyruvate hydratase complex	2.65	0.0705615	66.67	2	1	15

phosphopyruvate hydratase activity	2.65	0.0705615	66.67	2	1	4634
transcription antitermination factor activity, DNA binding	2.65	0.0705615	66.67	2	1	1073
integral component of membrane	2.65	0.0705615	66.67	2	1	16021
imidazoleglycerol-phosphate synthase activity	2.65	0.0705615	66.67	2	1	107
	2.65	0.0705615	66.67	2	1	42967
pyrophosphatase activity	2.65	0.0705615	66.67	2	1	16462
lactose transport	2.65	0.0705615	66.67	2	1	15767
dUTP diphosphatase activity	2.65	0.0705615	66.67	2	1	4170
rRNA (guanine-N7-)-methyltransferase activity	2.65	0.0705615	66.67	2	1	70043
rRNA (guanine-N7)-methylation	2.65	0.0705615	66.67	2	1	70476
L-serine metabolic process	2.65	0.0705615	66.67	2	1	6563
protein maturation	2.63	0.0717282	28.57	8	20	51604
dicarboxylic acid metabolic process	2.60	0.0739137	23.29	17	56	43648
glyceraldehyde-3-phosphate metabolic process	2.60	0.0741573	31.58	6	13	19682
RNA catabolic process	2.60	0.0741573	31.58	6	13	6401
monovalent inorganic cation transport	2.55	0.077961	24.53	13	40	15672
proton transmembrane transport	2.55	0.077961	24.53	13	40	1902600
fatty acid metabolic process	2.55	0.0782167	25.00	12	36	6631
cellular macromolecule catabolic process	2.43	0.0881283	24.07	13	41	44265
plasmid maintenance	2.43	0.0883682	36.36	4	7	6276
cellular amine metabolic process	2.41	0.0893931	25.00	11	33	44106
cellular biogenic amine metabolic process	2.41	0.0893931	25.00	11	33	6576

glucose metabolic process	2.41	0.0893931	25.00	11	33	6006
tyrosine metabolic process	2.41	0.0898416	42.86	3	4	6570
tyrosine biosynthetic process	2.41	0.0898416	42.86	3	4	6571
alanine metabolic process	2.41	0.0898416	42.86	3	4	6522
alanine biosynthetic process	2.41	0.0898416	42.86	3	4	6523
pyruvate family amino acid metabolic process	2.41	0.0898416	42.86	3	4	9078
pyruvate family amino acid biosynthetic process	2.41	0.0898416	42.86	3	4	9079
sodium ion binding	2.41	0.0898416	42.86	3	4	31402
protein transport	2.31	0.0990842	21.30	23	85	15031
response to abiotic stimulus	2.30	0.100159	21.01	25	94	9628
dicarboxylic acid biosynthetic process	2.28	0.101805	24.44	11	34	43650
oxidoreductase activity, acting on the aldehyde or oxo group of donors, NAD or NADP as acceptor	2.28	0.102224	25.71	9	26	16620
O antigen metabolic process	2.27	0.103811	31.25	5	11	46402
O antigen biosynthetic process	2.27	0.103811	31.25	5	11	9243

ribosomal small subunit binding	2.27	0.103811	31.25	5	11	43024
aromatic compound catabolic process	2.21	0.110099	22.08	17	60	19439
nucleobase-containing compound catabolic process	2.21	0.110099	22.08	17	60	34655
iron ion binding	2.19	0.111774	20.87	24	91	5506
chorismate metabolic process	2.15	0.116179	33.33	4	8	46417
chorismate biosynthetic process	2.15	0.116179	33.33	4	8	9423
macromolecule catabolic process	2.15	0.116758	22.39	15	52	9057
arginine metabolic process	2.14	0.117588	25.81	8	23	6525
arginine biosynthetic process	2.14	0.117588	25.81	8	23	6526

peptidoglycan metabolic process	2.08	0.124509	21.92	16	57	270
glycosaminoglycan metabolic process	2.08	0.124509	21.92	16	57	30203
aminoglycan metabolic process	2.08	0.124509	21.92	16	57	6022
[2Fe-2S] cluster assembly	2.07	0.126092	50.00	2	2	44571
	2.07	0.126092	50.00	2	2	1902765
protein maturation by protein folding	2.07	0.126092	50.00	2	2	22417
extrinsic component of periplasmic side of plasma membrane	2.07	0.126092	50.00	2	2	31236
aspartate-semialdehyde dehydrogenase activity	2.07	0.126092	50.00	2	2	4073
positive regulation of GTPase activity	2.07	0.126092	50.00	2	2	43547
uracil transmembrane transporter activity	2.07	0.126092	50.00	2	2	15210
pyrimidine nucleobase transport	2.07	0.126092	50.00	2	2	15855
uracil transport	2.07	0.126092	50.00	2	2	15857
uracil transmembrane transport	2.07	0.126092	50.00	2	2	1903791
pyrimidine nucleobase transmembrane transport	2.07	0.126092	50.00	2	2	1904082
pyrimidine-containing compound transmembrane transport	2.07	0.126092	50.00	2	2	72531
sialic acid transmembrane transporter activity	2.07	0.126092	50.00	2	2	15136
phosphate ion binding	2.07	0.126092	50.00	2	2	42301
carbon dioxide binding	2.07	0.126092	50.00	2	2	1902670
drug binding	2.07	0.126092	50.00	2	2	8144
cellular response to zinc ion starvation	2.07	0.126092	50.00	2	2	34224
glutamine catabolic process	2.07	0.126092	50.00	2	2	6543

negative regulation of fatty acid biosynthetic process	2.07	0.126092	50.00	2	2	45717
rRNA (cytosine) methyltransferase activity	2.07	0.126092	50.00	2	2	16434
selenocysteine incorporation	2.07	0.126092	50.00	2	2	1514
rRNA (cytosine-2'-O-)methyltransferase activity	2.07	0.126092	50.00	2	2	70677
tRNA (uracil) methyltransferase activity	2.07	0.126092	50.00	2	2	16300
transcription factor activity, bacterialtype RNA polymerase proximal promoter sequence-specific DNA binding	2.07	0.126092	50.00	2	2	1131
triphosphatase activity	2.07	0.126092	50.00	2	2	50355
chloride ion binding	2.07	0.126092	50.00	2	2	31404
aminoacyl-tRNA hydrolase activity	2.07	0.126092	50.00	2	2	4045
RNA helicase activity	2.07	0.126092	50.00	2	2	3724
L-alanine:2-oxoglutarate aminotransferase activity	2.07	0.126092	50.00	2	2	4021
beta-glucan biosynthetic process	2.07	0.126092	50.00	2	2	51274
protein-disulfide reductase activity	2.07	0.126092	50.00	2	2	47134
response to reactive oxygen species	2.07	0.126092	50.00	2	2	302
P-P-bond-hydrolysis-driven protein transmembrane transporter activity	2.07	0.126092	50.00	2	2	15450
ribulose-phosphate 3-epimerase activity	2.07	0.126092	50.00	2	2	4750
cellular carbohydrate metabolic process	2.07	0.126092	50.00	2	2	44262

bacterial-type RNA polymerase transcriptional activator activity, sequence-specific DNA binding	2.07	0.126092	50.00	2	2	1216
biotin carboxyl carrier protein biosynthetic process	2.07	0.126092	50.00	2	2	42966
peptide biosynthetic process	2.07	0.126092	50.00	2	2	43043
lipid-transporting ATPase activity	2.07	0.126092	50.00	2	2	34040
protein binding, bridging	2.07	0.126092	50.00	2	2	30674
polyamine transmembrane transport	2.06	0.126937	37.50	3	5	1902047
erythrose 4-phosphate/phosphoenolpyruvate family amino acid metabolic process	2.06	0.126937	37.50	3	5	1902221
erythrose 4-phosphate/phosphoenolpyruvate family amino acid biosynthetic process	2.06	0.126937	37.50	3	5	1902223
L-phenylalanine metabolic process	2.06	0.126937	37.50	3	5	6558
L-phenylalanine biosynthetic process	2.06	0.126937	37.50	3	5	9094
translational attenuation	2.06	0.126937	37.50	3	5	9386
drug transmembrane transport	2.01	0.133842	27.27	6	16	6855
ubiquinone biosynthetic process	2.01	0.133842	27.27	6	16	6744
defense response to bacterium	2.01	0.133842	27.27	6	16	42742
response to temperature stimulus	2.01	0.134317	24.32	9	28	9266
2 iron, 2 sulfur cluster binding	1.99	0.136664	22.41	13	45	51537
pentose-phosphate shunt, non-oxidative branch	1.92	0.147248	30.77	4	9	9052
enzyme regulator activity	1.92	0.147248	30.77	4	9	30234
ATP-dependent RNA helicase activity	1.92	0.147248	30.77	4	9	4004

transferase activity, transferring alkyl or aryl (other than methyl) groups	1.92	0.147248	30.77	4	9	16765
inorganic cation transmembrane transport	1.89	0.150721	22.03	13	46	98662
locomotion	1.87	0.153961	21.43	15	55	40011
taxis	1.87	0.153961	21.43	15	55	42330
glutamine metabolic process	1.85	0.157608	26.09	6	17	6541
inorganic ion transmembrane transport	1.82	0.16139	21.05	16	60	98660
neurotransmitter biosynthetic process	1.78	0.168375	33.33	3	6	42136
glycine metabolic process	1.78	0.168375	33.33	3	6	6544
glycine biosynthetic process	1.78	0.168375	33.33	3	6	6545
pyrimidine-containing compound metabolic process	1.77	0.171002	23.08	9	30	72527
pyrimidine-containing compound biosynthetic process	1.77	0.171002	23.08	9	30	72528
phospholipid metabolic process	1.73	0.177883	22.00	11	39	6644
phospholipid biosynthetic process	1.73	0.177883	22.00	11	39	8654
hexose metabolic process	1.73	0.177883	22.00	11	39	19318
monosaccharide metabolic process	1.73	0.177883	22.00	11	39	5996
metalloendopeptidase activity	1.72	0.179923	24.14	7	22	4222
RNA methylation	1.71	0.181088	28.57	4	10	1510
glutamate biosynthetic process	1.71	0.181088	28.57	4	10	6537
flavin adenine dinucleotide binding	1.70	0.182542	19.82	22	89	50660
cation transmembrane transport	1.67	0.187343	20.22	18	71	98655
plasmid recombination	1.67	0.188119	40.00	2	3	42150
intrinsic component of membrane	1.67	0.188119	40.00	2	3	31224
single-stranded RNA binding	1.67	0.188119	40.00	2	3	3727
cyanate metabolic process	1.67	0.188119	40.00	2	3	9439
cyanate catabolic process	1.67	0.188119	40.00	2	3	9440

regulation of isomerase activity	1.67	0.188119	40.00	2	3	10911
regulation of DNA topoisomerase (ATP-hydrolyzing) activity	1.67	0.188119	40.00	2	3	2000371
negative regulation of DNA topoisomerase (ATP-hydrolyzing) activity	1.67	0.188119	40.00	2	3	2000372
negative regulation of ATPase activity	1.67	0.188119	40.00	2	3	32780
negative regulation of catalytic activity	1.67	0.188119	40.00	2	3	43086
regulation of ATPase activity	1.67	0.188119	40.00	2	3	43462
regulation of catalytic activity	1.67	0.188119	40.00	2	3	50790
regulation of hydrolase activity	1.67	0.188119	40.00	2	3	51336
negative regulation of hydrolase activity	1.67	0.188119	40.00	2	3	51346
double-stranded RNA binding	1.67	0.188119	40.00	2	3	3725
peptide binding	1.67	0.188119	40.00	2	3	42277
transcription antitermination factor activity, RNA binding	1.67	0.188119	40.00	2	3	1072
proline biosynthetic process	1.67	0.188119	40.00	2	3	6561
purine nucleobase transmembrane transporter activity	1.67	0.188119	40.00	2	3	5345
adenine transmembrane transporter activity	1.67	0.188119	40.00	2	3	15207
glycerol ether metabolic process	1.67	0.188119	40.00	2	3	6662
S-adenosylmethionine metabolic process	1.67	0.188119	40.00	2	3	46500
S-adenosylmethionine biosynthetic process	1.67	0.188119	40.00	2	3	6556

succinate-semialdehyde dehydrogenase (NAD+) activity	1.67	0.188119	40.00	2	3	4777
response to nickel cation	1.67	0.188119	40.00	2	3	10045
heat shock protein binding	1.67	0.188119	40.00	2	3	31072
aspartate family amino acid metabolic process	1.63	0.196731	20.55	15	58	9066
aspartate family amino acid biosynthetic process	1.63	0.196731	20.55	15	58	9067
catalytic activity	1.58	0.205084	20.59	14	54	3824
liposaccharide metabolic process	1.56	0.209983	24.00	6	19	1903509
response to nutrient levels	1.56	0.209983	24.00	6	19	31667
response to starvation	1.56	0.209983	24.00	6	19	42594
aromatic amino acid family biosynthetic process, prephenate pathway	1.55	0.21301	30.00	3	7	9095
response to zinc ion	1.55	0.21301	30.00	3	7	10043
catalytic activity, acting on a protein	1.55	0.21301	30.00	3	7	140096
peptidase activity	1.55	0.21301	30.00	3	7	8233
mRNA catabolic process	1.54	0.214866	25.00	5	15	6402
pyrimidine nucleotide metabolic process	1.54	0.214866	25.00	5	15	6220
pyrimidine nucleotide biosynthetic process	1.54	0.214866	25.00	5	15	6221
neutral amino acid transport	1.53	0.21717	26.67	4	11	15804
regulation of cellular component biogenesis	1.51	0.220333	22.22	8	28	44087
endopeptidase activity	1.47	0.229182	22.58	7	24	4175
sulfur compound metabolic process	1.47	0.229535	18.94	25	107	6790

electron transfer activity	1.45	0.233483	21.43	9	33	9055
response to stress	1.43	0.23964	18.38	34	151	6950
lipopolysaccharide core region metabolic process	1.40	0.247036	23.81	5	16	46401
lipopolysaccharide core region biosynthetic process	1.40	0.247036	23.81	5	16	9244
cysteine desulfurase activity	1.37	0.253071	33.33	2	4	31071
glycine betaine transport	1.37	0.253071	33.33	2	4	31460
response to organonitrogen compound	1.37	0.253071	33.33	2	4	10243
RNA metabolic process	1.37	0.253071	33.33	2	4	16070
negative regulation of single-species biofilm formation on inanimate substrate	1.37	0.253071	33.33	2	4	1900232
negative regulation of organelle assembly	1.37	0.253071	33.33	2	4	1902116
negative regulation of bacterial-type flagellum assembly	1.37	0.253071	33.33	2	4	1902209
negative regulation of cell projection organization	1.37	0.253071	33.33	2	4	31345
5-phosphoribose 1-diphosphate biosynthetic process	1.37	0.253071	33.33	2	4	6015
cell wall macromolecule metabolic process	1.37	0.253071	33.33	2	4	44036
IMP salvage	1.37	0.253071	33.33	2	4	32264
tRNA (uracil-2'-O-)-methyltransferase activity	1.37	0.253071	33.33	2	4	52665
tRNA (cytosine-2'-O-)methyltransferase activity	1.37	0.253071	33.33	2	4	52666

response to hypochlorite	1.37	0.253071	33.33	2	4	1901530
response to reactive oxygen species	1.37	0.253071	33.33	2	4	302
response to oxidative stress	1.37	0.253071	33.33	2	4	6979
3-oxoacyl-[acyl-carrier-protein] synthase activity	1.37	0.253071	33.33	2	4	4315
threonine metabolic process	1.37	0.253071	33.33	2	4	6566
phosphorylase activity	1.37	0.253071	33.33	2	4	4645
hydroxymethyl-, formyl- and related transferase activity	1.37	0.253071	33.33	2	4	16742
oxidized pyrimidine nucleobase lesion DNA N-glycosylase activity	1.37	0.253071	33.33	2	4	703
alanine biosynthetic process	1.37	0.253071	33.33	2	4	6523
carbohydrate biosynthetic process	1.37	0.253071	33.33	2	4	16051
oxidoreductase activity, acting on other nitrogenous compounds as donors	1.37	0.253071	33.33	2	4	16661
lysophospholipase activity	1.37	0.253071	33.33	2	4	4622
cellular carbohydrate catabolic process	1.37	0.253071	33.33	2	4	44275
Entner-Doudoroff pathway	1.37	0.253071	33.33	2	4	61678
Entner-Doudoroff pathway through 6phosphogluconate	1.37	0.253071	33.33	2	4	9255
hydroxyacylglutathione hydrolase activity	1.37	0.253071	33.33	2	4	4416
organic cyclic compound catabolic process	1.37	0.254705	19.32	17	71	1901361
heterocycle catabolic process	1.37	0.254705	19.32	17	71	46700
bacteriocin transport	1.37	0.254957	25.00	4	12	43213
oxidoreductase activity	1.36	0.255955	20.93	9	34	16491
arginine catabolic process	1.35	0.25975	27.27	3	8	6527

ATPase activity, coupled	1.35	0.25975	27.27	3	8	42623
regulation of neurotransmitter levels	1.35	0.25975	27.27	3	8	1505
neurotransmitter metabolic process	1.35	0.25975	27.27	3	8	42133
regulation of biological quality	1.35	0.25975	27.27	3	8	65008
aspartate metabolic process	1.35	0.25975	27.27	3	8	6531
aspartate biosynthetic process	1.35	0.25975	27.27	3	8	6532
cellular carbohydrate metabolic process	1.32	0.267184	18.52	25	110	44262
regulation of cellular component organization	1.31	0.268581	21.05	8	30	51128
sulfur compound biosynthetic process	1.28	0.278472	19.23	15	63	44272
peptidoglycan metabolic process	1.27	0.280328	22.73	5	17	270
cellular nitrogen compound catabolic process	1.23	0.293532	18.63	19	83	44270
diaminopimelate metabolic process	1.21	0.297521	21.43	6	22	46451
lysine metabolic process	1.21	0.297521	21.43	6	22	6553
lysine biosynthetic process	1.21	0.297521	21.43	6	22	9085
lysine biosynthetic process via diaminopimelate	1.21	0.297521	21.43	6	22	9089
cellular polysaccharide biosynthetic process	1.18	0.306479	18.56	18	79	33692
cellular carbohydrate biosynthetic process	1.18	0.306479	18.56	18	79	34637
cellular polysaccharide metabolic process	1.18	0.306479	18.56	18	79	44264
glutamine metabolic process	1.18	0.307607	25.00	3	9	6541
glutamine biosynthetic process	1.18	0.307607	25.00	3	9	6542
translational termination	1.18	0.307607	25.00	3	9	6415
hydrolase activity	1.16	0.314417	21.74	5	18	16787

cation transport	1.15	0.317642	18.11	23	104	6812
catalytic complex	1.14	0.318363	28.57	2	5	1902494
periplasmic side of cell outer membrane	1.14	0.318363	28.57	2	5	31241
cellular response to cell envelope stress	1.14	0.318363	28.57	2	5	36460
binding	1.14	0.318363	28.57	2	5	5488
negative regulation of molecular function	1.14	0.318363	28.57	2	5	44092
regulation of molecular function	1.14	0.318363	28.57	2	5	65009
protein tetramerization	1.14	0.318363	28.57	2	5	51262
transcription initiation from bacterialtype RNA polymerase promoter	1.14	0.318363	28.57	2	5	1123
protein quality control for misfolded or incompletely synthesized proteins	1.14	0.318363	28.57	2	5	6515
intracellular membrane-bounded organelle	1.14	0.318363	28.57	2	5	43231
L-serine catabolic process	1.14	0.318363	28.57	2	5	6565
response to inorganic substance	1.11	0.328199	20.69	6	23	10035
glyoxylate metabolic process	1.10	0.33355	22.22	4	14	46487
transferase activity, transferring acyl groups other than amino-acyl groups	1.08	0.338117	20.00	7	28	16747
periplasmic space	1.04	0.351794	17.54	30	141	42597
polyamine transport	1.03	0.355715	23.08	3	10	15846
RNA secondary structure unwinding	1.03	0.355715	23.08	3	10	10501
ribonuclease activity	1.03	0.355715	23.08	3	10	4540
arginine biosynthetic process	0.99	0.37339	21.05	4	15	6526
diguanylate cyclase activity	0.99	0.37339	21.05	4	15	52621

thiamine-containing compound metabolic process	0.99	0.37339	21.05	4	15	42723
thiamine-containing compound biosynthetic process	0.99	0.37339	21.05	4	15	42724
thiamine metabolic process	0.99	0.37339	21.05	4	15	6772
thiamine biosynthetic process	0.99	0.37339	21.05	4	15	9228
nucleobase transport	0.96	0.382171	25.00	2	6	15851
negative regulation of organelle organization	0.96	0.382171	25.00	2	6	10639
antibiotic transport	0.96	0.382171	25.00	2	6	42891
ribokinase activity	0.96	0.382171	25.00	2	6	4747
regulation of cell division	0.96	0.382171	25.00	2	6	51302
DNA replication initiation	0.96	0.382171	25.00	2	6	6270
lipoprotein metabolic process	0.96	0.382171	25.00	2	6	42157
lipoprotein biosynthetic process	0.96	0.382171	25.00	2	6	42158
regulation of anion transmembrane transport	0.96	0.382171	25.00	2	6	1903959
regulation of anion transport	0.96	0.382171	25.00	2	6	44070
coenzyme binding	0.96	0.383738	20.00	5	20	50662
coenzyme metabolic process	0.94	0.391354	17.46	22	104	6732
DNA catabolic process	0.93	0.395128	18.92	7	30	6308
sulfate transmembrane transport	0.91	0.40334	21.43	3	11	1902358
sulfate transport	0.91	0.40334	21.43	3	11	8272
cellular response to phosphate starvation	0.91	0.40334	21.43	3	11	16036
lipid biosynthetic process	0.91	0.404165	18.03	11	50	8610
glycine biosynthetic process	0.89	0.412537	33.33	1	2	6545
glucose 6-phosphate metabolic process	0.89	0.412537	33.33	1	2	51156

NADP metabolic process	0.89	0.412537	33.33	1	2	6739
pentose-phosphate shunt, oxidative branch	0.89	0.412537	33.33	1	2	9051
response to amino acid	0.89	0.412537	33.33	1	2	43200
GMP biosynthetic process	0.89	0.412537	33.33	1	2	6177
glutamate-ammonia ligase activity	0.89	0.412537	33.33	1	2	4356
glutamine biosynthetic process	0.89	0.412537	33.33	1	2	6542
antibiotic catabolic process	0.89	0.412537	33.33	1	2	17001
glucosamine-6-phosphate deaminase activity	0.89	0.412537	33.33	1	2	4342
adenylate kinase activity	0.89	0.412537	33.33	1	2	4017
amidase activity	0.89	0.412537	33.33	1	2	4040
3-hydroxyoctanoyl-[acyl-carrierprotein] dehydratase activity	0.89	0.412537	33.33	1	2	47451
cell envelope Sec protein transport complex	0.89	0.412537	33.33	1	2	31522
cellular response to magnesium starvation	0.89	0.412537	33.33	1	2	10350
adenosine deaminase activity	0.89	0.412537	33.33	1	2	4000
potassium channel activity	0.89	0.412537	33.33	1	2	5267
positive regulation of oxidoreductase activity	0.89	0.412537	33.33	1	2	51353
transcriptional repressor activity, bacterial-type RNA polymerase proximal promoter sequence-specific DNA binding	0.89	0.412537	33.33	1	2	1141
cellular aldehyde metabolic process	0.86	0.425008	18.18	8	36	6081
coenzyme biosynthetic process	0.82	0.438802	17.07	21	102	9108

inorganic phosphate transmembrane transporter activity	0.81	0.443255	22.22	2	7	5315
negative regulation of single-species biofilm formation	0.81	0.443255	22.22	2	7	1900191
negative regulation of multi-organism process	0.81	0.443255	22.22	2	7	43901
DNA methylation	0.81	0.443255	22.22	2	7	6306
methylation	0.81	0.443255	22.22	2	7	32259
macromolecule methylation	0.81	0.443255	22.22	2	7	43414
DNA methylation or demethylation	0.81	0.443255	22.22	2	7	44728
DNA modification	0.81	0.443255	22.22	2	7	6304
DNA alkylation	0.81	0.443255	22.22	2	7	6305
DNA methylation	0.81	0.443255	22.22	2	7	6306
glyoxylate cycle	0.81	0.443255	22.22	2	7	6097
ATPase activity, coupled to transmembrane movement of ions, phosphorylative mechanism	0.81	0.443255	22.22	2	7	15662
active ion transmembrane transporter activity	0.81	0.443255	22.22	2	7	22853
ATPase coupled ion transmembrane transporter activity	0.81	0.443255	22.22	2	7	42625
drug:proton antiporter activity	0.81	0.443255	22.22	2	7	15307
aldehyde catabolic process	0.81	0.443255	22.22	2	7	46185
glyoxylate catabolic process	0.81	0.443255	22.22	2	7	9436
ATP-dependent peptidase activity	0.81	0.443255	22.22	2	7	4176
manganese ion binding	0.81	0.44376	17.20	16	77	30145
cellular component assembly	0.80	0.449714	17.54	10	47	22607
glutamate metabolic process	0.80	0.449883	20.00	3	12	6536
glutamate biosynthetic process	0.80	0.449883	20.00	3	12	6537

ion transmembrane transport	0.73	0.480505	16.67	24	120	34220
nickel cation binding	0.73	0.483162	17.65	6	28	16151
oxidation-reduction process	0.73	0.483222	16.35	136	696	55114
'de novo' pyrimidine nucleobase biosynthetic process	0.70	0.494866	18.75	3	13	6207
regulation of single-species biofilm formation on inanimate substrate	0.69	0.500817	20.00	2	8	1900231
proteolysis	0.69	0.500817	20.00	2	8	6508
D-ribose metabolic process	0.69	0.500817	20.00	2	8	6014
response to radiation	0.69	0.500817	20.00	2	8	9314
enzyme-directed rRNA 2'-Omethylation	0.69	0.500817	20.00	2	8	453
alcohol dehydrogenase (NAD) activity	0.69	0.500817	20.00	2	8	4022
mismatch repair complex	0.68	0.508018	25.00	1	3	32300
alkanesulfonate catabolic process	0.68	0.508018	25.00	1	3	46306
glycine catabolic process	0.68	0.508018	25.00	1	3	6546
negative regulation of protein catabolic process	0.68	0.508018	25.00	1	3	42177
proteolysis involved in cellular protein catabolic process	0.68	0.508018	25.00	1	3	51603
glutamate-cysteine ligase activity	0.68	0.508018	25.00	1	3	4357
glutathione metabolic process	0.68	0.508018	25.00	1	3	6749
glutathione biosynthetic process	0.68	0.508018	25.00	1	3	6750
tRNA thio-modification	0.68	0.508018	25.00	1	3	34227
signal transduction	0.67	0.510968	16.67	12	60	7165
pyruvate metabolic process	0.67	0.513171	17.14	6	29	6090
cellular component organization	0.66	0.519006	16.67	10	50	16043

cellular component organization or biogenesis	0.66	0.519006	16.67	10	50	71840
external encapsulating structure part	0.65	0.520845	16.39	20	102	44462
polysaccharide metabolic process	0.65	0.520845	16.39	20	102	5976
polysaccharide biosynthetic process	0.64	0.527142	16.36	18	92	271
carbohydrate derivative transport	0.64	0.527259	17.39	4	19	1901264
glucose metabolic process	0.64	0.527259	17.39	4	19	6006
pyridine nucleotide metabolic process	0.60	0.547371	16.36	9	46	19362
nicotinamide nucleotide metabolic process	0.60	0.547371	16.36	9	46	46496
oxidoreduction coenzyme metabolic process	0.60	0.547371	16.36	9	46	6733
localization	0.60	0.550285	16.12	39	203	51179
establishment of localization	0.60	0.550285	16.12	39	203	51234
transport	0.60	0.550285	16.12	39	203	6810
serine-type endopeptidase activity	0.60	0.55143	16.67	5	25	4252
organophosphate catabolic process	0.60	0.55143	16.67	5	25	46434
peptide catabolic process	0.59	0.554387	18.18	2	9	43171
recombinase activity	0.59	0.554387	18.18	2	9	150
ADP binding	0.59	0.554387	18.18	2	9	43531
NAD+ binding	0.59	0.554387	18.18	2	9	70403
threonine catabolic process	0.59	0.554387	18.18	2	9	6567
negative regulation of DNA-templated transcription, termination	0.59	0.554387	18.18	2	9	60567
primary active transmembrane transporter activity	0.59	0.554387	18.18	2	9	15399
P-P-bond-hydrolysis-driven transmembrane transporter activity	0.59	0.554387	18.18	2	9	15405

pyrophosphatase activity	0.59	0.554387	18.18	2	9	16462
hydrolase activity, acting on acid anhydrides	0.59	0.554387	18.18	2	9	16817
hydrolase activity, acting on acid anhydrides, in phosphorus-containing anhydrides	0.59	0.554387	18.18	2	9	16818
ATPase activity	0.59	0.554387	18.18	2	9	16887
nucleoside-triphosphatase activity	0.59	0.554387	18.18	2	9	17111
ATPase activity, coupled	0.59	0.554387	18.18	2	9	42623
ATPase activity, coupled to transmembrane movement of substances	0.59	0.554387	18.18	2	9	42626
ATPase activity, coupled to movement of substances	0.59	0.554387	18.18	2	9	43492
transferase activity, transferring aminoacyl groups	0.59	0.554387	18.18	2	9	16755
phosphorylation	0.57	0.562914	16.67	4	20	16310
nucleoside phosphate catabolic process	0.57	0.562914	16.67	4	20	1901292
nucleobase-containing small molecule biosynthetic process	0.57	0.562914	16.67	4	20	34404
pyruvate biosynthetic process	0.57	0.562914	16.67	4	20	42866
ADP metabolic process	0.57	0.562914	16.67	4	20	46031
nucleotide phosphorylation	0.57	0.562914	16.67	4	20	46939
glycolytic process	0.57	0.562914	16.67	4	20	6096
nucleoside diphosphate phosphorylation	0.57	0.562914	16.67	4	20	6165
ATP generation from ADP	0.57	0.562914	16.67	4	20	6757

nucleoside diphosphate metabolic process	0.57	0.562914	16.67	4	20	9132
purine nucleoside diphosphate metabolic process	0.57	0.562914	16.67	4	20	9135
nucleotide catabolic process	0.57	0.562914	16.67	4	20	9166
purine ribonucleoside diphosphate metabolic process	0.57	0.562914	16.67	4	20	9179
ribonucleoside diphosphate metabolic process	0.57	0.562914	16.67	4	20	9185
ion transport	0.55	0.577914	15.93	29	153	6811
inorganic anion transport	0.55	0.578802	16.67	3	15	15698
inorganic anion transmembrane transport	0.55	0.578802	16.67	3	15	98661
amine metabolic process	0.55	0.57956	15.91	14	74	9308
transferase activity, transferring acyl groups	0.54	0.581417	15.89	17	90	16746
spermidine transport	0.53	0.587993	20.00	1	4	15848
spermidine transmembrane transport	0.53	0.587993	20.00	1	4	1903711
RNA repair	0.53	0.587993	20.00	1	4	42245
double-strand break repair via homologous recombination	0.53	0.587993	20.00	1	4	724
DNA synthesis involved in DNA repair	0.53	0.587993	20.00	1	4	731
metallochaperone activity	0.53	0.587993	20.00	1	4	16530
ATPase-coupled phosphate ion transmembrane transporter activity	0.53	0.587993	20.00	1	4	15415
intrinsic component of membrane	0.53	0.587993	20.00	1	4	31224
DNA translocase activity	0.53	0.587993	20.00	1	4	15616
endodeoxyribonuclease activity	0.53	0.587993	20.00	1	4	4520

organic anion transport	0.53	0.587993	20.00	1	4	15711
succinyl-CoA metabolic process	0.53	0.587993	20.00	1	4	6104
amino-acid betaine biosynthetic process	0.53	0.587993	20.00	1	4	6578
glutathione biosynthetic process	0.53	0.587993	20.00	1	4	6750
translation release factor activity	0.53	0.587993	20.00	1	4	3747
cellular modified amino acid biosynthetic process	0.52	0.597042	16.00	4	21	42398
organic phosphonate transport	0.50	0.603743	16.67	2	10	15716
polysaccharide catabolic process	0.50	0.603743	16.67	2	10	272
regulation of transmembrane transport	0.50	0.603743	16.67	2	10	34762
regulation of ion transmembrane transport	0.50	0.603743	16.67	2	10	34765
regulation of ion transport	0.50	0.603743	16.67	2	10	43269
regulation of transport	0.50	0.603743	16.67	2	10	51049
transmembrane transport	0.50	0.607023	15.73	28	150	55085
methionine metabolic process	0.48	0.617316	15.79	3	16	6555
methionine biosynthetic process	0.48	0.617316	15.79	3	16	9086
intrinsic component of plasma membrane	0.48	0.617316	15.79	3	16	31226
extracellular polysaccharide biosynthetic process	0.48	0.617316	15.79	3	16	45226
nicotinamide nucleotide biosynthetic process	0.47	0.625338	15.38	8	44	19359
pyridine nucleotide biosynthetic process	0.47	0.625338	15.38	8	44	19363
cellular catabolic process	0.46	0.629719	15.63	35	189	44248

cellular aromatic compound metabolic process	0.43	0.64884	15.38	2	11	6725
negative regulation of bacterial-type flagellum-dependent cell motility	0.43	0.64884	15.38	2	11	1902201
negative regulation of cell motility	0.43	0.64884	15.38	2	11	2000146
negative regulation of locomotion	0.43	0.64884	15.38	2	11	40013
negative regulation of cellular component movement	0.43	0.64884	15.38	2	11	51271
amide transport	0.43	0.64884	15.38	2	11	42886
	0.43	0.64884	15.38	2	11	43234
folic acid biosynthetic process	0.43	0.64884	15.38	2	11	46656
aspartate kinase activity	0.42	0.654977	16.67	1	5	4072
arginine biosynthetic process via ornithine	0.42	0.654977	16.67	1	5	42450
RNA polymerase binding	0.42	0.654977	16.67	1	5	70063
purine nucleotide metabolic process	0.42	0.654977	16.67	1	5	6163
negative regulation of endoribonuclease activity	0.42	0.654977	16.67	1	5	60702
carbohydrate transmembrane transporter activity	0.42	0.654977	16.67	1	5	15144
regulation of DNA-templated transcription, termination	0.42	0.654977	16.67	1	5	31554
glycerol-3-phosphate metabolic process	0.42	0.654977	16.67	1	5	6072
organic substance metabolic process	0.42	0.654977	16.67	1	5	71704
succinate-CoA ligase (ADP-forming) activity	0.42	0.654977	16.67	1	5	4775
D-tagatose 6-phosphate metabolic process	0.42	0.654977	16.67	1	5	2001058

D-tagatose 6-phosphate catabolic process	0.42	0.654977	16.67	1	5	2001059
regulation of carbohydrate utilization	0.42	0.654977	16.67	1	5	43610
xenobiotic metabolic process	0.42	0.657516	15.00	9	51	6805
sulfur amino acid metabolic process	0.42	0.660276	14.81	4	23	96
sulfur amino acid biosynthetic process	0.42	0.660276	14.81	4	23	97
response to acid chemical	0.42	0.660276	14.81	4	23	1101
L-alpha-amino acid transmembrane transport	0.41	0.660365	14.89	7	40	1902475
chaperone-mediated protein folding	0.39	0.675362	14.63	6	35	61077
L-amino acid transport	0.38	0.682786	14.58	7	41	15807
NAD metabolic process	0.37	0.689249	14.29	4	24	19674
NAD biosynthetic process	0.37	0.689249	14.29	4	24	9435
Gram-negative-bacterium-type cell wall biogenesis	0.37	0.689756	14.29	2	12	43164
sulfate assimilation	0.37	0.689756	14.29	2	12	103
signal peptide processing	0.34	0.71108	14.29	1	6	6465
ammonium transmembrane transport	0.34	0.71108	14.29	1	6	72488
phosphogluconate dehydrogenase (decarboxylating) activity	0.34	0.71108	14.29	1	6	4616
asparagine metabolic process	0.34	0.71108	14.29	1	6	6528
asparagine biosynthetic process	0.34	0.71108	14.29	1	6	6529
cysteine biosynthetic process from serine	0.34	0.71108	14.29	1	6	6535
response to silver ion	0.34	0.71108	14.29	1	6	10272
tRNA wobble position uridine thiolation	0.34	0.71108	14.29	1	6	2143
protein kinase activity	0.34	0.71108	14.29	1	6	4672

type II protein secretion system complex	0.34	0.71108	14.29	1	6	15627
sister chromatid cohesion	0.34	0.71108	14.29	1	6	7062
C4-dicarboxylate transmembrane transporter activity	0.34	0.71108	14.29	1	6	15556
C4-dicarboxylate transport	0.34	0.71108	14.29	1	6	15740
allantoin catabolic process	0.34	0.71108	14.29	1	6	256
succinate metabolic process	0.34	0.71108	14.29	1	6	6105
transition metal ion transport	0.33	0.717927	13.89	5	31	41
iron ion transport	0.33	0.717927	13.89	5	31	6826
regulation of bacterial-type flagellumdependent cell motility	0.32	0.72666	13.33	2	13	1902021
tryptophan biosynthetic process	0.32	0.72666	13.33	2	13	162
indole-containing compound metabolic process	0.32	0.72666	13.33	2	13	42430
indole-containing compound biosynthetic process	0.32	0.72666	13.33	2	13	42435
indolalkylamine biosynthetic process	0.32	0.72666	13.33	2	13	46219
tryptophan metabolic process	0.32	0.72666	13.33	2	13	6568
indolalkylamine metabolic process	0.32	0.72666	13.33	2	13	6586
kinase activity	0.31	0.735292	15.07	44	248	16301
protein autophosphorylation	0.30	0.740881	13.51	5	32	46777
response to oxygen-containing compound	0.30	0.741809	13.33	4	26	1901700
biological regulation	0.29	0.74537	14.69	26	151	65007
alditol metabolic process	0.29	0.746577	13.04	3	20	19400
polyol metabolic process	0.29	0.746577	13.04	3	20	19751
glycerol metabolic process	0.29	0.746577	13.04	3	20	6071

endoribonuclease activity	0.29	0.746577	13.04	3	20	4521
phosphorylation	0.29	0.751924	14.97	44	250	16310
cell division	0.28	0.752211	14.29	16	96	51301
cysteine biosynthetic process	0.28	0.758067	12.50	1	7	19344
cysteine metabolic process	0.28	0.758067	12.50	1	7	6534
detoxification of copper ion	0.28	0.758067	12.50	1	7	10273
motor activity	0.28	0.758067	12.50	1	7	3774
sodium:dicarboxylate symporter activity	0.28	0.758067	12.50	1	7	17153
recombinational repair	0.28	0.758067	12.50	1	7	725
ATP-dependent DNA helicase activity	0.27	0.759773	12.50	2	14	4003
negative regulation of cellular component organization	0.27	0.759773	12.50	2	14	51129
pteridine-containing compound metabolic process	0.27	0.759773	12.50	2	14	42558
pteridine-containing compound biosynthetic process	0.27	0.759773	12.50	2	14	42559
folic acid metabolic process	0.27	0.759773	12.50	2	14	46655
folic acid biosynthetic process	0.27	0.759773	12.50	2	14	46656
folic acid-containing compound metabolic process	0.27	0.759773	12.50	2	14	6760
folic acid-containing compound biosynthetic process	0.27	0.759773	12.50	2	14	9396
tetrahydrofolate biosynthetic process	0.27	0.759773	12.50	2	14	46654
negative regulation of biological process	0.27	0.762433	13.16	5	33	48519
heme binding	0.27	0.764438	13.56	8	51	20037
cell	0.27	0.765428	12.90	4	27	5623

carboxylic acid catabolic process	0.25	0.777026	14.04	16	98	46395
exonuclease activity	0.25	0.780573	13.04	6	40	4527
regulation of localization	0.24	0.787325	12.50	4	28	32879
glutathione transferase activity	0.24	0.789353	11.76	2	15	4364
extrinsic component of cytoplasmic side of plasma membrane	0.24	0.789353	11.76	2	15	31234
phosphotransferase activity, alcohol group as acceptor	0.23	0.796721	12.96	7	47	16773
peroxidase activity	0.23	0.796783	12.00	3	22	4601
N-acetylglucosamine metabolic process	0.23	0.797418	11.11	1	8	6044
negative regulation of gene expression	0.23	0.797418	11.11	1	8	10629
negative regulation of DNA-binding transcription factor activity	0.23	0.797418	11.11	1	8	43433
outer membrane	0.22	0.800087	13.79	16	100	19867
cell outer membrane	0.22	0.800087	13.79	16	100	9279
metal ion transport	0.22	0.801422	12.50	5	35	30001
organic substance transport	0.22	0.805945	13.85	18	112	71702
oxidoreductase activity, acting on CHOH group of donors	0.21	0.807557	12.12	4	29	16614
cellular lipid metabolic process	0.20	0.815536	13.40	13	84	44255
lipid metabolic process	0.20	0.815536	13.40	13	84	6629
integral component of cell outer membrane	0.20	0.815671	11.11	2	16	45203
monocarboxylic acid catabolic process	0.20	0.815671	11.11	2	16	72329
cellular amino acid catabolic process	0.20	0.817352	13.46	14	90	9063
cell wall organization	0.19	0.823574	13.60	17	108	71555
transferase activity, transferring phosphorus-containing groups	0.19	0.825144	12.70	8	55	16772

cellular modified amino acid metabolic process	0.19	0.826192	11.76	4	30	6575
regulation of gene expression	0.19	0.826727	13.27	13	85	10468
response to external stimulus	0.19	0.826914	12.50	7	49	9605
response to extracellular stimulus	0.19	0.826914	12.50	7	49	9991
heme transporter activity	0.19	0.830374	10.00	1	9	15232
double-strand break repair	0.19	0.830374	10.00	1	9	6302
glycerophospholipid biosynthetic process	0.19	0.830374	10.00	1	9	46474
nickel cation transmembrane transport	0.19	0.830374	10.00	1	9	35444
nitrogen compound transport	0.18	0.83841	13.21	14	92	71705
N-terminal protein amino acid acetylation	0.18	0.839007	10.53	2	17	6474
DNA-templated transcription, initiation	0.18	0.839007	10.53	2	17	6352
protein histidine kinase activity	0.17	0.844415	12.00	6	44	4673
regulation of cellular macromolecule biosynthetic process	0.17	0.84757	13.00	13	87	2000112
anion transport	0.17	0.847665	12.66	10	69	6820
anion transmembrane transport	0.17	0.847665	12.66	10	69	98656
amino acid transmembrane transport	0.16	0.853464	12.07	7	51	3333
amino acid transport	0.16	0.853464	12.07	7	51	6865
iron-sulfur cluster binding	0.15	0.856561	14.07	37	226	51536
fimbrial usher porin activity	0.15	0.857973	9.09	1	10	15473
negative regulation of cellular macromolecule biosynthetic process	0.15	0.857973	9.09	1	10	2000113
chromosome condensation	0.15	0.857973	9.09	1	10	30261
'de novo' UMP biosynthetic process	0.15	0.857973	9.09	1	10	44205

amino acid-transporting ATPase activity	0.15	0.857973	9.09	1	10	15424
organic acid catabolic process	0.15	0.858948	13.11	16	106	16054
aminopeptidase activity	0.15	0.859634	10.00	2	18	4177
regulation of single-species biofilm formation	0.15	0.859634	10.00	2	18	1900190
regulation of cell motility	0.15	0.859634	10.00	2	18	2000145
regulation of locomotion	0.15	0.859634	10.00	2	18	40012
regulation of multi-organism process	0.15	0.859634	10.00	2	18	43900
regulation of cellular component movement	0.15	0.859634	10.00	2	18	51270
small molecule catabolic process	0.14	0.867313	13.01	16	107	44282
cytochrome complex assembly	0.14	0.870179	11.54	6	46	17004
cellular protein-containing complex assembly	0.14	0.870179	11.54	6	46	34622
protein-containing complex subunit organization	0.14	0.870179	11.54	6	46	43933
protein-containing complex assembly	0.14	0.870179	11.54	6	46	65003
sulfur compound transport	0.14	0.872425	10.34	3	26	72348
copper ion binding	0.14	0.872425	10.34	3	26	5507
ATPase activity, coupled to transmembrane movement of substances	0.14	0.872425	10.34	3	26	42626
response to desiccation	0.13	0.877815	9.52	2	19	9269
response to water deprivation	0.13	0.877815	9.52	2	19	9414
response to water	0.13	0.877815	9.52	2	19	9415
negative regulation of macromolecule biosynthetic process	0.13	0.881085	8.33	1	11	10558

negative regulation of macromolecule metabolic process	0.13	0.881085	8.33	1	11	10605
negative regulation of cellular metabolic process	0.13	0.881085	8.33	1	11	31324
negative regulation of cellular biosynthetic process	0.13	0.881085	8.33	1	11	31327
negative regulation of nitrogen compound metabolic process	0.13	0.881085	8.33	1	11	51172
negative regulation of biosynthetic process	0.13	0.881085	8.33	1	11	9890
negative regulation of metabolic process	0.13	0.881085	8.33	1	11	9892
transferase activity, transferring pentosyl groups	0.13	0.881085	8.33	1	11	16763
nonribosomal peptide biosynthetic process	0.13	0.881085	8.33	1	11	19184
alcohol metabolic process	0.12	0.886938	10.00	3	27	6066
tricarboxylic acid cycle	0.12	0.886938	10.00	3	27	6099
citrate metabolic process	0.12	0.886938	10.00	3	27	6101
tricarboxylic acid metabolic process	0.12	0.886938	10.00	3	27	72350
organic anion transport	0.12	0.887005	11.48	7	54	15711
carboxylic acid transmembrane transport	0.12	0.887005	11.48	7	54	1905039
carboxylic acid transport	0.12	0.887005	11.48	7	54	46942
transposition, DNA-mediated	0.11	0.892521	11.59	8	61	6313
nucleotide-sugar metabolic process	0.11	0.893799	9.09	2	20	9225
nucleotide-sugar biosynthetic process	0.11	0.893799	9.09	2	20	9226
amino sugar biosynthetic process	0.11	0.893799	9.09	2	20	46349

amino sugar metabolic process	0.11	0.893799	9.09	2	20	6040
regulation of DNA-templated transcription, initiation	0.11	0.893799	9.09	2	20	2000142
regulation of protein complex assembly	0.11	0.893799	9.09	2	20	43254
active transmembrane transporter activity	0.11	0.893799	9.09	2	20	22804
dephosphorylation	0.11	0.89802	11.69	9	68	16311
monocarboxylic acid biosynthetic process	0.11	0.899373	10.64	5	42	72330
organic cation transport	0.10	0.900438	7.69	1	12	15695
ammonium transport	0.10	0.900438	7.69	1	12	15696
glycerol metabolic process	0.10	0.900438	7.69	1	12	6071
regulation of biological process	0.10	0.903826	12.88	21	142	50789
regulation of macromolecule biosynthetic process	0.10	0.905556	12.15	13	94	10556
regulation of metabolic process	0.10	0.905556	12.15	13	94	19222
regulation of cellular metabolic process	0.10	0.905556	12.15	13	94	31323
regulation of cellular biosynthetic process	0.10	0.905556	12.15	13	94	31326
regulation of nitrogen compound metabolic process	0.10	0.905556	12.15	13	94	51171
regulation of macromolecule metabolic process	0.10	0.905556	12.15	13	94	60255
regulation of primary metabolic process	0.10	0.905556	12.15	13	94	80090
regulation of biosynthetic process	0.10	0.905556	12.15	13	94	9889
organic acid transport	0.10	0.905588	11.11	7	56	15849
organic acid transmembrane transport	0.10	0.905588	11.11	7	56	1903825

ion transmembrane transport	0.10	0.90782	8.70	2	21	34220
polysaccharide biosynthetic process	0.10	0.90782	8.70	2	21	271
cytochrome complex assembly	0.10	0.90782	8.70	2	21	17004
amine catabolic process	0.09	0.9111	10.71	6	50	9310
phosphate ion transmembrane transport	0.09	0.916645	7.14	1	13	35435
amino-acid betaine metabolic process	0.09	0.916645	7.14	1	13	6577
phosphorelay sensor kinase activity	0.08	0.919391	10.53	6	51	155
signal transduction by protein phosphorylation	0.08	0.919391	10.53	6	51	23014
DNA repair	0.08	0.921495	10.77	7	58	6281
hydrolase activity, hydrolyzing Oglycosyl compounds	0.08	0.927463	9.52	4	38	4553
carbohydrate derivative catabolic process	0.07	0.930215	6.67	1	14	1901136
cyclic-guanylate-specific phosphodiesterase activity	0.07	0.930215	6.67	1	14	71111
ATP-binding cassette (ABC) transporter complex, substrate-binding subunit-containing	0.07	0.930816	8.00	2	23	55052
cell division site	0.07	0.931287	8.82	3	31	32153
regulation of cellular process	0.07	0.933828	12.26	19	136	50794
regulation of transcription, DNAtemplated	0.07	0.935764	11.11	10	80	6355
DNA recombination	0.06	0.939496	11.50	13	100	6310
organonitrogen compound catabolic process	0.06	0.940892	12.36	22	156	1901565
amino acid transmembrane transporter activity	0.05	0.946806	9.43	5	48	15171

extracellular region	0.05	0.946859	8.33	3	33	5576
negative regulation of cellular process	0.05	0.946859	8.33	3	33	48523
antibiotic metabolic process	0.05	0.946859	8.33	3	33	16999
ion transmembrane transporter activity	0.05	0.94831	7.41	2	25	15075
defense response to virus	0.05	0.951092	5.88	1	16	51607
amino acid transmembrane transport	0.05	0.952094	10.26	8	70	3333
phosphorelay response regulator activity	0.05	0.95335	8.11	3	34	156
ethanolamine catabolic process	0.05	0.955388	7.14	2	26	46336
cellular response to stress	0.04	0.960256	9.72	7	65	33554
cellular response to stimulus	0.04	0.960256	9.72	7	65	51716
cellular response to DNA damage stimulus	0.04	0.960256	9.72	7	65	6974
transmembrane transporter activity	0.04	0.961534	6.90	2	27	22857
transporter activity	0.04	0.961534	6.90	2	27	5215
carbohydrate metabolic process	0.04	0.962266	13.77	80	501	5975
regulation of nucleic acid-templated transcription	0.04	0.965123	10.31	10	87	1903506
regulation of RNA biosynthetic process	0.04	0.965123	10.31	10	87	2001141
regulation of RNA metabolic process	0.04	0.965123	10.31	10	87	51252
arginine metabolic process	0.03	0.965727	5.26	1	18	6525
regulation of nucleobase-containing compound metabolic process	0.03	0.970936	10.10	10	89	19219
phosphoprotein phosphatase activity	0.03	0.971311	5.00	1	19	4721
anaerobic respiration	0.03	0.973029	12.02	28	205	9061
cell projection	0.02	0.977175	8.70	6	63	42995
pilus	0.02	0.977175	8.70	6	63	9289
cellular respiration	0.02	0.978402	12.08	32	233	45333

transcription, DNA-templated	0.02	0.97868	13.49	82	526	6351
protein modification process	0.02	0.980011	9.71	10	93	36211
cellular protein modification process	0.02	0.980011	9.71	10	93	6464
DNA integration	0.02	0.981756	8.45	6	65	15074
protein dephosphorylation	0.01	0.985919	4.17	1	23	6470
fatty acid oxidation	0.01	0.988576	5.41	2	35	19395
lipid modification	0.01	0.988576	5.41	2	35	30258
lipid oxidation	0.01	0.988576	5.41	2	35	34440
monocarboxylic acid metabolic process	0.01	0.989781	9.32	11	107	32787
transcription regulatory region DNA binding	0.01	0.990136	3.85	1	25	44212
cellular protein modification process	0.01	0.991745	3.70	1	26	6464
carbohydrate binding	0.01	0.992139	6.00	3	47	30246
single-species biofilm formation	0.01	0.992302	6.67	4	56	44010
DNA-binding transcription factor activity	0.01	0.99277	11.96	44	324	3700
fatty acid metabolic process	0.01	0.992831	5.00	2	38	6631
colanic acid metabolic process	0.01	0.993183	5.88	3	48	46377
colanic acid biosynthetic process	0.01	0.993183	5.88	3	48	9242
iron ion homeostasis	0.01	0.994759	4.76	2	40	55072
generation of precursor metabolites and energy	0.01	0.994936	11.39	36	280	6091
pilus organization	0.00	0.995952	3.23	1	30	43711
cellular response to extracellular stimulus	0.00	0.995952	3.23	1	30	31668
cellular response to external stimulus	0.00	0.995952	3.23	1	30	71496
cell communication	0.00	0.995952	3.23	1	30	7154
SOS response	0.00	0.995952	3.23	1	30	9432

organelle part	0.00	0.995989	13.34	121	786	44422
DNA binding	0.00	0.99711	13.28	125	816	3677
energy derivation by oxidation of organic compounds	0.00	0.998233	10.63	32	269	15980
membrane	0.00	0.998984	14.41	342	2032	16020
sequence-specific DNA binding	0.00	0.999003	8.43	14	152	43565
regulation of transcription, DNAtemplated	0.00	0.999122	12.15	79	571	6355
organelle inner membrane	0.00	0.999409	12.67	113	779	19866
organelle membrane	0.00	0.999409	12.67	113	779	31090
external encapsulating structure	0.00	0.999471	13.09	148	983	30312
cell wall	0.00	0.999471	13.09	148	983	5618
peptidoglycan-based cell wall	0.00	0.999471	13.09	148	983	9274
phosphorelay signal transduction system	0.00	0.999524	7.48	11	136	160
transporter activity	0.00	0.999579	10.03	32	287	5215
membrane	0.00	0.999592	12.80	129	879	16020
organic substance catabolic process	0.00	0.999604	11.81	75	560	1901575
catabolic process	0.00	0.999604	11.81	75	560	9056
oxidation-reduction process	0.00	0.999688	10.06	34	304	55114
cell adhesion	0.00	0.999853	5.05	5	94	7155
carbohydrate catabolic process	0.00	0.999945	9.88	40	365	16052
integral component of membrane	0.00	1	11.47	215	1660	16021

Table S1g. Gene ontology of 224 DEGs.

function	*Enrichment Score*	*Enrichment pvalue*	*% genes in group that are present*	*# genes in list, in group*	*# genes not in list, in group*	*GO ID*
phosphoenolpyruvate-dependent sugar phosphotransferase system	10.07	4.22E-05	40.00	4	6	9401
carbohydrate transport	8.90	0.000136334	30.77	4	9	8643
energy derivation by oxidation of organic compounds	8.14	0.000292458	5.65	17	284	15980
generation of precursor metabolites and energy	7.57	0.000515119	5.38	17	299	6091
colanic acid metabolic process	7.13	0.000803696	11.76	6	45	46377
colanic acid biosynthetic process	7.13	0.000803696	11.76	6	45	9242
iron ion binding	7.02	0.000897499	7.83	9	106	5506
oxidation-reduction process	6.81	0.0010993	5.03	17	321	55114
pyridine nucleotide metabolic process	6.72	0.00120398	10.91	6	49	19362
nicotinamide nucleotide metabolic process	6.72	0.00120398	10.91	6	49	46496
oxidoreduction coenzyme metabolic process	6.72	0.00120398	10.91	6	49	6733
glucose 6-phosphate metabolic process	6.56	0.00141393	66.67	2	1	51156
NADP metabolic process	6.56	0.00141393	66.67	2	1	6739
pentose-phosphate shunt, oxidative branch	6.56	0.00141393	66.67	2	1	9051
plasmid maintenance	6.50	0.00150047	27.27	3	8	6276
metal ion transport	6.39	0.00168292	12.50	5	35	30001
anaerobic respiration	6.36	0.00173118	5.58	13	220	9061
heme binding	6.35	0.00174169	10.17	6	53	20037
oxidation-reduction process	6.28	0.00187852	3.73	31	801	55114
glutamate decarboxylase activity	5.88	0.00278712	50.00	2	2	4351
glutamate metabolic process	5.88	0.00278712	50.00	2	2	6536
acetyl-CoA metabolic process	5.88	0.00278712	50.00	2	2	6084

acetyl-CoA biosynthetic process	5.88	0.00278712	50.00	2	2	6085
acetyl-CoA biosynthetic process from pyruvate	5.88	0.00278712	50.00	2	2	6086
copper ion homeostasis	5.88	0.00278712	50.00	2	2	55070
magnesium ion transport	5.88	0.00278712	50.00	2	2	15693
magnesium ion transmembrane transport	5.88	0.00278712	50.00	2	2	1903830
oxidoreductase activity, acting on paired donors, with incorporation or reduction of molecular oxygen, 2-oxoglutarate as one donor, and incorporation of one atom each of oxygen into both donors	5.88	0.00278712	50.00	2	2	16706
nitric oxide catabolic process	5.88	0.00278712	50.00	2	2	46210
protein histidine kinase binding	5.88	0.00278712	50.00	2	2	43424
pyridine-containing compound metabolic process	5.63	0.00360127	8.82	6	62	72524
'de novo' GDP-L-fucose biosynthetic process	5.39	0.0045784	40.00	2	3	42351
cellular respiration	5.25	0.00527265	4.91	13	252	45333
phosphorylase activity	5.00	0.006769	33.33	2	4	4645
dormancy process	4.67	0.00934078	28.57	2	5	22611
UDP-glucose metabolic process	4.67	0.00934078	28.57	2	5	6011
cellulose biosynthetic process	4.67	0.00934078	28.57	2	5	30244
CDP-diacylglycerol biosynthetic process	4.67	0.00934078	28.57	2	5	16024
catalytic complex	4.67	0.00934078	28.57	2	5	1902494
glucokinase activity	4.67	0.00934078	28.57	2	5	4340
glucose 6-phosphate metabolic process	4.67	0.00934078	28.57	2	5	51156
flavin adenine dinucleotide binding	4.53	0.0107804	6.31	7	104	50660
intracellular pH elevation	4.40	0.0122762	25.00	2	6	51454
NADP+ binding	4.40	0.0122762	25.00	2	6	70401
divalent metal ion transport	4.40	0.0122762	25.00	2	6	70838

divalent inorganic cation transport	4.40	0.0122762	25.00	2	6	72511
iron ion homeostasis	4.34	0.0130453	9.52	4	38	55072
hydrolase activity	4.32	0.0132861	13.04	3	20	16787
tryptophan biosynthetic process	4.16	0.0155583	22.22	2	7	162
aldehyde catabolic process	4.16	0.0155583	22.22	2	7	46185
glyoxylate catabolic process	4.16	0.0155583	22.22	2	7	9436
ATPase activity, coupled to transmembrane movement of ions, phosphorylative mechanism	4.16	0.0155583	22.22	2	7	15662
active ion transmembrane transporter activity	4.16	0.0155583	22.22	2	7	22853
ATPase coupled ion transmembrane transporter activity	4.16	0.0155583	22.22	2	7	42625
catalytic activity	4.11	0.0163583	7.35	5	63	3824
glycerophospholipid biosynthetic process	3.95	0.0191706	20.00	2	8	46474
thioester metabolic process	3.95	0.0191706	20.00	2	8	35383
thioester biosynthetic process	3.95	0.0191706	20.00	2	8	35384
acyl-CoA metabolic process	3.95	0.0191706	20.00	2	8	6637
acyl-CoA biosynthetic process	3.95	0.0191706	20.00	2	8	71616
nickel cation transmembrane transport	3.95	0.0191706	20.00	2	8	35444
cellular polysaccharide biosynthetic process	3.94	0.0194179	6.19	6	91	33692
cellular carbohydrate biosynthetic process	3.94	0.0194179	6.19	6	91	34637
cellular polysaccharide metabolic process	3.94	0.0194179	6.19	6	91	44264
transferase activity, transferring glycosyl groups	3.90	0.0203222	6.12	6	92	16757
fructose transmembrane transport	3.77	0.0230972	18.18	2	9	15755
ADP binding	3.77	0.0230972	18.18	2	9	43531
primary active transmembrane transporter activity	3.77	0.0230972	18.18	2	9	15399

P-P-bond-hydrolysis-driven transmembrane transporter activity	3.77	0.0230972	18.18	2	9	15405
pyrophosphatase activity	3.77	0.0230972	18.18	2	9	16462
hydrolase activity, acting on acid anhydrides	3.77	0.0230972	18.18	2	9	16817
hydrolase activity, acting on acid anhydrides, in phosphorus-containing anhydrides	3.77	0.0230972	18.18	2	9	16818
ATPase activity	3.77	0.0230972	18.18	2	9	16887
nucleoside-triphosphatase activity	3.77	0.0230972	18.18	2	9	17111
ATPase activity, coupled	3.77	0.0230972	18.18	2	9	42623
ATPase activity, coupled to transmembrane movement of substances	3.77	0.0230972	18.18	2	9	42626
ATPase activity, coupled to movement of substances	3.77	0.0230972	18.18	2	9	43492
carbohydrate derivative metabolic process	3.64	0.0263167	3.99	13	313	1901135
dephosphorylation	3.63	0.0265415	6.49	5	72	16311
nicotinamide nucleotide biosynthetic process	3.62	0.0266898	7.69	4	48	19359
pyridine nucleotide biosynthetic process	3.62	0.0266898	7.69	4	48	19363
cytochrome complex assembly	3.62	0.0266898	7.69	4	48	17004
cellular protein-containing complex assembly	3.62	0.0266898	7.69	4	48	34622
protein-containing complex subunit organization	3.62	0.0266898	7.69	4	48	43933
protein-containing complex assembly	3.62	0.0266898	7.69	4	48	65003
transferase activity, transferring pentosyl groups	3.60	0.027323	16.67	2	10	16763
chorismate metabolic process	3.60	0.027323	16.67	2	10	46417
chorismate biosynthetic process	3.60	0.027323	16.67	2	10	9423
metal ion transport	3.60	0.027323	16.67	2	10	30001

cellular carbohydrate metabolic process	3.56	0.0285565	5.19	7	128	44262
sequence-specific DNA binding	3.53	0.0291854	4.82	8	158	43565
pilus organization	3.52	0.0297029	9.68	3	28	43711
transferase activity, transferring alkyl or aryl (other than methyl) groups	3.45	0.0318329	15.38	2	11	16765
glycerol metabolic process	3.45	0.0318329	15.38	2	11	6071
oxidoreductase activity, acting on paired donors, with incorporation or reduction of molecular oxygen	3.45	0.0318329	15.38	2	11	16705
polysaccharide biosynthetic process	3.40	0.0334727	5.45	6	104	271
cellular component assembly	3.33	0.0358336	7.02	4	53	22607
carbohydrate derivative biosynthetic process	3.32	0.0362428	4.04	11	261	1901137
pyrimidine nucleobase metabolic process	3.31	0.0366127	14.29	2	12	6206
glutamate biosynthetic process	3.31	0.0366127	14.29	2	12	6537
cellular response to phosphate starvation	3.31	0.0366127	14.29	2	12	16036
tryptophan biosynthetic process	3.18	0.0416486	13.33	2	13	162
indole-containing compound metabolic process	3.18	0.0416486	13.33	2	13	42430
indole-containing compound biosynthetic process	3.18	0.0416486	13.33	2	13	42435
indolalkylamine biosynthetic process	3.18	0.0416486	13.33	2	13	46219
tryptophan metabolic process	3.18	0.0416486	13.33	2	13	6568
indolalkylamine metabolic process	3.18	0.0416486	13.33	2	13	6586
cellular component organization	3.17	0.0420888	6.67	4	56	16043
cellular component organization or biogenesis	3.17	0.0420888	6.67	4	56	71840
amine metabolic process	3.13	0.0435682	5.68	5	83	9308
transition metal ion transport	3.13	0.043609	8.33	3	33	41

iron ion transport	3.13	0.043609	8.33	3	33	6826
cofactor metabolic process	3.07	0.0464311	4.67	7	143	51186
fermentation	3.07	0.0465809	6.45	4	58	6113
transferase activity, transferring phosphoruscontaining groups	3.02	0.0489234	6.35	4	59	16772
polysaccharide metabolic process	2.97	0.0511944	4.92	6	116	5976
dioxygenase activity	2.95	0.0524355	11.76	2	15	51213
pyridine-containing compound biosynthetic process	2.92	0.0538005	6.15	4	61	72525
DNA binding	2.92	0.0540917	2.98	28	913	3677
NADP binding	2.87	0.0566793	7.50	3	37	50661
glyoxylate metabolic process	2.84	0.0581611	11.11	2	16	46487
monocarboxylic acid catabolic process	2.84	0.0581611	11.11	2	16	72329
coenzyme metabolic process	2.84	0.0581732	4.76	6	120	6732
cellular biogenic amine biosynthetic process	2.75	0.0638342	7.14	3	39	42401
amine biosynthetic process	2.75	0.0638342	7.14	3	39	9309
phosphopyruvate hydratase complex	2.74	0.0644008	33.33	1	2	15
phosphopyruvate hydratase activity	2.74	0.0644008	33.33	1	2	4634
CTP biosynthetic process	2.74	0.0644008	33.33	1	2	6241
glycine biosynthetic process	2.74	0.0644008	33.33	1	2	6545
'de novo' CTP biosynthetic process	2.74	0.0644008	33.33	1	2	44210
5-amino-6-(5-phosphoribitylamino)uracil phosphatase activity	2.74	0.0644008	33.33	1	2	43726
regulation of carbohydrate catabolic process	2.74	0.0644008	33.33	1	2	43470
glucosamine-6-phosphate deaminase activity	2.74	0.0644008	33.33	1	2	4342
nucleoside bisphosphate metabolic process	2.66	0.0702164	10.00	2	18	33865
nucleoside bisphosphate biosynthetic process	2.66	0.0702164	10.00	2	18	33866

ribonucleoside bisphosphate metabolic process	2.66	0.0702164	10.00	2	18	33875
ribonucleoside bisphosphate biosynthetic process	2.66	0.0702164	10.00	2	18	34030
purine nucleoside bisphosphate metabolic process	2.66	0.0702164	10.00	2	18	34032
purine nucleoside bisphosphate biosynthetic process	2.66	0.0702164	10.00	2	18	34033
cellular amine metabolic process	2.64	0.071385	6.82	3	41	44106
cellular biogenic amine metabolic process	2.64	0.071385	6.82	3	41	6576
transcription, DNA-templated	2.63	0.0720575	3.13	19	589	6351
response to desiccation	2.57	0.0765232	9.52	2	19	9269
response to water deprivation	2.57	0.0765232	9.52	2	19	9414
response to water	2.57	0.0765232	9.52	2	19	9415
amino sugar biosynthetic process	2.49	0.0830017	9.09	2	20	46349
amino sugar metabolic process	2.49	0.0830017	9.09	2	20	6040
active transmembrane transporter activity	2.49	0.0830017	9.09	2	20	22804
localization	2.48	0.0840409	3.72	9	233	51179
establishment of localization	2.48	0.0840409	3.72	9	233	51234
transport	2.48	0.0840409	3.72	9	233	6810
dihydrofolate reductase activity	2.47	0.0849387	25.00	1	3	4146
2,3-dihydroxybenzoate-serine ligase activity	2.47	0.0849387	25.00	1	3	47527
transferase activity, transferring acyl groups	2.46	0.0855754	4.67	5	102	16746
nucleobase-containing small molecule metabolic process	2.42	0.0891171	3.67	9	236	55086
polysaccharide biosynthetic process	2.41	0.0896413	8.70	2	21	271
glucose metabolic process	2.41	0.0896413	8.70	2	21	6006
phosphorylation	2.34	0.0964319	8.33	2	22	16310

nucleoside phosphate catabolic process	2.34	0.0964319	8.33	2	22	1901292
nucleobase-containing small molecule biosynthetic process	2.34	0.0964319	8.33	2	22	34404
pyruvate biosynthetic process	2.34	0.0964319	8.33	2	22	42866
ADP metabolic process	2.34	0.0964319	8.33	2	22	46031
nucleotide phosphorylation	2.34	0.0964319	8.33	2	22	46939
glycolytic process	2.34	0.0964319	8.33	2	22	6096
nucleoside diphosphate phosphorylation	2.34	0.0964319	8.33	2	22	6165
ATP generation from ADP	2.34	0.0964319	8.33	2	22	6757
nucleoside diphosphate metabolic process	2.34	0.0964319	8.33	2	22	9132
purine nucleoside diphosphate metabolic process	2.34	0.0964319	8.33	2	22	9135
nucleotide catabolic process	2.34	0.0964319	8.33	2	22	9166
purine ribonucleoside diphosphate metabolic process	2.34	0.0964319	8.33	2	22	9179
ribonucleoside diphosphate metabolic process	2.34	0.0964319	8.33	2	22	9185
nucleoside phosphate metabolic process	2.27	0.103727	4.08	6	141	6753
nucleotide metabolic process	2.27	0.103727	4.08	6	141	9117
lipoic acid binding	2.25	0.105029	20.00	1	4	31405
DNA-binding transcription factor activity	2.22	0.108752	3.26	12	356	3700
response to acid chemical	2.14	0.117615	7.41	2	25	1101
cellular protein modification process	2.14	0.117615	7.41	2	25	6464
ion transmembrane transporter activity	2.14	0.117615	7.41	2	25	15075
regulation of transcription, DNA-templated	2.13	0.118407	2.92	19	631	6355
glycerol-3-phosphate metabolic process	2.08	0.124681	16.67	1	5	6072
cellular response to stress	2.08	0.124681	16.67	1	5	33554
amide biosynthetic process	2.08	0.124903	3.87	6	149	43604

NAD metabolic process	2.08	0.124916	7.14	2	26	19674
NAD biosynthetic process	2.08	0.124916	7.14	2	26	9435
response to inorganic substance	2.02	0.132323	6.90	2	27	10035
transmembrane transporter activity	2.02	0.132323	6.90	2	27	22857
transporter activity	2.02	0.132323	6.90	2	27	5215
carbohydrate metabolic process	2.01	0.134286	2.93	17	564	5975
carbohydrate biosynthetic process	1.97	0.139137	3.75	6	154	16051
organophosphate catabolic process	1.97	0.139828	6.67	2	28	46434
response to oxygen-containing compound	1.97	0.139828	6.67	2	28	1901700
protein quality control for misfolded or incompletely synthesized proteins	1.94	0.143904	14.29	1	6	6515
protein kinase activity	1.94	0.143904	14.29	1	6	4672
cation transport	1.92	0.146287	3.94	5	122	6812
organophosphate metabolic process	1.90	0.14909	3.38	8	229	19637
phosphate-containing compound metabolic process	1.90	0.14909	3.38	8	229	6796
cellular amide metabolic process	1.85	0.157173	3.61	6	160	43603
phenol-containing compound metabolic process	1.82	0.162708	12.50	1	7	18958
siderophore biosynthetic process	1.82	0.162708	12.50	1	7	19290
siderophore biosynthetic process from catechol	1.82	0.162708	12.50	1	7	19540
secondary metabolic process	1.82	0.162708	12.50	1	7	19748
secondary metabolite biosynthetic process	1.82	0.162708	12.50	1	7	44550
phenol-containing compound biosynthetic process	1.82	0.162708	12.50	1	7	46189
siderophore metabolic process	1.82	0.162708	12.50	1	7	9237
enterobactin metabolic process	1.82	0.162708	12.50	1	7	9238

enterobactin biosynthetic process	1.82	0.162708	12.50	1	7	9239
catechol-containing compound metabolic process	1.82	0.162708	12.50	1	7	9712
catechol-containing compound biosynthetic process	1.82	0.162708	12.50	1	7	9713
oxidoreductase activity, acting on CH-OH group of donors	1.81	0.162853	6.06	2	31	16614
transferase activity, transferring acyl groups other than amino-acyl groups	1.72	0.178556	5.71	2	33	16747
pyruvate metabolic process	1.72	0.178556	5.71	2	33	6090
oxidoreductase activity, acting on the aldehyde or oxo group of donors, NAD or NADP as acceptor	1.72	0.178556	5.71	2	33	16620
N-acetylglucosamine metabolic process	1.71	0.181102	11.11	1	8	6044
cellular modified amino acid catabolic process	1.71	0.181102	11.11	1	8	42219
carnitine catabolic process	1.71	0.181102	11.11	1	8	42413
amino-acid betaine catabolic process	1.71	0.181102	11.11	1	8	6579
carnitine metabolic process	1.71	0.181102	11.11	1	8	9437
ATP-dependent peptidase activity	1.71	0.181102	11.11	1	8	4176
protein-containing complex	1.68	0.186494	5.56	2	34	32991
cell projection	1.65	0.192613	4.35	3	66	42995
pilus	1.65	0.192613	4.35	3	66	9289
transposition, DNA-mediated	1.65	0.192613	4.35	3	66	6313
protein serine/threonine kinase activity	1.61	0.199094	10.00	1	9	4674
catalytic activity, acting on a protein	1.61	0.199094	10.00	1	9	140096
peptidase activity	1.61	0.199094	10.00	1	9	8233
fimbrial usher porin activity	1.53	0.216694	9.09	1	10	15473

ammonium ion metabolic process	1.53	0.216694	9.09	1	10	97164
peptide biosynthetic process	1.53	0.217004	3.67	4	105	43043
peptide metabolic process	1.53	0.217004	3.67	4	105	6518
cellular nitrogen compound biosynthetic process	1.52	0.218837	2.87	11	372	44271
cofactor biosynthetic process	1.51	0.220753	3.40	5	142	51188
chaperone-mediated protein folding	1.48	0.226807	4.88	2	39	61077
protein catabolic process	1.45	0.233909	8.33	1	11	30163
nonribosomal peptide biosynthetic process	1.45	0.233909	8.33	1	11	19184
hydrolase activity, hydrolyzing O-glycosyl compounds	1.45	0.234956	4.76	2	40	4553
electron transfer activity	1.45	0.234956	4.76	2	40	9055
oxidoreductase activity, acting on the CHOH group of donors, NAD or NADP as acceptor	1.45	0.234956	4.76	2	40	16616
cellular macromolecule biosynthetic process	1.45	0.235701	2.87	10	339	34645
carboxylic acid catabolic process	1.42	0.240534	3.51	4	110	46395
aromatic amino acid family metabolic process	1.41	0.243121	4.65	2	41	9072
aromatic amino acid family biosynthetic process	1.41	0.243121	4.65	2	41	9073
oxidoreductase activity	1.41	0.243121	4.65	2	41	16491
molecular_function	1.41	0.245089	3.27	5	148	3674
cellular aldehyde metabolic process	1.38	0.251299	4.55	2	42	6081
cellular metabolic process	1.35	0.259468	2.40	45	1827	44237
ATP metabolic process	1.35	0.259485	4.44	2	43	46034
ATP biosynthetic process	1.35	0.259485	4.44	2	43	6754

nucleoside monophosphate metabolic process	1.35	0.259485	4.44	2	43	9123
nucleoside monophosphate biosynthetic process	1.35	0.259485	4.44	2	43	9124
purine nucleoside monophosphate metabolic process	1.35	0.259485	4.44	2	43	9126
purine nucleoside monophosphate biosynthetic process	1.35	0.259485	4.44	2	43	9127
nucleoside triphosphate metabolic process	1.35	0.259485	4.44	2	43	9141
nucleoside triphosphate biosynthetic process	1.35	0.259485	4.44	2	43	9142
purine nucleoside triphosphate metabolic process	1.35	0.259485	4.44	2	43	9144
purine nucleoside triphosphate biosynthetic process	1.35	0.259485	4.44	2	43	9145
ribonucleoside monophosphate biosynthetic process	1.35	0.259485	4.44	2	43	9156
ribonucleoside monophosphate metabolic process	1.35	0.259485	4.44	2	43	9161
purine ribonucleoside monophosphate metabolic process	1.35	0.259485	4.44	2	43	9167
purine ribonucleoside monophosphate biosynthetic process	1.35	0.259485	4.44	2	43	9168
ribonucleoside triphosphate metabolic process	1.35	0.259485	4.44	2	43	9199
ribonucleoside triphosphate biosynthetic process	1.35	0.259485	4.44	2	43	9201
purine ribonucleoside triphosphate metabolic process	1.35	0.259485	4.44	2	43	9205

purine ribonucleoside triphosphate biosynthetic process	1.35	0.259485	4.44	2	43	9206
dicarboxylic acid biosynthetic process	1.35	0.259485	4.44	2	43	43650
amino-acid betaine metabolic process	1.32	0.267221	7.14	1	13	6577
monocarboxylic acid biosynthetic process	1.29	0.275863	4.26	2	45	72330
organic acid catabolic process	1.28	0.279298	3.28	4	118	16054
macromolecule biosynthetic process	1.27	0.282232	2.73	10	356	9059
acetyltransferase activity	1.26	0.283333	6.67	1	14	16407
small molecule catabolic process	1.26	0.284218	3.25	4	119	44282
coenzyme biosynthetic process	1.26	0.284218	3.25	4	119	9108
phosphorus metabolic process	1.25	0.287912	2.81	8	277	6793
phospholipid metabolic process	1.20	0.30039	4.00	2	48	6644
phospholipid biosynthetic process	1.20	0.30039	4.00	2	48	8654
organophosphate biosynthetic process	1.16	0.313633	2.86	6	204	90407
phosphorylation	1.15	0.317227	2.72	8	286	16310
organic substance transport	1.14	0.318981	3.08	4	126	71702
transferase activity	1.14	0.320697	2.44	24	961	16740
pyridoxal phosphate binding	1.13	0.322356	3.30	3	88	30170
nucleobase-containing small molecule interconversion	1.13	0.322356	3.30	3	88	15949
organic hydroxy compound metabolic process	1.12	0.324781	3.77	2	51	1901615
pyrimidine nucleotide biosynthetic process	1.11	0.329589	5.56	1	17	6221
cellular process	1.11	0.330705	2.34	47	1965	9987
metabolic process	1.10	0.33454	2.32	54	2275	8152
DNA-templated transcription, initiation	1.07	0.344339	5.26	1	18	6352
aromatic compound biosynthetic process	1.06	0.344824	2.69	7	253	19438
amine catabolic process	1.05	0.348955	3.57	2	54	9310

translation	1.03	0.358601	3.09	3	94	6412
ion transport	0.99	0.369758	2.75	5	177	6811
inorganic cation transmembrane transport	0.99	0.372837	3.39	2	57	98662
lipid biosynthetic process	0.95	0.388565	3.28	2	59	8610
cellular nitrogen compound catabolic process	0.95	0.388619	2.94	3	99	44270
nucleoside phosphate biosynthetic process	0.94	0.389199	2.78	4	140	1901293
nucleotide biosynthetic process	0.94	0.389199	2.78	4	140	9165
organic substance biosynthetic process	0.94	0.390114	2.36	22	910	1901576
biosynthetic process	0.94	0.390114	2.36	22	910	9058
carbohydrate catabolic process	0.92	0.396836	2.47	10	395	16052
biological_process	0.92	0.400146	2.26	67	2896	8150
glutamine metabolic process	0.92	0.400184	4.35	1	22	6541
alditol metabolic process	0.92	0.400184	4.35	1	22	19400
polyol metabolic process	0.92	0.400184	4.35	1	22	19751
glycerol metabolic process	0.92	0.400184	4.35	1	22	6071
organic hydroxy compound biosynthetic process	0.92	0.400184	4.35	1	22	1901617
cellular protein metabolic process	0.80	0.448483	2.50	5	195	44267
ribose phosphate biosynthetic process	0.80	0.449604	2.90	2	67	46390
purine ribonucleotide metabolic process	0.80	0.449604	2.90	2	67	9150
purine ribonucleotide biosynthetic process	0.80	0.449604	2.90	2	67	9152
ribonucleotide metabolic process	0.80	0.449604	2.90	2	67	9259
ribonucleotide biosynthetic process	0.80	0.449604	2.90	2	67	9260
cellular biosynthetic process	0.80	0.450089	2.30	20	851	44249
polyamine biosynthetic process	0.80	0.451303	3.70	1	26	6596
purine nucleobase metabolic process	0.80	0.451303	3.70	1	26	6144
locomotion	0.78	0.456998	2.86	2	68	40011

taxis	0.78	0.456998	2.86	2	68	42330
kinase activity	0.77	0.460866	2.40	7	285	16301
heterocycle biosynthetic process	0.76	0.468053	2.38	7	287	18130
membrane	0.76	0.468273	2.23	53	2321	16020
outer membrane	0.75	0.470456	2.59	3	113	19867
cell outer membrane	0.75	0.470456	2.59	3	113	9279
signal transduction	0.75	0.471613	2.78	2	70	7165
polyamine metabolic process	0.74	0.475215	3.45	1	28	6595
dicarboxylic acid metabolic process	0.74	0.478832	2.74	2	71	43648
alcohol metabolic process	0.72	0.48678	3.33	1	29	6066
serine-type endopeptidase activity	0.72	0.48678	3.33	1	29	4252
nucleobase-containing compound biosynthetic process	0.71	0.492172	2.42	4	161	34654
inorganic ion transmembrane transport	0.69	0.500128	2.63	2	74	98660
organic cyclic compound biosynthetic process	0.69	0.503631	2.30	7	297	1901362
external encapsulating structure part	0.69	0.504058	2.46	3	119	44462
cellular nitrogen compound metabolic process	0.68	0.506478	2.23	18	788	34641
aromatic compound catabolic process	0.68	0.507103	2.60	2	75	19439
nucleobase-containing compound catabolic process	0.68	0.507103	2.60	2	75	34655
sulfur compound biosynthetic process	0.67	0.514015	2.56	2	76	44272
periplasmic space	0.65	0.520364	2.34	4	167	42597
iron-sulfur cluster binding	0.65	0.520782	2.28	6	257	51536
aerobic respiration	0.65	0.520863	2.53	2	77	9060
cellular modified amino acid metabolic process	0.63	0.530563	2.94	1	33	6575

organic substance catabolic process	0.63	0.534489	2.20	14	621	1901575
catabolic process	0.63	0.534489	2.20	14	621	9056
ribose phosphate metabolic process	0.61	0.541022	2.44	2	80	19693
cellular catabolic process	0.60	0.549031	2.23	5	219	44248
extracellular region	0.60	0.551043	2.78	1	35	5576
organonitrogen compound catabolic process	0.59	0.552311	2.25	4	174	1901565
cytoplasm	0.59	0.556184	2.17	29	1305	5737
organic cyclic compound catabolic process	0.55	0.579565	2.27	2	86	1901361
heterocycle catabolic process	0.55	0.579565	2.27	2	86	46700
cation transmembrane transport	0.53	0.585756	2.25	2	87	98655
organic acid biosynthetic process	0.51	0.600438	2.11	6	279	16053
carboxylic acid biosynthetic process	0.51	0.600438	2.11	6	279	46394
cellular macromolecule metabolic process	0.50	0.609156	2.10	12	560	44260
organonitrogen compound metabolic process	0.48	0.620614	2.10	20	932	1901564
purine nucleotide metabolic process	0.48	0.621492	2.11	2	93	6163
purine nucleotide biosynthetic process	0.48	0.621492	2.11	2	93	6164
purine-containing compound biosynthetic process	0.48	0.621492	2.11	2	93	72522
purine-containing compound metabolic process	0.47	0.627214	2.08	2	94	72521
response to antibiotic	0.46	0.630997	2.04	3	144	46677
cellular lipid metabolic process	0.46	0.632869	2.06	2	95	44255
lipid metabolic process	0.46	0.632869	2.06	2	95	6629
membrane	0.45	0.638594	2.08	21	987	16020
ATP binding	0.44	0.642844	2.06	15	713	5524
cell adhesion	0.44	0.64398	2.02	2	97	7155

cellular aromatic compound metabolic process	0.41	0.66039	2.03	13	628	6725
cellular amino acid metabolic process	0.41	0.664152	2.04	1	48	6520
protein modification process	0.41	0.665408	1.94	2	101	36211
cellular protein modification process	0.41	0.665408	1.94	2	101	6464
protein metabolic process	0.40	0.667906	1.95	5	251	19538
cellular amino acid catabolic process	0.40	0.670602	1.92	2	102	9063
carbohydrate binding	0.40	0.671576	2.00	1	49	30246
organelle inner membrane	0.38	0.686895	2.02	18	874	19866
organelle membrane	0.38	0.686895	2.02	18	874	31090
transporter activity	0.34	0.708311	1.88	6	313	5215
cell wall macromolecule metabolic process	0.34	0.712817	1.79	1	55	44036
cell wall macromolecule biosynthetic process	0.34	0.712817	1.79	1	55	44038
aminoglycan biosynthetic process	0.34	0.712817	1.79	1	55	6023
glycosaminoglycan biosynthetic process	0.34	0.712817	1.79	1	55	6024
cellular component macromolecule biosynthetic process	0.34	0.712817	1.79	1	55	70589
peptidoglycan biosynthetic process	0.34	0.712817	1.79	1	55	9252
small molecule metabolic process	0.34	0.714364	1.97	16	795	44281
organelle part	0.34	0.714476	1.98	18	889	44422
organic substance metabolic process	0.33	0.717749	2.06	40	1905	71704
small molecule biosynthetic process	0.32	0.727906	1.84	6	320	44283
monocarboxylic acid metabolic process	0.31	0.736668	1.69	2	116	32787
single-species biofilm formation	0.30	0.73741	1.67	1	59	44010
response to abiotic stimulus	0.30	0.740929	1.68	2	117	9628
intracellular	0.29	0.746435	1.75	4	225	5622

integral component of membrane	0.29	0.748564	2.03	38	1837	16021
heterocycle metabolic process	0.28	0.753104	1.90	13	673	46483
organonitrogen compound biosynthetic process	0.27	0.764867	1.87	12	631	1901566
cell wall organization	0.27	0.765284	1.60	2	123	71555
organic cyclic compound metabolic process	0.26	0.771267	1.87	13	683	1901360
nucleobase-containing compound metabolic process	0.25	0.775816	1.82	10	539	6139
sulfur compound metabolic process	0.23	0.791181	1.52	2	130	6790
peptidoglycan metabolic process	0.22	0.803777	1.37	1	72	270
glycosaminoglycan metabolic process	0.22	0.803777	1.37	1	72	30203
aminoglycan metabolic process	0.22	0.803777	1.37	1	72	6022
response to chemical	0.22	0.804829	1.47	2	134	42221
ion transmembrane transport	0.19	0.829783	1.39	2	142	34220
external encapsulating structure	0.18	0.831576	1.86	21	1110	30312
cell wall	0.18	0.831576	1.86	21	1110	5618
peptidoglycan-based cell wall	0.18	0.831576	1.86	21	1110	9274
phosphorelay signal transduction system	0.18	0.83838	1.36	2	145	160
primary metabolic process	0.16	0.848528	1.91	32	1647	44238
protein folding	0.15	0.856942	1.30	2	152	6457
carboxylic acid metabolic process	0.15	0.857474	1.62	8	485	19752
oxoacid metabolic process	0.15	0.857474	1.62	8	485	43436
DNA replication	0.14	0.871953	1.09	1	91	6260
manganese ion binding	0.13	0.874802	1.08	1	92	30145
organic acid metabolic process	0.13	0.875989	1.58	8	498	6082
cytoplasm	0.12	0.887243	1.78	22	1212	5737
drug metabolic process	0.11	0.897972	1.16	2	171	17144

intracellular part	0.10	0.903558	1.88	40	2089	44424
nucleotide binding	0.10	0.904123	1.63	13	783	166
transmembrane transport	0.10	0.906799	1.12	2	176	55085
macromolecule metabolic process	0.09	0.911828	1.60	12	740	43170
nitrogen compound metabolic process	0.09	0.912093	1.75	23	1292	6807
macromolecule modification	0.09	0.913344	1.10	2	180	43412
response to stress	0.09	0.917969	1.08	2	183	6950
protein binding	0.09	0.918041	1.69	19	1103	5515
cell part	0.08	0.920911	1.90	50	2580	44464
cellular_component	0.08	0.924684	1.90	50	2587	5575
cellular amino acid metabolic process	0.06	0.944861	1.18	4	335	6520
alpha-amino acid metabolic process	0.05	0.954839	0.92	2	215	1901605
alpha-amino acid biosynthetic process	0.05	0.954839	0.92	2	215	1901607
cellular amino acid biosynthetic process	0.05	0.955688	0.92	2	216	8652
nucleic acid binding	0.02	0.978092	0.59	1	169	3676
DNA metabolic process	0.02	0.983335	0.55	1	181	6259
response to stimulus	0.00	0.995832	0.59	2	335	50896
nucleic acid metabolic process	0.00	0.999086	0.32	1	307	90304

Table S1h. Gene ontology of 7 DEGs.

function	***Enrichment Score***	***Enrichment p-value***	***% genes in group that are present***	***# genes in list, in group***	***# genes not in list, in group***	***GO ID***
phosphopyruvate hydratase complex	6.61	0.00135216	33.33	1	2	15
phosphopyruvate hydratase activity	6.61	0.00135216	33.33	1	2	4634
CTP biosynthetic process	6.61	0.00135216	33.33	1	2	6241
glycine biosynthetic process	6.61	0.00135216	33.33	1	2	6545
'de novo' CTP biosynthetic process	6.61	0.00135216	33.33	1	2	44210
dihydrofolate reductase activity	6.32	0.00180261	25.00	1	3	4146

oxidation-reduction process	6.24	0.00194872	0.36	3	829	55114
pyrimidine nucleotide biosynthetic process	4.82	0.0080947	5.56	1	17	6221
glutamine metabolic process	4.57	0.0103354	4.35	1	22	6541
extracellular region	4.13	0.0161456	2.78	1	35	5576
NADP binding	4.02	0.0179287	2.50	1	39	50661
membrane	3.09	0.0453775	0.13	3	2371	16020
response to antibiotic	2.74	0.0648321	0.68	1	146	46677
nucleic acid binding	2.59	0.0747151	0.59	1	169	3676
cytoplasm	2.40	0.0903785	0.16	2	1232	5737
integral component of membrane	1.64	0.19342	0.11	2	1873	16021
intracellular part	1.42	0.241574	0.09	2	2127	44424
transcription, DNA-templated	1.39	0.24987	0.16	1	607	6351
regulation of transcription, DNAtemplated	1.33	0.265397	0.15	1	649	6355
ATP binding	1.23	0.293662	0.14	1	727	5524
nucleotide binding	1.15	0.317704	0.13	1	795	166
cell part	1.06	0.345153	0.08	2	2628	44464
cellular_component	1.06	0.346662	0.08	2	2635	5575
DNA binding	1.00	0.367134	0.11	1	940	3677
transferase activity	0.96	0.381646	0.10	1	984	16740
protein binding	0.85	0.42541	0.09	1	1121	5515
cytoplasm	0.72	0.488981	0.07	1	1333	5737

Supplementary Table 2. List of Enriched pathways with p-value <0.05.

Growth Condition			***Venn categorization***	***Pathway Name***	***Enrichment Score***	***Enrichment p-value***	***genes in list, in pathway***
Unrestricted DO condition	Up-regulated 1564	1208	only in the culture at Unrestricted DO condition	Pentose and glucuronate interconversions	7.6	0.0005075	8
				Fructose and mannose metabolism	5.7	0.00342227	8
				Pyruvate metabolism	3.8	0.0234013	8
				Phosphotransferase system (PTS)	3.7	0.0251226	7
				Ascorbate and aldarate metabolism	3.7	0.0255347	4
				Microbial metabolism in diverse environments	3.5	0.0302282	24
				Sulfur relay system	3.5	0.0311249	4
				Butanoate metabolism	3.4	0.0336619	6
		349	shared with the up-regulated DEGs at restricted DO condition	Geraniol degradation	7.2	0.00071069	3
				beta-Alanine metabolism	7.2	0.00075175	5
				Phosphotransferase system (PTS)	6.0	0.00241965	8
				Fatty acid metabolism	5.4	0.00441847	5
				Fructose and mannose metabolism	5.3	0.00483887	7
				Fatty acid degradation	5.0	0.00661143	4
				Arginine and proline metabolism	4.6	0.0100884	5
				Citrate cycle (TCA cycle)	4.3	0.0142528	5
				Benzoate degradation	4.1	0.016538	3
				Valine, leucine and isoleucine degradation	4.1	0.016538	3
				Pyruvate metabolism	3.6	0.0265654	7
				Carbon metabolism	3.3	0.0364298	11
				Biosynthesis of unsaturated fatty acids	3.2	0.0423621	2

		7	shared with the downregulated DEGs at restricted DO condition				
	Down -regulated	1066	only in the culture at Unrestricted DO condition	Ribosome	26.3	3.73E-12	46
				Aminoacyl-tRNA biosynthesis	17.1	3.84E-08	19
				Biosynthesis of amino acids	8.5	0.0002043	44
				Flagellar assembly	6.6	0.00135541	11
				2-Oxocarboxylic acid metabolism	5.8	0.00296912	13
				RNA polymerase	5.8	0.00313748	4
				Oxidative phosphorylation	4.8	0.00820193	17
				Arginine biosynthesis	4.8	0.00836002	9
				Pantothenate and CoA biosynthesis	4.3	0.013868	11
				Biosynthesis of antibiotics	4.3	0.0141828	61
				Vitamin B6 metabolism	4.2	0.0149629	6
				Terpenoid backbone biosynthesis	4.0	0.0179197	7
				Protein export	3.9	0.0200795	9
				Histidine metabolism	3.8	0.0216185	5
				Cationic antimicrobial peptide (CAMP) resistance	3.8	0.0225407	14
				Cysteine and methionine metabolism	3.7	0.0255692	12
				Valine, leucine and isoleucine biosynthesis	3.5	0.0293519	8
				Phenylalanine, tyrosine and tryptophan biosynthesis	3.2	0.0405213	9

				Novobiocin biosynthesis	3.1	0.0437891	3
		508	shared with the downregulated DEGs at restricted DO condition	Glycerophospholipid metabolism	4.88338	0.0075714	5
				Glycerolipid metabolism	4.38819	0.0124232	3
				Purine metabolism	3.50082	0.0301726	8
				Alanine, aspartate and glutamate metabolism	3.21704	0.0400736	4
				Pyrimidine metabolism	2.96674	0.0514707	6
				Other glycan degradation	2.09638	0.1229	1
				Dioxin degradation	1.82968	0.160465	1
				Xylene degradation	1.82968	0.160465	1
				Glyoxylate and dicarboxylate metabolism	1.51765	0.219226	3
				Biosynthesis of secondary metabolites	1.36197	0.256156	15
				Nitrotoluene degradation	1.33214	0.263912	1
				Nitrogen metabolism	1.2953	0.273816	2
				Methane metabolism	1.23785	0.290006	2
				Lipopolysaccharide biosynthesis	1.13242	0.322251	2
				Benzoate degradation	1.03623	0.354791	1
				Lysine degradation	0.96088	0.382556	1
				Galactose metabolism	0.953069	0.385556	2
				Bacterial secretion system	0.953069	0.385556	2
				Starch and sucrose metabolism	0.840701	0.431408	2
				One carbon pool by folate	0.778736	0.458986	1
				Glycine, serine and threonine metabolism	0.743485	0.475454	2
				Fructose and mannose metabolism	0.713985	0.489689	2
				RNA degradation	0.683823	0.504684	1
				Bacterial chemotaxis	0.683823	0.504684	1
		44	shared with the up-	Citrate cycle (TCA cycle)	19.7739	2.58E-09	8
				Carbon metabolism	10.4679	2.84E-05	9

			regulated DEGs at restricted DO condition	Biosynthesis of antibiotics	6.96595	0.00094347	10
				Oxidative phosphorylation	5.45561	0.00427227	4
				2-Oxocarboxylic acid metabolism	4.74824	0.00866693	3
				Microbial metabolism in diverse environments	4.38348	0.0124819	9
				Lysine degradation	4.28046	0.0138363	2
				C5-Branched dibasic acid metabolism	4.28046	0.0138363	2
				Fatty acid biosynthesis	3.95186	0.0192189	2
				Propanoate metabolism	3.70559	0.0245858	3
				beta-Lactam resistance	3.32991	0.0357963	2
				Ribosome	3.27746	0.0377238	4
				Fatty acid metabolism	3.13397	0.0435445	2
				Biosynthesis of secondary metabolites	3.12913	0.0437559	9
Restricted DO condition	Up-regulated 547	154	only in the culture at restricted DO condition	Oxidative phosphorylation	6.4	0.00164033	6
				Citrate cycle (TCA cycle)	3.0	0.0498283	3
				Carbon metabolism	3.0	0.0515864	7
				ABC transporters	2.7	0.0676333	9
				Pyruvate metabolism	2.5	0.0853883	4
				Butanoate metabolism	2.2	0.109327	3
				D-Alanine metabolism	2.1	0.124152	1
				Novobiocin biosynthesis	2.1	0.124152	1
				Protein export	2.1	0.125029	2
				Microbial metabolism in diverse environments	2.0	0.140446	11
				Nitrotoluene degradation	1.6	0.207233	1
				Monobactam biosynthesis	1.5	0.233172	1

				Tryptophan metabolism	1.5	0.233172	1
				Vancomycin resistance	1.5	0.233172	1
				Bacterial secretion system	1.3	0.267655	2

				Galactose metabolism	1.3	0.267655	2
				Two-component system	1.2	0.301532	6
				Cationic antimicrobial peptide (CAMP) resistance	1.1	0.316785	2
				Lysine biosynthesis	1.0	0.350905	1
				Biosynthesis of secondary metabolites	1.0	0.351096	11
				Glyoxylate and dicarboxylate metabolism	1.0	0.353228	2
				Glycolysis / Gluconeogenesis	0.9	0.400793	2
				Phosphotransferase system (PTS)	0.9	0.412451	2
				Biofilm formation - Escherichia coli	0.9	0.412451	2
				Sulfur relay system	0.8	0.45088	1
				beta-Lactam resistance	0.8	0.45088	1
				Glutathione metabolism	0.8	0.468983	1
				Ubiquinone and other terpenoid-quinone biosynthesis	0.7	0.486501	1
				Phenylalanine, tyrosine and tryptophan biosynthesis	0.7	0.503453	1
				Nicotinate and nicotinamide metabolism	0.7	0.519857	1
				Porphyrin and chlorophyll metabolism	0.6	0.535729	1
		349	shared with the up-regulated DEGs at Unrestricted DO condition	Pathways listed above in Unrestricted DO conditon			

		44	shared with the up-regulated DEGs at Unrestricted DO condition	Pathways listed above in Unrestricted DO conditon			
	Down -regulated	224	only in the culture at restricted DO condition	Ribosome	5.44772	0.00430613	7
				Biosynthesis of siderophore group nonribosomal peptides	4.23261	0.0145145	2
				Two-component system	3.36334	0.0346195	8
				Glycerolipid metabolism	3.17546	0.041775	2
				Biosynthesis of secondary metabolites	3.04738	0.0474831	13
		508	shared with the downregulated DEGs at Unrestricted DO condition	Pathways listed above in Unrestricted DO conditon			
		7	shared with the up-regulated DEGs at Unrestricted DO condition	Pathways listed above in Unrestricted DO conditon			

Supplementary Table 3. Proteomics data with the fold change expression and p-values of the detected proteins.

Protein IDs	Protein names	pvalue_Unrestricted DO(latelog vs. log)	Fold-Change_Unrestricted DO(latelog vs. log)	pvalue_Restricted DO(latelog vs. log)	Fold-Change_Restricted DO(latelog vs. log)
A0A140N2H0	Acetolactate synthase isozyme 1 large subunit	2.43E-09	20.3147	0.094013	1.63759
A0A140NG09	Threonine synthase	2.76E-05	2.15316	1.64E-08	3.26829
A0A140NE69	3-isopropylmalate dehydratase large subunit	9.75E-09	6.13818	0.00714344	1.41729
A0A140N8Q5	Galactitol-specific phosphotransferase enzyme IIA component	0.00293229	-1.51707	0.000161785	1.27554
A0A140NC35	Bifunctional aspartokinase/homoserine dehydrogenase 1;Aspartokinase;Homoserine dehydrog	0.00015586	2.27832	6.94E-08	4.12194
A0A140NGU5	Acetyl-coenzyme A synthetase	8.27E-08	12.0774	1.83E-05	6.47779
D9IX93	Glutathione-binding protein GsiB	0.129471	1.17039	2.08E-06	1.7062
A0A140N6V3	N-ethylmaleimide reductase	0.00843976	1.17928	1.98E-05	1.30633
A0A140NDI3	Uncharacterized protein YjiM	0.732792	1.76963	2.43E-07	16.837
A0A140N8Z3	HTH-type transcriptional regulator IscR	1.84E-05	-1.53907	0.00153291	-1.68133
A0A140NFA8	Acyl-coenzyme A dehydrogenase	1.71E-07	12.5601	0.000415059	4.10884
A0A140NBI6	Hydroxyacylglutathione hydrolase	0.291652	1.10281	6.82E-07	2.0388
A0A140N490	Glycogen synthase	5.92E-05	1.84566	2.41E-06	1.76156
A0A140NFC3	Inner membrane protein YagU	0.0836811	1.12197	7.66E-07	1.78831
A0A140NE26	Type I restriction enzyme EcoKI M protein	4.12E-06	-3.85538	1.91E-06	-3.83447
A0A140N6M9	Glycogen phosphorylase	0.00101562	1.55529	6.38E-06	1.70998
A0A140N6E5	D-tagatose-1,6-bisphosphate aldolase subunit GatZ	0.250905	-1.24176	6.28E-05	1.62514
A0A140N6Z3		1.69E-05	2.52747	2.33E-05	1.67661
A0A140N508	Bacterioferritin	0.00239694	2.84158	2.90E-06	3.0002

A0A140NC64	Inner membrane protein YlaC	2.77E-06	-5.17215	2.04E-05	-2.12064
A0A140N4W8	sn-glycerol-3-phosphate-binding periplasmic protein UgpB	8.65E-07	7.27	0.00251072	2.63186

A0A140N3K4	Uncharacterized protein YicH	6.90E-05	1.43824	5.05E-05	1.32044
A0A140NDZ1	Pyruvate dehydrogenase [ubiquinone];Alphapeptide	0.00978476	2.45123	1.30E-05	2.29071
A0A140N991	Transaldolase A	0.0469605	2.00877	3.85E-06	3.3868
A0A140NBF7	Catalase HPII	0.599877	1.24091	1.05E-05	2.77419
A0A140NAN3	Isocitrate dehydrogenase [NADP]	2.39E-06	1.6421	2.33E-05	1.44042
A0A140ND02	Periplasmic oligopeptide-binding protein	9.99E-07	5.30815	0.000597558	1.99768
A0A140N4A1	Mannitol-1-phosphate 5-dehydrogenase	0.798637	1.03944	6.63E-06	2.17302
A0A140N6N9	4-alpha-glucanotransferase	0.000634846	-1.8667	2.99E-05	-1.69652
A0A140N9M6	Glutamate/aspartate periplasmic-binding protein	2.28E-06	9.73431	7.79E-05	3.96199
A0A140N935	Uncharacterized protein YgaU	0.00692245	2.28161	4.59E-06	3.17264
A0A140NBT9	Cell division protein FtsA	0.00832994	1.13652	0.000123818	1.21649
A0A140N312	Universal stress protein A	2.73E-05	2.50178	4.97E-05	1.69262
A0A140NBJ1	Uncharacterized protein YccJ	0.422374	1.43316	1.30E-05	3.13032
A0A140N9S7	Anaerobic glycerol-3-phosphate dehydrogenase subunit B	0.0711969	1.34873	0.533496	-1.04076
A0A140SS89	Fatty acid oxidation complex subunit alpha;Enoyl-CoA hydratase/Delta(3)-cisDelta(2)-tr	2.90E-06	14.915	0.00159815	6.03833
A0A140N937	HTH-type transcriptional repressor RspR	0.00407496	-1.26702	8.73E-06	1.57069
A0A140N7N3	Glutamate synthase [NADPH] large chain	0.00367113	1.51595	1.10E-05	1.89368
A0A140SS32	Divalent-cation tolerance protein CutA	0.0256569	1.40814	1.21E-05	2.01962

A0A140NAX3	NADH dehydrogenase	0.096863	-1.45439	0.000169267	1.68769
A0A140N6G0	Enolase	0.0589339	-1.19605	9.68E-05	1.4419
A0A140NAI9	Uncharacterized oxidoreductase YbdH	3.75E-06	18.5134	0.0751307	3.32072
A0A140NEW5	Aromatic-amino-acid aminotransferase	0.000578999	1.47761	8.57E-05	1.42905
A0A140NF74	Bifunctional aspartokinase/homoserine dehydrogenase 2;Aspartokinase;Homoserine dehydrog	2.26E-06	2.72526	0.325482	1.11459
A0A140SS93	Putative carboxymethylenebutenolidase	5.89E-06	4.97381	9.37E-05	2.59789

A0A140NAC0	Fructokinase	0.0386683	1.26427	0.000210101	1.4278
A0A140NF92	Universal stress protein D	0.0385144	1.28842	7.52E-06	2.04084
A0A140N822	Cytidine deaminase	0.0839435	-1.35907	5.65E-06	2.78871
A0A140NBC5	NADP-specific glutamate dehydrogenase	2.85E-06	3.98143	0.00210689	1.78565
A0A140N8D8	Histidinol-phosphate aminotransferase	4.57E-06	3.24125	0.000283124	1.90483
A0A140N801	Glutathione synthetase	0.134634	1.18421	7.33E-05	1.56798
A0A140NCB1	Quinone oxidoreductase 1	0.111484	1.48994	4.24E-05	2.13966
A0A140NA96	Probable protein deglycase	0.252399	1.09539	8.93E-05	1.42698
A0A140SSA6	Xaa-Pro dipeptidase	0.000363275	1.43818	0.000311854	1.30636
A0A140N781	Arabinose 5-phosphate isomerase GutQ	0.857162	1.01901	0.000219735	1.45217
A0A140N953	Uncharacterized protein YecA	0.113711	1.20018	2.45E-05	1.76839
A0A140N9P4	Dual-specificity RNA methyltransferase RlmN	3.77E-05	-1.88178	3.55E-05	-2.13077
A0A140NBI8	Uncharacterized protein YeaK	0.131578	1.14279	3.15E-05	1.59028
A0A140N5N0	Uncharacterized N-acetyltransferase YhbS	0.000498319	1.71506	4.32E-05	1.7076
A0A140NFZ3	Uncharacterized protein YjjU	0.621392	1.04462	0.176353	1.07708
A0A140NE48	Isoaspartyl dipeptidase	0.537074	1.38424	3.50E-05	3.19997
A0A140N3B4	Uronate isomerase	0.0021169	2.48712	2.05E-05	2.72964

A0A140NB21		0.0865826	-1.17844	0.00225561	1.25788
A0A140N775	Malate dehydrogenase	5.22E-06	1.95414	0.00288044	1.28314
A0A140NB56	Glucose-1-phosphatase	0.0976415	1.60665	7.05E-05	2.20873
A0A140N7T0	Uncharacterized protein YhbW	0.069388	1.28076	0.000724349	1.41491
A0A140N9J3	Sugar phosphatase YfbT	0.0138121	1.30788	8.65E-05	1.52563
A0A140N9V4	Malate:quinone oxidoreductase	3.06E-05	-2.22167	7.43E-05	-1.93723
A0A140N782		0.0163377	2.31931	0.00276989	1.48996
A0A140N6N2	Glycerol-3-phosphate dehydrogenase [NAD(P)+]	0.932823	-1.00431	0.151077	1.05826
A0A140N802	Glycerophosphoryl diester phosphodiesterase	0.0281292	1.45426	0.000318404	-1.56872
A0A140N987	Uncharacterized protein YeaC	2.51E-05	-1.99988	0.0245958	1.17169
A0A140N870	Galactitol-1-phosphate 5-dehydrogenase	0.0529112	-1.90882	0.0149288	1.32736
A0A140NDN9	DNA protection during starvation protein	0.0030181	2.07565	4.16E-05	2.26298

A0A140NAH0	Fructose-bisphosphate aldolase class 1	0.165741	1.72689	5.15E-05	2.80627
A0A140N7V2	ATP phosphoribosyltransferase	8.09E-06	3.14906	0.00680923	1.57451
A0A140N7Q4	Polyphosphate kinase	0.368279	1.0821	3.55E-05	1.63582
A0A140NAC2	Galactose/methyl galactoside import ATPbinding protein MglA	0.00939136	1.61388	0.000265141	1.62136
A0A140NC19	Putative GTP cyclohydrolase 1 type 2	0.00156563	1.46815	0.00108816	1.31502
A0A140NAD7	Peroxiredoxin OsmC	0.00901235	2.92024	7.71E-05	2.57935
A0A140N914	Putative NAD(P)H nitroreductase YdjA	0.0179916	1.34144	0.000183061	1.52256
A0A140NCM6	3(2),5-bisphosphate nucleotidase CysQ	0.000125895	1.83956	0.000341668	1.47235
A0A140N3X6	Periplasmic dipeptide transport protein	1.30E-05	12.1438	0.0221175	3.16446
A0A140SS73	Glycerol kinase	7.56E-05	2.16527	0.307018	1.07343
A0A140NC46	N-acetylglucosamine-6-phosphate deacetylase	0.00101879	1.38308	0.00054724	1.30507

A0A140NAV3	Copper-exporting P-type ATPase A	0.481702	-1.09316	0.00139656	1.38099
A0A140NF10	Dihydroxy-acid dehydratase	8.22E-06	2.80902	0.150171	1.20454
A0A140NBD3	Phospho-2-dehydro-3-deoxyheptonate aldolase, Phe-sensitive	0.0039083	1.47924	0.000409993	1.44532
A0A140NAB8	Glutaredoxin-2	0.169009	1.44514	6.47E-05	2.41307
A0A140NCJ4	D-amino acid dehydrogenase	0.00864463	1.54228	1.16E-05	2.94978
A0A140NCH2	Oligopeptide transport ATP-binding protein OppF	1.10E-05	3.05608	0.00466697	1.55276
A0A140NGH9	Deoxyribose-phosphate aldolase	0.0466372	-1.4172	3.62E-05	2.1897
A0A140NFP9	Aconitate hydratase B	1.53E-05	1.48206	0.00380915	1.20073
A0A140NFW2	UPF0294 protein YafD	0.0797168	-1.31165	0.000149733	1.71246
A0A140NES2	7-cyano-7-deazaguanine synthase	0.524762	1.06714	0.000104079	1.59186
A0A140N8M5	Oligopeptide transport ATP-binding protein OppD	1.15E-05	3.19795	0.0156547	1.45709
A0A140N9F7	Protein YdgA	0.00109184	1.2887	0.0357648	1.10316
A0A140ND38	L-threonine dehydratase biosynthetic IlvA	1.05E-05	2.46569	0.0424897	1.26288
A0A140N6L4	Imidazole glycerol phosphate synthase subunit HisF	2.52E-05	2.19329	0.000775284	1.55528
A0A140NE95	Regulator of nucleoside diphosphate kinase	0.744969	1.03333	0.803038	-1.01493

A0A140NEL5	Triosephosphate isomerase	0.987205	-1.00168	0.000471171	1.43903
A0A140N510	Phosphoenolpyruvate carboxykinase [ATP]	9.39E-05	1.97459	0.00180221	1.3673
A0A140N5V3	Uncharacterized oxidoreductase YgjR	0.442372	1.15346	0.000189031	1.83032
A0A140NCI1	PTS-dependent dihydroxyacetone kinase, ADPbinding subunit DhaL	0.175009	1.15365	0.000474758	-1.42312
A0A140NEB3	Methionine aminotransferase	2.41E-05	10.249	0.136431	2.48119

A0A140NDI4	Phosphoserine aminotransferase	0.022985	1.29009	0.000192642	1.50506
A0A140NCJ7	Argininosuccinate lyase	0.000567937	1.63802	0.718973	-1.02189
A0A140NDQ3	3-isopropylmalate dehydratase small subunit	1.91E-05	5.89741	0.119345	1.62992
A0A140N6N1	Aerobic glycerol-3-phosphate dehydrogenase	0.45094	-1.07792	0.00604598	1.24389
A0A140NHP9	D-ribose-binding periplasmic protein	0.000425926	2.3575	0.0129687	1.29693
A0A140NC10	Citrate synthase	2.16E-05	3.05201	0.0252249	1.54909
A0A140NEC9	Homoserine O-succinyltransferase	2.50E-05	4.81984	0.0107578	2.23215
A0A140N8F0	Long-chain-fatty-acid--CoA ligase	2.80E-05	3.70595	0.00784963	2.06202
A0A140NAD8	Pyrimidine-specific ribonucleoside hydrolase RihA	0.0575904	1.21233	0.148397	-1.09228
A0A140N7B0	Acetylornithine/succinyldiaminopimelate aminotransferase	0.0691975	1.16262	0.000686322	1.31851
A0A140NBQ0	Glutathione import ATP-binding protein GsiA	0.954992	-1.01042	0.000243291	1.81227
A0A140NB26	Uncharacterized HTH-type transcriptional regulator YdjF	0.00143962	1.38121	0.00125818	1.2812
A0A140NA64	D-tagatose-1,6-bisphosphate aldolase subunit GatY	0.150699	-2.06964	0.723452	-1.04163
A0A140N813	Agmatinase	0.723082	-1.02288	0.000798118	1.27054
A0A140ND42	NAD/NADP-dependent betaine aldehyde dehydrogenase	7.34E-05	1.52999	0.0181277	1.23646
A0A140SS53	Peptidase E	0.239337	1.20201	0.0342808	1.21026
A0A140NG25	Inner membrane protein YjiY	0.00761416	-2.23949	5.45E-05	3.03499
A0A140N714	Uncharacterized protein YebG	0.000251431	-1.95812	0.00258456	-1.37309
A0A140N9B6	Succinylornithine transaminase	3.49E-05	22.1155	0.0749183	5.08943
A0A140NEZ0	Cation efflux system protein CusF	0.00244565	-2.17656	0.000215137	-1.96617

A0A140ND68	Aspartate aminotransferase	0.282427	-1.09952	7.79E-05	1.58543
A0A140NBF2	PTS system mannose-specific EIIAB component;Mannose-specific phosphotransferase enzyme	0.211088	1.36041	0.000239892	2.00352
A0A140N459	PanD maturation factor	0.61584	-1.04078	0.000122	1.48929
A0A140SS95		2.87E-05	7.33188	0.0160044	2.4878
A0A140NAZ8	Polyamine aminopropyltransferase	2.77E-05	1.91314	0.562052	1.0572
A0A140NC71	Uncharacterized protein YbiB	0.014582	1.40911	0.000358277	1.54568
A0A140N934	3-mercaptopyruvate sulfurtransferase	0.00030183	1.86488	0.0012429	1.43642
A0A140NE56	Mannonate dehydratase	0.00093467	2.11305	0.000383016	1.75308
A0A140N7W7	Bifunctional glutathionylspermidine synthetase/amidase;Glutathionylspermidine amidase;G	0.00938825	-1.42948	0.000140682	1.68022
A0A140N6Q3	Glycine betaine-binding periplasmic protein	2.59E-05	3.4689	0.0776295	-1.23614
A0A140N5V9	L-glyceraldehyde 3-phosphate reductase	0.131802	1.44473	0.000231678	2.0475
A0A140N8R4	Cell division protein ZapA	0.336572	1.08124	0.000657536	1.34524
A0A140NDA3	Protein BolA	1.99E-05	4.14568	0.299689	1.20246
A0A140NDB2	Uncharacterized protein YbjX	7.96E-05	-2.20082	0.000660657	-1.77336
A0A140N6F9	UPF0339 protein YegP	0.00488761	5.49022	7.88E-05	5.26348
A0A140NBL1	Ribonucleotide monophosphatase NagD	0.0995232	1.09248	0.0190779	1.11463
A0A140SS69	Glycerol dehydrogenase	0.010943	1.57346	0.000746483	1.56281
A0A140N9C2	Thiosulfate-binding protein	0.0145378	-1.20395	0.0142382	1.15008
A0A140N8X2	Transcriptional regulatory protein PhoP	0.00211758	1.36095	0.000287891	1.40743
A0A140NDL0	Pyruvate dehydrogenase E1 component	0.0150298	-1.56647	0.000925533	1.55573
A0A140NFD2	D-methionine-binding lipoprotein MetQ	4.28E-05	3.23663	0.0039382	1.70635
A0A140N753	Maltodextrin phosphorylase	0.00161403	-2.00158	0.000934424	-1.60758

A0A140N9A3	Cyclopropane-fatty-acyl-phospholipid synthase	0.000935362	-3.14953	0.021061	-1.34972
A0A140NBG3	Esterase FrsA	0.0393617	-1.21783	0.00040768	1.41465
A0A140NE55	D-allose-binding periplasmic protein	0.139114	1.40457	0.012094	1.36265
A0A140NCK8	Aconitate hydratase A	0.000121379	2.61309	0.000945058	1.82741
A0A140NFA2	Protein YiiM	0.046793	1.23774	0.00630786	1.25257
A0A140N5W6	Histidinol dehydrogenase	3.86E-05	2.94387	0.0185775	1.46305
A0A140N6D7	6-phospho-beta-glucosidase BglA	0.812779	1.02018	0.00467084	1.24571
A0A140N8N0	S-ribosylhomocysteine lyase	0.321821	1.12619	0.000341143	1.57696
A0A140NDC1	UPF0227 protein YcfP	0.134409	1.22638	0.000608208	1.54099
A0A140NA63	Maltose O-acetyltransferase	0.380117	-1.133	0.000764866	-1.54635
A0A140NF85	Putative glutamine amidotransferase YafJ	0.0640148	1.231	0.000105418	1.70808
A0A140N821	Fructose-bisphosphate aldolase class 2	0.386374	-1.10701	0.00207461	1.37464
A0A140NFZ6	Osmotically-inducible protein Y	0.000178796	3.34922	0.00197931	1.74507
A0A140NGT4		0.00840682	1.45605	0.00403149	1.33025
A0A140SSB0	Uridine phosphorylase	0.889072	-1.02072	0.000198653	1.83657
A0A140NDA1	Peptidase T	0.0246061	1.58137	0.000778948	1.67157
A0A140NE27	Dihydrolipoyllysine-residue acetyltransferase component of pyruvate dehydrogenase compl	0.0427748	-1.44486	0.00263693	1.46023
A0A140N5E1	Ribulose-phosphate 3-epimerase	0.182767	1.19394	0.000248917	1.693
A0A140NCT2	Fumarate reductase flavoprotein subunit	0.0128699	1.3581	0.00854362	1.25277
A0A140N9Y3	HTH-type transcriptional regulator GalS	0.0781787	2.07086	0.000255789	2.76627
A0A140NHQ0	5,10-methylenetetrahydrofolate reductase	8.08E-05	11.989	0.0495614	3.55347
A0A140NAQ0	Protein mrp	0.0543514	-1.16039	0.0145576	1.16469
A0A140SS67	Phosphoenolpyruvate carboxylase	0.146005	1.22269	0.00297076	1.38824

A0A140N6I7	Protein SseB	0.000279932	-1.35611	0.155301	1.06756
A0A140N730	Ribosome-associated inhibitor A	4.03E-05	3.61211	0.206967	-1.22379
A0A140NAS5	Leucine-responsive regulatory protein	5.49E-05	2.76109	0.0404144	1.31064
A0A140N487	2-amino-3-ketobutyrate coenzyme A ligase	0.108267	1.21584	0.00207412	1.38152
A0A140NA20	PTS-dependent dihydroxyacetone kinase, dihydroxyacetone-binding subunit DhaK	0.114401	1.29021	0.00367467	-1.41192
A0A140N9D5	Cysteine desulfurase IscS	0.0193951	-1.17032	0.00154134	-1.39262
A0A140NAM7	Oligopeptide transport system permease protein OppB	8.47E-05	3.22703	0.00607648	1.7344
A0A140N9N5	Phosphoenolpyruvate-protein phosphotransferase	0.294588	1.06461	0.00334662	1.19822
A0A140NC00	Uncharacterized oxidoreductase YdgJ	0.0520819	1.24561	0.00574097	1.27846
A0A140N7E4	Putative peroxiredoxin bcp	0.0753035	1.21817	0.0712486	1.14118
A0A140N7E7	P-protein;Chorismate mutase;Prephenate dehydratase	0.000105925	2.62271	0.00504671	1.75257
A0A140NFM3	ATP-dependent RNA helicase HrpB	0.00852371	-1.19878	0.00445938	1.18118
A0A140SS84	Acetylornithine deacetylase	0.00793383	1.6998	0.0300131	1.25599
A0A140N9I7	3-ketoacyl-CoA thiolase	0.000102506	6.53034	0.0069228	2.82615
A0A140N8V5	CDP-diacylglycerol--serine Ophosphatidyltransferase	0.0379296	1.16264	0.0369774	1.12255
A0A140N7A3	Lipopolysaccharide export system ATP-binding protein LptB	0.310833	1.07293	0.00679963	1.19448
A0A140NBK8	Glutathione S-transferase GstB	0.0508316	1.34954	0.000197494	1.86105
A0A140N7T9	Cystathionine beta-lyase MetC	0.000578414	2.30902	0.000645183	1.91764
A0A140NFQ2	HTH-type transcriptional regulator HdfR	0.941346	-1.00966	0.00542708	1.34245
A0A140N3I5	2,5-diketo-D-gluconic acid reductase A	0.000689122	3.38042	0.000510266	2.54592

A0A140N8N2	Bacterial non-heme ferritin	0.666009	1.05713	0.00105456	1.50072
A0A140NDS5	Hydrolase YafV	0.0481587	1.27745	0.00311764	1.34768
A0A140N899	Glucose-6-phosphate 1-dehydrogenase;Extracellular death factor	0.0142846	1.25066	0.0961362	1.10432
A0A140NBM8	Oxidoreductase YdhF	0.706968	1.05995	0.114919	1.15655
A0A140N5B5	Alcohol dehydrogenase YqhD	0.0466777	1.39033	0.000658756	1.64802
A0A140N9M9	1,4-dihydroxy-2-naphthoyl-CoA synthase	0.0668721	-1.18187	0.00249454	1.28876
A0A140NDU1	Non-specific ribonucleoside hydrolase RihC	0.775409	1.07697	0.00677687	1.50599
A0A140N5L9	Cysteine synthase A	0.764636	-1.04685	0.00179008	1.50739
A0A140N9N9	Osmotically-inducible lipoprotein E	0.00333031	2.47789	0.00123121	1.85494
A0A140NC79	Cold shock-like protein CspE	0.00143685	-1.45223	0.000525651	-1.45734
A0A140NDY3	Soluble lytic murein transglycosylase	0.012477	1.29644	0.0042645	1.26819
A0A140NFT0	Ferredoxin--NADP reductase	0.15609	1.20993	0.00173865	1.44663
A0A140N3Y7	Protein tas	0.0519033	1.42505	0.00299373	1.47544
A0A140NBC2	Inner membrane protein YebE	0.00505687	1.81453	0.000311192	2.01939
A0A140SS66	Uncharacterized protein YjbR	0.318733	1.28512	0.000367257	2.19577

A0A140NAI0	Peptide chain release factor 1	0.249879	1.09887	0.000204053	1.49388
A0A140N8B5	NMN amidohydrolase-like protein YfaY	0.0358229	1.19823	0.914973	1.00575
A0A140NDB1	Cytosol non-specific dipeptidase	0.397498	1.10651	0.0041535	1.34367
A0A140NHP0	Branched-chain-amino-acid aminotransferase	8.45E-05	2.71237	0.668466	1.07587
A0A140N9U3	Uncharacterized protein YdiJ	0.000895375	2.15982	0.000627546	1.94736
A0A140NC83	Transcriptional regulatory protein TyrR	0.0748439	1.21419	0.00155417	1.38036
A0A140NC63	Probable TonB-dependent receptor YncD	0.00555484	-1.40922	0.00106615	-1.42321
A0A140N8E8	GTP pyrophosphokinase	0.0150755	1.30339	0.00240656	1.32383

A0A140N4G4	Glycine dehydrogenase (decarboxylating)	0.873191	-1.0261	0.449511	-1.06991
A0A140SSA7	Acetolactate synthase isozyme 2 large subunit	7.55E-05	1.92966	0.267605	-1.11775
A0A140N3P8	Aerobic respiration control sensor protein ArcB	0.678894	1.04531	0.0180922	1.22512
A0A140N7K6	Ribonuclease G	0.289536	1.10606	0.000680332	1.43526
A0A140NFW0	6-phosphogluconate phosphatase	0.0320367	1.36092	0.00180129	1.45885
A0A140NBB5	Aldehyde reductase YahK	0.141656	1.67835	0.00190283	1.9029
A0A140N5V0	Glucarate dehydratase	0.740421	1.04998	0.00210205	1.49221
A0A140N4Y8	Protein RecA	0.126289	1.1349	0.00289537	1.2708
A0A140NE58	Isoaspartyl peptidase;Isoaspartyl peptidase subunit alpha;Isoaspartyl peptidase subunit	0.501032	1.06694	0.0200044	1.20276
A0A140N5Y3	Multifunctional CCA protein;CCA-adding enzyme;2-nucleotidase;2,3-cyclic phosphodiestera	0.738841	1.021	0.0057003	1.19375
A0A140NCD4	Universal stress protein G	0.000176195	3.37447	0.00989659	1.8845
A0A140NGD0	Trehalose-6-phosphate hydrolase	0.00124711	-2.56942	0.00166913	-1.87681
A0A140N420	L-fucose isomerase	0.000113235	8.35686	0.179522	-1.30305
A0A140N551	Protein lysine acetyltransferase Pka	0.0257842	1.76724	0.00161471	1.76889
A0A140NC74	Universal stress protein E	0.000850643	2.1549	0.010133	1.41052
A0A140N989	ATP-dependent RNA helicase SrmB	0.421924	1.05343	0.000568266	1.32741
A0A140N9Z6	Lactaldehyde dehydrogenase	0.000361405	4.94896	0.00280688	2.70226
A0A140N5X8	Sulfate adenylyltransferase subunit 2	0.000129711	-4.21824	0.120884	-1.29585
A0A140NEG9	Acetylglutamate kinase	0.00131717	1.9477	0.459804	1.07125
A0A140NE23	Asparagine synthetase B [glutaminehydrolyzing]	0.000426082	3.54761	0.00336116	1.98671
A0A140N9I3	Anti-sigma-E factor RseA	0.200923	-1.11065	0.00321421	1.27124

A0A140N8Q7		0.000239342	4.19984	0.822913	-1.03087
A0A140N619	Anaerobic glycerol-3-phosphate dehydrogenase subunit A	0.294504	1.34973	0.615702	1.06121
A0A140N4F1	ADP-heptose--LPS heptosyltransferase 2	0.0081891	-1.28106	0.000738469	1.37911
A0A140N9V3	Glutamine-binding periplasmic protein	0.000714258	2.98743	0.266191	1.14809
A0A140NB24	Uncharacterized oxidoreductase YajO	0.0429651	1.60703	0.00185398	1.70752
A0A140N6Z4	Isochorismatase family protein YecD	0.0441382	1.32431	0.00203918	1.45294
A0A140NDX8	GTPase HflX	0.0550207	1.12659	0.213464	1.05899
A0A140N852	NADPH-dependent curcumin reductase	0.425932	1.48367	0.00103834	2.54908
A0A140NDY8	Phosphatidylserine decarboxylase proenzyme;Phosphatidylserine decarboxylase alpha chain	0.0033683	1.2357	0.176508	1.0651
A0A140NDT0	Aminopeptidase N	0.0263323	1.32238	0.0008652	1.50963
A0A140NCT8	PTS-dependent dihydroxyacetone kinase, phosphotransferase subunit DhaM;Phosphocarrier p	0.198544	-1.10078	0.00483263	-1.22938
A0A140NB50	Cell division topological specificity factor	0.0342045	1.31592	0.00125391	1.48063
A0A140NGI3	Probable L-ascorbate-6-phosphate lactonase UlaG	0.0203452	-2.38959	0.728681	1.04539
A0A140N479	DNA mismatch repair protein MutS	0.005588	-1.21096	0.00187859	-1.22534
A0A140NH97	Sensor protein BasS	0.000823785	-2.03677	0.0132302	-1.36845
A0A140N7Z9	2,4-dienoyl-CoA reductase [NADPH]	0.000221539	8.17846	0.0337651	2.73456
A0A140NDT7	HTH-type transcriptional regulator MetR	0.000239792	4.05451	0.129736	1.71658
A0A140N655	Glycine betaine/L-proline transport ATP-binding protein ProV	0.000297383	2.59558	0.0070189	-1.6656
A0A140N8Q2	3-dehydroquinate dehydratase	0.0815799	1.24996	0.00171191	1.45442
A0A140N6V7	Uracil-DNA glycosylase	0.0075665	1.19334	0.0912938	-1.08252

A0A140NCL5	Periplasmic trehalase	0.130672	2.2123	0.00193345	2.26133
A0A140N788	Glutamate synthase [NADPH] small chain	0.0654597	1.5048	0.00114978	1.81793
A0A140N901	L-fucose mutarotase	0.000595089	2.2787	0.00857508	1.488
A0A140N4Y5	Aspartate-semialdehyde dehydrogenase	0.00143613	1.90196	0.0135885	1.35589
A0A140N4S8	Outer membrane protein assembly factor BamC	0.0123547	1.24631	0.139252	1.09118
A0A140N9Q4	Ribonucleoside-diphosphate reductase 1 subunit alpha	0.0985932	1.11243	0.000173568	-1.48596
A0A140N6R6	Glyoxylate/hydroxypyruvate reductase B	0.0179561	1.42492	0.00864307	1.33167
A0A140N7B3	4-aminobutyrate aminotransferase GabT	0.000616052	3.59192	0.00617896	1.83409
A0A140N725	Phosphoadenosine phosphosulfate reductase	0.000453411	-1.97768	0.00805046	-1.43614
A0A140N4B0	L-seryl-tRNA(Sec) selenium transferase	0.0522758	1.25793	0.00054381	1.58039
A0A140NEF7	Phosphopentomutase	0.0414992	-1.33931	0.00133415	1.53818
A0A140N7U0	Uncharacterized protein YraP	0.0012693	1.50757	0.185977	1.09981
A0A140NG27	Lipoate-protein ligase A	0.0135879	1.44177	0.0835866	1.16817
A0A140N201	PTS system mannitol-specific EIICBA component;Mannitol permease IIC component;Mannitol-	0.00333756	2.06816	0.00150523	1.87498
A0A140NAD1	Histidine biosynthesis bifunctional protein HisIE;Phosphoribosyl-AMP cyclohydrolase;Pho	0.000630784	2.06955	0.00780831	1.4724
A0A140NAT9	Flagellar hook protein FlgE	0.000345634	-39.3814	0.378747	-3.08
A0A140N5U8	Lysine/arginine/ornithine-binding periplasmic protein	0.000391248	11.5136	0.0678143	5.28912
A0A140NA98	Lipopolysaccharide assembly protein B	0.585244	1.06779	0.00182213	1.46253
A0A140NBN1	NADP-dependent 3-hydroxy acid dehydrogenase YdfG	0.676	1.06826	0.000433701	1.92944
A0A140NCD0	Maltose-binding periplasmic protein	0.0277891	-2.01165	0.0221619	-1.43894

A0A140NGF5	Uncharacterized protein YjgR	0.0316748	1.62391	0.00311473	1.6354
A0A140N9W3	Uncharacterized oxidoreductase YbiC	0.0120791	1.59263	0.0011538	1.7249
A0A140NBF0	Superoxide dismutase [Fe]	0.725956	-1.0458	0.000309571	1.85877
A0A140NB08		0.352446	1.19479	0.00200515	1.67268
A0A140N5R4	Probable acrylyl-CoA reductase AcuI	0.258811	1.21123	0.00695925	1.42875
A0A140NCE4	Aldehyde-alcohol dehydrogenase;Alcohol dehydrogenase;Acetaldehyde dehydrogenase [acetyl	0.180578	-1.49072	0.000786712	2.38761
A0A140N762	Arginine repressor	0.641332	1.05863	0.4803	1.05807
A0A140N9X2	Inner membrane protein YejM	0.171576	1.18328	0.00169324	1.47428
A0A140NF71	HTH-type transcriptional regulator YjdC	0.253349	1.32123	0.00124241	1.97411
A0A140NDQ1	Xanthine phosphoribosyltransferase	0.000208321	-1.57381	0.8031	-1.01696
A0A140N893	D-lactate dehydrogenase	0.338716	-1.23737	0.00112493	1.94188
A0A140N6V1	FKBP-type peptidyl-prolyl cis-trans isomerase SlyD	0.517685	-1.08493	0.0310927	1.23301
A0A140N8F6	HTH-type transcriptional regulator ArgP	0.000495431	1.61765	0.0262952	1.22882
A0A140NGB9	Ornithine carbamoyltransferase chain I	0.00028579	2.31009	0.360216	1.11242
A0A140N8R5	LOG family protein YgdH	0.114236	1.21805	0.502715	-1.05381
A0A140N5I2	NADP-dependent malic enzyme	0.000729027	2.38266	0.0209443	1.42651
A0A140N784	3-dehydroquinate synthase	0.240544	1.1666	0.000700723	1.66668
A0A140NFF5	Penicillin-binding protein 1B;Penicillininsensitive transglycosylase;Penicillin-sensit	0.709449	1.01866	0.00407676	1.177
A0A140NI62	Lipopolysaccharide biosynthesis protein WzzE	0.0637787	-1.12145	0.002502	-1.2209
A0A140NC67	Phosphoglucomutase	0.0391845	1.28812	0.01101	1.28292

A0A140N9W2	Voltage-gated potassium channel Kch	0.0738988	1.28221	0.00398904	1.41744
A0A140N6A0	Probable lipoprotein YiaD	0.179143	1.07281	0.000490212	-1.34716
A0A140N6I3		0.121399	1.29879	0.74472	-1.03108
A0A140ND59		0.0009739	4.51242	0.012966	1.7967
P00935	Cystathionine gamma-synthase	0.000437058	7.62246	0.137461	2.27432
A0A140N6C7	2-dehydro-3-deoxygluconokinase	0.0258513	1.33206	0.0183259	1.25494
A0A140N8W5	1-(5-phosphoribosyl)-5-[(5-phosphoribosylamino)methylideneamino] imidazole-4-carboxamid	0.000621799	2.10902	0.0129024	1.48194
A0A140N7R1	Putative acid--amine ligase YgiC	0.196162	1.0573	0.0875698	1.06827
A0A140NCS9	Ribose-phosphate pyrophosphokinase	0.0103578	-1.18076	0.388867	1.03896
A0A140NDF8	UPF0502 protein YceH	0.00535147	1.58284	0.0022432	1.55783
A0A140N9C3	Glucokinase	0.263519	1.35062	0.00293728	1.82297
P06993	HTH-type transcriptional regulator MalT	0.168173	1.26297	0.279405	-1.11394
A0A140NF66	Catalase-peroxidase	0.0746232	1.36063	0.0271844	1.29658
A0A140NBG5	ATP-dependent Clp protease ATP-binding subunit ClpA	0.0426431	1.35156	0.126766	1.15333
A0A140N319	RNase adapter protein RapZ	0.714966	-1.04518	0.000766611	1.63208
A0A140NI72	Ketol-acid reductoisomerase	0.00200593	1.65839	0.446296	1.06861
A0A140NFJ8	Uncharacterized ABC transporter ATP-binding protein YadG	0.376135	1.08988	0.0173514	1.22673
A0A140N498	Malate synthase G	0.000903181	2.55661	0.0212235	1.49223
A0A140NHM2	dTDP-4-amino-4,6-dideoxygalactose transaminase	0.089019	-1.17177	0.051982	1.15299
P39160	D-mannonate oxidoreductase	0.00182606	2.0416	0.189282	1.16483

A0A140NBC1	HTH-type transcriptional repressor PurR	0.000522323	1.64509	0.0791439	1.21736
A0A140N9P6	Succinate semialdehyde dehydrogenase [NAD(P)+] Sad	0.0317813	-1.28515	0.00692111	1.31038
A0A140N7M4	Uncharacterized protein YegU	0.00133138	1.73999	0.0173221	1.32828
A0A140NCW6	Orotidine 5-phosphate decarboxylase	0.000800964	-1.39052	0.0144534	-1.20601
A0A140N8Y8	NAD(P) transhydrogenase subunit beta	0.00126266	1.49181	0.0351982	1.19488
A0A140N7G6	Protein YdcF	0.0305648	1.4403	0.00337552	1.52543
A0A140NDK4	ATP-binding/permease protein CydD	0.644485	-1.05339	0.0173115	1.26041
A0A140NGF8	Tryptophanase	0.40298	2.60187	0.00262183	3.34186
A0A140NFW3	Homoserine kinase	0.024901	2.48433	0.00108611	3.18367
A0A140NE28	Alpha-galactosidase	0.133812	-1.52553	0.103951	1.26127
A0A140N5R8	Fatty acid oxidation complex subunit alpha;Enoyl-CoA hydratase/3-hydroxybutyrylCoA epi	0.0010287	4.90706	0.00937016	2.6881
A0A140NAF9	Lipoyl synthase	0.0137064	-1.83433	0.0103469	1.5145
A0A140N826	Phosphohistidine phosphatase SixA	0.000418628	-1.65733	0.127008	-1.1531
A0A140N6I0	HTH-type transcriptional regulator GalR	0.310286	-1.19834	0.115967	1.19325

A0A140NEW9	Anaerobic ribonucleoside-triphosphate reductase	0.0155315	-1.55512	0.00422478	-1.5308
A0A140N4R0	Glutaredoxin-3	0.11476	1.2818	0.0308546	1.2772
A0A140N6M2	Uncharacterized protein YniA	0.017571	3.79322	0.00201678	3.1367
A0A140NA05	Endonuclease 4	0.0630081	-1.25972	0.149003	1.12934
A0A140N4V4	Cell division ATP-binding protein FtsE	0.174598	1.1436	0.0053283	1.30844
A0A140N8C1	Glycine cleavage system transcriptional activator	0.361719	1.07152	0.00134084	1.34447

A0A140N856	Histidine-binding periplasmic protein	0.000564211	1.89285	0.12005	1.19405
A0A140NBJ6	Sensor protein RstB	0.0338357	-1.62164	0.0243022	1.38335
A0A140NBE7	Formate acetyltransferase 1	0.40691	-1.14567	0.0011627	-1.79182
A0A140NFB8	UPF0253 protein YaeP	0.432365	1.15467	0.00243444	1.68749
A0A140NCP4	Transcription-repair-coupling factor	0.106756	-1.11457	0.0509585	1.11924
A0A140N8H0	Sulfate adenylyltransferase subunit 1	0.000984898	-2.8492	0.0173224	-1.72466
A0A140NC65	Peptide chain release factor 3	0.0384626	-1.1677	0.000775118	-1.41201
A0A140NAJ6	Protein AsmA	0.105415	1.15876	0.00813276	1.25498
A0A140NC89		0.00061571	2.78524	0.0742821	1.40456
A0A140N9T5	NAD-dependent malic enzyme	0.157895	-1.11001	0.983242	-1.00115
A0A140N6T9	Pyridoxine kinase	0.153145	1.18558	0.0133873	1.28783
A0A140NCV1	Uridylate kinase	0.00413628	-1.24004	0.0615491	1.10862
A0A140NBZ7		0.735453	1.08635	0.0697817	1.29889
A0A140SS26	DNA mismatch repair protein MutL	0.251561	1.12509	0.016149	1.24574
A0A140N982	Bifunctional protein PutA;Proline dehydrogenase;Delta-1-pyrroline-5-carboxylate dehydro	0.829862	-1.06361	0.00975143	1.64452
A0A140NER9	DNA polymerase I	0.951388	1.00448	0.0338656	1.15544
A0A140NFK0	Methionine synthase	0.115107	1.1375	0.618959	-1.02982
A0A140N417	G/U mismatch-specific DNA glycosylase	0.177346	1.37394	0.00180255	1.9836
A0A140NGX6	Chorismate pyruvate-lyase	0.120838	1.332	0.0043664	1.57408
A0A140NDN8	Catabolite repressor/activator	0.585762	1.087	0.0109254	1.38892
A0A140NGG3	Aerobic respiration control protein ArcA	0.0261162	1.36027	0.00200111	1.54111
A0A140N8L0	NAD-dependent dihydropyrimidine dehydrogenase subunit PreT	0.105965	1.63532	0.0243632	1.4846

A0A140NDR8	Ubiquinone/menaquinone biosynthesis Cmethyltransferase UbiE	0.474231	-1.06118	0.130543	1.10766
A0A140ND30	UDP-N-acetylmuramoylalanine--D-glutamate ligase	0.345605	1.06492	0.0186444	1.16474
C5W865	Sulfite reductase [NADPH] hemoprotein betacomponent	0.00164149	-1.43055	0.0162313	-1.28475
A0A140N9J5	Peptide methionine sulfoxide reductase MsrB	0.00437662	1.51659	0.00329823	1.56668
A0A140NEM4	Superoxide dismutase [Mn]	0.341153	1.3845	0.00195637	-2.3987
A0A140N6Z1	Mannose-6-phosphate isomerase	0.770723	1.04767	0.005818	1.48751
A0A140N5T6	Erythronate-4-phosphate dehydrogenase	0.95555	1.00367	0.0341087	1.14164
P0ACX3	Putative monooxygenase YdhR	0.716786	1.10146	0.00179478	2.16381
A0A140NA80	Succinate dehydrogenase flavoprotein subunit	0.00294042	1.38149	0.0675412	1.21392
A0A140N923	Iron-sulfur cluster assembly scaffold protein IscU	0.00559744	-1.24644	0.394779	-1.06545
A0A140NB59	ATP-dependent 6-phosphofructokinase isozyme 2	0.697992	1.0964	0.00384582	1.7807
A0A140NDI9	3-methyl-2-oxobutanoate hydroxymethyltransferase	0.409017	1.1517	0.00746143	1.47818
A0A140N640	2,3-bisphosphoglycerate-independent phosphoglycerate mutase	0.201532	-1.15461	0.00872747	1.31451
A0A140SSB8	Putative uroporphyrinogen-III Cmethyltransferase	0.00937642	1.39677	0.0426092	1.20559
A0A140N5V1	Enhancing lycopene biosynthesis protein 2	0.074536	1.41509	0.146543	1.18546
A0A140SSB3	Bifunctional protein GlmU;UDP-Nacetylglucosamine pyrophosphorylase;Glucosamine-1-phosp	0.871367	-1.02181	0.00295111	-1.50994
A0A140NF56	Chromate reductase	0.540384	1.06494	0.183655	1.10993
A0A140N231	HTH-type transcriptional regulator YiaJ	0.715606	1.04617	0.00466712	1.41906

A0A140NB99	Cysteine desulfurase	0.00364565	2.80261	0.0457879	1.45229
A0A140N7Z4	Uncharacterized protein YeiR	0.122618	-1.32095	0.517973	1.07029
A0A140N5D3	Disulfide-bond oxidoreductase YghU	0.232933	1.44539	0.00767865	1.75544
A0A140N7S8	Probable protease SohB	0.22795	1.10907	0.00474589	1.28993
P0AFB1	Lipoprotein NlpI	0.328301	-1.15778	0.00434194	1.50346
A0A140N5E7	Exopolyphosphatase	0.931146	-1.00716	0.00717837	1.26166
A0A140NFA1	Transcriptional regulatory protein CpxR	0.0310764	1.36059	0.0040603	1.46554
A0A140NCG6	Cytochrome bd-I ubiquinol oxidase subunit 1	0.2136	-1.80154	0.0360137	1.56507
A0A140NBB3	Pyruvate kinase II	0.0329876	1.40921	0.0339111	1.27659
A0A140N916		0.0370123	-2.10877	0.600035	-1.08171
A0A140N9H1	Beta-barrel assembly-enhancing protease	0.021399	1.36748	0.0213656	1.27416
A0A140N6Z9	30S ribosomal protein S5	0.970103	-1.00253	0.00098687	-1.44991
A0A140NCD7	Glucose-6-phosphate isomerase	0.411352	-1.23123	0.0172523	1.50902
A0A140N6W3	Phosphoenolpyruvate-protein phosphotransferase PtsP	0.983552	-1.00251	0.00509449	1.40768
A0A140N7C6	NH(3)-dependent NAD(+) synthetase	0.0689089	1.23629	0.00436971	1.38441
A0A140N6A8	Outer membrane protein TolC	0.7189	-1.04891	0.0122858	1.34548
A0A140NB33	Protein translocase subunit SecF	0.144128	-1.07655	0.044339	-1.09913
A0A140N8G3	Aminomethyltransferase	0.341129	1.26108	0.676259	-1.05457
A0A140N8M8	Exodeoxyribonuclease I	0.113518	-1.2486	0.153958	1.14712
A0A140NDZ2	Thymidine phosphorylase	0.430819	-1.24543	0.00292458	2.04701
A0A140N7Z8	Dihydropteroate synthase	0.0196564	-1.13536	0.00687178	1.15624
A0A140N7A7	Probable phospholipid ABC transporter-binding protein MlaD	0.24573	1.10337	0.358127	-1.06127
A0A140NBL6	Molybdopterin molybdenumtransferase	0.532586	-1.05764	0.0138236	1.23881

A0A140N6I5	Selenide, water dikinase	0.763982	1.07679	0.0132709	1.55506
A0A140NAC4	PhoH-like protein	0.210675	1.1288	0.720826	1.02511
A0A140NDK0	Uncharacterized protein YahJ	0.305491	1.10067	0.00648629	1.29698
A0A140NCL7	Ribosomal large subunit pseudouridine synthase B	0.050933	1.15444	0.00414842	-1.3302
A0A140NBF8	ABC transporter arginine-binding protein 1	0.00102721	2.61365	0.942496	-1.01077
A0A140N8J1	DNA topoisomerase 3	0.52614	1.08768	0.0244964	1.2845
A0A140NDE9	Uncharacterized protein YcbX	0.0775385	1.21803	0.0332248	1.21391
A0A140N210	Selenocysteine-specific elongation factor	0.0574857	1.2241	0.00816692	1.29987
A0A140N785	Transcriptional regulator NanR	0.208459	1.12259	0.0187883	1.21954
A0A140NEN2	Uncharacterized protein YjeI	0.0317564	1.51214	0.0131322	1.44206
A0A140N7V1	Hydrogenase-2 operon protein HybA	0.0765619	-1.71899	0.688161	-1.05866
A0A140NGZ8		0.160087	1.34423	0.00902978	1.55141
A0A140N9N7	UDP-4-amino-4-deoxy-L-arabinose-oxoglutarate aminotransferase	0.0697773	1.33582	0.0154449	1.36397
A0A140NDE0	Proline--tRNA ligase	0.0195514	-1.156	0.60778	-1.02327
A0A140N7H9	Pyridoxine 5-phosphate synthase	0.111927	1.27975	0.0171813	1.34871
A0A140N5J9	Putative osmoprotectant uptake system substrate-binding protein OsmF	0.0879765	2.48117	0.0142646	1.91355
P0AEE5	D-galactose-binding periplasmic protein	0.852323	-1.077	0.0539726	1.46612
A0A140N6P1	Phosphoenolpyruvate synthase regulatory protein	0.449673	1.11406	0.62869	1.04662
A0A140N2F9	Uncharacterized oxidoreductase YhhX	0.863924	1.03033	0.0103916	1.46593
A0A140N929	Transcriptional regulator KdgR	0.297402	1.16119	0.00552705	1.4659
A0A140NBX3		0.815146	1.03667	0.00655081	1.48167

A0A140N564	Signal recognition particle receptor FtsY	0.583248	1.04352	0.0888966	1.12185
A0A140NAV5	Protein YceI	0.00136394	1.69975	0.148635	1.21506
A0A140NEV1	Cytosol aminopeptidase	0.00649339	1.56707	0.0300404	1.29704
A0A140NBR9	Glutamine transport ATP-binding protein GlnQ	0.00126172	2.38563	0.141228	1.27541
A0A140NA86	Penicillin-binding protein activator LpoB	0.254851	1.17684	0.00378305	1.52045
A0A140N7T2	tRNA pseudouridine synthase A	0.155244	-1.10368	0.0794505	1.11062
A0A140N4Y7	ATP-dependent RNA helicase DeaD	0.00210805	-1.44908	0.025462	-1.25913
A0A140NBS3	Dihydrolipoyl dehydrogenase	0.572903	1.03802	0.00211838	1.28884
A0A140NDD5	ATP-binding/permease protein CydC	0.360027	-1.11837	0.149513	1.14049
A0A140NCP3	Uncharacterized protein YbbN	0.0348968	1.22062	0.0257643	1.19518
A0A140N877	Hydrogenase-2 small chain	0.0249731	-1.78268	0.39604	-1.11916
A0A140NA29	Alanine racemase, catabolic	0.00325824	1.90612	0.0868034	1.50414
A0A140N9M5	Oxidoreductase UcpA	0.013823	1.71371	0.399362	-1.10585
A0A140NGL5	Type I restriction enzyme EcoKI R protein	0.0422911	1.32528	0.650549	1.04041
A0A140NAC5	Periplasmic murein peptide-binding protein	0.00455451	1.76736	0.10766	1.22072
A0A140NB95	Vitamin B12 transport periplasmic protein BtuE	0.411847	1.19656	0.00916249	1.58471
A0A140NB73	Alpha,alpha-trehalose-phosphate synthase [UDP-forming]	0.176952	2.24916	0.00951365	2.20426
A0A140N850	Modulator of drug activity B	0.888378	-1.02546	0.433968	1.09177
A0A140N6F6	Oligopeptidase A	0.0388345	1.18362	0.670927	1.02496
A0A140NE14		0.110088	-1.56461	0.00661516	1.8014
A0A140N8D2	Metalloprotease LoiP	0.687461	1.05694	0.212936	1.13049
A0A140N9N3	Uncharacterized protein YbeL	0.0034484	2.99948	0.0517367	1.50986
A0A140NEY3		0.154502	1.12922	0.2586	-1.0774

A0A140N6P5	Fructose-1-phosphate phosphatase YqaB	0.169161	1.26268	0.0150491	1.40119
A0A140N8B6	Uncharacterized protein YggN	0.00122178	1.70665	0.76698	-1.02965
A0A140NCK3	Inhibitor of g-type lysozyme	0.692172	1.16665	0.012469	1.82388
A0A140N849	USG-1 protein	0.957105	-1.00632	0.00824148	1.35822
A0A140N8T5	HTH-type transcriptional regulator HexR	0.215008	1.27124	0.928648	-1.01016
A0A140ND98		0.400626	-1.05959	0.0306035	-1.15341
A0A140NCX5	DNA topoisomerase 1	0.00271224	1.16379	0.0625621	-1.0761
A0A140NCU5	6,7-dimethyl-8-ribityllumazine synthase	0.949536	1.00605	0.0950415	1.14307
A0A140NAR1	Beta-hexosaminidase	0.582574	1.05546	0.714373	-1.02709
A0A140NCI3	Phage shock protein A	0.0222837	1.72695	0.0041292	1.90569
A0A140NBR3	Carbonic anhydrase 2	0.262184	-1.12154	0.259977	-1.09106
A0A140NC42	Inner membrane protein YbaL	0.00346289	1.86549	0.198001	-1.1763
A0A140NEW2	Uncharacterized protein YajD	0.0452248	1.27025	0.245767	1.10115
A0A140NEZ7	Nucleoside-specific channel-forming protein tsx	0.508662	-1.08502	0.00767717	1.38289
A0A140N737	UPF0304 protein YfbU	0.230898	1.20172	0.0291158	1.30647
A0A140ND10	Peptidyl-prolyl cis-trans isomerase B	0.812843	-1.02101	0.0211454	1.21533
A0A140N2V6		0.478339	-1.12951	0.0189	1.39059
A0A140N9R8	Thiol:disulfide interchange protein DsbG	0.274785	1.4157	0.00244649	2.53602
A0A140NGD1	HTH-type transcriptional regulator TreR	0.00461576	1.57929	0.0125764	1.45831
A0A140N8G9	Sulfite reductase [NADPH] flavoprotein alphacomponent	0.00631957	-1.30588	0.0528032	-1.20917
A0A140NGN0	Aspartate ammonia-lyase	0.661506	-1.09563	0.323423	1.13288
A0A140ND08	Adenylate cyclase	0.00242472	-2.40468	0.418589	-1.13133
A0A140N8S8	Lysine--tRNA ligase	0.0343895	-1.24523	0.217447	1.09645

A0A140NDQ6	3-ketoacyl-CoA thiolase	0.00368286	14.7503	0.0228817	8.89807
A0A140N6N3	Phosphoribosylaminoimidazolesuccinocarboxamide synthase	0.161839	2.23704	0.0062499	2.59771
A0A140NB70	Paraquat-inducible protein B	0.0134611	-1.20266	0.249373	1.06505
A0A140NB87	Nicotinate phosphoribosyltransferase	0.00769646	-1.66507	0.00829802	-1.64905
A0A140N2B1	Glutathione reductase	0.567539	-1.05505	0.023805	1.22132
A0A140N7U4		0.22169	-1.07843	0.510689	1.03354
A0A140NHF7	LexA repressor	0.241287	1.20233	0.478838	1.07561
A0A140NBE6	Low specificity L-threonine aldolase	0.0530139	1.32301	0.0760282	1.20684
A0A140N5B4	30S ribosomal protein S13	0.471222	-1.05609	0.00146583	-1.43866
A0A140SSA5	ATP-dependent RNA helicase RhlB	0.532854	1.06046	0.023855	1.22217
A0A140NA92	3-oxoacyl-[acyl-carrier-protein] synthase 2	0.00475019	-1.30887	0.477034	-1.04634
A0A140N3N8	N-acetylneuraminate lyase	0.59134	-1.11516	0.0861321	-1.26885
A0A140N8E6	Galactitol-specific phosphotransferase enzyme IIB component	0.896957	1.06655	0.0540213	1.56726
A0A140NDN0	D-alanine--D-alanine ligase B	0.848169	-1.01809	0.0536545	1.17493
A0A140NA34	Anaerobic glycerol-3-phosphate dehydrogenase subunit C	0.830848	-1.13918	0.735837	-1.07082
A0A140NBG7	Fumarate hydratase class II	0.0871716	1.56633	0.0123704	1.64752
A0A140NBV2	UvrABC system protein B	0.829531	-1.03278	0.00341632	1.61759
A0A140N643	Hydrogenase 2 maturation protease	0.0811317	-1.30644	0.697007	1.03882
A0A140N8C2	Glycerol-3-phosphate transporter	0.490417	1.25262	0.0316893	-1.54702
A0A140NAF5	AMP nucleosidase	0.22997	1.30091	0.131416	1.23387
A0A140N2G7	Glucose-1-phosphate adenylyltransferase	0.0100596	2.6394	0.018459	1.84056
A0A140NHV7	Alpha-D-glucose-1-phosphate phosphatase YihX	0.00337238	2.48592	0.0463615	1.55857

A0A140NEG8	Pyrimidine 5-nucleotidase YjjG	0.742668	-1.03844	0.337157	1.08643
A0A140N8L8	Imidazole glycerol phosphate synthase subunit HisH	0.00369884	2.35137	0.0384954	1.57438
A0A140SSB6	Flavin mononucleotide phosphatase YigB	0.190211	1.18712	0.0866713	1.1892
A0A140SS47	UPF0047 protein YjbQ	0.102245	1.32138	0.00659153	1.5491
A0A140NCW8	Cation efflux system protein CusA	0.00896426	-1.79241	0.0598683	-1.32514
A0A140NAY6	Chaperone protein YcdY	0.832904	-1.01464	0.465951	1.04264
A0A140NCX0	DNA-damage-inducible protein I	0.0369131	1.29934	0.0531061	1.20877
A0A140N3U1	tRNA-modifying protein YgfZ	0.594472	1.06146	0.297786	1.09296
A0A140NCN3	Spermidine/putrescine-binding periplasmic protein	0.00268405	1.51868	0.108743	1.20476
A0A140NGE1	Fructose-1,6-bisphosphatase class 1	0.110605	1.11672	0.0206744	-1.16744
A0A140ND33	Ribosomal RNA large subunit methyltransferase I	0.0282479	-1.23173	0.00495976	-1.36834
A0A140N340	50S ribosomal protein L27	0.291321	-1.05884	0.00192815	-1.2506
A0A140NHG8	Maltose/maltodextrin import ATP-binding protein MalK	0.0396602	-2.09084	0.0644679	-1.44367
A0A140N627	S-adenosylmethionine synthase	0.114492	1.24032	0.0251438	1.2956
A0A140NC58	L-Ala-D/L-Glu epimerase	0.045785	1.73031	0.009201	1.79322
A0A140NAY3	Histidine biosynthesis bifunctional protein HisB;Histidinol-phosphatase;Imidazoleglycer	0.00388437	2.27203	0.0454718	1.52551
A0A140N836		0.00619488	1.53092	0.0417154	1.27697
A0A140NAN1	D-lactate dehydrogenase	0.839467	1.03658	0.0566871	1.29153
A0A140N534	Phosphoglycolate phosphatase	0.712168	-1.04101	0.0424906	1.21638
A0A140NEW0	Uncharacterized protein YbaE	0.283649	1.65215	0.0224974	1.82017
A0A140SSC5	ATP synthase epsilon chain	0.606176	1.04835	0.0930276	1.14224

A0A140NBX8	Putative ABC transporter arginine-binding protein 2	0.0144342	1.80031	0.0580094	1.35501
A0A140N494	Serine acetyltransferase	0.138518	1.12393	0.00946214	1.23678
A0A140N7C3	Argininosuccinate synthase	0.00609836	1.64175	0.824753	-1.0242
A0A140N964	tRNA pseudouridine synthase D	0.00473915	-1.19323	0.953948	-1.00251
A0A140NEK7	Inosine-guanosine kinase	0.0031263	-1.6485	0.201479	-1.20466
A0A140NET2	Trigger factor	0.116257	1.14626	0.00280535	-1.40555
A0A140NCM4	Fatty acid metabolism regulator protein	0.171605	-1.15154	0.0959344	-1.15094
A0A140NEK5	Uncharacterized GTP-binding protein YjiA	0.0125155	1.53145	0.0230275	1.37508
A0A140NDF0	D-alanine--D-alanine ligase A	0.0400974	1.23953	0.0222479	-1.23698
A0A140N9Q3	GMP synthase [glutamine-hydrolyzing]	0.00325588	-1.38921	0.941896	1.00669
A0A140NBM7	Leucine--tRNA ligase	0.0444913	-1.18705	0.239774	-1.07981
A0A140N7J3	Sulfate/thiosulfate import ATP-binding protein CysA	0.00196958	-2.42736	0.527018	1.13754
A0A140N530	Uncharacterized protein YfcL	0.186601	-1.37174	0.00714242	-1.78939
A0A140NBY5	4-hydroxythreonine-4-phosphate dehydrogenase	0.200938	-1.13302	0.02957	1.21494
A0A140NFN5	UDP-N-acetylmuramoyl-L-alanyl-D-glutamate-2,6-diaminopimelate ligase	0.42385	-1.09803	0.11917	1.15766
A0A140NEB0	2-octaprenyl-3-methyl-6-methoxy-1,4benzoquinol hydroxylase	0.476923	-1.18486	0.00643809	1.82516
A0A140NCT4	Multidrug efflux pump subunit AcrB	0.00599847	1.30035	0.253139	-1.08088
A0A140NCC1	Glycerol-3-phosphate acyltransferase	0.739379	1.01838	0.354891	1.04556
A0A140N645	Ecotin	0.010723	1.53122	0.216537	-1.14584
A0A140N4S7	Stringent starvation protein A	0.167867	-1.1275	0.0886702	1.1347

A0A140N2L2	ATP-dependent DNA helicase RecG	0.0790925	-1.17106	0.916475	1.00695
A0A140NAA1	Ribonuclease E	0.0126173	1.27505	0.371078	-1.06347
A0A140N5L3	ATP-dependent zinc metalloprotease FtsH	0.345708	1.07706	0.205509	1.08733
A0A140NAY4	Aminodeoxychorismate synthase component 1	0.00607751	-1.31332	0.156084	-1.10915
A0A140NAW9	Adenylate kinase	0.337388	1.11335	0.0849048	1.17372
P46859	Thermoresistant gluconokinase	0.158799	1.17941	0.00293114	-1.60045
A0A140N8Y0	Fumarate hydratase class I, aerobic	0.00470272	1.44654	0.0683637	1.20556
A0A140NE25	Glutamine--fructose-6-phosphate aminotransferase [isomerizing]	0.00270712	-1.17515	0.197586	-1.05397
A0A140N5D2	Penicillin-binding protein 1A;Penicillininsensitive transglycosylase;Penicillin-sensit	0.142878	-1.09463	0.00300127	-1.26537
A0A140NBQ1	ATP-dependent RNA helicase RhlE	0.00642505	-1.53652	0.0602766	-1.33482
A0A140NEJ3	Oxygen-insensitive NAD(P)H nitroreductase	0.939505	1.01033	0.19138	1.1438
A0A140NEF8	Ribonuclease I	0.510235	-1.08231	0.218905	1.12078
A0A140NBW3	Sensor kinase CusS	0.0527586	-1.40081	0.422306	-1.09345
A0A140NG49	Blue copper oxidase CueO	0.167545	1.19775	0.166534	-1.14558
A0A140NF01	Transcription termination factor Rho	0.0337084	-1.2788	0.372751	-1.07555
A0A140N8D7	Hydroxymethylpyrimidine/phosphomethylpyrimidine kinase	0.685702	1.04673	0.40597	-1.07407
A0A140N556	Peptide deformylase	0.0652602	-1.33409	0.398987	-1.09176
A0A140NFM7	50S ribosomal protein L1	0.0864086	-1.24561	0.00441819	-1.51833
A0A140N5K0	2-octaprenyl-6-methoxyphenol hydroxylase	0.524845	-1.10783	0.016768	1.41442
A0A140NFC6	UPF0234 protein YajQ	0.649976	1.087	0.0245457	1.41114
A0A140N8Z2	N-acetylmuramoyl-L-alanine amidase AmiC	0.462105	1.06517	0.0126851	1.25055

A0A140NEJ4	Met repressor	0.324142	1.13617	0.0139659	1.35742
A0A140N2U8	Glycine--tRNA ligase beta subunit	0.723004	-1.03995	0.544313	-1.05213
A0A140N8M2	Nicotinamide-nucleotide amidohydrolase PncC	0.534686	1.09865	0.0380725	1.30217
A0A140N429	L-serine dehydratase 2	0.119512	-2.0171	0.00819777	-2.51381
A0A140NDR7	UDP-N-acetylmuramate--L-alanyl-gamma-Dglutamyl-meso-2,6-diaminoheptandioate ligase	0.661677	1.03971	0.555917	-1.04259
A0A140N644	Glutamate--cysteine ligase	0.0294118	1.27295	0.880669	1.01155
A0A140NB54	RNA chaperone ProQ	0.164583	-1.10392	0.031616	-1.15822
A0A140NGB8	Bis(5-nucleosyl)-tetraphosphatase [symmetrical]	0.323544	-1.27616	0.0327537	1.47419
A0A140NAQ1	Lipoprotein-releasing system ATP-binding protein LolD	0.0074631	-1.36944	0.992412	-1.00074
A0A140N6P6	D-3-phosphoglycerate dehydrogenase	0.0631987	1.64673	0.0104352	1.82047
A0A140N8K8	Uncharacterized protein YciO	0.111121	1.25951	0.0138188	1.39402
A0A140NFF9	Protein YihD	0.407625	-1.1663	0.0467623	1.33312
A0A140NCZ8	S-adenosylmethionine decarboxylase proenzyme;S-adenosylmethionine decarboxylase beta ch	0.00291056	2.24286	0.332436	1.22159
A0A140NGV4	UvrABC system protein A	0.660871	-1.06914	0.0701137	1.24686
A0A140N9H4	1,4-dihydroxy-2-naphthoyl-CoA hydrolase	0.0193287	1.98305	0.0140211	1.90382
A0A140N5J6	2-hydroxy-3-oxopropionate reductase	0.877386	1.0963	0.0911643	-1.54368
A0A140NC38	Esterase YbfF	0.648051	-1.04473	0.0908827	1.1524
A0A140N7N1	Glutathione S-transferase GstA	0.09966	1.59102	0.0242106	1.59784
A0A140N7Q1	Uridine kinase	0.00606016	-1.44954	0.998251	1.00019
A0A140NG83	UDP-N-acetyl-D-mannosamine dehydrogenase	0.394351	-1.10734	0.00533043	-1.45931

A0A140NF95	Phosphoethanolamine transferase EptA	0.482471	1.07705	0.00508211	-1.41097
A0A140NEQ7	Apolipoprotein N-acyltransferase	0.915118	1.01171	0.599444	1.04489
A0A140SSA8	Probable protein kinase UbiB	0.95996	1.00504	0.161887	1.12469
A0A140N7X0	Arabinose 5-phosphate isomerase KdsD	0.767456	1.05118	0.0128772	1.48613
A0A140NDK1	Quinoprotein glucose dehydrogenase	0.00775112	-1.49784	0.048494	-1.28464
A0A140NFF1	Oxygen-independent coproporphyrinogen-III oxidase	0.402327	-1.11364	0.155536	1.15396
A0A140NE32	Mrr restriction system protein	0.0368774	1.42582	0.038365	1.32576
A0A140N8L5	Probable Fe(2+)-trafficking protein	0.600498	1.09024	0.0893269	1.24118
A0A140NFT3	Riboflavin biosynthesis protein RibF;Riboflavin kinase;FMN adenylyltransferase	0.166786	1.13559	0.035731	1.1954
A0A140NF68	Cys-tRNA(Pro)/Cys-tRNA(Cys) deacylase YbaK	0.944298	-1.01243	0.0355093	1.36122
A0A140N2J1	33 kDa chaperonin	0.00664327	1.30057	0.0582872	-1.17317
A0A140N4M5	Inositol-1-monophosphatase	0.0299159	-1.48575	0.020387	-1.70072
A0A140N7T6	Tryptophan synthase beta chain	0.218821	1.24749	0.0988568	1.24437
A0A140N5J7	3-deoxy-D-manno-octulosonate 8-phosphate phosphatase KdsC	0.231027	-1.28398	0.0558871	1.3469
A0A140N465	Coenzyme A biosynthesis bifunctional protein CoaBC;Phosphopantothenoylcysteine decarbox	0.92062	-1.00973	0.134459	1.13303
A0A140N6P8	GDP-mannose pyrophosphatase NudK	0.685835	-1.08869	0.0283365	1.45544
A0A140NFW5	Protein CreA	0.0162772	1.54636	0.0551082	-1.31163
A0A140NCM5	Probable pyruvate-flavodoxin oxidoreductase	0.668203	1.06399	0.107941	1.20324
A0A140N993	NAD(P)H dehydrogenase (quinone)	0.316018	1.78695	0.0346027	1.90138
A0A140N843	2-octaprenylphenol hydroxylase	0.479994	-1.12932	0.755149	1.03663

A0A140NDZ5	Ribosomal RNA small subunit methyltransferase C	0.657342	-1.04952	0.651198	1.03852
A0A140SSB4	Uncharacterized protein YigI	0.193908	1.14071	0.913843	-1.01399
A0A140N9A4	Glucose-specific phosphotransferase enzyme IIA component	0.493233	1.1707	0.0328674	1.46786
A0A140N7R5	Probable 4-deoxy-4-formamido-L-arabinosephosphoundecaprenol deformylase ArnD	0.854206	-1.03675	0.212799	1.18451
A0A140ND61	Chaperone protein HtpG	0.0204876	1.30267	0.0550599	-1.20057
A0A140NFF3	Phosphoheptose isomerase	0.424586	1.12919	0.193578	1.15963
A0A140N6T1	N-acetylmuramic acid 6-phosphate etherase	0.757441	1.06305	0.13694	-1.23432
A0A140N7C4	Phosphoribosylglycinamide formyltransferase	0.0108407	1.74788	0.0541233	1.40217
A0A140NA88	Cytochrome bo(3) ubiquinol oxidase subunit 1	0.00945501	2.38783	0.672829	-1.1892
A0A140NBY1	Transcriptional regulator SlyA	0.57145	1.08318	0.0157084	1.39383
A0A140N6K1	Amino-acid acetyltransferase	0.273899	1.37488	0.0122158	1.87507
A0A140N3M3	Metalloprotease TldD	0.283692	-1.10744	0.809654	1.0179
A0A140NF24	DNA-binding protein HU-alpha	0.0599057	1.37076	0.0397464	-1.32767
A0A140N9J8	Transketolase 2	0.223141	2.36498	0.0264871	2.22572
A0A140N5H1	Inner membrane protein YhcB	0.00925054	1.39023	0.373037	1.08275
A0A140NAF3	Iron uptake system component EfeO	0.617364	1.08153	0.00655986	-1.60171
A0A140NHE6	Alanine racemase, biosynthetic	0.00650779	-1.36592	0.317139	1.0868
A0A140NBE2	N-methyl-L-tryptophan oxidase	0.0166215	1.28237	0.324405	1.07805
A0A140N6C1	DNA gyrase subunit B	0.833347	-1.01283	0.0900484	-1.10357
A0A140N7M5	Acetate kinase	0.0305547	-1.47224	0.146845	1.20256
A0A140ND69	Gamma-glutamyl phosphate reductase	0.317418	1.13074	0.0976825	1.1843

A0A140NE43	tRNA-2-methylthio-N(6)-dimethylallyladenosine synthase	0.0196799	-1.2478	0.0233938	-1.24192
A0A140NEQ0	ATP-dependent Clp protease ATP-binding subunit ClpX	0.401906	-1.10578	0.0567931	-1.22244
A0A140N5A3	50S ribosomal protein L5	0.276803	-1.12049	0.00544789	-1.48326
A0A140NFA7	Methionine import ATP-binding protein MetN	0.0122618	1.90675	0.0472739	1.53271
A0A140NI52	UDP-N-acetyl-D-mannosaminuronic acid transferase	0.0307152	-1.24777	0.0230969	-1.24748
A0A140ND07	1,6-anhydro-N-acetylmuramyl-L-alanine amidase AmpD	0.25665	1.29049	0.885302	-1.02
A0A140N7I1	Threonylcarbamoyl-AMP synthase	0.070955	-1.31473	0.606068	-1.05486
A0A140NAX1	L-serine dehydratase 1	0.138135	-1.25832	0.529793	-1.06974
A0A140NFJ7	Pyridoxal phosphate phosphatase YigL	0.011971	-1.51951	0.717057	-1.04037
A0A140N6C3	Uncharacterized protein YebT	0.594156	-1.10244	0.0104509	1.60039
A0A140N5T5	Periplasmic pH-dependent serine endoprotease DegQ	0.122599	1.39812	0.0360854	1.43753
A0A140NDG9	Uncharacterized protein YcbL	0.890733	-1.01551	0.0241655	1.2818
A0A140NG13		0.00416577	2.9697	0.808342	1.06644
A0A140NF30	N5-carboxyaminoimidazole ribonucleotide mutase	0.00720965	2.65765	0.162314	1.58295
A0A140N7M2	Co-chaperone protein HscB	0.0403909	-1.23789	0.0675866	-1.23957
A0A140NEA4	Ferrienterobactin receptor	0.876506	-1.48567	0.442463	-1.29507
A0A140NDS1	Molybdenum cofactor biosynthesis protein B	0.675833	1.03869	0.0104061	1.29616
A0A140N8X9	Serine hydroxymethyltransferase	0.137763	1.5101	0.0415217	1.5154
A0A140NEP7	Glucosamine-6-phosphate deaminase	0.127273	1.39498	0.725081	1.04875
A0A140NAD5	Protein YeeZ	0.241336	1.229	0.109234	1.23827

A0A140N7P0	Exodeoxyribonuclease 7 large subunit	0.0227384	-1.47305	0.0997772	1.23699
A0A140NE35	GMP reductase	0.481745	1.04503	0.0753434	1.11321
P0A8W5	UPF0301 protein YqgE	0.812035	-1.03315	0.125446	-1.18653
A0A140NDJ4	Ribosomal protein S12 methylthiotransferase RimO	0.0264825	-1.37972	0.125153	-1.28241
A0A140NAR9	Holliday junction ATP-dependent DNA helicase RuvB	0.273191	-1.15417	0.349339	1.09682
A0A140NF40	tRNA threonylcarbamoyladenosine biosynthesis protein TsaE	0.786163	-1.03685	0.109583	1.19352
A0A140N4C5	Inner membrane protein YgaP	0.145372	1.4874	0.148175	1.28761
A0A140NHK8	Uroporphyrinogen-III synthase	0.633977	1.11968	0.028741	1.52763

A0A140NF86	ATP-dependent protease ATPase subunit HslU	0.639184	1.05148	0.00493838	-1.51633
A0A140NE88	Ribosomal RNA small subunit methyltransferase A	0.123427	-1.32082	0.0527433	-1.32585
A0A140N7I9	Phosphoribosylformylglycinamidine synthase	0.0187342	2.68943	0.044303	1.89123
A0A140N751	Uracil phosphoribosyltransferase	0.0290375	-1.16791	0.0233533	-1.18127
A0A140N9R6	Exoribonuclease 2	0.0242113	-1.33537	0.029211	-1.33452
A0A140NE87	Single-stranded DNA-binding protein	0.0258031	1.31015	0.340898	1.0883
A0A140NH83	CDP-diacylglycerol pyrophosphatase	0.354567	1.07198	0.0667253	-1.13902
A0A140N6W2	Anhydro-N-acetylmuramic acid kinase	0.603963	1.13464	0.0259591	1.56676
A0A140NE13	Ferric uptake regulation protein	0.66624	1.05011	0.160485	1.14249
A0A140SS21	23S rRNA (guanosine-2-O-)-methyltransferase RlmB	0.5039	1.07463	0.777316	1.02389
A0A140N770	4-hydroxy-tetrahydrodipicolinate synthase	0.167058	1.15719	0.603614	1.0428
A0A140NEF9	Pantothenate kinase	0.749805	1.01952	0.018637	-1.16969

A0A140NEC0	Lysine-sensitive aspartokinase 3	0.947748	-1.00641	0.450568	1.06178
A0A140N3E6	Glutamate-ammonia-ligase adenylyltransferase	0.225597	1.21321	0.0230842	1.39993
A0A140N8V9	Adenylyl-sulfate kinase	0.0122452	-2.55623	0.673648	-1.08958
A0A140ND46	Dihydroorotase	0.475176	-1.06269	0.0255535	1.21455
A0A140NAU5	Phosphoribosylglycinamide formyltransferase 2	0.0123092	3.59649	0.0749923	2.09204
A0A140ND88	3-phosphoshikimate 1-carboxyvinyltransferase	0.0251461	1.17731	0.1055	1.10541
A0A140N7B4	Probable phospholipid ABC transporter-binding protein MlaB	0.610317	1.17614	0.110848	1.37906
A0A140N7T7		0.822596	-1.02306	0.0954412	1.16364
P0ADR6	Ribosomal RNA large subunit methyltransferase M	0.687004	1.03715	0.0351092	1.20796
A0A140N5L7	50S ribosomal protein L24	0.562321	1.05207	0.00632675	-1.38593
A0A140NF15	Isocitrate lyase	0.239396	3.95281	0.0142002	5.29563
A0A140N729	Protein DcrB	0.361021	1.12826	0.988557	-1.00139
A0A140NFG5	Methionine aminopeptidase	0.141901	-1.1296	0.638279	-1.03148
A0A140N5V4	Phosphodiesterase YfcE	0.41261	-1.12227	0.324332	1.11102
P35340	Alkyl hydroperoxide reductase subunit F	0.519936	-1.09556	0.0568096	1.26609
A0A140NB38	KHG/KDPG aldolase;4-hydroxy-2-oxoglutarate aldolase;2-dehydro-3-deoxy-phosphogluconate	0.99546	-1.00116	0.0187493	1.55778
A0A140NBD7	Pyridoxine/pyridoxamine 5-phosphate oxidase	0.0584526	-1.35412	0.0655468	1.27345
A0A140NEF1		0.35021	1.09683	0.0945981	1.15832
A0A140N7C7	Elongation factor G	0.228722	-1.07524	0.0125151	-1.20879
A0A140NDW1	Pyruvate formate-lyase 1-activating enzyme	0.291609	-1.19498	0.0171489	1.48118
A0A140NAH1	Protein YchN	0.931225	-1.02226	0.152955	1.27923
A0A140N500	Transcriptional repressor MprA	0.482929	-1.10023	0.630694	-1.04988

A0A140NCE0	Cation efflux system protein CusC	0.0482656	-1.5072	0.341426	-1.14089
A0A140NFX8	DNA polymerase III subunit alpha	0.640168	-1.0501	0.226161	1.11183
A0A140NEI9	Protein TolB	0.280166	1.10787	0.0408303	1.20513
A0A140N9N0	Arginine transport ATP-binding protein ArtP	0.029503	1.48182	0.100795	-1.26014
A0A140N8Z4	Protein MtfA	0.0466968	1.60466	0.331455	1.16137
A0A140N9W7	Amidophosphoribosyltransferase	0.0101399	2.55723	0.695373	1.15704
A0A140NCZ7	Taurine-binding periplasmic protein	0.479585	-1.0992	0.0359369	1.29958
A0A140ND70	Multidrug efflux pump subunit AcrA	0.010339	1.41978	0.317444	1.10852
A0A140N6G5	Uncharacterized protein YhiI	0.00807709	1.67756	0.607805	1.07324
A0A140N7E6	Threonine--tRNA ligase	0.208229	1.22216	0.075463	1.26415
A0A140N9Y4	Cysteine synthase B	0.988406	-1.00089	0.177728	-1.10724
A0A140NAE3	Glyoxylate/hydroxypyruvate reductase A	0.0371134	1.29174	0.103506	-1.17997
A0A140NHY7	Molybdenum cofactor guanylyltransferase	0.746108	1.06484	0.0821424	1.308
A0A140N727	Epimerase family protein YfcH	0.0451885	1.53634	0.0392172	1.47894
A0A140N582	Rod shape-determining protein MreB	0.396552	-1.05144	0.254263	-1.06285
A0A140N307		0.827806	1.05668	0.0181114	1.73759
A0A140NE18	Hypoxanthine phosphoribosyltransferase	0.415959	-1.21436	0.00903715	-2.09515
A0A140N955	UPF0053 inner membrane protein YfjD	0.944366	-1.014	0.0369029	1.4359
A0A140N9N6	2Fe-2S ferredoxin	0.0242548	-1.20652	0.055671	-1.16122
A0A140N7S5	1-acyl-sn-glycerol-3-phosphate acyltransferase	0.850774	-1.01401	0.102498	1.12128
A0A140NBF4	Succinyl-CoA ligase [ADP-forming] subunit beta	0.0142553	1.31409	0.763263	1.03078
A0A140N5S1	Galactonate operon transcriptional repressor	0.0972731	1.59505	0.170578	1.28943
A0A140N8Z8	Lipid A biosynthesis myristoyltransferase	0.752198	-1.04121	0.634876	1.04776
A0A140NDH7	Fructose-1,6-bisphosphatase 1 class 2	0.136955	1.35754	0.570883	-1.08049

A0A140NFZ9	Chaperone protein DnaJ	0.088205	1.11929	0.0185256	-1.18993
A0A140NF32	50S ribosomal protein L11	0.0658781	-1.25598	0.0220288	-1.35636
A0A140NAK0	Murein tetrapeptide carboxypeptidase	0.326711	1.1757	0.057455	1.30944
A0A140N6N7	Sorbitol-6-phosphate 2-dehydrogenase	0.186603	-1.71714	0.184452	1.35963
A0A140NCD5	Cardiolipin synthase A	0.512887	1.12128	0.0239556	1.45959
HEN_OVALBUMIN		0.0385886	-1.17185	0.0514138	1.16634
A0A140N8J5	Hydrogenase isoenzymes formation protein HypE	0.0339008	1.85524	0.0439152	1.71988
A0A140NEY8		0.275683	1.22767	0.555339	1.07979
A0A140NC78	PTS system N-acetylglucosamine-specific EIICBA component;N-acetylglucosamine permease I	0.0303705	1.66564	0.89197	1.02054
A0A140NBX4	Ribonuclease T	0.0106571	-1.3832	0.30896	-1.11224
A0A140NAQ9	tRNA (cmo5U34)-methyltransferase	0.0214395	-1.24476	0.0844047	-1.16584
A0A140NFM6	Cell division protein FtsZ	0.821535	-1.02962	0.510263	-1.06861
A0A140N913	3-oxoacyl-[acyl-carrier-protein] synthase 3	0.65526	-1.03139	0.0110118	-1.23396
A0A140N2Z9	30S ribosomal protein S9	0.811243	1.03417	0.0105228	-1.66137
A0A140NBI5	Tat proofreading chaperone DmsD	0.999548	-1.0001	0.0595894	1.31954
A0A140N5W3	Glutamate-pyruvate aminotransferase AlaA	0.1581	-1.22102	0.15962	1.17354
A0A140NBP4	Ribosomal RNA large subunit methyltransferase F	0.455401	-1.21403	0.116516	1.33806
A0A140N6F1	Hydrogenase-2 large chain	0.231313	-1.19561	0.954229	1.00623
A0A140NAJ9	Oxygen-dependent choline dehydrogenase	0.0327838	1.35533	0.0611419	1.31403
A0A140N7L7	Histidine transport ATP-binding protein HisP	0.00881889	1.98202	0.792566	-1.05374
A0A140N6T6	Siroheme synthase;Uroporphyrinogen-III Cmethyltransferase;Precorrin-2 dehydrogenase;Si	0.395163	1.07261	0.215058	-1.09416

A0A140NCG0	Thiol peroxidase	0.0583798	1.2989	0.0333201	1.37827
A0A140NDG1	Uncharacterized lipoprotein YbjP	0.429098	1.87019	0.0351656	2.56321
A0A140SSC4	tRNA uridine 5-carboxymethylaminomethyl modification enzyme MnmG	0.0131534	-1.64012	0.283435	-1.20463
A0A140NAV6	Periplasmic serine endoprotease DegP	0.0461741	1.51111	0.443634	1.11481
A0A140NFS6	UDP-N-acetylmuramoyl-tripeptide--D-alanyl-Dalanine ligase	0.161545	1.18458	0.920201	1.00923
A0A140N457	Guanylate kinase	0.0621808	-1.32033	0.0635129	-1.2766
A0A140NHI4	Phospholipase A1	0.731463	-1.09172	0.245672	1.22268
A0A140ND17	Protein translocase subunit SecA	0.265463	1.13373	0.454693	-1.06943
A0A140NBG9	Pyridoxamine kinase	0.0242919	-1.17548	0.356238	-1.05486
A0A140SSA2	Glutamine synthetase	0.0393068	2.04014	0.533861	1.13483
A0A140N7B7	Inosine-5-monophosphate dehydrogenase	0.0167272	-1.80472	0.461043	1.19506
A0A140NED8	Metalloprotease PmbA	0.561455	-1.05412	0.739987	-1.02518
A0A140NAF7	Formyltetrahydrofolate deformylase	0.281187	1.17023	0.0247396	1.39054
A0A140SS76	Bifunctional purine biosynthesis protein PurH;Phosphoribosylaminoimidazolecarboxamide f	0.0402813	3.25422	0.0653981	2.16038
B8LFD5	Lactose operon repressor	0.204302	-1.39089	0.0671806	1.448
A0A140ND85	Phosphate-specific transport system accessory protein PhoU	0.497118	1.10156	0.0266111	1.37128
A0A140N3Q3	L-lactate dehydrogenase	0.0831958	-1.49376	0.235067	1.21282
A0A140N8V8		0.3437	-1.12477	0.0882291	-1.20671
A0A140NDD7		0.492589	-1.06404	0.0145938	-1.28918
A0A140NFK2	30S ribosomal protein S2	0.15524	-1.14873	0.0196336	-1.28661

A0A140NC02	Putative ribosome biogenesis GTPase RsgA	0.114536	1.32444	0.0501623	1.3641
B8LFD6	Beta-galactosidase	0.0751964	-2.16628	0.205594	-1.37659
A0A140N8C8	Uncharacterized protein YgiM	0.133031	1.29824	0.176032	1.19973
A0A140NHS0	ATP synthase subunit beta	0.380214	1.06442	0.147643	1.10005
A0A140N5P6	Protein YrdA	0.0761514	1.48324	0.186751	-1.23898
A0A140NF44	HTH-type transcriptional repressor FabR	0.884752	-1.01692	0.53613	1.05939
A0A140NCR3	Protein FdhE	0.410173	1.11889	0.475319	-1.07838

A0A140NAB6	Undecaprenyl phosphate-alpha-4-amino-4deoxy-L-arabinose arabinosyl transferase	0.636303	1.06948	0.0444807	1.30941
A0A140NBQ5	7-alpha-hydroxysteroid dehydrogenase	0.1555	1.62856	0.0353464	1.84106
A0A140N9T6	Ubiquinone biosynthesis O-methyltransferase	0.938988	1.01698	0.219382	1.21682
A0A140NAT5	Peptide transport system ATP-binding protein SapD	0.876543	-1.03127	0.228716	1.1949
A0A140N9F0	Adenosine deaminase	0.0171139	-1.48167	0.270905	-1.17409
A0A140NBW4	Riboflavin synthase	0.686185	1.11918	0.0518346	1.54334
A0A140N6J8	UTP--glucose-1-phosphate uridylyltransferase	0.789866	1.02664	0.480878	1.05991
A0A140N9C4	Outer membrane lipoprotein SlyB	0.421212	1.13455	0.0254469	1.42642
A0A140N5X5	DNA primase	0.884884	-1.01692	0.641053	1.04439
A0A140N6D3	Uncharacterized protein YidR	0.733429	-1.09042	0.0596896	1.47377
A0A140N880	tRNA (mo5U34)-methyltransferase	0.164401	-1.18777	0.0382864	-1.36898
P0AEG4	Thiol:disulfide interchange protein DsbA	0.64786	1.06275	0.0449591	1.29291
A0A140NC56	Purine nucleoside phosphorylase DeoD-type	0.882938	-1.01985	0.295495	1.12034
A0A140NH47	N-acetyl-gamma-glutamyl-phosphate reductase	0.106978	1.63723	0.478212	-1.14371
A0A140ND23	3-oxoacyl-[acyl-carrier-protein] reductase FabG	0.246683	1.13375	0.570038	1.05057
A0A140N810	Transketolase 1	0.358206	-1.09341	0.835766	-1.01652

A0A140N9R2	Phosphoribosylformylglycinamidine cyclo-ligase	0.0213164	2.78745	0.220138	1.48835
A0A140N702	NAD(P) transhydrogenase subunit alpha	0.0497757	1.41395	0.159225	1.21985
A0A140NDW9	2,3,4,5-tetrahydropyridine-2,6-dicarboxylate Nsuccinyltransferase	0.827549	-1.04385	0.274562	1.17375
A0A140N5Q1	tRNA N6-adenosine threonylcarbamoyltransferase	0.167909	-1.19231	0.0918046	-1.20964
A0A140NHM8		0.056569	1.33252	0.809479	-1.04096
A0A140N2P2	Uncharacterized protein YibN	0.322085	1.10621	0.396605	-1.07435
A0A140NCU1	Malonyl CoA-acyl carrier protein transacylase	0.346016	1.10385	0.269253	1.10308
A0A140N544	T-protein;Chorismate mutase;Prephenate dehydrogenase	0.358423	2.28622	0.0267379	4.20131
A0A140NC97	Alkyl hydroperoxide reductase subunit C	0.345292	1.24249	0.087894	1.36698
A0A140NCQ6	Adenylosuccinate lyase	0.115986	1.3715	0.0596948	1.39361
A0A140NDE6	Acetyl-coenzyme A carboxylase carboxyl transferase subunit alpha	0.31135	1.11372	0.024833	-1.29129
A0A140N932	CTP synthase	0.035787	-1.42849	0.163129	-1.21388
A0A140NA84	Phosphate acetyltransferase	0.138356	-1.33446	0.0772489	1.33813
A0A140NDM3	UDP-N-acetylglucosamine--N-acetylmuramyl(pentapeptide) pyrophosphoryl-undecaprenol N-a	0.912923	-1.0129	0.245317	1.12264
A0A140NDD4	Nuclease SbcCD subunit D	0.388272	-1.21278	0.342619	1.1614
A0A140N8E1	Phosphoglycerate kinase	0.881725	-1.03531	0.170707	1.26741
A0A140N719	Ribosome maturation factor RimM	0.0324837	-1.27761	0.171862	-1.16397
A0A140NCU6	Uncharacterized lipoprotein YceB	0.0312312	1.37253	0.155855	1.19396
A0A140NCE9	Malate synthase A	0.646383	2.77869	0.0304774	8.29028
A0A140N4F6	Ribose-5-phosphate isomerase A	0.363153	-1.2658	0.199197	1.26554

A0A140N4P3	4-hydroxy-3-methylbut-2-en-1-yl diphosphate synthase (flavodoxin)	0.881624	-1.01129	0.712288	-1.02434
A0A140NCR7	Recombination protein RecR	0.166969	1.16637	0.248852	1.11324
A0A140N1P7	Sugar phosphatase YidA	0.302475	-1.18255	0.31795	1.13365
A0A140N3T1	Xaa-Pro aminopeptidase	0.695707	1.10792	0.0564166	1.5122
A0A140NEM8	Ferrochelatase	0.344993	1.20301	0.473228	1.10631
A0A140NAZ7	MltA-interacting protein	0.42991	1.12343	0.0541888	1.4869
A0A140NF50	Fumarate reductase iron-sulfur subunit	0.436572	1.48106	0.281456	1.33112
A0A140NB62	23S rRNA (guanine(745)-N(1))methyltransferase	0.896578	-1.0523	0.0316273	2.02854
A0A140NCM7	Protein UshA;UDP-sugar hydrolase;5nucleotidase	0.0259785	1.92585	0.558334	1.12325
A0A140N9Y8	Glucans biosynthesis protein D	0.0353052	1.3349	0.114105	1.21926
P17952	UDP-N-acetylmuramate--L-alanine ligase	0.919602	1.01287	0.772578	-1.0294
A0A140N421	Ribosomal RNA large subunit methyltransferase J	0.149588	-1.23238	0.155613	-1.18715
A0A140N765	Uncharacterized protein YhdP	0.66593	-1.08193	0.0853368	1.30834
A0A140NAT8		0.836401	1.02552	0.270673	-1.20151
A0A140N996	Aspartate--tRNA ligase	0.449565	1.08116	0.0499642	1.22265
A0A140N903	PTS system glucose-specific EIICB component;Glucose permease IIC component;Glucose-spec	0.271507	1.24363	0.638204	-1.06819
A0A140NGG2	FKBP-type 22 kDa peptidyl-prolyl cis-trans isomerase	0.72154	-1.05654	0.016095	-1.58751
A0A140NF19	Ribokinase	0.330084	1.26155	0.483935	1.12063
A0A140NGW4	tRNA-dihydrouridine synthase A	0.0356059	1.18998	0.658395	-1.03494

A0A140NCS6	Cytochrome bo(3) ubiquinol oxidase subunit 2	0.146246	1.16337	0.0665489	1.24862
A0A140N773	Putative membrane protein IgaA homolog	0.0716787	1.38892	0.283123	1.1608
A0A140N8Y2	Thymidylate synthase	0.820687	1.02388	0.76384	-1.0261
A0A140N6C8	Membrane-bound lytic murein transglycosylase A	0.532676	-1.05834	0.0921686	-1.15733
A0A140N5S7	50S ribosomal protein L3 glutamine methyltransferase	0.715861	1.08037	0.0353496	1.52981
A0A140N6J0	DNA-directed RNA polymerase subunit omega	0.203927	-1.18855	0.0421325	-1.315
A0A140N8F4	Probable phosphatase YcdX	0.952131	-1.0114	0.0918107	1.31076
A0A140N9N4	5-deoxynucleotidase YfbR	0.966085	1.01313	0.154648	1.35687
A0A140N9V8	Pyruvate kinase I	0.0552607	-1.61832	0.162717	1.31365
A0A140N9J2	Ribosomal protein S12 methylthiotransferase accessory factor YcaO	0.994496	-1.00055	0.0199072	-1.26349
P60390	Ribosomal RNA small subunit methyltransferase H	0.0324907	1.27298	0.86364	1.0151
A0A140N5I0	FKBP-type peptidyl-prolyl cis-trans isomerase FkpA	0.0599594	1.50549	0.157971	-1.27842
A0A140N5D7	50S ribosomal protein L21	0.554862	1.06249	0.0339535	-1.32467
A0A140NF13	S-(hydroxymethyl)glutathione dehydrogenase	0.47991	1.14598	0.58064	-1.08026
A0A140NFD8	N utilization substance protein B	0.153581	-1.66807	0.135745	-1.45165
A0A140N5F4	Polyribonucleotide nucleotidyltransferase	0.423416	1.06278	0.452237	-1.05128
A0A140NCI4	N5-carboxyaminoimidazole ribonucleotide synthase	0.0441061	2.53806	0.142174	1.66772
A0A140N8Q9	Asparagine--tRNA ligase	0.0666162	-1.14456	0.0970769	-1.12182
A0A140N9G9	3-oxoacyl-[acyl-carrier-protein] synthase 1	0.113847	1.22776	0.156011	-1.17091
A0A140N8V2	Septum site-determining protein MinD	0.0823922	1.39091	0.171035	1.23294

A0A140N3F8	3,4-dihydroxy-2-butanone 4-phosphate synthase	0.27582	-1.6815	0.305701	1.31116
A0A140N613	UPF0701 protein YicC	0.58922	-1.05776	0.639038	1.04131
A0A140NCP8	Ribosomal silencing factor RsfS	0.749781	1.29514	0.0707406	2.22427
A0A140NHS9	Sensor protein CpxA	0.591177	1.07616	0.873574	-1.01714
A0A140N583	Bifunctional protein HldE;D-beta-D-heptose 7phosphate kinase;D-beta-D-heptose 1-phosph	0.292953	1.12429	0.70974	1.03404
A0A140N536	Signal recognition particle protein	0.550196	-1.05875	0.0251788	-1.27058
A0A140N8F2	7-carboxy-7-deazaguanine synthase	0.861808	-1.03184	0.345257	-1.13962
A0A140N7G1	DnaA initiator-associating protein DiaA	0.0304923	1.29951	0.299689	1.11092
A0A140N4V0	Nitrogen regulatory protein	0.785037	-1.04353	0.0939406	1.26285
A0A140N6Y7	Transcriptional regulatory protein GlrR	0.736748	-1.05346	0.898511	1.01519
A0A140N6H9	tRNA (cytidine/uridine-2-O-)-methyltransferase TrmJ	0.108837	-1.24197	0.218299	1.14837
A0A140NF78	Cell division protein FtsN	0.586624	-1.16903	0.0515261	-1.61757
A0A140N9P3	NADH-quinone oxidoreductase subunit G	0.527663	-1.04499	0.0467081	1.15715
A0A140N5X0	UDP-N-acetylglucosamine 1carboxyvinyltransferase	0.886578	1.02515	0.0225017	-1.56237
A0A140NAP2	Spermidine/putrescine import ATP-binding protein PotA	0.241668	1.14962	0.77983	1.04073
A0A140N9V2	Tryptophan synthase alpha chain	0.16988	1.56846	0.101396	1.50681
A0A140NBR4	Chromosome partition protein MukB	0.930929	-1.00933	0.489685	1.06436
A0A140N8C6	Methionine--tRNA ligase	0.682635	-1.03018	0.532256	1.04101
A0A140N2N4	Uncharacterized ABC transporter ATP-binding protein YheS	0.079887	-1.21613	0.331323	-1.09361
A0A140N783	Glyceraldehyde-3-phosphate dehydrogenase A	0.472862	-1.12763	0.0924437	1.28248
A0A140N599	DNA topoisomerase 4 subunit B	0.194306	-1.33112	0.0420415	-1.73655

A0A140NFV3	Chaperone protein DnaK	0.757913	1.03519	0.0714377	-1.21674
A0A140NAC7	UDP-glucose 6-dehydrogenase	0.847513	-1.02437	0.741352	1.03383
A0A140NFS3	HTH-type transcriptional repressor CytR	0.938352	1.01656	0.295196	1.1813
A0A140NCN5	DNA polymerase III subunit tau	0.598616	1.06773	0.121474	1.19078
P04846	Lipoprotein 28	0.280494	1.27214	0.167698	1.27125
A0A140N738	Cell division protein FtsX	0.0821529	1.22132	0.481658	1.06727
A0A140N7Y4	Ribosomal small subunit pseudouridine synthase A	0.280086	-1.17406	0.360549	1.11445
A0A140NGH1	30S ribosomal protein S18	0.578333	1.17083	0.0229478	-2.22382
A0A140N5E8	Phosphoglucosamine mutase	0.0746263	1.16455	0.239565	1.09119
A0A140SSA1	Protein HemY	0.811416	1.07448	0.0807709	1.51619
A0A140N5P0	Ribosomal RNA small subunit methyltransferase I	0.373585	-1.12889	0.284848	1.12828
A0A140N8I2	Uncharacterized HTH-type transcriptional regulator YciT	0.236153	1.09664	0.174328	1.10203
A0A140NA37	Protein YcgL	0.199836	1.13347	0.294203	1.09218
A0A140NAL9	ABC transporter ATP-binding protein uup	0.125463	-1.27507	0.110867	-1.34537
A0A140N9U2	Uncharacterized oxidoreductase YciK	0.0266682	1.41511	0.521502	1.08445
A0A140NCN6	Uncharacterized protein YiiQ	0.487946	1.20654	0.217792	-1.27244
A0A140NB60	RNA polymerase-associated protein RapA	0.147356	-1.19789	0.0480053	-1.29825
A0A140N9F1	Long-chain fatty acid transport protein	0.078762	2.56412	0.08735	2.85595
A0A140N6F8	Outer membrane protein slp	0.629261	-1.89371	0.0642108	3.17203
A0A140N3H4	50S ribosomal protein L14	0.385439	-1.12081	0.0506245	-1.38612
A0A140N9H5	Uncharacterized tRNA/rRNA methyltransferase YfiF	0.198028	1.15088	0.262918	1.1104

A0A140NBC4	Outer-membrane lipoprotein carrier protein	0.10537	1.21691	0.601731	1.05162
A0A140NBF5	Serine--tRNA ligase	0.608216	-1.06547	0.67357	-1.04317
A0A140NE11	Oxygen-insensitive NADPH nitroreductase	0.389081	1.17289	0.326833	1.15074
A0A140N969	Phospho-2-dehydro-3-deoxyheptonate aldolase, Trp-sensitive	0.0512078	-1.30458	0.159987	-1.18839
A0A140NCZ5	UPF0092 membrane protein YajC	0.12447	1.29975	0.115499	1.26574
A0A140NDR0	Uncharacterized lipoprotein YmbA	0.541903	-1.06961	0.0414603	1.26567
A0A140N7B6	GTPase ObgE/CgtA	0.0266744	-1.2011	0.702449	-1.0264
P23003	tRNA/tmRNA (uracil-C(5))-methyltransferase	0.124925	-1.21357	0.181064	-1.20951
A0A140N537	30S ribosomal protein S8	0.975018	-1.0039	0.0293872	-1.41431
A0A140N4Z5	Cyclic di-GMP-binding protein	0.0619056	1.42252	0.587626	1.07964
A0A140N587	Bifunctional polymyxin resistance protein ArnA;UDP-4-amino-4-deoxy-L-arabinose formyltr	0.928164	1.01465	0.245749	1.16653
A0A140N940	Uncharacterized protein YfgD	0.438695	1.21486	0.419282	1.15343
A0A140N8K1	Uncharacterized HTH-type transcriptional regulator YeiE	0.428464	1.20686	0.870372	1.02706
A0A140N5H6	Biosynthetic arginine decarboxylase	0.45358	1.07944	0.581852	-1.04841
A0A140N6M4	ADP-L-glycero-D-manno-heptose-6-epimerase	0.643457	-1.07478	0.612759	1.06348
A0A140N9R4	Translation initiation factor IF-3;Translation initiation factor IF-3, N-terminally pro	0.270488	1.16016	0.522815	1.07149
A0A140N5Q7	Inorganic triphosphatase	0.059679	1.22523	0.701804	1.03369
A0A140N5C1	Ribosomal RNA small subunit methyltransferase B	0.197442	-1.21102	0.873914	1.0183
A0A140NC86	Magnesium and cobalt efflux protein CorC	0.9335	-1.04095	0.117571	1.63325
A0A140NDN5	Nitrogen regulation protein NR(I)	0.0358419	2.5656	0.646346	1.1431

A0A140NCH5	Cation efflux system protein CusB	0.0878614	-1.8396	0.990269	1.0027
A0A140SS75	ATP-dependent 6-phosphofructokinase isozyme 1	0.595143	1.17188	0.0648615	1.5979
A0A140NAB7	Glutamine--tRNA ligase	0.06324	-1.21538	0.284679	-1.10067
A0A140N6A5	Chaperone protein ClpB	0.701952	1.04853	0.508259	1.07009
A0A140N9D3	Phosphocarrier protein HPr	0.752144	-1.07803	0.0893601	1.40811
A0A140SSB1	Ribosomal RNA small subunit methyltransferase G	0.143194	-1.19373	0.371837	-1.09348
A0A140N5A9	Fe/S biogenesis protein NfuA	0.97712	1.00618	0.0266427	-1.74228
A0A140N6R1	DNA-binding protein StpA	0.465063	-1.09353	0.558358	1.0606
A0A140NBR2	Probable L,D-transpeptidase YbiS	0.136399	-1.21328	0.351622	-1.14682
A0A140N6X2	Electron transport complex subunit RsxG	0.866369	1.02731	0.0424266	1.39405
A0A140NAW7	Tail-specific protease	0.629668	-1.04903	0.387086	-1.0776
A0A140NGF2	UPF0053 inner membrane protein YtfL	0.550382	-1.10404	0.716177	-1.04732
A0A140SS57	Phosphoribosylamine--glycine ligase	0.0536147	2.73995	0.192976	1.69601
A0A140NF57	Hydrogen peroxide-inducible genes activator	0.0833038	1.47581	0.224903	1.24355
A0A140ND53	Zinc uptake regulation protein	0.373763	1.25612	0.0487857	1.627
A0A140N820	Sensor histidine kinase RcsC	0.906143	-1.01782	0.080228	1.27956
A0A140N7S3	Probable quinol monooxygenase YgiN	0.960487	-1.01061	0.291873	1.18795
A0A140N683	RNA polymerase sigma factor RpoD	0.50081	1.16497	0.0338913	-1.69767
A0A140NAL3	Heat shock protein HslJ	0.0341975	-1.58066	0.969856	1.00628
A0A140NAK1	HTH-type transcriptional regulator CysB	0.0511238	1.43666	0.275974	-1.22065
A0A140NFR6	Chaperone SurA	0.167585	1.12659	0.647535	-1.03386
A0A140N831	Periplasmic beta-glucosidase	0.484297	1.08005	0.247591	-1.119

A0A140N9Z8	Multiphosphoryl transfer protein;Phosphocarrier protein HPr;Fructosespecific phosphotr	0.098021	-1.40307	0.101761	1.42061
A0A140NBK3	Flavodoxin-1	0.768917	1.06305	0.320428	1.17214
Q5H772	4-aminobutyrate aminotransferase PuuE	0.0317716	2.69455	0.819175	-1.09987
A0A140N3Z1	Protein YhjJ	0.238753	1.15718	0.289885	1.11798
A0A140NEN8	Negative modulator of initiation of replication	0.0875743	-1.64932	0.294814	-1.25496
A0A140NEY7	Farnesyl diphosphate synthase	0.502544	-1.18132	0.592506	1.09776
A0A140NCV3	Peptidyl-prolyl cis-trans isomerase D	0.0853364	1.21465	0.908754	1.01047
A0A140N4K1	30S ribosomal protein S3	0.29711	-1.21958	0.0453576	-1.51275
A0A140N4Z4	Glycerol-3-phosphate regulon repressor	0.0411313	1.27793	0.714804	1.03633
A0A140NBA5	30S ribosomal protein S1	0.270354	-1.10131	0.0534566	-1.1955
A0A140N7L1	p-aminobenzoyl-glutamate hydrolase subunit A	0.251565	1.88203	0.403548	-1.28595
A0A140N9L7	N-acetylglucosamine repressor	0.591039	-1.10314	0.201237	1.21351
A0A140N9G6	Outer membrane protein assembly factor BamD	0.142322	1.16782	0.532502	-1.05646
A0A140NG24	Poly(A) polymerase I	0.901155	-1.01117	0.0313612	-1.25455
A0A140NFF4	Queuine tRNA-ribosyltransferase	0.345011	-1.20653	0.14623	-1.48915
A0A140N4F2	Cold shock protein CspA	0.112035	-1.78676	0.0949955	-1.82951
A0A140NGH6	PTS system trehalose-specific EIIBC component;Trehalose-specific phosphotransferase enz	0.163667	-2.05904	0.152187	-1.65956
A0A140NH17	Uroporphyrinogen decarboxylase	0.177248	1.17788	0.520045	-1.06628
A0A140NCI6	Elongation factor Tu 2;Elongation factor Tu 1	0.902338	-1.00777	0.0698734	-1.12756
A0A140NE54	UPF0250 protein YbeD	0.409639	-1.10992	0.519124	-1.06973

A0A140NEQ9	Transcriptional regulatory protein BasR	0.13497	1.13565	0.437626	-1.05855
A0A140NFY3		0.0865598	1.71307	0.739381	1.0732
A0A140NFX6	Membrane protein insertase YidC	0.0579094	-1.16794	0.231588	-1.09393
A0A140N4C7	Bifunctional (p)ppGpp synthase/hydrolase SpoT;GTP pyrophosphokinase;Guanosine-3,5bis(d	0.78178	1.03926	0.172743	1.18359
A0A140NAY2	DNA polymerase III subunit delta	0.870233	-1.02071	0.260724	1.13125
A0A140NG19	NAD(P)H-flavin reductase	0.292003	-1.17486	0.288594	1.1445
A0A140NCY5	Thiamine-monophosphate kinase	0.942092	-1.01085	0.0383761	1.4008
A0A140NB90	Copper homeostasis protein CutC	0.444813	1.16818	0.677955	1.06472
A0A140N9H2	D-serine dehydratase	0.782811	-1.11236	0.220094	1.37575
A0A140N8B7	Cysteine desulfurase CsdA	0.0413009	-1.35647	0.475465	-1.09695
A0A140NB77		0.589253	-1.07264	0.309763	-1.11971
A0A140NIC8	tRNA modification GTPase MnmE	0.482219	-1.11116	0.243942	-1.16043
A0A140N866	Uncharacterized Nudix hydrolase YfcD	0.296232	-1.1414	0.711869	-1.03882
A0A140ND72	ATP synthase subunit alpha	0.25046	1.10991	0.388134	1.07095
A0A140N8V0	dTDP-glucose 4,6-dehydratase 1	0.784086	1.05432	0.305744	1.17052
A0A140ND51	Thiamine-binding periplasmic protein	0.437981	-1.24316	0.153457	1.37062
A0A140NBN8	D-alanyl-D-alanine carboxypeptidase DacA	0.511545	-1.06574	0.0416957	-1.25767
A0A140N4B1		0.232464	1.57467	0.114206	-1.61207
A0A140N883		0.832053	-1.05312	0.529201	1.11705
A0A140ND50	Ribosomal RNA large subunit methyltransferase K/L;23S rRNA m2G2445 methyltransferase;23	0.0838421	-1.21207	0.243431	-1.13984
A0A140N1Z5	tRNA (cytidine(34)-2-O)-methyltransferase	0.0742219	-1.22338	0.235002	-1.12768
A0A140N8N1	Multidrug export protein EmrA	0.525971	-1.06802	0.953765	1.00509

A0A140SS71	50S ribosomal protein L31	0.747628	-1.07546	0.0524355	-1.83547
A0A140NDB7	UPF0246 protein YaaA	0.860667	1.02157	0.149821	1.17568
A0A140N8U6	Phospho-2-dehydro-3-deoxyheptonate aldolase, Tyr-sensitive	0.537861	4.58899	0.0677103	8.45259
A0A140N448	Lead, cadmium, zinc and mercury-transporting ATPase	0.184433	1.23869	0.769784	-1.03744
A0A140N7A5	Nucleoside diphosphate kinase	0.0680056	1.41523	0.467258	1.15376
A0A140NCG3	Uncharacterized protein YjaG	0.681745	-1.04886	0.307617	1.10969
A0A140NAT6	Phosphogluconate dehydratase	0.269001	-1.08469	0.075877	-1.15628
A0A140NBM4	Bifunctional uridylyltransferase/uridylylremoving enzyme;[Protein-PII] uridylyltransfe	0.699922	1.07162	0.440415	1.11591
A0A140NFB9	Formate dehydrogenase-O major subunit	0.489802	-1.06246	0.397061	1.06856
A0A140NAT1		0.378205	1.10634	0.112479	1.19047
A0A140NCV4	S-adenosylmethionine:tRNA ribosyltransferaseisomerase	0.304034	-1.26496	0.0613021	-1.64772
A0A140NB97	Uncharacterized deoxyribonuclease YcfH	0.116221	-1.29611	0.642474	1.06222
A0A140NF33	HTH-type transcriptional repressor NsrR	0.826999	1.04211	0.796279	1.03786
A0A140NC43	Uncharacterized protein YbgK	0.089537	1.40433	0.857529	1.02769
A0A140NHC7	GTP-binding protein TypA/BipA	0.0525276	-1.28486	0.581601	-1.06174
A0A140N4C9	Ribosomal RNA small subunit methyltransferase E	0.143903	-1.18413	0.961614	-1.00457
A0A140NH75	Cell division protein ZapB	0.389376	1.22184	0.79852	1.0439
A0A140NHQ8	ATP-dependent protease subunit HslV	0.454563	1.23334	0.0724814	-1.60066
A0A140N6E2		0.0573491	-2.07543	0.913508	-1.04634
A0A140N8B9		0.170583	-1.31379	0.0938112	-1.40457
A0A140NEC8	DNA polymerase III subunit delta	0.405098	-1.07507	0.346491	1.07647

A0A140NEF0		0.377093	-1.18074	0.104407	-1.47859
A0A140NFP6	Peptidyl-prolyl cis-trans isomerase C	0.507076	1.08532	0.367953	1.10049
A0A140NB75	Delta-aminolevulinic acid dehydratase	0.392189	-1.20356	0.773743	1.04715
A0A140NAN5	Outer membrane protein F	0.78619	1.06273	0.830357	1.03582
A0A140NAY7	Uncharacterized lipoprotein YbaY	0.634097	2.66457	0.123324	3.12646
A0A140NDX6	dTDP-glucose 4,6-dehydratase 2	0.0748323	-1.26426	0.751929	-1.03459
Q46920	NADPH-dependent 7-cyano-7-deazaguanine reductase	0.346786	1.14291	0.104507	-1.24738
A0A140SS46	tRNA dimethylallyltransferase	0.235514	-1.15778	0.973966	-1.00331
A0A140NEK4	Modulator of FtsH protease HflC	0.142695	1.24279	0.294112	1.14225
A0A140NBN7	Tyrosine--tRNA ligase	0.931672	1.01158	0.109202	1.22864
A0A140NEK3	Regulator of ribonuclease activity A	0.525295	1.20632	0.0917498	1.55117
A0A140NA12	Outer-membrane lipoprotein LolB	0.394663	-1.34158	0.278374	1.30521
A0A140N9F5	Outer membrane protein assembly factor BamB	0.869562	1.02388	0.846582	-1.02286
A0A140NBB9	Flagellar L-ring protein	0.119062	-1.87975	0.430739	-1.55879
A0A140N665	Putative lipoprotein AcfD homolog	0.282137	-1.32784	0.074127	-1.66072
A0A140NF64	Cytosine deaminase	0.885746	1.01923	0.0674184	1.28445
A0A140N9E5	Glutamate--tRNA ligase	0.0515414	-1.20721	0.704916	1.03336
A0A140N4R6	Uncharacterized protein YibL	0.232517	-1.16158	0.751504	1.03332
A0A140N452	Sensor protein QseC	0.118756	1.39243	0.452008	-1.13452
A0A140N4Q1	Lipopolysaccharide heptosyltransferase 1	0.714169	-1.17479	0.23366	1.41983
A0A140N2X4	Ribosomal protein L11 methyltransferase	0.203008	-1.16846	0.6111	-1.05327
A0A140N3L9	50S ribosomal protein L28	0.50571	-1.20935	0.0570802	-1.87669
A0A140NA93	UPF0053 protein YegH	0.861348	1.03812	0.126477	1.33566

A0A140N2S3	50S ribosomal protein L22	0.369611	-1.15218	0.520377	1.13534
A0A140NGI8	DNA polymerase III subunit psi	0.489033	-1.121	0.155409	1.23533
A0A140NBN5	3-hydroxydecanoyl-[acyl-carrier-protein] dehydratase	0.146857	1.45612	0.17349	-1.48753
A0A140N8S7	Cytidylate kinase	0.132122	-1.11092	0.461077	-1.04683
A0A140N814	S-formylglutathione hydrolase YeiG	0.866931	1.70406	0.126883	4.05408
A0A140N745		0.838328	1.01437	0.275955	1.07513
A0A140NHJ4	DNA helicase II	0.229677	-1.2275	0.440638	-1.11337
A0A140N6Y2	Positive transcription regulator EvgA	0.0764453	1.40307	0.431245	1.13616
A0A140NDZ9	Succinate dehydrogenase iron-sulfur subunit	0.14007	1.27762	0.768942	1.05559
A0A140N6N0	Phenylalanine--tRNA ligase alpha subunit	0.14274	1.1447	0.153438	-1.14097

A0A140NDV6	Uncharacterized protein YigA	0.13061	-1.46839	0.242253	-1.28732
A0A140NAM8	Glutamate 5-kinase	0.42469	-1.14141	0.680314	1.05618
A0A140NE46	D-alanyl-D-alanine carboxypeptidase DacC	0.106741	1.42475	0.304022	1.21193
A0A140N7T4	DNA topoisomerase 4 subunit A	0.669844	1.06932	0.462728	-1.10036
A0A140N539	Bifunctional protein FolC;Folylpolyglutamate synthase;Dihydrofolate synthase	0.0689453	-1.23393	0.577981	1.05721
A0A140N8P0	RecBCD enzyme subunit RecC	0.0751564	-1.84364	0.553363	1.23362
A0A140N6X6	Cellulose synthase operon protein C	0.672503	1.13559	0.474188	1.16535
A0A140N711	50S ribosomal protein L15	0.470614	-1.10372	0.0961088	-1.31092
A0A140N9G0	Dihydroorotate dehydrogenase (quinone)	0.425673	-1.05981	0.760583	-1.02454
A0A140N9G2	Succinyl-CoA ligase [ADP-forming] subunit alpha	0.224739	1.16018	0.129389	1.2229
A0A140NG68	Cell division protein ZapD	0.688461	-1.08893	0.155274	1.30296
A0A140NCW5	Uncharacterized lipoprotein YajG	0.4573	-1.12562	0.0816544	-1.38496
A0A140N4N0	Orotate phosphoribosyltransferase	0.135621	-1.19535	0.188557	-1.16255

A0A140N598	50S ribosomal protein L13	0.258234	-1.31128	0.103563	-1.48895
A0A140N5K8	50S ribosomal protein L4	0.757722	1.07487	0.0825089	-1.71412
A0A140NFE6	5-methylthioadenosine/Sadenosylhomocysteine nucleosidase	0.711563	-1.03717	0.599591	-1.04664
A0A140N6R9	Shikimate kinase 1	0.750325	1.03397	0.0687637	-1.25022
A0A140NA72	Uncharacterized protease YegQ	0.79017	-1.03418	0.937099	1.01233
A0A140NFC7	3-hydroxyacyl-[acyl-carrier-protein] dehydratase FabZ	0.452934	-1.25456	0.595105	1.11992
A0A140N8Q6	2-dehydro-3-deoxyphosphooctonate aldolase	0.45669	-1.43918	0.175194	1.61337
A0A140NH27	DNA-directed RNA polymerase subunit beta	0.226229	-1.10798	0.423304	-1.0627
A0A140NDT9	Chromosome partition protein MukF	0.722885	-1.03588	0.649268	-1.04048
A0A140N604	Penicillin-binding protein activator LpoA	0.517135	1.05788	0.667132	-1.03369
A0A140N394	Transcriptional regulatory protein OmpR	0.233288	1.33733	0.323838	1.21318
A0A140N4M0	50S ribosomal protein L17	0.269768	-1.22016	0.109974	-1.36774
A0A140N7Z6	Glutamyl-tRNA reductase	0.304495	-1.37857	0.815022	-1.05213
A0A140NI35	Magnesium transport protein CorA	0.20787	1.21094	0.192227	1.24387
A0A140NGF0	Uncharacterized protein YtfJ	0.0784366	1.98395	0.455332	1.29958
A0A140NBF1	Glucans biosynthesis protein G	0.890496	-1.01343	0.265256	1.10581
A0A140N1Y4	L-threonine 3-dehydrogenase	0.490747	1.14569	0.858943	1.02755
A0A140NBS9	UPF0307 protein YjgA	0.346553	1.15062	0.14014	1.23351
A0A140N692	Uncharacterized protein YiaF	0.522561	1.13299	0.763091	1.04703
A0A140N2T1	50S ribosomal protein L6	0.769396	-1.05596	0.293465	-1.30105
A0A140N9P2	Rare lipoprotein A	0.431729	1.35958	0.13354	1.63388
A0A140NBS6	Lipid A export ATP-binding/permease protein MsbA	0.326708	1.1123	0.104741	-1.20051

A0A140N5C8	Uncharacterized lipoprotein YfhM	0.33453	1.21886	0.92587	1.01483
A0A140N997	L-asparaginase 1	0.239994	-1.1074	0.135109	-1.13971
A0A140N793	Chaperone protein HscA	0.167682	-1.19721	0.230155	-1.17822
A0A140N7Q2	Probable phospholipid-binding protein MlaC	0.18147	1.26603	0.966264	-1.00598
A0A140NDH1	Glutamate-1-semialdehyde 2,1-aminomutase	0.891191	1.01467	0.931395	1.00797
A0A140NDN1	Sugar phosphatase YbiV	0.29621	-1.26356	0.372446	1.17472
CON__P00761		0.912832	1.01807	0.0759022	-1.41492
A0A140NCR0	Ribonuclease R	0.246411	1.41288	0.155657	1.47011
A0A140NB71	Sensor protein PhoQ	0.761389	1.06221	0.373787	1.15564
A0A140N4V5	Probable phospholipid ABC transporter permease protein MlaE	0.724249	-1.31113	0.115179	-2.42123
A0A140NBM6	Methylglyoxal synthase	0.557889	1.24019	0.646735	1.11935
A0A140NAU8	Ribosomal-protein-alanine acetyltransferase	0.665331	1.12052	0.225068	1.29874
A0A140N6B2	Uncharacterized protein YggE	0.299045	1.22882	0.366776	-1.15984
A0A140NFT4	Carbamoyl-phosphate synthase small chain	0.119574	1.61869	0.840586	1.04737
A0A140N9D9	2,3-bisphosphoglycerate-dependent phosphoglycerate mutase	0.578682	1.15506	0.630934	1.09706
A0A140NA53	Adenine phosphoribosyltransferase	0.636455	1.03844	0.0771496	-1.16865
A0A140N8X8	Protein YecM	0.739188	1.04948	0.339629	1.12874
A0A140NFX2	Probable phosphoglycerate mutase GpmB	0.760708	1.36812	0.107455	-3.34068
A0A140NAA3	Riboflavin biosynthesis protein RibD;Diaminohydroxyphosphoribosylaminopyrimidine deamin	0.997776	1.00042	0.113354	1.26231
A0A140NGH2	Energy-dependent translational throttle protein EttA	0.187386	1.16352	0.861365	1.0172

A0A140N5X9	Ribosomal RNA large subunit methyltransferase E	0.168525	1.25546	0.244847	-1.19319
A0A140NFV5	LPS-assembly protein LptD	0.684918	1.04912	0.892437	1.01378
A0A140NF41	ATP synthase gamma chain	0.771103	1.05098	0.276716	1.17568
A0A140NBC0	DNA repair protein RadA	0.0961985	1.19567	0.580972	1.05388
A0A140NCD3	Recombination-associated protein RdgC	0.123591	-1.13602	0.705261	-1.03113
A0A140NAC6	Lipid A biosynthesis lauroyltransferase	0.893538	1.03501	0.251699	1.27214
A0A140NAT4	Macrolide export protein MacA	0.762528	1.52837	0.127913	3.55617
A0A140NDX7	Trifunctional NAD biosynthesis/regulator protein NadR;Transcriptional regulator NadR;Ni	0.464967	1.13954	0.889907	1.02001
A0A140NAP6	Nitrate/nitrite response regulator protein NarL	0.41804	1.21537	0.192094	1.31568
A0A140NFT6	ATP synthase subunit delta	0.741068	1.04476	0.461113	1.0886
A0A140N8H2	Nucleoid-associated protein YejK	0.848784	1.0125	0.198517	-1.08659
A0A140NAM6	Uncharacterized protein YecJ	0.820979	1.03424	0.902582	-1.01521
A0A140N7F3	Protein YffB	0.496132	1.13184	0.779205	1.04181
A0A140N9E7	ATP-dependent RNA helicase HrpA	0.85298	1.02101	0.111888	1.19948
A0A140N6F4	Flavohemoprotein	0.829412	-1.04909	0.297878	1.21166
A0A140NEK1	Adenylosuccinate synthetase	0.261592	1.21069	0.793916	1.03691
A0A140NBJ5	Outer membrane lipoprotein RcsF	0.617238	1.12203	0.173187	1.32338
A0A140NDW0	Regulator of sigma-E protease RseP	0.858886	1.03698	0.3943	1.15385
A0A140N9Z7	NADH-quinone oxidoreductase subunit F	0.712645	-1.02172	0.946206	1.00365
A0A140N1Q5	Small heat shock protein IbpA	0.524645	1.17975	0.10757	-1.53527
P00957	Alanine--tRNA ligase	0.823414	-1.02469	0.251077	-1.125
A0A140N9I5	D-erythro-7,8-dihydroneopterin triphosphate epimerase	0.700122	1.13859	0.343546	1.27004

A0A140NEX7	ABC transporter periplasmic-binding protein YtfQ	0.918696	8.6363	0.15141	21.9908
A0A140N4Y2		0.546491	-1.14758	0.525622	1.12135
A0A140N3P1	UPF0001 protein YggS	0.251988	-1.10839	0.660388	1.03566
A0A140NCJ1	Peptide transport periplasmic protein SapA	0.815305	-1.04018	0.0969153	1.34324
A0A140N621	Phosphopantetheine adenylyltransferase	0.239375	1.20933	0.328957	1.15035
A0A140NDL3	Outer membrane protein X	0.140989	-2.57457	0.333512	1.80807
A0A140NEX9	Replicative DNA helicase	0.374702	-1.11014	0.75027	1.03292
A0A140N3D6	cAMP-activated global transcriptional regulator CRP	0.576777	1.13227	0.135491	1.37608
A0A140NEB2	4-hydroxy-tetrahydrodipicolinate reductase	0.476481	1.136	0.682253	-1.06182
A0A140NDY4	Guanosine-5-triphosphate,3-diphosphate pyrophosphatase	0.281721	-1.18083	0.806007	-1.03212
A0A140N739	Thioredoxin-2	0.585861	1.28087	0.134062	1.82935
A0A140NE66	2-oxoglutarate dehydrogenase E1 component	0.979705	1.00217	0.657738	-1.04349
A0A140N7J9	Biotin carboxylase	0.292238	-1.13443	0.518266	-1.08861
A0A140N626	DNA gyrase subunit A	0.188348	1.11204	0.555264	1.04389
A0A140NAQ7	Uncharacterized protein YcjX	0.307616	1.1198	0.574975	1.05631
A0A140N827	D-erythrose-4-phosphate dehydrogenase	0.244168	1.22031	0.396629	-1.13401
A0A140NA83	Enoyl-[acyl-carrier-protein] reductase [NADH] FabI	0.416548	-1.11346	0.400635	-1.10382
A0A140NHU6	Formate dehydrogenase-O iron-sulfur subunit	0.293924	-1.40752	0.438849	-1.21335
A0A140N7Y8	Octaprenyl-diphosphate synthase	0.983451	-1.00362	0.315699	-1.16514
A0A140N958	Uncharacterized protein YpfJ	0.794474	1.06892	0.198353	1.33296
A0A140N825	Uncharacterized protein YcgM	0.319186	1.34782	0.163157	1.55414

A0A140N6P4	Nucleoside triphosphate pyrophosphohydrolase	0.975485	-1.00658	0.343267	1.18501
A0A140SS92	Sulfate-binding protein	0.655216	-2.16968	0.263295	-2.23011
A0A140NFM4	Protein CyaY	0.683059	-1.08362	0.8252	1.03546
A0A140N6E7	Peptide chain release factor 2	0.983679	-1.0024	0.101514	-1.23024
A0A140NA85	Deoxycytidine triphosphate deaminase	0.781836	1.08432	0.218455	-1.35465
A0A140NDV9	Transaldolase B	0.848937	-1.02307	0.50156	1.07422
A0A140NDX1	Replication-associated recombination protein A	0.519179	1.12523	0.592003	1.08481
A0A140NBN4	Iron-sulfur cluster insertion protein ErpA	0.216314	-1.25314	0.750963	1.04938
A0A140N4K8	Nitrogen regulatory protein P-II 1	0.288184	1.1864	0.732598	1.04737
A0A140N7T3		0.126373	1.23831	0.565495	1.07668
A0A140SS28	Elongation factor P--(R)-beta-lysine ligase	0.233447	-1.16512	0.316738	-1.12704
A0A140N9U0	Nitrate/nitrite response regulator protein NarP	0.910423	1.45016	0.170048	6.78088
A0A140SS80	DNA-directed RNA polymerase subunit beta	0.765853	-1.02502	0.200322	-1.1112
A0A140NGD3	Phosphate-binding protein PstS	0.603872	1.1214	0.137496	1.39273
A0A140N5B7	Iron-binding protein IscA	0.147106	-1.72703	0.567595	-1.25414
A0A140NES8	Penicillin-binding protein 2	0.60505	-1.14864	0.314237	1.25045
A0A140N7C1	Tryptophan--tRNA ligase	0.795583	1.04477	0.839723	1.02881
A0A140N3G7	50S ribosomal protein L3	0.914598	1.09248	0.135263	-2.89657
A0A140NH65	60 kDa chaperonin	0.197598	1.24728	0.697051	-1.05851
A0A140N690	Glycine--tRNA ligase alpha subunit	0.326075	-1.1301	0.58876	-1.06111
A0A140N998	Ribonuclease 3	0.160286	-1.26979	0.443163	-1.1283
A0A140NBL4	Outer membrane protein assembly factor BamA	0.175563	1.23714	0.49398	1.10131
A0A140NEW8	ATP-dependent Clp protease proteolytic subunit	0.326795	1.23822	0.524211	1.12219

A0A140N8S5		0.760582	-1.10656	0.295171	-1.32176
A0A140N6X3	Glutamate-pyruvate aminotransferase AlaC	0.674395	1.10082	0.484604	1.13954
A0A140NDS7	Peptide methionine sulfoxide reductase MsrA	0.550887	-1.83174	0.153664	3.81645
A0A140N8V3		0.606152	-1.17024	0.504432	1.1691
A0A140N6J7	Prolipoprotein diacylglyceryl transferase	0.513523	-1.09317	0.798134	-1.03052
A0A140SS74	Ribosomal large subunit pseudouridine synthase F	0.334688	1.35591	0.326649	1.29565
A0A140N733	2-C-methyl-D-erythritol 4-phosphate cytidylyltransferase	0.617491	-1.108	0.483403	-1.12778
A0A140NC45	Uncharacterized ABC transporter ATP-binding protein YbiT	0.556259	1.08944	0.179421	-1.24703
A0A140N2D9	Chromosomal replication initiator protein DnaA	0.412826	-1.14589	0.478422	-1.10787
A0A140N4G2	Protein-export protein SecB	0.232251	1.17575	0.911566	-1.01313
A0A140NF03	Acetate operon repressor	0.39922	1.17984	0.524915	-1.1111
A0A140N4N4	Peptidase B	0.505461	1.05147	0.355436	-1.06879
A0A140NBS1	50S ribosomal protein L20	0.813151	-1.05935	0.639743	-1.17041
A0A140NBS0	DNA replication terminus site-binding protein	0.72759	1.07535	0.257984	1.236
A0A140N5X6	NADH-quinone oxidoreductase subunit H	0.816771	-1.04835	0.392234	1.16094
A0A140N2L7	Inactive ribonuclease PH	0.433635	-1.05819	0.196834	-1.1037
A0A140N779	Undecaprenyl-phosphate 4-deoxy-4formamido-L-arabinose transferase	0.817953	1.03716	0.905374	1.01607
A0A140NHL8	Transcription termination/antitermination protein NusG	0.690926	1.03444	0.433463	1.06465
A0A140NDX4	Dihydrolipoyllysine-residue succinyltransferase component of 2-oxoglutarate dehydrogena	0.220874	1.16344	0.561587	-1.07651
A0A140N9F2	Protein TolR	0.155395	-1.41992	0.590798	-1.1308

A0A140NAU1	Uncharacterized protein YbjS	0.137492	-1.18567	0.872097	1.01714
A0A140NDC8	Ribonuclease HII	0.724061	-1.06917	0.468942	-1.12461
A0A140NCF6		0.657508	-1.07472	0.330896	1.20621
A0A140NDA8		0.61373	-1.08024	0.947907	1.00856
A0A140N7C5	Transcription elongation factor GreA	0.151813	1.18974	0.829094	-1.02547
A0A140NFM9	UDP-N-acetylglucosamine 2-epimerase	0.591459	-1.13975	0.457932	1.16135
A0A140NG32	Pantothenate synthetase	0.848447	-1.13228	0.774637	-1.11497
A0A140N7R0	D-alanyl-D-alanine carboxypeptidase DacB	0.982041	1.00137	0.561653	-1.03417
A0A140NF80	Nucleoid-associated protein YbaB	0.838325	1.03024	0.488685	1.09389
A0A140N679	1-phosphofructokinase	0.223586	-1.21977	0.65223	-1.06693
A0A140NCM3	tRNA-specific 2-thiouridylase MnmA	0.240841	-1.14911	0.361207	-1.11414
P0ABQ4	Dihydrofolate reductase	0.482349	-1.11816	0.192545	-1.23744
A0A140N4L0	50S ribosomal protein L18	0.87134	-1.37946	0.181747	-6.91256
A0A140NGM4	HTH-type transcriptional regulator UlaR	0.193615	1.357	0.909779	1.02273
A0A140NFE9	Chaperone protein Skp	0.850688	1.18128	0.387276	-1.56221
A0A140NDQ0	Regulator of ribonuclease activity B	0.726601	-1.70884	0.554261	-1.44382
A0A140NCY4	High frequency lysogenization protein HflD	0.690456	1.05825	0.414765	1.11076
A0A140NGG7	30S ribosomal protein S6;30S ribosomal protein S6, fully modified isoform;30S ribosomal	0.951169	-1.02538	0.267952	-2.00218
A0A140N377	Thiosulfate sulfurtransferase GlpE	0.463937	1.10188	0.989837	-1.00148
A0A140NAV7	50S ribosomal protein L16 arginine hydroxylase	0.863859	-1.03406	0.163118	-1.32329
A0A140NBN2		0.350122	1.23889	0.25789	1.30968
A0A140NG90		0.859358	1.0262	0.160567	1.24893
A0A140NAR3	Cysteine--tRNA ligase	0.986025	1.00209	0.158429	-1.19551

A0A140NAV2	Free methionine-R-sulfoxide reductase	0.502606	-1.11347	0.213182	1.22202
A0A140NGF3	Molybdopterin adenylyltransferase	0.776225	-1.0537	0.467841	1.12349
A0A140N6Z2	50S ribosomal protein L16	0.580071	-1.08416	0.188925	-1.22317
A0A140NAU7	Ribosome-recycling factor	0.572032	1.08687	0.272801	-1.16677
A0A140N6Y5	30S ribosomal protein S10	0.610733	-1.22661	0.246417	-1.85773
A0A140N8N3	UTP--glucose-1-phosphate uridylyltransferase	0.218036	1.20451	0.568958	1.08199
A0A140N8J7	dTDP-4-dehydrorhamnose reductase	0.549574	1.05457	0.299527	-1.09392
A0A140NAW2	Cold shock-like protein CspC	0.297547	1.24141	0.478277	-1.13966
A0A140NDD2	Phosphoserine phosphatase	0.388511	1.23953	0.399716	-1.20143
A0A140NBQ7	2-deoxyglucose-6-phosphate phosphatase	0.224264	1.53761	0.486255	-1.28412
A0A140NGV6	Valine--tRNA ligase	0.894311	-1.01698	0.193943	-1.18189
A0A140ND04	Nuclease SbcCD subunit C	0.928981	-1.01189	0.229329	1.16993
A0A140N7L9	30S ribosomal protein S11	0.826851	-1.03335	0.193194	-1.24206
A0A140N7A8	Probable deferrochelatase/peroxidase YfeX	0.261157	1.2068	0.797573	1.03832
A0A140N460	Cell division protein FtsP	0.888209	1.01339	0.594086	-1.04779
A0A140N811	30S ribosomal protein S15	0.865781	1.05243	0.181048	-1.58587
A0A140N942	GTPase Der	0.544548	-1.06546	0.73233	-1.03305
A0A140N7X1	Probable transcriptional regulatory protein YeeN	0.486543	-1.11874	0.533296	-1.09345
A0A140NFU3	30S ribosomal protein S20	0.849522	-1.23232	0.189817	-4.28525
A0A140NDE3	Outer membrane protein A	0.908973	1.02565	0.224238	1.37253
A0A140N7D0	DNA ligase	0.537954	-1.08963	0.271394	-1.16105
A0A140NG17	Right origin-binding protein	0.412629	1.14801	0.408558	-1.16567
A0A140N528	30S ribosomal protein S19	0.643693	-1.05467	0.205566	-1.16315

A0A140N8X3	GTPase Era	0.190449	-1.71108	0.997624	-1.00122
A0A140NGL1	Miniconductance mechanosensitive channel MscM	0.845131	-1.09181	0.764885	-1.09842
A0A140N5N9	Trk system potassium uptake protein TrkA	0.610644	-1.09997	0.938113	1.01236
A0A140N5S4	Sulfur acceptor protein CsdE	0.324008	-1.13266	0.616196	-1.05933
A0A140NC11	Peptidoglycan-associated lipoprotein	0.535329	1.06048	0.420174	1.08568
A0A140N6T7	50S ribosomal protein L19	0.474335	-1.18327	0.508673	-1.20325
A0A140N919	UPF0070 protein YfgM	0.400162	1.1338	0.511145	-1.09317
A0A140NAP1	Uncharacterized protein YehS	0.366851	-1.20475	0.757651	-1.05586
A0A140N5I1	GTP cyclohydrolase 1	0.439401	1.32619	0.29356	1.43275
A0A140N742	Protease HtpX	0.294789	-1.17137	0.61547	1.08255
A0A140N666	Valine--pyruvate aminotransferase	0.28346	-1.19289	0.458015	1.13143
A0A140N4H6	Thiol:disulfide interchange protein DsbC	0.463617	1.26762	0.75737	-1.08173
A0A140N8N4	Phenylalanine--tRNA ligase beta subunit	0.248609	1.17121	0.720789	-1.04583
A0A140NG07	Protein RdoA	0.911577	1.02719	0.271632	-1.28062
A0A140NFJ6	Nicotinate-nucleotide pyrophosphorylase [carboxylating]	0.86366	-1.02046	0.530691	-1.07052
A0A140NFG9	3-octaprenyl-4-hydroxybenzoate carboxy-lyase	0.344687	1.14379	0.359961	-1.13925
A0A140NDD0	Thymidylate kinase	0.543412	1.0486	0.34901	1.07423
A0A140NB23	Peptidyl-tRNA hydrolase	0.680656	-1.04786	0.446312	-1.08448
A0A140NFE1		0.210043	-1.15802	0.847586	-1.02136
A0A140N8H7		0.484367	1.45324	0.764868	1.29813
A0A140N763	Protein YhgF	0.952019	1.00755	0.265654	-1.14732
A0A140N7K3	Acetyl-coenzyme A carboxylase carboxyl transferase subunit beta	0.688908	1.05135	0.276758	-1.14401
A0A140N8U7	DNA translocase FtsK	0.755785	1.05672	0.550049	-1.09858

A0A140N7B9	Exodeoxyribonuclease III	0.885979	1.01298	0.260131	-1.10777
A0A140NEF5	Bifunctional protein FolD;Methylenetetrahydrofolate dehydrogenase;Methenyltetrahydrofol	0.966157	1.00987	0.968441	-1.0076
A0A140NDM9	Protein GnsA	0.547975	1.24592	0.795931	1.07575
A0A140N560	NADH-quinone oxidoreductase subunit B	0.278081	-1.12381	0.617154	-1.0524
A0A140N6D6		0.61491	-1.17168	0.286686	1.38594
A0A140N6C6	Elongation factor 4	0.785869	1.04007	0.246802	-1.19465
A0A140NCA6	tRNA sulfurtransferase	0.312188	-1.26161	0.650004	-1.11561
A0A140N908	Membrane-bound lytic murein transglycosylase B	0.976911	-1.00379	0.670404	-1.05196
A0A140NCH9	DNA-binding protein H-NS	0.762261	-1.0922	0.436536	1.21648
A0A140NC04	Tellurite methyltransferase	0.821794	-1.06554	0.273194	1.35268
A0A140N6S0	2-C-methyl-D-erythritol 2,4-cyclodiphosphate synthase	0.791794	-1.03039	0.346478	-1.1098
A0A140N6I8	Murein hydrolase activator EnvC	0.390833	1.09673	0.566108	1.06012
A0A140N896	Ribosomal large subunit pseudouridine synthase C	0.96406	1.01964	0.264767	-1.76059
A0A140NCB5	Transcriptional repressor NrdR	0.415374	-1.11184	0.995633	1.00065
A0A140N8Y3	tRNA threonylcarbamoyladenosine biosynthesis protein TsaB	0.653691	-1.21029	0.32152	-1.47378
A0A140N8B3	Protease 3	0.260275	1.19896	0.898508	-1.01944
A0A140N9Z5	Cell division protein ZipA	0.987759	-1.00242	0.991038	1.00156
A0A140NHR0	Protein MioC	0.281374	-1.10224	0.7664	1.02588
A0A140NEP3	Mechanosensitive channel MscK	0.44987	-1.15934	0.543073	-1.11485
A0A140N9X7		0.438262	-1.11789	0.845437	1.03042

A0A140NBC7	Chromosome partition protein MukE	0.758561	1.07433	0.44434	1.17393
A0A140N9H6	Protein YdgH	0.972053	1.00461	0.865774	1.02027
A0A140NB69	Integration host factor subunit alpha	0.625162	1.21775	0.474	1.27288
A0A140NCE2	Pyrroline-5-carboxylate reductase	0.711904	1.09366	0.492511	-1.15892
A0A140SS63	50S ribosomal protein L7/L12	0.891719	1.0236	0.290638	-1.21526
A0A140N2U0	DNA-directed RNA polymerase subunit alpha	0.836335	-1.02437	0.305304	-1.12783
A0A140NA26	Lipopolysaccharide core heptose(II)-phosphate phosphatase	0.511913	1.15463	0.906103	1.02261
A0A140N7N2	Methionyl-tRNA formyltransferase	0.74555	1.04218	0.469923	-1.09111
A0A140N548	30S ribosomal protein S4	0.655202	-1.07928	0.425928	-1.1372
A0A140NBK6	Protease 4	0.887022	1.02379	0.313759	-1.18042
A0A140SS30	Elongation factor P	0.658319	1.0412	0.485839	1.06968
P62517	Glucans biosynthesis glucosyltransferase H	0.973032	1.00628	0.305477	1.22771
A0A140NBV0	Peptidoglycan synthase FtsI	0.831027	1.04131	0.794913	-1.04424
A0A140NGD5	4-hydroxy-3-methylbut-2-enyl diphosphate reductase	0.632537	-1.09402	0.893569	1.02236
A0A140NFA9	DNA-binding protein HU-beta	0.980243	1.01794	0.99342	-1.00381
A0A140N983	Arginine--tRNA ligase	0.577021	-1.09036	0.382431	-1.15243
A0A140N8W6	Bifunctional protein Aas;2acylglycerophosphoethanolamine acyltransferase;Acyl-[acyl-ca	0.634646	1.11296	0.804092	-1.04948
A0A140N7A0	Ribonucleoside-diphosphate reductase 1 subunit beta	0.751106	1.04016	0.571847	-1.0679
A0A140N5V8	Phosphomannomutase	0.549149	-1.11732	0.751359	1.05382
A0A140N9K4	NADH-quinone oxidoreductase subunit E	0.401877	-1.16493	0.536646	-1.11817

A0A140N4F0	DNA polymerase III subunit beta	0.550347	-1.0877	0.529781	1.08724
A0A140N9E8	Putative Lon protease homolog	0.725714	-1.03936	0.340047	-1.11486
A0A140N7J1	50S ribosomal protein L2	0.666569	-1.10463	0.84914	-1.0528
A0A140N3A3	ADP compounds hydrolase NudE	0.422452	1.06413	0.612805	1.03871
A0A140N846	50S ribosomal protein L25	0.600845	1.04477	0.659762	-1.0358
A0A140N884	Aminodeoxychorismate lyase	0.922425	1.04206	0.519065	-1.25372
A0A140SS82	UDP-N-acetylenolpyruvoylglucosamine reductase	0.642093	-1.07161	0.662824	-1.06181
CON__P35908		0.46013	-1.79392	0.593791	-1.40466
A0A140NBS2	2-iminobutanoate/2-iminopropanoate deaminase	0.967514	-1.03201	0.641982	1.27775
A0A140NAN4	Chain length determinant protein	0.748787	-1.04099	0.408968	-1.11606
A0A140N332	Probable phospholipid import ATP-binding protein MlaF	0.600769	1.101	0.598477	1.09335
A0A140NEV3	1-deoxy-D-xylulose-5-phosphate synthase	0.67046	1.05287	0.691101	-1.04601
A0A140NDB6	50S ribosomal protein L10	0.668771	-1.05478	0.796464	-1.03523
A0A140N3T4	Translation initiation factor IF-2	0.907911	1.01016	0.366752	-1.08294
A0A140NAR5	Ribosome-binding ATPase YchF	0.786931	-1.03799	0.707665	-1.04909
A0A140NE42	LPS-assembly lipoprotein LptE	0.945347	-1.04392	0.504516	-1.39163
A0A140NGW9	Aspartate carbamoyltransferase catalytic chain	0.744603	1.15762	0.457679	1.49237
A0A140NEC6	Aspartate carbamoyltransferase regulatory chain	0.621305	1.2339	0.970837	-1.02036
A0A140NCA7	GTP cyclohydrolase-2	0.506953	-1.11525	0.590092	1.08829
CON__P13645		0.832869	-1.1079	0.506842	-1.51425
A0A140N5Y7	Ribosome-binding factor A	0.673272	1.03526	0.484904	-1.06102

A0A140NDD6	1-deoxy-D-xylulose 5-phosphate reductoisomerase	0.55653	-1.17829	0.716444	1.09473
A0A140N7D6	Transcription termination/antitermination protein NusA	0.98758	1.00212	0.382239	-1.13064
A0A140ND34	Release factor glutamine methyltransferase	0.851824	-1.02505	0.393201	1.12205
A0A140NFD7	Elongation factor Ts	0.803436	-1.05607	0.527643	-1.16637
A0A140N601	ADP-ribose pyrophosphatase	0.41717	1.15028	0.943239	-1.01163
A0A140ND77	3-deoxy-manno-octulosonate cytidylyltransferase	0.910644	-1.02176	0.524494	1.12135
A0A140NEN6	10 kDa chaperonin	0.727954	1.20241	0.426116	-1.54777
A0A140NBZ3	Modulator of FtsH protease HflK	0.434938	1.19024	0.709548	1.08627
A0A140NGA3	UPF0438 protein YifE	0.841205	1.13874	0.407548	-1.82821
A0A140NDA6	Thioredoxin reductase	0.425386	1.08377	0.795772	-1.02611
A0A140N9I4	Tetraacyldisaccharide 4-kinase	0.529841	-1.11521	0.889694	-1.02255
A0A140N472	Protein-L-isoaspartate O-methyltransferase	0.784042	1.05569	0.964724	1.00788
A0A140N8R1	Transcriptional regulatory protein BaeR	0.679501	1.08706	0.715344	-1.07002
A0A140N4E7	Small-conductance mechanosensitive channel	0.722768	1.05569	0.942122	-1.01029
A0A140N6W8	30S ribosomal protein S7	0.493986	-1.15574	0.765946	1.06721
A0A140N7P3	RNA polymerase sigma-54 factor	0.694928	1.10699	0.553946	1.15538
A0A140N3N3	tRNA (guanine-N(7)-)-methyltransferase	0.799209	-1.04093	0.455338	-1.12917
A0A140N6R8	Cell division protein DamX	0.980461	1.01583	0.618875	-1.29108
A0A140NDV1	50S ribosomal protein L9	0.616159	-1.09418	0.901304	-1.02406
CON__P35527		0.894834	1.06833	0.503516	1.36442
A0A140NEN1	Uncharacterized protein YbcJ	0.672695	-1.21866	0.872338	-1.06448
A0A140N7Q7	Transcriptional regulatory protein RstA	0.618239	-1.12766	0.747629	1.08704

A0A140NEF6	Inorganic pyrophosphatase	0.535846	-1.2434	0.826695	-1.07297
A0A140NEL3	Oligoribonuclease	0.832097	-1.2307	0.705544	-1.31009
A0A140N4H3	30S ribosomal protein S12	0.705693	-1.42225	0.666086	-1.37748
A0A140N9Q6	Uncharacterized symporter YdjN	0.642746	-1.09358	0.666002	1.08429
A0A140N885	NADH-quinone oxidoreductase subunit I	0.955794	-1.00848	0.739643	1.04905
A0A140NDC7	Protein translocase subunit SecD	0.592532	1.07683	0.755037	-1.04319
A0A140NB91	Helicase IV	0.791576	1.03771	0.995808	-1.00069
A0A140N5Z1	Murein hydrolase activator NlpD	0.940492	-1.04249	0.696193	-1.20375
A0A140N3M9	Serine endoprotease DegS	0.92824	-1.02425	0.931421	1.02083
A0A140SSC0	Thioredoxin-1	0.895611	-1.04063	0.647803	-1.13921
A0A140NG05	UPF0325 protein YaeH	0.893868	1.07749	0.972465	-1.01591
A0A140NAW6	Glucose-1-phosphate thymidylyltransferase 1	0.841408	1.04845	0.65573	1.10693
A0A140N4T2	Low-affinity inorganic phosphate transporter 1	0.899939	-1.02954	0.631746	1.11809
A0A140N6J6	Histidine--tRNA ligase	0.883614	1.01613	0.907315	-1.01323
A0A140N9X9	Glutaredoxin-4	0.904568	1.0123	0.819394	1.02298
A0A140NI98	ATP synthase subunit b	0.935347	-1.02446	0.912267	1.03666
A0A140NEJ9	HTH-type transcriptional repressor AllR	0.835839	1.05871	0.767086	1.08104
A0A140N7T1	Succinyl-diaminopimelate desuccinylase	0.70609	1.04337	0.926452	1.01028
CON__P04264		0.942137	-1.0364	0.741466	1.16654
A0A140NDH6	UPF0434 protein YcaR	0.958948	-1.01719	0.731105	1.12512
A0A140N731	Cytoskeleton protein RodZ	0.90812	1.03236	0.984675	-1.00504
A0A140NBA0	Probable transcriptional regulatory protein YebC	0.93888	1.02121	0.954028	1.01587

Supplementary Table 4. Structural records obtained from Blastp of rARU against PDB database.

Accession	*Description*	*Max score*	*Total score*	*Query cover*	*E value*	*Identity*
4NP4_A	Chain A, Clostridium difficile toxin B CROP domain in complex with FAB domains of neutralizing antibody bezlotoxumab	204	1298	99%	2.00E-59	47%
6AR6_A	Chain A, Clostridioides difficile toxinB with DLD-4 darpin	202	873	99%	5.00E-53	47%
5UMI_C	Chain C, Clostridium difficile TcdA-CROPs bound to PA50 Fab	506	1910	99%	6.00E-175	100%
2G7C_A	Chain A, Clostridium Difficile Toxin A Fragment Bound To Agal(1,3)bgal(1,4) Bglcnac	487	1892	99%	1.00E-167	95%
4NBY_A	Chain A, Crystal Structure Of Tcda-a2 Bound To Two Molecules Of A20.1 Vhh	485	1722	99%	6.00E-167	94%
4NC0_A	Chain A, Crystal Structure Of Tcda-a2 Bound To A26.8 Vhh	476	1870	99%	3.00E-163	94%
4NBZ_A	Chain A, Crystal Structure Of Tcda-a1 Bound To A26.8 Vhh	265	1378	99%	8.00E-84	94%
4NBX_A	Chain A, Crystal Structure Of Clostridium Difficile Toxin A Fragment Tcda-a1 Bound To A20.1 Vhh	265	1376	99%	1.00E-83	94%
5NGY_A	Chain A, Crystal structure of Leuconostoc citreum NRRL B1299 dextransucrase DSR-M	65.5	493	99%	3.00E-10	29%
5LFC_A	Chain A, Crystal Structure Of Leuconostoc Citreum Nrrl B-1299 N-terminally Truncated Dextransucrase Dsr-m	65.1	492	99%	3.00E-10	29%
2BIB_A	Chain A, Crystal Structure Of The Complete Modular Teichioic Acid Phosphorylcholine Esterase Pce (Cbpe) From Streptococcus Pneumoniae	57	587	99%	6.00E-08	25%
2QJ6_A	Chain A, Crystal Structure Analysis Of A 14 Repeat C-Terminal Fragment Of Toxin Tcda In Clostridium Difficile	643	2255	98%	0	100%
2V04_A	Chain A, CRYSTAL STRUCTURE OF CHOLINE BINDING PROTEIN F FROM STREPTOCOCCUS PNEUMONIAE	76.6	674	96%	1.00E-14	26%
2VYU_A	Chain A, Crystal Structure Of Choline Binding Protein F From Streptococcus Pneumoniae In The Presence Of A Peptidoglycan Analogue (Tetrasaccharide-Pentapeptide)	76.6	610	96%	2.00E-14	26%
2F6E_A	Chain A, Clostridium Difficile Toxin A C-Terminal Fragment 1 (Tcda-F1)	247	1103	95%	3.00E-77	95%

4IWT_A	Chain A, Crystal Structure Of The C-teminal Choline-binding Domain Of The Streptococcus Pneumoniae Prophage Lyta	43.1	384	84%	2.00E-04	26%
4NC2_A	Chain A, Crystal Structure Of Tcdb-b1 Bound To B39 Vhh	88.6	720	83%	2.00E-20	55%
2BML_A	Chain A, Ofloxacin-Like Antibiotics Inhibit Pneumococcal Cell Wall Degrading Virulence Factors	45.1	432	79%	3.00E-05	35%
1GVM_A	Chain A, Choline Binding Domain Of The Major Autolysin (CLyta) From Streptococcus Pneumoniae	45.4	401	75%	3.00E-05	35%
1HCX_A	Chain A, Choline Binding Domain Of The Major Autolysin (CLyta) From Streptococcus Pneumoniae	45.1	399	75%	3.00E-05	35%
4TVD_A	Chain A, N-terminally Truncated Dextransucrase Dsr-e From Leuconostoc Mesenteroides Nrrl B-1299 In Complex With Dglucose	42	113	74%	0.004	27%
4TTU_A	Chain A, N-terminally Truncated Dextransucrase Dsr-e From Leuconostoc Mesenteroides Nrrl B-1299 In Complex With Isomaltotriose	42	113	74%	0.004	27%
3TTO_A	Chain A, Crystal structure of Leuconostoc mesenteroides NRRL B-1299 N-terminally truncated dextransucrase DSR-E in triclinic form	42	113	74%	0.004	27%
4X36_A	Chain A, Crystal Structure Of The Autolysin Lyta From Streptococcus Pneumoniae Tigr4	47	278	71%	6.00E-05	27%
3HIA_A	Chain A, Crystal Structure Of The Choline Binding Domain Of Spr1274 In Streptococcus Pneumoniae	45.8	505	63%	9.00E-06	36%
1H09_A	Chain A, Multimodular Pneumococcal Cell Wall Endolysin From Phage Cp-1	44.7	273	58%	3.00E-04	29%
1OBA_A	Chain A, Multimodular Pneumococcal Cell Wall Endolysin From Phage Cp-1 Complexed With Choline	44.7	273	58%	3.00E-04	29%
2IXV_A	Chain A, Crystal Structure Of The Modular Cpl-1 Endolysin Complexed With A Peptidoglycan Analogue (E94q Mutant)	44.7	273	58%	3.00E-04	29%
2IXU_A	Chain A, Crystal structure of the modular Cpl-1 endolysin complexed with a peptidoglycan analogue (wild-type endolysin)	44.7	273	58%	3.00E-04	29%

1H8G_A	Chain A, C-Terminal Domain Of The Major Autolysin (C-Lyta) From Streptococcus Pneumoniae	38.9	206	44%	0.003	29%

Supplementary Table 5:

S5a) ScanProsite analysis of the rARU protein sequence expressed in this study.

Input Protein Sequence rARU (865 AA)

```
IEFNLVTGWQTINGKKYYFDINTGAALTSYKIINGKHFYFNNDGVMQLGVFKGPDGFEYFAPANTQ
NNNIEGQAIVYQSKFLTLNGKKYYFDNNSKAVTGWRIINNEKYYFNPNNAIAAVGLQVIDNNKYYF
NPDTAIISKGWQTVNGSRYYFDTDTAIAFNGYKTIDGKHFYFDSDCVVKIGVFSTSNGFEYFAPAN
TYNNNIEGQAIVYQSKFLTLNGKKYYFDNNSKAVTGWQTIDSKKYYFNTNTAEAATGWQTIDGKKY
YFNTNTAEAATGWQTIDGKKYYFNTNTAIASTGYTIINGKHFYFNTDGIMQIGVFKGPNGFEYFAP
ANTDANNIEGQAILYQNEFLTLNGKKYYFGSDSKAVTGWRIINNKKYYFNPNNAIAAIHLCTINND
KYYFSYDGILQNGYITIERNNFYFDANNESKMVTGVFKGPNGFEYFAPANTHNNNIEGQAIVYQNK
FLTLNGKKYYFDNDSKAVTGWQTIDGKKYYFNLNTAEAATGWQTIDGKKYYFNLNTAEAATGWQTI
DGKKYYFNTNTFIASTGYTSINGKHFYFNTDGIMQIGVFKGPNGFEYFAPANTDANNIEGQAILYQ
NKFLTLNGKKYYFGSDSKAVTGLRTIDGKKYYFNTNTAVAVTGWQTINGKKYYFNTNTSIASTGYT
IISGKHFYFNTDGIMQIGVFKGPDGFEYFAPANTDANNIEGQAIRYQNRFLYLHDNIYYFGNNSKA
ATGWVTIDGNRYYFEPNTAMGANGYKTIDNKNFYFRNGLPQIGVFKGSNGFEYFAPANTDANNIEG
QAIRYQNRFLHLLGKIYYFGNNSKAVTGWQTINGKVYYFMPDTAMAAAGGLFEIDGVIYFFGVDGV
KAPGIYG
```

hits by profiles: [31 hits (by 1 profile) on 1 sequence]

PS51170 CW Cell wall-binding repeat profile

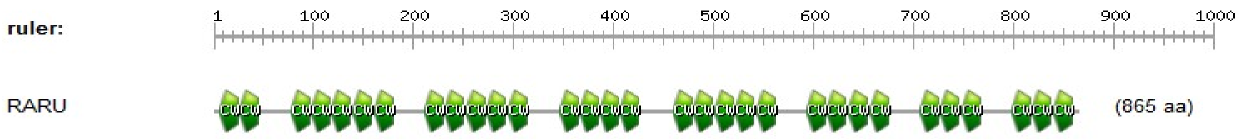

Supplementary Table 5:

S5b) 15 sequences containing cell wall binding repeat profiles (CW, PS51170) found so far in other proteins through ScanProsite.

Organism	gene	Protein	No of cell wall binding repeats
Clostridioides difficile	*toxA*	Toxin A	32
Clostridioides difficile	*toxB*	Toxin B	18
Streptococcus downei	*gtfI*	Glucosyltransferase-I	13
Streptococcus pneumoniae	*lytB*	Putative endo-beta-Nacetylglucosaminidase	15
Streptococcus mutans serotype c (strain ATCC 700610 / UA159)	*gtfD*	Glucosyltransferase-S	14
Streptococcus mutans serotype c (strain ATCC 700610 / UA159)	*gtfB*	Glucosyltransferase-I	13
Streptococcus downei (Streptococcus sobrinus)	N/A	Glucosyltransferase-I	13
Streptococcus pneumoniae serotype 4 (strain ATCC BAA334 / TIGR4)	*lytB*	Putative endo-beta-Nacetylglucosaminidase	13
Streptococcus downei (Streptococcus sobrinus)	*gtfS*	Glucosyltransferase-S	12
Streptococcus mutans serotype c (strain ATCC 700610 / UA159)	*gtfC*	Glucosyltransferase-SI	11
Pneumococcus phage Dp-1 (Bacteriophage Dp-1)	PAL	Lysin	6
Streptococcus pneumoniae serotype 4 (strain ATCC BAA334 / TIGR4)	*lytA*	Autolysin	6
Streptococcus pneumoniae phage HB-3	HBL	Lytic amidase	6
Streptococcus phage Cp-1 (Bacteriophage Cp-1)	CPL1	Lysozyme	5
Streptococcus phage Cp-9 (Bacteriophage Cp-9)	CPL9	Lysozyme	5

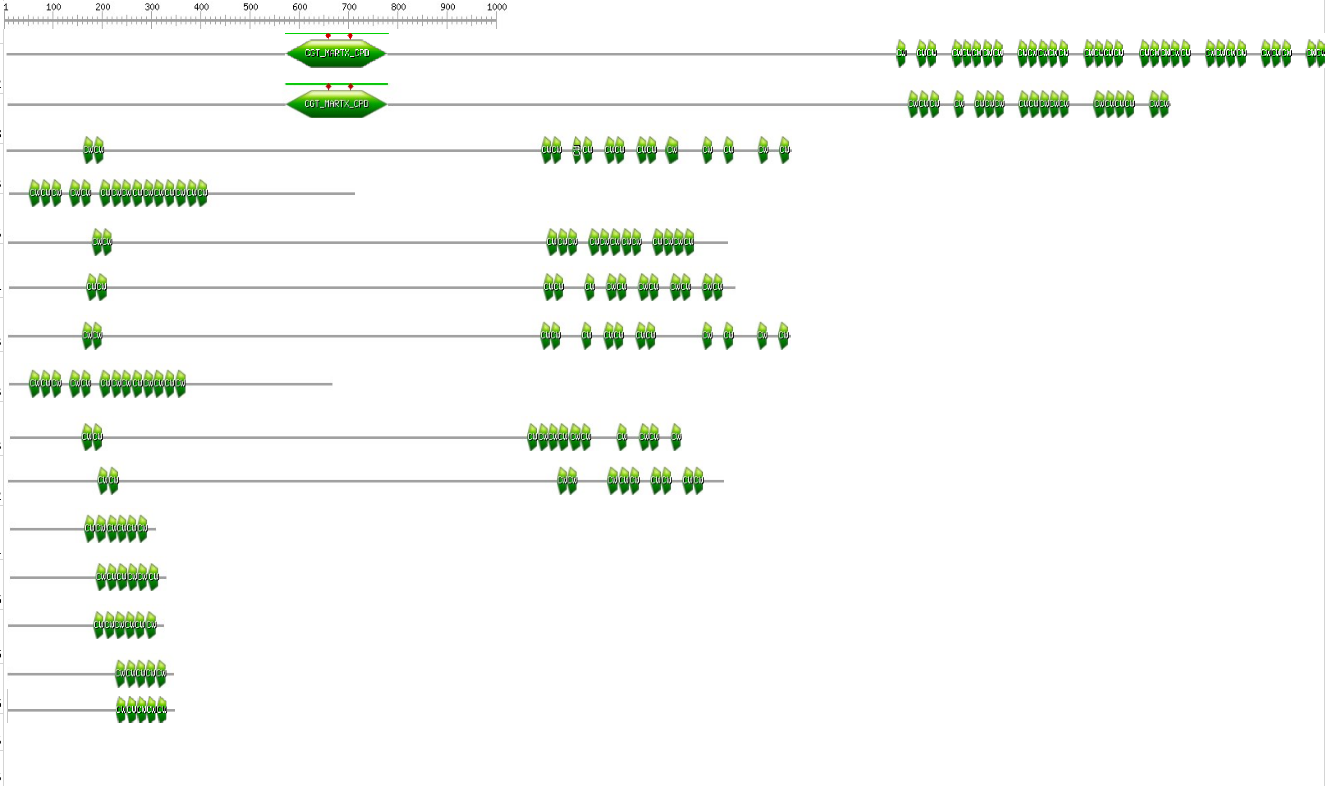

Supplement: Supplementary file 1 — Supplementary file. [file 41598_2020_59978_MOESM1_ESM.pdf]
